# The still mysterious roles of cysteine-containing glutathione transferases in plants

**DOI:** 10.3389/fphar.2014.00192

**Published:** 2014-08-20

**Authors:** Pierre-Alexandre Lallement, Bastiaan Brouwer, Olivier Keech, Arnaud Hecker, Nicolas Rouhier

**Affiliations:** ^1^UMR1136, Interactions Arbres - Microorganismes, Université de LorraineVandoeuvre-lès-Nancy, France; ^2^UMR1136, Interactions Arbres - Microorganismes, INRAChampenoux, France; ^3^Department of Plant Physiology, Umeå Plant Science Centre, Umeå UniversityUmeå, Sweden

**Keywords:** cysteines, deglutathionylation, glutathione transferases, photosynthetic organisms, phylogeny

## Abstract

Glutathione transferases (GSTs) represent a widespread multigenic enzyme family able to modify a broad range of molecules. These notably include secondary metabolites and exogenous substrates often referred to as xenobiotics, usually for their detoxification, subsequent transport or export. To achieve this, these enzymes can bind non-substrate ligands (ligandin function) and/or catalyze the conjugation of glutathione onto the targeted molecules, the latter activity being exhibited by GSTs having a serine or a tyrosine as catalytic residues. Besides, other GST members possess a catalytic cysteine residue, a substitution that radically changes enzyme properties. Instead of promoting GSH-conjugation reactions, cysteine-containing GSTs (Cys-GSTs) are able to perform deglutathionylation reactions similarly to glutaredoxins but the targets are usually different since glutaredoxin substrates are mostly oxidized proteins and Cys-GST substrates are metabolites. The Cys-GSTs are found in most organisms and form several classes. While Beta and Omega GSTs and chloride intracellular channel proteins (CLICs) are not found in plants, these organisms possess microsomal ProstaGlandin E-Synthase type 2, glutathionyl hydroquinone reductases, Lambda, Iota and Hemerythrin GSTs and dehydroascorbate reductases (DHARs); the four last classes being restricted to the green lineage. In plants, whereas the role of DHARs is clearly associated to the reduction of dehydroascorbate to ascorbate, the physiological roles of other Cys-GSTs remain largely unknown. In this context, a genomic and phylogenetic analysis of Cys-GSTs in photosynthetic organisms provides an updated classification that is discussed in the light of the recent literature about the functional and structural properties of Cys-GSTs. Considering the antioxidant potencies of phenolic compounds and more generally of secondary metabolites, the connection of GSTs with secondary metabolism may be interesting from a pharmacological perspective.

## Introduction

Glutathione is a tripeptide with the sequence γGlu-Cys-Gly that is mostly present in reduced (GSH) or disulfide (GSSG) forms, even though nitrosoglutathione (GSNO) may represent another important source. Under physiological conditions, free glutathione is present in concentrations ranging from 1 to 10 mM with the reduced form largely predominating over the oxidized form (Gutscher et al., 2008; Pallardo et al., 2009). As such, glutathione is the major non-protein thiol source in eukaryote cells, likely constituting a crucial redox buffer (Rouhier et al., 2008). Glutathione can also fulfill additional roles. In eukaryotes, glutathione is essential for a proper development, controlling in particular cell-cycle progression. Apart from development, glutathione is crucial for stress response by (i) neutralizing radicals, (ii) participating in heavy metal tolerance, either directly or as a constitutive element of phytochelatins, (iii) contributing to the regeneration of antioxidant molecules such as ascorbate and α-tocopherol, and (iv) providing electrons and protons to glutathione transferases (GSTs) or to peroxiredoxins, both with and without the involvement of glutaredoxins (Grxs) for peroxide removal (Rouhier et al., 2008). While the exact role of glutathione has not been completely defined, it is clear that, depending on its redox state, glutathione can react with various intracellular molecules and that glutathionylation/deglutathionylation reactions of both proteins and smaller compounds are central to GSH functions. Protein glutathionylation is a reversible post-translational modification that is now recognized as a major signaling or protective mechanism. It occurs under basal non-stress conditions but has mostly been documented in response to oxidative stress conditions (Zaffagnini et al., 2012). The reversible reaction i.e., deglutathionylation, occurs either by the intervention of Grxs or by direct thiol/disulfide exchange reactions with GSH once an appropriate GSH/GSSG ratio has been restored.

In addition to proteins, glutathionylation of metabolites has also attracted a lot of attention as it constitutes an intermediate step in a number of metabolic processes and detoxification pathways. It has been well-established that most organisms possess a three-step detoxification system to eliminate endogenous and exogenous toxic compounds (Coleman et al., 1997; Morel et al., 2013). In the first step, enzymes such as cytochrome P450 monooxygenases catalyze various reactions (oxidation, reduction or hydrolysis) to expose or introduce a functional moiety on hydrophobic substrates. In the second step, conjugating enzymes perform addition reactions (e.g., acetyl, methyl, glucuronic acid) on these newly modified, electrophilic substrates. The glutathione addition onto electrophilic molecules is well-recognized and is mediated by specific classes of GSTs having usually a serine or a tyrosine as a catalytic residue. Finally, glutathionylated products are either exported from the cells or sequestered in vacuoles. In plants, GSTs have been identified by showing glutathionylation of the herbicide atrazine (Lamoureux et al., 1970). Most subsequent studies have focused on these GST types that are here referred to as glutathionylating GSTs, although other biochemical activities have been described for some GST classes. To cite a few, numerous GSTs exhibit GSH-dependent peroxidase activities reducing simple peroxides but also organic hydroperoxides (Tang and Tu, 1994; Marrs, 1996; Hurst et al., 1998). Theta GSTs were shown to catalyze the isomerization of maleylacetoacetate into fumarylacetoacetate, a key component of the catabolism of tyrosine and phenylalanine (Thom et al., 2001; Fernandez-Canon et al., 2002). Besides these catalytic functions, GSTs could also exhibit ligandin functions, binding hydrophobic substrates in a so-called L-site for transport and storage purposes. In plants, it has been documented that GSTs with ligandin properties are not only implicated in the transport of anthocyanins and flavonoids but also of hormones such as auxin and cytokinin, which suggests a possible role in cell signaling (Smith et al., 2003; Kitamura et al., 2004; Moons, 2005).

With the increasing number of biochemical studies, it became clear that several GSTs do not have a glutathionylation activity but instead catalyze the opposite reaction; deglutathionylation (Dixon and Edwards, 2010a,b; Xun et al., 2010; Board, 2011; Meux et al., 2011). This capacity usually originates from the replacement of the catalytic serine or tyrosine residues in the active site motif by a cysteinyl residue as demonstrated for mammalian, insect and fungal Omega GSTs (GSTOs) (Board et al., 2000; Kim et al., 2006; Yamamoto et al., 2009; Meux et al., 2013), for plant Lambda GSTs (GSTLs) (Dixon and Edwards, 2010b; Lallement et al., 2014) and for bacterial and fungal glutathionyl hydroquinone reductases (GHRs) (Xun et al., 2010; Meux et al., 2011). However, the physiological functions of these enzymes have rarely been elucidated. Human GSTOs may be involved in arsenic biotransformation, reducing methyl and dimethyl arsenate (Zakharyan et al., 2001; Burmeister et al., 2008), whereas plant GSTLs may be involved in flavonoid metabolism and/or trafficking (Dixon and Edwards, 2010b). Interestingly, while the role of GSTs has classically been associated to the modification of small molecules and the role of glutaredoxins to the deglutathionylation of proteins, it has recently been shown that human GSTO1-1 can deglutathionylate β-actin, which should prompt us to consider proteins as GST substrates (Menon and Board, 2013). Concerning GHRs, the bacterial and fungal members characterized so far are involved in the catabolism of chlorinated quinones and in lignin degradation through the deglutathionylation of glutathionylated intermediates (Reddy and Gold, 2001; Masai et al., 2003; Huang et al., 2008; Meux et al., 2011). While these glutathionylated compounds constitute intermediates in catabolism pathways, they may also constitute intermediates for biosynthetic pathways as shown for sulfur-containing defense molecules such as camalexins or glucosinolates (Su et al., 2011). However, the roles of GSTs in the secondary metabolism are less documented compared to the ones of cytochrome P450 monooxygenases and to their involvement in xenobiotic detoxification. A plausible explanation is that intracellular GSH-conjugated compounds have rarely been successfully identified from plant extracts, possibly due to their transient nature or to the difficulty to isolate them. As examples, glutathionylated compounds have been identified as precursors of aromas in fruits (Fedrizzi et al., 2012; Peña-Gallego et al., 2012) or as conjugated oxylipins upon leaf infiltration of keto-fatty acids (Davoine et al., 2005).

Based on the biochemical properties of GSTs and their functional association with cytochrome P450 monooxygenases, acquiring fundamental knowledge about GST functions, regulation and substrates may be beneficial for diverse pharmaceutical and biotechnological applications. In biotechnology, the ability of some GSTs to catalyze GSH-conjugation reactions has been exploited not only for the development of sensitive biosensors or enzyme assays for the determination of the concentration of various pesticides and herbicides (Chronopoulou and Labrou, 2009) but also for the development of herbicide and stress-tolerant plants. Moreover, among the thousands of natural plant products, including polyphenols, flavonoids, alkaloids, and quinones, several molecules possess antimicrobial, anticarcinogenic, anti-inflammatory, or antioxidant properties (Lewis and Ausubel, 2006; Saleem et al., 2010) not to speak about unidentified or untested molecules. Although they are often relatively low abundant molecules, they also constitute a recognized source of molecules important for the cosmetic industry (fragrance) or for nutrition (gustatory perception/dietary complements) contributing to extend the color or aroma palette. For instance, GSTs, notably those from the Pi and Alpha classes, are known to be present in the olfactory epithelium and particularly in the covering mucus layer, where they would serve for metabolizing odorant molecules (Aceto et al., 1993; Debat et al., 2007). Overall, by recognizing and eventually modifying a wide range of antioxidant molecules, GSTs could represent promising enzymes in diagnosis and monitoring cancer invasion, liver, kidney, Alzheimer's and Parkinson's diseases (Chronopoulou and Labrou, 2009). They also have a considerable interest for isolating new secondary metabolites or for developing molecules (drugs or antimicrobial compounds) with different or improved pharmacological properties. As an example, Canfosfamide (TLK286, TELCYTA®), a cancer cell-activated prodrug, was designed to exploit the elevated levels and the activity of glutathione S-transferase P1-1 (GSTP1-1) that is overexpressed in many human cancer cells (Tew, 2005). Hence, GSTs could be useful for product transformation but also for synthetic biology or metabolic engineering approaches, with the aim of generating new chemical entities.

Over the past years, the GST classification has constantly evolved, notably due to the increase of genomic data and to the presence of particular isoforms in a specific subset of organisms. The objective of this review is to present an overview of cysteine-containing GST (Cys-GST) classes in photosynthetic organisms by describing known data concerning the gene expression, the protein subcellular localization and their biochemical and structural properties.

### The GST family in photosynthetic organisms

**Figure 1:**
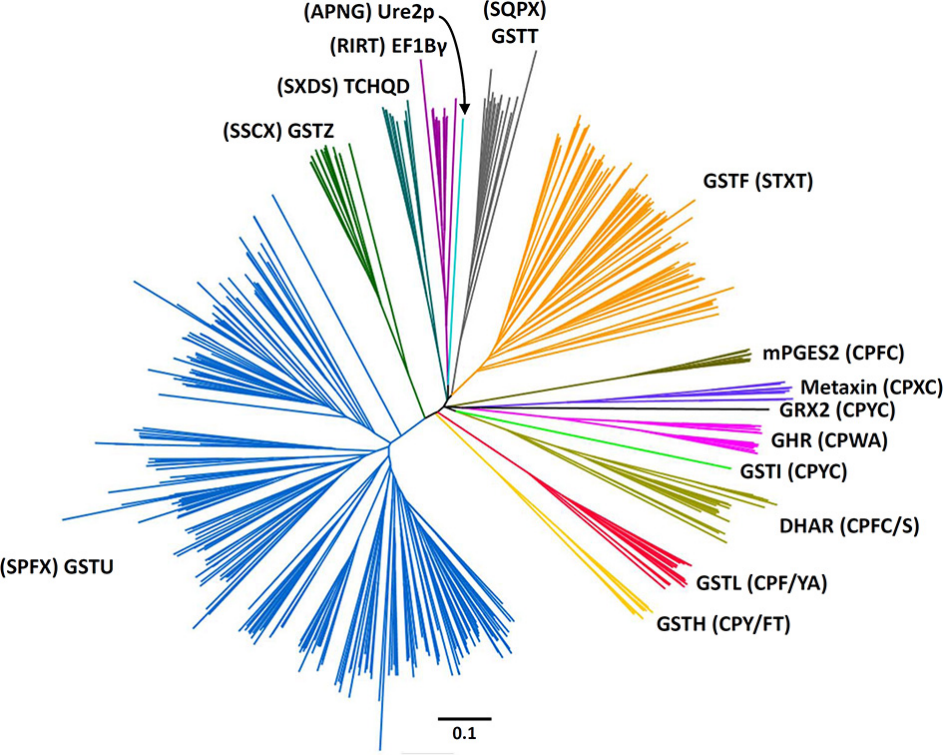
**Rooted phylogenetic tree of plant GSTs**. The sequences used are those identified in *Arabidopsis thaliana* (Lan et al., 2009), *Hordeum vulgare* (Rezaei et al., 2013), *Oryza sativa* (Lan et al., 2009), *Physcomitrella patens* (Liu et al., 2013), *Pinus tabulaeformis* (Lan et al., 2013), *Populus trichocarpa* (Lan et al., 2009), and *Solanum lycopersicum* (Csiszar et al., 2014). Sequences were aligned with PROMALS3D and alignment manually adjusted with Seaview software (Gouy et al., 2010). The phylogenetic tree was constructed with BioNJ (Gascuel, 1997) in Seaview, rooted with *E. coli* glutaredoxin 2 and edited with Figtree software (http://tree.bio.ed.ac.uk/software/figtree/). The robustness of the branches was assessed by the bootstrap method with 500 replications. Various classes can be distinguished: Dehydroascorbate reductase (DHAR), Elongation factor 1Bγ (EF1Bγ), Glutathionyl hydroquinone reductase (GHR), Phi (GSTF), Hemerythrin (GSTH), Iota (GSTI), Lambda (GSTL), Theta (GSTT), Tau (GSTU), Zeta (GSTZ), Microsomal prostaglandin E synthase type 2 (mPGES-2), Tetrachloro-hydroquinone dehalogenase (TCHQD), and Ure2p. The scale marker represents 0.1 substitutions per residue. For clarity, the names of the sequences have not been indicated but all sequences are available in the Supplementary Material.

The present phylogenetic analysis focuses on photosynthetic organisms and as a basis uses the Cd00570 Sequence Cluster of the “conserved domains” tool in NCBI, which includes the GST classes that contain the typical N-terminal thioredoxin (Trx) domain found in GSTs. This cluster is part of the thioredoxin superfamily, among other well-known clusters such as thioredoxin, glutaredoxin, peroxiredoxin, protein disulfide isomerase (PDI), and disulfide bond A (DsbA) oxidoreductase to name a few. The main criterion used is *a minima* the presence of the two classical GST domains, the N-terminal thioredoxin-like domain with a β1α1β2α2β3β4α3 topology and a C-terminal all-helical domain, that together form a typical GST fold. For this reason, Kappa GSTs and mPGES-1 (microsomal ProstaGlandin E-Synthase type 1), one subclass of MAPEGs (Membrane Associated Proteins in Eicosanoid and Glutathione metabolism) (Bresell et al., 2005), enzymes often integrated into the GST superfamily, are not considered here, even though some terrestrial plants and algae possess at least one mPGES-1 representative. The phylogenetic analysis of all GSTs found in eukaryote photosynthetic organisms has been fitted to the aforementioned criterion, which allows identification of 14 classes (Figure 1). The sequences used were those present in model organisms including a gymnosperm: *Pinus tabulaeformis*, several angiosperms: *Arabidopsis thaliana*, *Populus trichocarpa*, *Oryza sativa*, *Solanum lycopersicum*, and *Hordeum vulgare*, a lycophyte: *Selaginella moellendorffii* and a moss: *Physcomitrella patens*.

Among these 14 classes, Tau, Phi, Zeta, Theta, and tetrachloro-hydroquinone dehalogenase (TCHQD) classes clearly contain GSTs with a catalytic serine. The nature of the catalytic residue in the EF1Bγ and Ure2p classes is less clear, but RIRT and APNG motifs are found at a position similar to the active site signature in other GSTs. Finally, the seven other classes (Iota GSTs (GSTIs), Hemerythrin GSTs (GSTHs), Dehydroascorbate (DHA) reductases (DHARs), GSTLs, GHRs, mPGES-2s, and metaxins) contain members that clearly display a very conserved cysteine in the active site motif, hence suggesting that they belong to Cys-GSTs. Metaxins are part of the mitochondrial translocation system of the mitochondrial outer membrane, being anchored through their C-terminal region whereas the rest of the protein is oriented to the cytosol (Lister et al., 2007). However, they have not been integrated in this study. Indeed, although having the typical GST fold, none of the cysteine of the CPxC signature found in plant sequences is conserved in other organisms notably mammals, and there is no evidence for a cysteine involvement or for a requirement of GSH for their function. Concerning mPGES-2s, they were initially not considered as GSTs because GSH was not absolutely required for the detected activity e.g., the isomerization of ProstaGlandin H_2_ (PGH_2_) (Tanikawa et al., 2002) and because they exhibited a low similarity with GSTs identified at that time. However, based on (i) its typical GST structure, (ii) the identification of another activity strictly requiring GSH, and (iii) the identification of additional more closely related Cys-GSTs (Yamada et al., 2005; Takusagawa, 2013), mPGES-2s do in fact belong to the GST family. It is worth mentioning that Beta GSTs (GSTBs) and GSTOs as well as chloride intracellular channel proteins (CLICs), which belong to Cys-GSTs, are not found in plants. Moreover, particular proteins that are listed as putative GST members under the name “2-GST_N” have not been considered here. Although they have two repeated N-terminal Trx domains and a quite conserved CPFC motif in one of them, they lack the C-terminal domain. Since the GSTI and GSTH isoforms have not yet been characterized, we have essentially focused the following parts on the four remaining Cys-GST classes, describing in detail the current knowledge on DHAR, GHR, GSTL and mPGES-2 enzymes.

### Distribution of cysteinyl GSTs in photosynthetic organisms: gene content, structure, and organization

**Figure 2:**
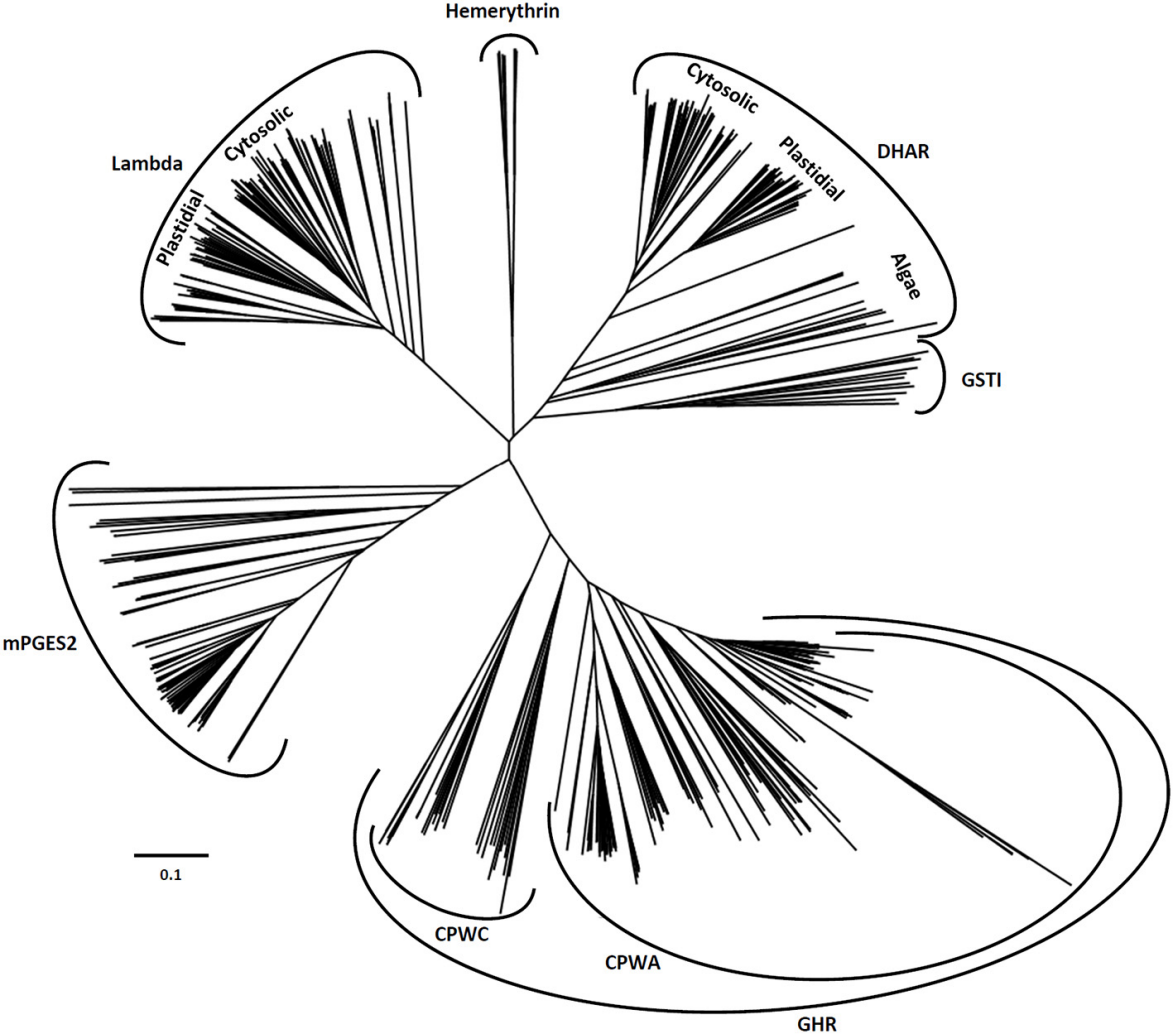
**Unrooted phylogenetic tree of Cys-GSTs present in the green lineage**. Sequences were aligned with PROMALS3D using 1Z9H, 3PPU, and 4PQH PDB structures as templates. Then the alignment has been manually adjusted with Seaview software. The phylogenetic tree was constructed with BioNJ and edited with Figtree software (http://tree.bio.ed.ac.uk/software/figtree/). The robustness of the branches was assessed by the bootstrap method with 500 replications. The scale marker represents 0.1 substitutions per residue. For clarity, the names of the sequences have not been indicated but all sequences are available in the Supplementary Material.

After retrieving all Cys-GST sequences present in representative cyanobacterial and algal genomes as well as in all completely sequenced terrestrial plant genomes, a comparative genomic analysis was performed to get an accurate classification of Cys-GSTs in the green lineage. The resulting phylogenetic tree confirmed six major clades corresponding to the 6 earlier defined classes (Figure 2). In previous phylogenetic analyses conducted with other gene families of the thioredoxin superfamily, e.g., thioredoxins, glutaredoxins, peroxiredoxins and PDIs, the gene structure (number of exons in eukaryotic genes) was conserved and coherent with the classes (Meyer et al., 2002; Rouhier and Jacquot, 2005; Rouhier et al., 2006; Selles et al., 2011). Here, the gene structure was not informative as it was not at all conserved among species, even the phylogenetically close ones.

The DHAR class is essentially present in terrestrial plants. Indeed, this class is absent in cyanobacteria and a single gene, that likely represents the ancestor DHAR gene, is found in a few algae of the chlorophyceae and trebouxiophyceae classes but not in prasinophyceae. The number of DHAR genes in a given species usually lies between 2 and 3 (Table 1). For species possessing a higher number of genes, the increase is not due to tandem duplication as the genes are neither found at adjacent positions nor on the same chromosome in most cases. In our chosen well-annotated reference genomes, there are two genes in *O. sativa* and *S. moellendorffii* and three in *A. thaliana*, *P. trichocarpa*, and *P. patens*. DHARs are split in two well-differentiated subgroups, the genes coding for chloroplastic proteins (CPFC active site motif) and those coding for proteins that are likely all cytosolic (CPFS active site motif) (Figure 2) with algal sequences being dispersed but on the same branch. Since algal genes code for proteins that are devoid of targeting sequences, the ancestral gene might be the one coding for the cytosolic members. Among terrestrial plants, all organisms have at least one member in each clade.

**Table 1:** **Cys-GST gene content in sequenced chlorophytes and embryophytes**.

										**DHAR**	**GHR**	**GSTL**	**mPGES-2**	**GSTI**	**GSTH**	**Total**
**VIRIDIPLANTAE**																
	** Chlorophyte**															
	* Chlamydomonas reinhardtii v5.5*									1	3	0	1	1	0	6
	* Chlorella sp. NC64A*									1	2	0	1	1	0	5
	* Coccomyxa subellipsoidea C-169 v2.0*									2	2	0	1	1	0	6
	* Micromonas pusilla CCMP1545 v3.0*									0	2	0	1	1	0	4
	* Micromonas pusilla RCC299 v3.0*									0	2	0	1	1	0	4
	* Ostreococcus lucimarinus v2.0*									0	1	0	1	1	0	3
	* Volvox carteri v2.0*									1	0	0	1	1	0	3
	** Embryophyte**															
	* Physcomitrella patens v3.0*									3	2	1	2	1	8	17
		** Tracheophyte**														
		* Selaginella moellendorffii v1.0*								2	5	0	1	1	2	11
			** Angiosperm**													
				** *Grass***												
				* Brachypodium distachyon v1.2*						2	2	2	1	0	0	7
				* Oryza sativa v7.0*						2	2	3	1	0	0	8
				* Panicum virgatum v1.1*						3	3	6	1	0	0	13
				* Setaria italica v2.1*						2	2	4	1	0	0	9
				* Sorghum bicolor v2.1*						3	2	4	1	0	0	10
				* Zea mays 6a*						4	7	4	1	0	0	16
				** Eudicot**												
				* Aquilegia coerulea v1.1*						2	2	6	1	0	0	11
					** Pentapetalae**											
					* Mimulus guttatus v2.0*					2	2	3	2	0	0	9
					* Solanum lycopersicum iTAG2.3*					2	2	5	1	0	0	10
					* Solanum tuberosum v3.4*					2	2	3	2	0	0	9
					* Vitis vinifera Genoscope.12X*					2	1	4	2	0	0	9
						** Rosid**										
							** Poplar-Malvidae**									
							* Eucalyptus grandis v1.1*			3	3	8	3	0	0	17
							* Populus trichocarpa v3.0*			3	2	3	3	0	0	11
								** Brassicales-Malvales**								
								* Carica papaya ASGPBv0.4*		2	0	2	1	0	0	5
								* Gossypium raimondii v2.1*		3	2	3	2	0	0	10
								* Theobroma cacao v1.1*		2	3	2	2	0	0	9
									** Brassicaceae**							
									* Arabidopsis lyrata v1.0*	3	4	2	1	0	0	10
									* Arabidopsis thaliana TAIR10*	3	4	3	1	0	0	11
									* Boechera stricta v1.2*	3	4	3	1	0	0	11
									* Brassica rapa FPsc v1.3*	5	4	3	1	0	0	13
									* Capsella grandiflora v1.1*	3	4	2	1	0	0	10
									* Capsella rubella v1.0*	3	2	1	1	0	0	7
									* Eutrema salsugineum v1.0*	3	3	3	1	0	0	10
									** Citrus**							
									* Citrus sinensis v1.1*	2	2	3	2	0	0	9
									* Citrus clementina v1.0*	2	2	3	2	0	0	9
							** Fabidae**									
							* Linum usitatissimum v1.0*			5	4	4	2	0	0	15
							* Manihot esculenta v4.1*			2	1	2	2	0	0	7
							* Ricinus communis v0.1*			3	2	3	1	0	0	9
								** Nitrogen-fixing**								
								* Cucumis sativus v1.0*		2	2	3	2	0	0	9
								* Fragaria vesca v1.1*		2	2	3	2	0	0	9
								* Glycine max Wm82.a2.v1*		4	2	5	3	0	0	14
								* Malus domestica v1.0*		8	5	6	4	0	0	23
								* Medicago truncatula Mt4.0v1*		2	1	4	2	0	0	9
								* Phaseolus vulgaris v1.0*		2	2	4	2	0	0	10
								* Prunus persica v1.0*		2	2	2	2	0	0	8

Sequences have been retrieved from Phytozome 10 and Joint Genome Institute databases. They are provided as Supplementary Material.

GSTLs appear unique to terrestrial plants, the number of genes ranging generally from 2 to 4 with the exception of *S. moellendorffii*, where the gene seems absent (Table 1). In *A. thaliana*, two genes (*AtGSTL1* and *AtGSTL2*) are repeated in tandem on the chromosome 5, likely indicating a recent duplication event. On the other hand, specific expansions have arisen in some species such as *Aquilegia coerulea*, *Malus domestica*, *Eucalyptus grandis*, and *Panicum virgatum* which have 5–8 genes. In this case, some events of tandem duplication have largely contributed to this increase. This is particularly true in *Eucalyptus grandis*, a species in which there are two gene clusters, one having a series of four genes in a row. In the phylogenetic tree, the genes coding for the chloroplastic and cytosolic isoforms clearly separate into two groups. Since the single *GSTL* gene found in *P. patens* encodes a chloroplastic protein, the ancestral version in the green lineage should be the chloroplastic-encoding gene. On the other hand, the absence of *GSTL* genes in cyanobacteria and algae raises the question of the appearance of these isoforms. In a few cyanobacteria and algae, there are orphan, non-annotated sequences sharing similar active site motifs (CPYA). For this reason, it is tempting to speculate that these sequences might correspond to the ancestral gene and that it has been lost in most organisms. The fact that the overall similarity of these orphan sequences with GSTLs is low and that they do not necessarily form a single clade with GSTLs could come from their rapid and independent evolution. This will have to be further explored when additional genomes and sequences will be available.

The GHR class is widespread, with at least one gene present in almost all analyzed photosynthetic organisms and in most species. There are between 2 and 4 GHR genes (Table 1). The absence of gene in some species might be due to either annotation problems or gene loss. Furthermore, the gene family expansion found in some species, e.g. *Zea mays*, *S. moellendorffii*, and *M. domestica* cannot be explained by tandem duplication in this case. For *M. domestica*, which has by far the highest number of Cys-GST genes (23 genes) and exhibits gene expansion in all classes, this can be explained by a recent genome-wide duplication (Velasco et al., 2010). The widespread nature of GHRs is also true outside photosynthetic organisms since they are present almost everywhere including archaea of the halobacteriaceae order, but excluding mammals (Table 2). Overall, this suggests that GHRs have crucial functions, or at least functions that cannot be ensured by other GSTs.

**Table 2:** **Characteristics and distribution of Cys-GSTs**.

**Class**	**Origin**	**Typical catalytic motif**	**Average amino acid length**	**Oligomerization state**
GSTB	Bacteria	GA_12_CS	210	Dimer
GSTO	Mammals, insects, fungi	_35_CPFA	250	Dimer
CLIC	Animals	_35_CPFS	250	Monomer Dimer Oligomer
GSTL	Terrestrial plants	_40_CPF/YA	230	Monomer
DHAR	Algae, terrestrial plants	_20_CPFC/S	220	Monomer
GHR	Some metazoan but animals, algae, terrestrial plants, fungi, cyanobacteria, bacteria, archaea	_50_CPWA	330	Dimer
mPGES-2	Animals, protists, algae, terrestrial plants	_110_CPFC	310	Dimer
GSTH	Bryophyta, lycophyta	_50_CPF/YT	510	?
GSTI	Algae, bryophyta, lycophyta	_120_CPYC	490	?

The case of CLIC proteins is particular since it exists under both a monomeric soluble and an oligomeric transmembrane form. Moreover, the formation of an intramolecular disulfide bond promotes a structural transition that exposes a large hydrophobic surface changing the monomer into a non-covalent dimer (Littler et al., 2004).

Regarding the mPGES-2 class, its genes are absent in cyanobacteria whereas at least one gene is present in algae and terrestrial plants. This suggests that mPGES-2 proteins may have a widespread and essential function. Since most organisms retained only one gene, the duplication observed in some specific organisms probably derives from isolated events. Additionally, these GSTs are also largely distributed among kingdoms since they are found in mammals, nematodes, insects, and trypanosomatids but not fungi (Table 2).

The last two classes, GSTI and GSTH, are restricted to specific organisms. The GSTIs are found as a single gene in some cyanobacteria, algae, and in non-vascular plants (*S. moellendorffii*, and *P. patens*). In the phylogenetic tree, they form a single clade that is close to DHAR, possibly indicating that DHARs derive from GSTIs. The fact that GSTIs have been lost at some steps in the green lineage evolution and are no longer present in most terrestrial plants may also indicate that the associated function(s) disappeared or that other GSTs fulfill similar roles. The distribution of GSTHs is even more puzzling as from current available genomes, they are only found in *S. moellendorffii* and *P. patens*. The presence of 8 genes in *P. patens* is particularly striking, taking into account that, from the analysis of EST sequences, there is evidence for the expression of six genes. In *P. patens*, all these genes form a single gene cluster that likely originates from several duplication events.

### Sequence characteristics and domain organization of cysteinyl GSTs

From the phylogenetic tree and the amino acid sequence alignments, there are key sequence differences that allow differentiation of each class. In addition to describing conserved motifs typical of each class, we have paid attention to the presence of N- and C-terminal extensions or of sequence insertions. Some differences are also reflected at the structural level since DHARs and GSTLs are monomeric enzymes, whereas GHRs and mPGES-2s are dimeric enzymes (Table 2). Thus, the residues forming the dimeric interface should also constitute a good criterion for distinguishing monomeric from dimeric proteins. This will be discussed further when relevant, either in this section or in the section dealing with the structural characteristics. It is worth noting that N-terminal extensions corresponding to predicted targeting sequences have been excluded when describing the size of the proteins and the percentage identity among isoforms.

**Figure 3:**
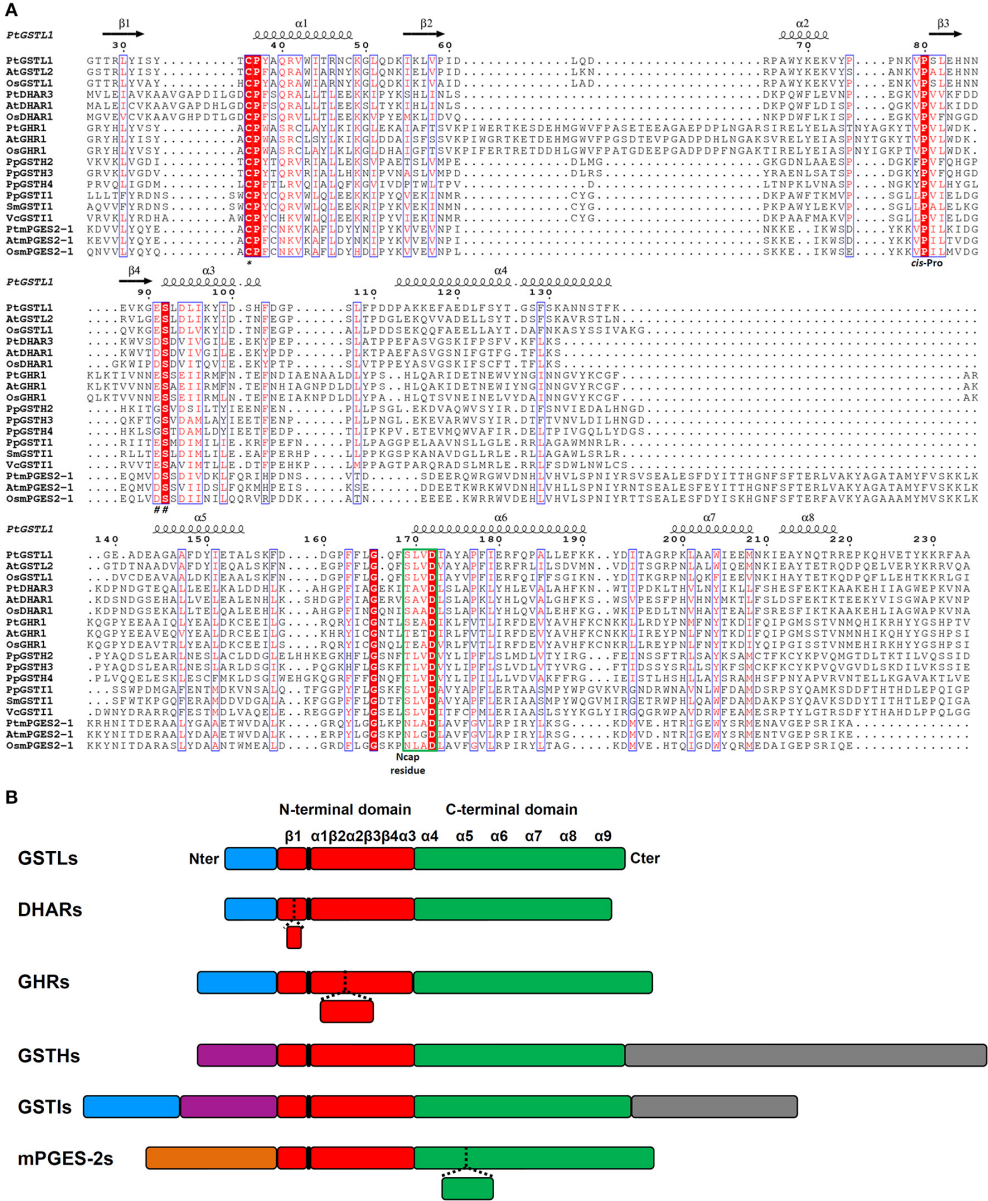
**Amino acid alignment and protein architecture of plant Cys-GSTs. (A)** Amino acid sequence alignment of three representative members from each Cys-GST class. The sequences were structurally aligned using PROMALS3D server using as references the solved structures of PtGSTL1 [PDB code 4PQH (Lallement et al., 2014)], PtGSTL3 [PDB code 4PQI (Lallement et al., 2014)], *Phanerochaete chrysosporium* GHR1 [PDB code 3PPU (Meux et al., 2011)], and *Macaca fascicularis* mPGES-2 [PDB code 1Z9H (Yamada et al., 2005)] since there is no structure available for DHARs, GSTIs, and GSTHs. Since the structure of poplar GSTL1 has been solved, its secondary structures have been indicated as reference using ESPript 3.0 (http://espript.ibcp.fr/ESPript/ESPript/index.php), with the helices and the arrows corresponding respectively to α-helices and to β-strands. Strictly conserved residues are marked in white characters on a red background, whereas residues with similar functional groups are in red characters on white background. The indicated numbering corresponds to that of PtGSTL1 which has been used as a whole. For clarity, N- and C-terminal extensions present in Cys-GSTs have been removed from the alignment to keep only the sequences corresponding to secondary structures forming the GST fold. At is for *Arabidopsis thaliana*, Pt for *Populus trichocarpa*, Os for *Oryza sativa*, Pp for *Physcomitrella patens*, Sm for *Selaginella moellendorffii*, and Vc for *Volvox carteri*. The catalytic cysteine (^*^), *cis*-proline (*cis*-Pro), residues stabilizing the γ-glutamate residue of glutathione (##) and N-cap residue are shown. The N-capping box is surrounded in green. **(B)** Schematic representation of the protein architecture of plant Cys-GSTs. The N-terminal Trx-like domain and the all-helical C-terminal domain are represented respectively in red and green. Blue boxes correspond to putative or confirmed targeting sequences. The orange box corresponds to the membrane anchoring tail of mPGES-2. Purple boxes represent N-terminal extensions that do not correspond to targeting sequences and gray boxes represent additional C-terminal domains. The position of the active site motif harboring the catalytic cysteine is indicated in black. The presence of inserted sequences in some classes corresponds to dashed lines in other classes. Secondary structures are shown as α-helices and β-strands. The size of the boxes is proportional to the length in amino acids.

From previous phylogenetic analyses conducted with Trx superfamily members, important features to consider have been defined. First, the nature and the position of the active site motif is an essential element. It should be recurrently situated at the beginning of the first α-helix of the thioredoxin fold, which does not mean that extra α-helices cannot be found before. For this reason, the position of the catalytic cysteine can vary although it is generally found around position 20 to 50 (Table 2). The second major characteristic used for comparison is the nature of the residue found before a very conserved *cis*-proline that is typical of proteins of the Trx superfamily. This *cis*-proline is generally positioned 30 to 40 amino acids after the active site signature. According to this feature, an alignment of three representative sequences from each class allowed determining the residues that are conserved among Cys-GSTs (Figure 3A). The numbering used is the one corresponding to poplar GSTL1. There are six residues that are mostly conserved in all Cys-GST classes, or at least in the sequences used for the alignment. As expected, the catalytic Cys, found at position 36, and the *cis*-Pro discussed above at position 80, are conserved. Interestingly the Pro37 adjacent to the catalytic Cys is also found in the vast majority of sequences. Hence, the differences between GST classes will be essentially visible by checking the two other positions of the CPxx active site motif. Besides, three other residues are also commonly found in all GSTs. As expected for enzymes that share the same co-substrate, some of the residues contributing to the recognition of GSH, Ser92 and Asp172, are conserved. The last conserved residue is a Gly at position 166 for which function is unknown.

With these features in mind, the difference between classes has been simply analyzed by looking at some specific criteria: (i) the percentage identity, (ii) the size of the proteins, (iii) the presence of extra domains, and (iv) the presence of three specific signatures, i.e. the active site sequence motif and the residues immediately before the *cis*-Pro and before the serine involved in GSH binding. Concerning the first criterion, the percentage identity between members of a given class is usually above 50%, whereas it is usually below 20% between classes.

Protein sizes vary slightly within classes, but they vary more significantly between classes. DHARs and GSTLs are the shorter Cys-GSTs, since they have about the same size ranging from 210 to 220 amino acids for the former and from 230 and 240 amino acids for the latter (Table 2). Nevertheless, compared to all other Cys-GSTs, DHARs have a nine amino acid insertion before the α1 helix and thus the active site motif (Figure 3). GHRs and mPGES-2s also have approximately the same size, *ca* 330 residues, but the sequence insertions explaining the difference with DHARs or GSTLs are not found at the same position. In the case of mPGES-2, the difference comes from the presence of an N-terminal membrane-anchoring region and of an insertion of about 40 residues between the α4 and α5 helices (Figure 3B). This insertion is different from the 20 amino acid insertion found between α3 and α4 helices in vertebrate isoforms which corresponds to two α-helices and two β-sheets (this is further discussed in Section 3D Structures). For GHRs, the size difference is essentially linked to insertions in the Trx domain (*ca* 35 amino acids between the active site motif and the *cis*-Pro, i.e. between the β2 strand and α2 helix) and to a final extension of 20 to 25 amino acids. The latter contains most of the residues responsible for the atypical GHR dimerization (see Section 3D Structures). Finally, GSTIs and GSTHs contain about 500 residues. GSTIs are slightly extended in the N-terminal part, but it is not yet clear whether this is a targeting sequence. Most of the additional sequence (around 120–140 residues) is present at the C-terminus and could correspond to a phycoerythrin α-subunit domain found in phycobilisome proteins (Figure 3B). GSTHs are also extended at the C-terminal end but this is due to the presence of an hemerythrin domain of *ca* 150–200 residues as its name suggests, followed by about 100 additional amino acids with no domain annotation (Figure 3B).

In the next part, we focused on the conservation of the sequence signatures mentioned above. If we consider the CPYC or CPFC active sites found in glutaredoxins as a reference, all plant Cys-GSTs display a reminiscent catalytic motif that differs by only one residue, with the exception of most GHRs which have two variations in their CPWA motif (Table 2). In these proteins, the catalytic cysteine is usually found at position 50. It is interesting to note that most algae have two GHR members including one isoform with an atypical CPWC motif (Figure 2). Except two algal sequences having a CPYC active site, mPGES-2s have usually a very conserved CPFC motif found around the position 110 owing to the presence of the N-terminal membrane-anchoring region. For DHARs, except a few sequences where the catalytic cysteine seems to be replaced by a glycine, the active site motif, found around the position 20, is usually quite conserved being of the CPFC or CPFS form. Among GSTLs, the active site motif is found around position 40 and it is mostly of the CP[F/Y]A form. The similarity with GSTOs (active site sequence and size of the proteins) might suggest a common origin. This is further supported by the fact that organisms having GSTOs do not have GSTLs and *vice versa*. Another extremely interesting observation is that GSTLs with SPFA motifs can be found in a few analyzed species as *E. grandis*, *Linum usitatissinum*, *M. domestica*, and *Ricinus communis*. This is also true for some fungal GSTOs found in particular in *Phanerochaete chrysosporium* or *carnosa* and *Trametes versicolor* where the classical CPY/FA motif is replaced by a SPY[C/S] motif (Morel et al., 2009). Although it should confer opposite properties (glutathionylating vs. deglutathionylating activities) to the proteins, this suggests that GST genes can be maintained in genomes as long as the replaced amino acid conserves catalytic functions. Concerning GSTIs and GSTHs, the fact that these sequences are restricted to a few species and that the number of sequences available is low makes the analysis of amino acid conservation less robust. Nevertheless, it appears that most GSTI sequences exhibit a conserved WCPYC motif, except one representative from *C. reinhardtii* that has a RCPYC sequence. If it turns out that the N-terminal extension is indeed a targeting sequence, this motif is located around the position 60, otherwise it is located around the position 120. In GSTHs, the active sequence is CP[F/Y]T and depending on the isoform considered, it is usually found around position 50 or 70.

Finally, the residues associated to the *cis*-Pro80 and to Ser92 may help to definitely discriminate GST classes. The classes that cannot be differentiated using these signatures are GSTLs and GHRs which usually exhibit VP and ES motives, and DHARs and mPGES-2 which have VP and DS motives. However, as explained above, the other factors will allow distinguishing them. The last two classes, GSTIs and GSTHs, have specific recognizable sequences, LP and ES or [F/Y]P and GS, respectively.

### Gene expression

To date, there is not much data available on plant Cys-GSTs, both at genetic and physiological levels. Nonetheless, the physiological roles of these enzymes can certainly be better understood by delineating the gene expression in plant organs or in response to environmental constraints. Since only partial information is available for each class, analyzing microarray experiments represents a valuable approach ahead of targeted expression studies. Hereto, *A. thaliana* microarray experiments were analyzed to assess both the developmental expression patterns of each GST using the EFP browser (Winter et al., 2007) and the transcriptional regulation occurring in response to environmental stimuli using Genevestigator (3072 perturbations were analyzed) (Hruz et al., 2008). Among the 14 genes coding for Cys-GSTs in Arabidopsis, four groups can be distinguished based on the absolute levels of expression, ranging from high to low: (i) DHAR1, DHAR3 and GSTL3, (ii) GSTL2, DHAR2 and GHR1, (iii) mPGES-2, GHR2 and GHR4, and (iv) GSTL1 and GHR3.

**Figure 4:**
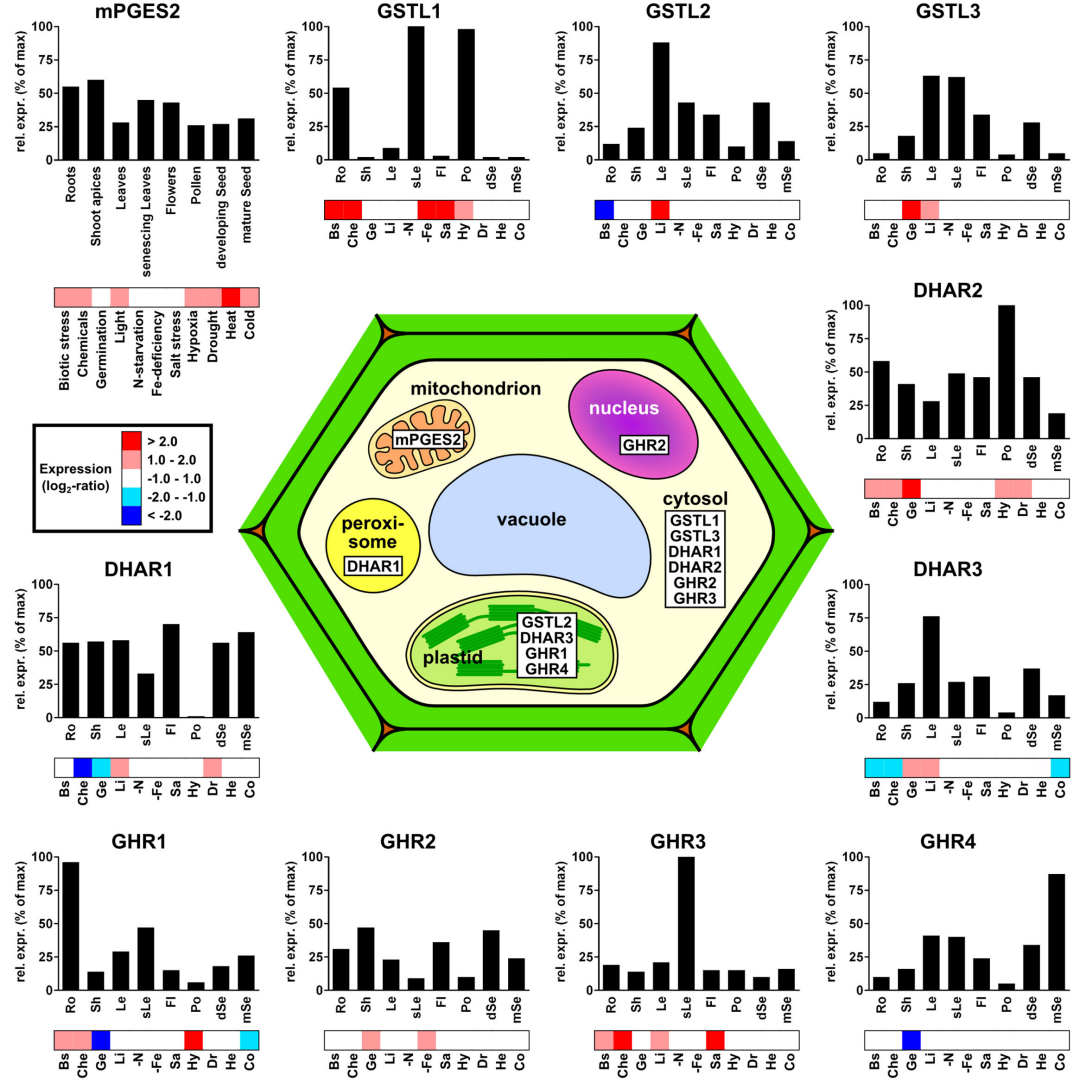
**Subcellular localization and gene expression profiles of the different *Arabidopsis thaliana* Cys-GSTs**. For each GST, the relative transcript expression is shown in relation to both plant developmental stages (bar-graphs) and perturbations (heatmaps). For clarity, the number of developmental conditions was reduced to eight classes, where each gene was normalized to its maximum expression within the selected dataset (see Materials and Methods). For the response to perturbations, the expression values were organized into five color-coded groups based on their log_2_-ratios. For clarity, the numerous perturbations included in the dataset were grouped into 11 classes: Biotic stress (Bs), Chemicals (Che), Germination (Ge), Light (Li), N-starvation (-N), Fe-deficiency (-Fe), Salt stress (Sa), Hypoxia (Hy), Drought (Dr), Heat (He), and Cold (Co).

The expression of mPGES-2 genes has never been studied in plants. In mammals, however, the genes and corresponding enzymes have been shown to be constitutively expressed and involved in prostaglandin E metabolism, respectively (Murakami et al., 2003). In Arabidopsis, *mPGES-2* transcripts are found in all analyzed organs and at quite constant level (Figure 4). Based on the data available in Genevestigator, *A. thaliana mPGES-2* is the most significantly regulated gene in response to environmental stresses among Cys-GSTs. It is overexpressed in response to cold, drought, hypoxia, increases in light, chemical and biotic stresses, and shows its strongest up-regulation under heat stress. Altogether, this suggests that this gene may be involved in general tolerance mechanisms to biotic and abiotic stresses.

Regarding *GSTL* genes, *AtGSTL2* and *AtGSTL3* show similar expression patterns, particularly in green tissues, such as leaves, flower sepals, siliques, and developing seeds, whereas *AtGSTL1* transcripts are almost exclusively found in roots, senescing leaves, and pollen (Figure 4). In terms of stress responses, *AtGSTL1* is strongly up-regulated in several conditions including biotic interactions, treatment with chemicals, salt and iron-starvation stresses and, to a lesser extent, in response to hypoxia. This is consistent with a study showing that the *AtGSTL1* gene is induced in root cell cultures in response to buthionine sulfoximine (BSO), tert-butyl hydroperoxide, dichlormid, and 2,4 dichlorophenoxy acetic acid (Dixon et al., 2002). This also corroborates the observation that expression of tomato *GSTL3* is induced by salt treatments in both roots and leaves (Csiszar et al., 2014). Interestingly, in contrast to *AtGSTL1*, *AtGSTL2*, and *AtGSTL3* are less responsive to environmental stress factors, but respond specifically to conditions involving increases in light, such as germination and light-shifts. These differences in expression patterns may help identifying the function of the three *AtGSTL* genes. Additionally, a number of studies has explored the expression and tissue distribution of *GSTLs* in other plant species. For instance, the three rice *GSTL* genes are all differentially expressed in response to arsenic treatments (Kumar et al., 2013a). Moreover, *OsGSTL1* and *OsGSTL2* are both constitutively expressed and involved in xenobiotic and oxidative stress tolerance in rice, whereas *OsGSTL2* is also specifically up-regulated in roots after herbicide (chlorsulfuron and glyphosate) and hormone treatments (salicylic acid and naphthalene acetic acid) (Hu et al., 2009, 2011a,b). Consistently, Arabidopsis transgenic lines expressing *OsGSTL2* are more tolerant to abiotic stresses such as heavy metals, cold, drought, and salt stress (Kumar et al., 2013a,b). Recently, the expression of the three poplar GSTL genes was studied in a naturally growing *Populus trichocarpa* adult tree (Lallement et al., 2014). One of these genes, *PtGSTL3*, generates two transcripts by alternative splicing, *PtGSTL3A* and *PtGSTL3B*, the latter being very weakly expressed. While *PtGSTL2* and *PtGSTL3A* seem to be constitutively expressed, all PtGSTL genes are preferentially expressed in the reproductive organs (flowers, fruits, buds) (Lallement et al., 2014). Nevertheless, poplar *GSTLs* have also been detected in leaves and roots (Lan et al., 2009). Altogether these results suggest that *GSTLs* are mainly expressed in organs that have a more intense secondary metabolism, which is consistent with the proposal that GSTLs are involved in the biosynthesis and/or maintenance of the flavonoid pool (see the Section Enzymatic Properties and Physiological Roles).

The three *A. thaliana DHAR* genes are expressed in most organs tested. Although *AtDHAR1* and *AtDHAR3* are either weakly or not expressed in pollen, this may be compensated by *AtDHAR2* which has its highest expression in this organ (Figure 4). Aside from this, the only notable difference is that *AtDHAR3* is relatively strongly expressed in leaves, which is consistent with its predicted localization to plastids (Figure 4). In other organisms where *DHAR* expression was studied, the genes were shown to be expressed in most tissues/organs. This is the case for the three poplar *DHARs* in roots, shoots, leaves, phloem, and buds (Lan et al., 2009; Tang and Yang, 2013) and for one *DHAR* from *Pinus bungeana* in buds, needles, phloem from stems, roots, and seedlings (Yang et al., 2009). However, in the moss *P. patens*, one of the three genes does not seem to be expressed at all (Liu et al., 2013). In response to environmental variations, the microarray data of *A. thaliana* indicates that *AtDHAR2* is the most responsive gene being up-regulated during germination and in response to biotic stress, chemicals, hypoxia, and drought. In contrast, *AtDHAR1* and *AtDHAR3* are up-regulated in only two conditions, excess light or drought and excess light or germination, respectively. Surprisingly, both genes are down-regulated in response to chemicals, a condition where GSTs are usually over-expressed. In plants, several independent studies have been performed with the aim of addressing the role of *DHARs* during stress response. For instance, *AtDHAR1* is up-regulated in response to norflurazon, menadione, paraquat, and antimycin A, treatments known to produce reactive oxygen species (Chew et al., 2003). On the other hand, *AtDHAR2* expression is induced in root cell cultures in response to BSO and chloro-dinitrobenzene (CDNB) (Dixon et al., 2002). However, in other organisms, *DHARs* are not always regulated in the same manner in response to stress conditions. For example, *DHAR2* from *P. patens* is strongly down-regulated by the addition of H_2_O_2_, salt, salicylic acid, and atrazine (Liu et al., 2013), whereas poplar *DHAR2*, in contrast to poplar *DHAR3*, is up-regulated in shoots but not in roots in response to H_2_O_2_, atrazine and to a lesser extent CDNB (Lan et al., 2009). Altogether these data point to a crucial function of DHARs and consequently ascorbic acid for stress responses, although the pattern of expression and regulation of DHAR genes differ from one organism to another.

Since GHRs were only recently identified in plants, they have not been studied in detail and very little is known about their expression and regulation. In yeast, GHR genes were formerly referred to as GSTO1, GSTO2, and GSTO3. The *GSTO1* gene, which encodes a peroxisomal protein involved in sulfur metabolism, was shown to be induced by oxidative stress conditions (Barreto et al., 2006). Based on the analysis of *A. thaliana* microarrays, it is clear that GHRs are among the Cys-GSTs that are the least expressed, even though they are expressed in all plant organs analyzed. Apart from *GHR2*, which shows no preferential expression, the genes are each predominantly expressed in a particular organ, roots for *GHR1*, senescent leaves for *GHR3* and mature seeds for *GHR4* (Figure 4). In response to stress conditions, *A. thaliana* GHR genes are differentially regulated as well. *AtGHR4* appears only to be down-regulated during germination. While *AtGHR1* is down-regulated during germination and in response to cold, it is up-regulated in response to chemicals and biotic stress, together with *GHR3*. However, the expression of *AtGHR1* is also increased by hypoxia, while *AtGHR3* is increased in response to salt treatments and increases in light. Finally, in contrast to *AtGHR1* and *AtGHR4*, *AtGHR2* is up-regulated during germination and also shows up-regulation in response to iron starvation.

### Subcellular localization

Deciphering the subcellular localization of all these proteins should also contribute to the understanding of their biological role. The data present in the literature for *A. thaliana* and poplar Cys-GSTs, originating from proteomic studies or from GFP fusion experiments have been compiled together with bioinformatic predictions for the presence of targeting sequences and summarized in Figure 4 and Table 3. First, while not much is known about plant mPGES-2s, mammalian mPGES-2s exhibit a dual subcellular localization associated to both the Golgi membrane through their N-terminal part and the cytoplasm after proteolytic cleavage of the N-terminal hydrophobic domain (Tanikawa et al., 2002; Murakami et al., 2003). Similarly, plant mPGES-2 proteins possess an N-terminal extension, but it is predicted to correspond to a mitochondrial or plastid targeting sequence. This might be supported by the identification of this protein in two proteomic studies of mitochondrial protein fractions (Table 3) (Heazlewood et al., 2004; Klodmann et al., 2011). However, a careful inspection of the nature of the amino acids present in this region is rather consistent with a membrane-anchoring tail. Accordingly, the Arabidopsis ortholog was also identified in a proteomic study of plasma membrane proteins and an N-terminal transmembrane domain is indeed predicted by some prediction programs devoted to their identification (Table 3) (Mitra et al., 2009). Altogether, these data will have to be firmly established by complementary experiments, especially if a cleavage could also generate soluble isoforms. The mPGES-2s could be the only membrane associated Cys-GSTs since no other protein was predicted to possess a membrane-anchoring region.

**Table 3:** **Subcellular localization of *Arabidopsis thaliana* Cys-GST members and of poplar orthologs**.

**Gene name**	**Accession number**	**Predicted subcellular localization**	**Confirmed localization and other proteomic evidence**	**Amino acid length**	**Orthologs in poplar**	**References**
DHAR1	At1g19570	Elsewhere	*- FP fusions:* cytosol^a^, peroxisome^b^ *- High-throughput proteomic:* mitochondria^c^, cytosol^d^, plasma membrane^e^, chloroplast^f^	213	*- FP fusions:* DHAR2: cytosol^b^, DHAR3: cytosol^b^	^a^Grefen et al., 2010 ^b^Reumann et al., 2009 ^c^Chew et al., 2003 ^d^Ito et al., 2011 ^e^Marmagne et al., 2007 ^f^Peltier et al., 2006
DHAR2	At1g75270	Elsewhere	*- High-throughput proteomic*: cytosol^a^, plasma membrane^b^	213	*- FP fusions:* DHAR2: cytosol^b^, DHAR3: cytosol^b^	^a^Ito et al., 2011 ^b^Marmagne et al., 2007 ^c^Tang and Yang, 2013
DHAR3	At5g16710	Chloroplast	*- High-throughput proteomic*: chloroplast^a^	258	*- FP fusions:* DHAR1: chloroplast^b^	^a^Zybailov et al., 2008 ^b^Tang and Yang, 2013
GSTL1	At5g02780	Elsewhere	None	237	*- FP fusions:* GSTL2, GSTL3A & B: nucleocytoplasmic^a^	^a^Lallement et al., 2014
GSTL2	At3g55040	Chloroplast	*- FP fusions*: peroxisome^a^, *- High-throughput proteomic*: chloroplast^b-d^	292	*- FP fusions:* GSTL1: chloroplast^e^	^a^Dixon et al., 2009 ^b^Zybailov et al., 2008 ^c^Peltier et al., 2006 ^d^Ferro et al., 2010 ^e^Lallement et al., 2014
GSTL3	At5g02790	Elsewhere	*- High-throughput proteomic*: cytosol^a^	235	*- FP fusions:* GSTL2, GSTL3A & B: nucleocytoplasmic^b^	^a^Ito et al., 2011 ^b^Lallement et al., 2014
GHR1	At4g19880	Chloroplast	*- High-throughput proteomic*: cytosol^a^, chloroplast^b^	356		^a^Ito et al., 2011 ^b^Klodmann et al., 2011
GHR2	At5g45020	Elsewhere	None	325		
GHR3	At5g44990	Elsewhere	None	350		
GHR4	At5g44000	Chloroplast	*- High-throughput proteomic*: chloroplast^a^	399		^a^Ferro et al., 2010
mPGES-2	At5g42150	Mitochondria or chloroplast	*- High-throughput proteomic*: mitochondria^a,b^, plasma membrane^c^	315		^a^Heazlewood et al., 2004 ^b^Klodmann et al., 2011 ^c^Mitra et al., 2009

The prediction of subcellular localization was performed by compiling results obtained from various softwares as Predotar, TargetP, and Wolfpsort. Experimental confirmation consisting of high-throughput proteome analyses and fusions with fluorescent proteins (FP fusions) and associated references are indicated when available.

Among DHARs, based on its occurrence in chloroplast proteome analyses and the presence of an N-terminal extension in the protein sequence, *A. thaliana* DHAR3 should be chloroplastic (Table 3). Poplar and *P. patens* orthologs have a similar localization (Liu et al., 2013; Tang and Yang, 2013). The two other proteins from Arabidopsis are predicted to be cytosolic as they do not exhibit visible targeting sequences. However, proteome analyses and YFP fusion proteins indicate that AtDHAR1 is also present in peroxisomes (Reumann et al., 2009; Grefen et al., 2010).

Concerning GSTLs, based on proteome analyses and on the presence of an N-terminal predicted targeting sequence, AtGSTL2 is clearly a plastidial protein and it may also be present in peroxisomes as shown by GFP fusion experiments as well as in the cytosol (Dixon et al., 2009). The poplar ortholog, PtGSTL1, is also present in plastids but *a priori* not in peroxisomes (Lallement et al., 2014) and the *P. patens* isoform is also plastidial (Liu et al., 2013). The two other GSTLs found in *A. thaliana* and *P. trichocarpa* should be cytosolic proteins, although a nuclear localization was observed when poplar proteins were fused to GFP (Lallement et al., 2014). Considering the absence of a clear NLS (nuclear localization signal), this nuclear localization is more likely due to a passive diffusion through nuclear pore rather than to a specific targeting.

Concerning GHRs, there is no information available yet. Among the four isoforms found in *A. thaliana*, two of them are predicted to be chloroplastic proteins and have additionally been indeed identified from high-throughput proteomic analyses (Table 3). Concerning Hemerythrin GSTs, the proteins do not exhibit clear targeting sequences suggesting cytosolic localization. Accordingly, four GSTHs from *P. patens*, PpGSTH1, 2, 3, and 7, presented a nucleo-cytoplasmic localization in GFP fusion experiments (Liu et al., 2013). Concerning GSTIs, according to the existence of clearly visible N-terminal extensions in some representative members, several prediction programs indicate that they could be targeted either to the chloroplasts or to mitochondria, although this remains to be demonstrated experimentally.

### 3D structures

At the structural level, GSTs consist of an N-terminal domain adopting a thioredoxin fold and an all-helical C-terminal domain (Atkinson and Babbitt, 2009). The GSH binding site, or G site, is located in a cleft formed between the two domains and most of the residues contacting GSH are provided by the N-terminal domain. The binding site for the hydrophobic electrophiles, or H site, is located immediately adjacent to the G site and forms part of the solvent-exposed cleft between both domains. For the H site, most of the residues contacting the electrophiles are provided by the C-terminal domain. Both sites form the protein active site. Moreover, non-catalytic ligandin sites (L site) were defined in GSTs. Two types of L site may be roughly distinguished: those overlapping partially with the H site and those located at the dimer interface straddling the two fold axis (Litwack et al., 1971; McTigue et al., 1995; Rossjohn et al., 1998; Smith et al., 2003; Axarli et al., 2004; Dixon et al., 2008; Brock et al., 2013).

**Figure 5:**
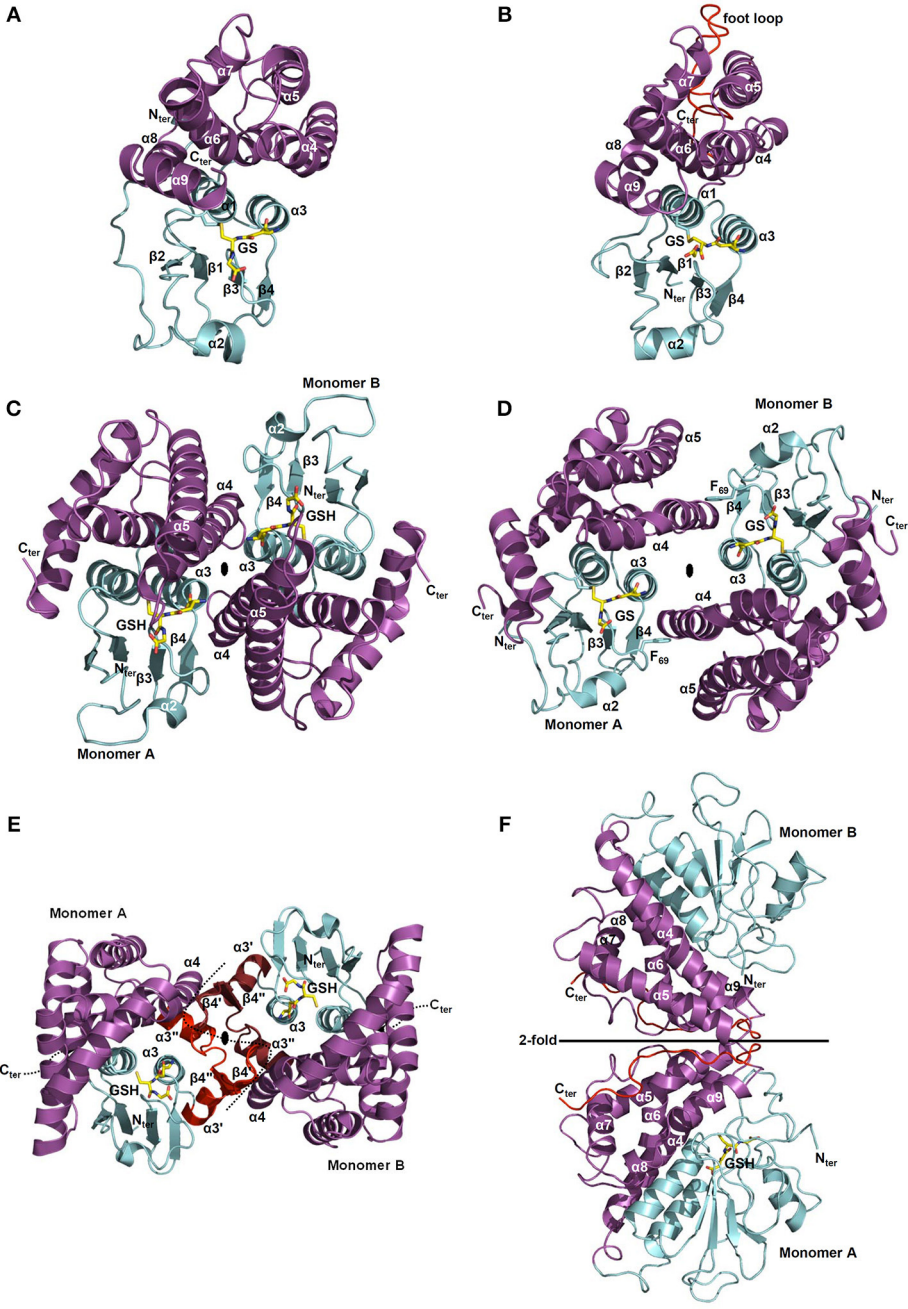
**Structural organization of Cys-GSTs**. All structures are shown as cartoon with the N- and C-terminal domains colored in cyan and in purple, respectively. Glutathione (GSH) or glutathione adducts (GS) are represented as sticks. In **F**, glutathione is only present in monomer A. All figures have been prepared with Pymol software. **(A,B)** Monomeric organization of **(A)** GSTL3 from *Populus trichocarpa* (PDB code 4PQI) and **(B)** CLIC1 from *Homo sapiens* (PDB code 1K0M). These monomeric enzymes illustrate the classical GST fold which consists of an N-terminal domain adopting a thioredoxin fold (β1α1β2α2β3β4α3) and an all helical C-terminal domain. Human CLIC1 **(B)** harbors a long negatively charged loop also referred as “foot loop” (colored in red) inserted between helices 5 and 6. This loop is characteristic of CLICs and might be responsible for interaction with other proteins. The glutathione adduct (GS) has been modeled based on the superimposition with a glutathionylated version of *Homo sapiens* CLIC1 (PDB code 1K0N). **(C,D)** Classical dimerization mode of GSTs as shown using **(C)** *Ochrobactrum anthropi* GSTB (PDB code 2NTO) and **(D)** *Homo sapiens* GSTO1-1 (PDB code 1EEM). The monomers associate along a structural C2 axis. The N-terminal domain (loop α2-β3, strand β4 and helix α3) of one subunit interacts with the C-terminal domain (helices α4 and α5) of the other monomer. The dimer interface is either hydrophilic **(C)** or hydrophobic **(D)**. The hydrophobic interaction is characterized by the insertion of a phenylalanine (or a tyrosine) residue belonging to the α2-β3 loop into a hydrophobic pocket located between helices α4 and α5 of the C-terminal domain of the other subunit (“lock-and-key” motif). **(E)** *Macaca fascicularis* mPGES-2 (PDB code 2PBJ). The dimerization occurs via a α3′β4′β4″α3″ structure (colored in red) inserted between α3 and α4 that interacts with those of the other monomer (colored in ruby). Note that this insertion is not found in plant sequences. **(F)** *Phanerochaete chrysosporium* GHR1 (3PPU). The two monomers interact via their C-terminal domain (in red) and are related to each other by a 2-fold symmetry axis.

The N-terminal thioredoxin domain is often described as two distinct motifs: an N-terminal motif (β1α1β2) and a C-terminal motif (β3β4α3) linked by helix α2 and which together form a four β-sheet in the order 2134 with β3 anti-parallel to the others (Figure 5A). Despite the low primary sequence conservation between GST classes, the position of the key residues is maintained. As mentioned above, the cysteine or serine of the catalytic signature is located at the beginning of α1 helix which also contains charged residues involved in the proton transfer reaction. For GSTs having a catalytic tyrosine residue, it is positioned at the end of the β1 strand. The invariant *cis*-Pro residue is located in the loop region before β3 and is thought to be implicated in the maintenance of the enzyme fold rather than playing a role in the enzymatic reaction (Figure 3A) (Allocati et al., 1999). The residues responsible for the non-covalent anchoring of GSH are well-conserved in most known GSTs. Glutamate/aspartate/glutamine residues and the adjacent serine residue in the loop β4-α3, stabilize the charged group of GluGS (γ-glutamate residue of glutathione). The main chain of a valine/leucine/isoleucine/threonine residue that precedes the conserved *cis*-proline is hydrogen-bonded to the backbone of CysGS (cysteine residue of glutathione) (Figure 3A). In the loop β2-α2, a charged residue (lysine, arginine), not present in all GSTs, makes a salt bridge with the carboxyl group of GlyGS (glycine residue of glutathione). In addition to these usual interactions with GSH, other less conserved residues can also contribute to the stabilization of GSH in specific cases.

The C-terminal domain exhibits a bundle of helices whose number varies between each class. This less conserved domain, compared to the N-terminal domain, notably contains a well-conserved N-capping box (S/TXXD) including the S/T N-cap residue (Figure 3A) and a hydrophobic staple motif located at the N-terminal part of α6 helix. The N-capping box motif has been proposed to participate to the nucleation of helices as well as their folding and stabilization by forming reciprocal main chain-side chain hydrogen bonds between the N-cap (Ser/Thr) and the N3 (Glu/Asp) residues. The hydrophobic staple motif consists of a specific i,i+5 hydrophobic interaction between a residue (N′) that precedes the N-cap residue and a residue (N4) located within the α6-helix. The nomenclature commonly used is as follows: N-N′-Ncap-N1-N2-N3-N4 (XhS/TXXDh, with h: hydrophobic residue and X: non conserved residue) (Richardson and Richardson, 1988). These two local structural motifs have a critical role in protein folding and stability of α-helices. The substitution of the capping residue greatly destabilizes the structure of GSTs, as well as their folding. It has also been proposed that the hydrophobic staple motif represents an evolutionarily conserved determinant for rapid folding of the enzyme. In addition, a glycine residue located four amino acids upstream (three residues in GSTLs) the N-cap residue (S/T) is also well-conserved in GSTs and is likely essential for folding by stabilizing the GXXh(S/T)XXDh conserved loop-helix substructure (Kong et al., 2003) demonstrating the importance of these motifs both for protein folding and stability. Concerning the residues involved in the H site, they are generally hydrophobic and are located in a crevice between the N- and C-terminal domains at the vicinity of the G site. The nature of the amino acids contributing to the substrate recognition in this H-site has not been identified so often since it generally requires the crystal structure of complexes. Moreover, from known examples, they are quite variable among GST classes which likely explain the diversity of substrates accommodated by the different GSTs but may at the same time also explain the lack of specificity among certain classes for some substrates (Wilce and Parker, 1994; Armstrong, 1997). For these reasons, we will not discuss in detail the structure and residues forming the H site in each GST class.

With the exception of a few classes such as GSTLs, DHARs and soluble CLICs that exist as monomeric enzymes (Figures 5A,B) (Dixon et al., 2002), other GSTs are mostly dimeric proteins and very often adopt the same dimerization mode. Both subunits are connected along a structural C2 axis roughly parallel to helix bundle axis (binary axial symmetry). The main interactions between the two subunits are held between the N-terminal domain of one subunit and the C-terminal domain of the other. Thus, the loop α2-β3, the strand β4 and the helix α3 of one subunit interacts with the helices α4 and α5 of the other subunit as in GSTBs (Figure 5C). This dimer is considered as the classical dimerization mode in GSTs. In Theta, Sigma and Beta members, the interaction surface is rather hydrophilic whereas in Alpha, Phi, Mu, Omega, Pi, Tau, Zeta, and FuA GSTs, the surface is more hydrophobic (Frova, 2006). The hydrophobic interaction is characterized by a hydrophobic “lock-and-key” (or “ball and socket”) motif which holds the two protomers together and which is established due to the side chain of a phenylalanine (or a tyrosine) residue (key) belonging to the α2-β3 loop (Dirr et al., 1994). This residue is inserted into a hydrophobic pocket (lock) located between helices α4 and α5 of the C-terminal domain of the other subunit as shown for human GSTO1-1 (Figure 5D). This particular interaction is absent in Theta, Sigma, Beta, and Tau members and is replaced by an extensive network of polar interactions (Figure 5C) (Armstrong, 1997; Stevens et al., 2000). Beyond the canonical dimer, other dimerization modes have been described for GSTs. For example, FuA GST dimeric arrangement is close to the one observed in the canonical dimer in that their C2 axis is along the same direction (Figure S1A). In FuA GSTs, the two protomers are translated in the interface plane bringing the α-helical domains closer to each other. An additional β-hairpin (β2′−β2″) inserted between α2 and β3 inhibits the formation of the regular GST dimer and acts as a lid over the G site (Mathieu et al., 2012). A GST from the soil bacterium *Ralstonia solanacearum* (PDB code 4KF9) exhibits a similar dimerization mode as GSTFuA. In this case, the β-hairpin (β2′−β2″) is absent but a long C-terminal extension, which extends the β-sheet structure, prevents the formation of the classical dimer (Figure S1B). In *Macaca fascicularis* mPGES-2, the dimerization remains similar to the canonical assembly and occurs through an insertion of two α-helices and two β-strands (α3′β4′β4″α3″) between α3 and α4 that interacts with those of the other monomer (Figure 5E) (Yamada et al., 2005; Yamada and Takusagawa, 2007). However, this insertion seems to be specific to vertebrates and is absent in photosynthetic organisms, which suggests a different organization. In GHRs, the mode of dimerization is completely different, the monomers associate exclusively via their C-terminal domain and notably via a coil of about 20 residues that follows the helix α9 (Meux et al., 2011). Helix α9 is a structural characteristic that is also found in GSTOs (Board et al., 2000), Tau GSTs (Thom et al., 2002), Delta GSTs (Oakley et al., 2001) and GSTLs (Lallement et al., 2014). The 20 C-terminal residues of one monomer mainly interact with the N-terminal end of helix α5 and with the C-terminal end of helix α6 of the other monomer, allowing the formation of a dimer that completely differs from the usual GST dimer (Figure 5F) (Meux et al., 2011). In addition, a recently characterized GST from *Leishmania infantum* (TDR1 protein) does not exhibit the canonical dimerization mode but consists of a unique trimer of subunits each containing two glutathione S-transferase domains (Figure S1C) (Fyfe et al., 2012). While the diversity of GST quaternary structures might still grow with the release and accumulation of structural data, the majority of GSTs adopts the canonical dimeric quaternary structure.

To date, there are 10 structures of bacterial GSTBs (Table 4). A *B. xenovorans* GSTB structure has been obtained in complex with GSH in the G site and the physiological product, 2-hydroxy-6-oxo-6-phenyl-2,4-dienoate, in the H site (Tocheva et al., 2006). Concerning CLICs, structures from three organisms, *Caenorhabditis elegans*, *Drosophila melanogaster*, and *Homo sapiens* are available (Harrop et al., 2001; Littler et al., 2008). Besides, a few structures have been obtained for the other cysteinyl-GSTs but not for DHAR. For mPGES-2s, only the structure of the *M. fascicularis* isoform has been solved (Yamada et al., 2005). Concerning GSTOs, in addition to one structure from *Bombyx mori* GSTO3 (Chen et al., 2011), several structures are known for human GSTO1 and GSTO2, alone or in complex with GSH or some substrates (Table 4). Recently, the first 3D structures of GSTLs (poplar GSTL1 and L3) in complex with glutathione have been solved (Lallement et al., 2014). Finally, a few GHR/xi GST structures have been solved from various organisms, PcGHR1/Xi GST from *P. chrysosporium*, YqjG from *E. coli*, and its ortholog from *Corynebacterium glutamicum*, *Gordonia bronchialis*, and *Sphingobium chlorophenolicum* namely PcpF, but none from plants (Meux et al., 2011; Green et al., 2012).

**Table 4:** **Tridimensional structures of Cys-GSTs from all kingdoms**.

**Class**	**Name**	**Organism**	**Ligand 1**	**Ligand 2**	**PDB**	**References**
?	TDR1	*L. infantum*	GSH	1,2-Ethanediol	4AGS	Fyfe et al., 2012
?	LigG	*S. paucimobilis*	GSH	SO^2-^_4_; Acetate	4G10	Meux et al., 2012
GSTB	BphK	*B. xenovorans*	GSH	2-Hydroxy-6-oxo-6-phenylhexa-2,4-dienoic acid	2DSA	Tocheva et al., 2006
GSTB		*E. coli*	GTS	–	1A0F	Nishida et al., 1998
GSTB		*M. haemolytica*	GSH	Triethylene glycol; Cl^−^; Acetate	4IW9	Unpublished
GSTB		*M. capsulatus*	GSH	Glycerol	3UAR	Unpublished
GSTB		*O. anthropi*	GSH	SO^2-^_4_	2NTO	Federici et al., 2007
GSTB		*P. mirabilis*	GSH	–	1PMT	Rossjohn et al., 1998
GSTB		*S. flexneri*	GSH	–	4KGI	Unpublished
GSTB		*S. paucimobilis*	GSH	–	1F2E	Unpublished
GSTB		*X. fastidiosa*	GSH	Cl^−^	2X64	Unpublished
GSTB		*Y. pestis*	GSH	Glycerol	4G9H	Unpublished
CLIC	EXC-4	*C. elegans*	–	Ca^2+^	2YV9	Littler et al., 2008
CLIC		*D. melanogaster*	–	Ca^2+^; I^−^	2YV7	Littler et al., 2008
CLIC	CLIC1	*H. sapiens*	GSH	–	1K0N	Harrop et al., 2001
CLIC	CLIC4	*H. sapiens*	–	–	2AHE	Littler et al., 2004
CLIC	CLIC2	*H. sapiens*	GSH	–	2R4V	Cromer et al., 2007
CLIC	CLIC3	*H. sapiens*	–	SO^2-^_4_	3FY7	Littler et al., 2010
GRX	Grx2	*E. coli*	–	–	1G7O	Xia et al., 2001
GRX	Grx2	*S. enterica*	GSH	SO^2-^_4_; Cl^−^	3IR4	Unpublished
GSTO^*^	GSTO3-3	*B. mori*	–	Glycerol	3RBT	Chen et al., 2011
GSTO	GSTO1-1	*H. sapiens*	GSH	SO^2-^_4_	1EEM	Board et al., 2000
GSTO	GSTO2-2	*H. sapiens*	GSH	Cl^−^	3Q19	Zhou et al., 2012
mPGES-2		*M. fascicularis*	–	Indomethacin; Cl^−^; Acetate	1Z9H	Yamada et al., 2005
GHR	YqjG	*E. coli*	–	GS-menadione	4G0K	Green et al., 2012
GHR		*C. glutamicum*	–	1,2-Ethanediol; Glycerol	3M1G	Unpublished
GHR		*P. chrysosporium*	GSH	–	3PPU	Meux et al., 2011
GHR	PcpF	*S. chlorophenolicum*	–	–	4FQU	Green et al., 2012
GHR		*G. bronchialis*	–	–	4PTS	Unpublished
GSTL	GSTL3	*P. trichocarpa*	GSH	Ca^2+^	4PQI	Lallement et al., 2014
GSTL	GSTL1	*P. trichocarpa*	GSH	Na^+^	4PQH	Lallement et al., 2014

Available Cys-GST structures have been retrieved from the RCSB Protein data bank (http://www.rcsb.org/pdb/home/home.do). Only the first solved structures of wild-type isoforms have been listed, but for several proteins as human CLIC or GSTOs or E. coli YqjG, other structures have been obtained either for mutated proteins or for wild-type proteins in complex with GSH or another second ligand. For instance, in the case of E. coli YqjG, there are 3 structures described in the same study, one in apoform, one with GSH and one with GS-menadione. Beyond GSH, structures of Cys-GSTs with physiological substrates have been obtained in rare cases. The compounds indicated in the ligand 2 column essentially come from crystallization solutions.

* GSTO3-3 from B. mori is phylogenetically related to and classified as GSTOs although it displays an asparagine instead of the catalytic cysteine. GTS is for glutathione sulfonate.

### Enzymatic properties and physiological roles

As already mentioned, owing to the presence of a catalytic cysteine residue, Cys-GSTs have particular enzymatic properties since they should in principle catalyze deglutathionylation reactions by performing nucleophilic attacks on various GSH-conjugated substrates (Board et al., 2000; Dixon et al., 2002; Meux et al., 2011). Accordingly, most if not all GSTLs, GHRs, GSTOs and DHARs characterized so far exhibit thiol-transferase and DHAR activities but no transferase, peroxidase or isomerase activities except for a Beta GST from *Proteus mirabilis*, which possesses a slight peroxidase activity on cumene hydroperoxide (*k*_cat_ around 0.01 s^−1^) and a non-negligible GSH transferase activity on CDNB (*k*_cat_ around 2 s^−1^) (Table 5) (Federici et al., 2010). This was surprising since the transferase, peroxidase or isomerase activities are usually specific to Ser- or Tyr-containing GSTs as Phi, Tau, and Zeta GSTs. Indeed, it necessitates the activation of thiolate form of glutathione for direct glutathionylation reaction toward non-conjugated substrates (Dirr et al., 1994; Armstrong, 1997; Roxas et al., 1997). In the absence of known physiological substrates, hydroxyl-ethyl disulfide (HED), and DHA are often used to characterize the activity of recombinant GSTs as well as glutaredoxins, but it turns out that most glutathione-dependent oxidoreductases display such activities with very similar kinetic parameters (Table 5). The only notable exception is DHARs, for which DHA reduction is truly relevant. Consistently, they reduce DHA into ascorbate with a better efficiency (*k*_cat_ around 10^4^ s^−1^, *k*_cat_/*K*_m_ around 10^7^ M^−1^.s^−1^) compared to the other enzymes (*k*_cat_ around 10^2^ s^−1^) and to the reduction of glutathionylated-mercaptoethanol, the product formed upon incubation between GSH and HED (*k*_cat_ around 10^2^ s^−1^) (Table 5) (Dixon et al., 2002). The DHAR-mediated DHA reduction follows a ping-pong mechanism (Dixon et al., 2002; Shimaoka et al., 2003).

**Table 5:** **Enzymatic and ligandin activities detected for Cys-GSTs**.

		**GSTL**	**DHAR**	**GHR**	**mPGES-2**	**GSTB**	**GSTO**
Thiol-transferase		10–10^2^a^^b^^c^^	10–10^2^a^^	10^2^–10^3^e^^	?	?	10^2^–10^3^e^^g^^g^^
DHA reductase		10–10^2^a^^b^^c^^	10^3^–10^4abd^	10–10^2^e^^	?	?	10–10^3^e^^g^^g^^
Glutathionylation		nd ^a^^c^	nd ^a^	nd ^e^	?	10–10^3^i^^j^^	nd–1 ^m^
Deglutathionylation	PAP-SG	10^2^–10^3^c^^	?	nd ^e^	?	?	10^3^–10^4^^***^^^g^^g^^n^^
	(Cl)Qui-SG	nd ^c^	?	10^2^–10^3^e^^f^^	?	?	nd ^g^
	TET-SG	nd ^c^	?	?	?	?	10^3^g^^
	Q-SG	1–10^2^b^^c^^	?	?	?	?	?
PGH_2_ isomerization		?	?	?	10^2^ ^*^ ^h^	?	?
PGH_2_ degradation		?	?	?	10^3^ ^**^ ^h^	?	?
Peroxidase		nd ^a^^c^	nd ^a^	nd ^e^	?	0.1 ^k^	nd ^m^
Esterase		0.01–0.1 ^c^	?	nd ^g^	?	?	10 ^****^ ^g^
Ligandin		?	?	?		Antibiotics ^k^^l^	Nitro-phenacyl glutathione ^o^ Tocopherol esters ^p^

The data representing turnover numbers in min^−1^ have been extracted from the following references:

a (Dixon et al., 2002);

b (Dixon and Edwards, 2010b);

c (Lallement et al., 2014);

d (Tang and Yang, 2013);

e (Meux et al., 2011);

f (Lam et al., 2012);

g (Meux et al., 2013);

h (Yamada and Takusagawa, 2007);

i (Allocati et al., 2000);

j (Allocati et al., 2008);

k (Perito et al., 1996);

l (Allocati et al., 2009);

m (Board et al., 2000);

n (Board and Anders, 2007);

o (Brock et al., 2013);

p (Sampayo-Reyes and Zakharyan, 2006).

Nd, not detected; ? not examined;

* GSH-independent activity,

** GSH-dependent activity,

*** a slightly different substrate, acetophenone, was used (Board and Anders, 2007),

**** k_cat_/K_m_ in mM^−1^.min^−1^.

**Figure 6:**
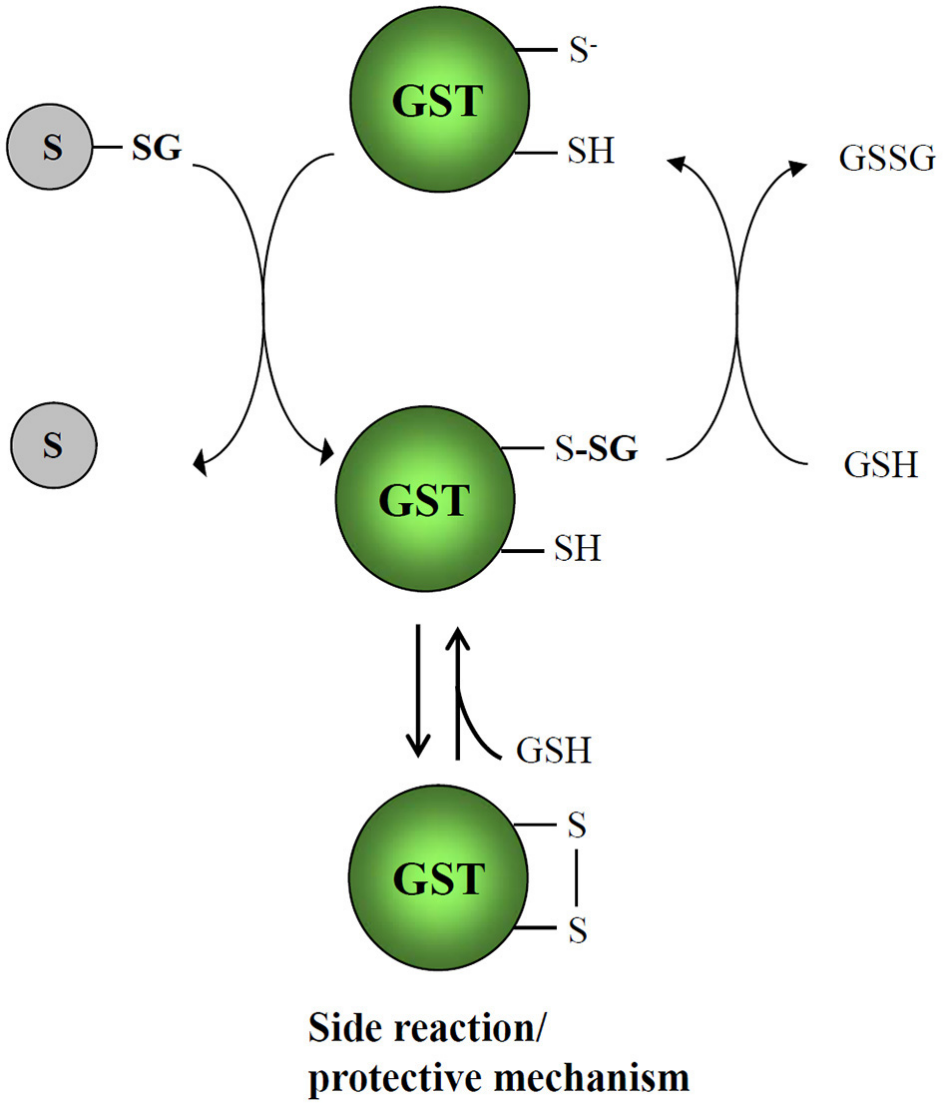
**Catalytic mechanisms of Cys-GSTs**. The deglutathionylation of GSH-conjugated substrates occurs via the nucleophilic attack of the catalytic cysteine which is assumed to be at least partially under the thiolate form at physiological pH, owing to a decreased pKa value. Consequently, the catalytic cysteine is itself glutathionylated and it is regenerated using a glutathione molecule. For Cys-GSTs having another cysteine either in the active site (some DHAR isoforms) or at proximity (some GSTL isoforms), the identification of proteins with an intramolecular disulfide suggests that this might constitute either an intermediate step of the catalytic mechanism or more likely a protective mechanism that prevents oxidation of the catalytic cysteine into sulfenic acid forms or eventually higher oxidized forms as sulfinic or sulfonic acid forms. In the case of the formation of a disulfide an additional glutathione molecule would be required. It may be that thioredoxin participate to this reduction step as DHAR was isolated as a thioredoxin targets.

Based on several previous studies, a proposed catalytic mechanism that should apply for any glutathionylated substrate and any Cys-GST is presented in Figure 6. Since many Cys-GSTs characterized so far either structurally or biochemically have been shown to form mixed disulfides with GSH, there is little doubt that the catalytic cysteine performs a nucleophilic attack on GSH-conjugated substrates. The catalytic cysteine of Cys-GSTs becomes glutathionylated while the product of the reaction is released. The regeneration of these glutathionylated GST forms requires a GSH molecule, forming GSSG as another end product. While reduced Cys-GSTs are ready for another catalytic cycle, GSSG will be reduced back to GSH by glutathione reductase at the expense of NADPH. Since most Cys-GSTs have a single cysteine in the active site motif, they should follow this reaction mechanism. However, a few isoforms have an additional cysteine in the active site. This is the case of some DHAR isoforms which have CPFC active sites. For instance, *A. thaliana* DHAR3 was shown to form an intramolecular disulfide upon GSSG treatment by mass spectrometry (Dixon et al., 2002). Hence, it is possible that it constitutes either an intermediate step of the catalytic mechanism or possibly in other circumstances a protective mechanism that prevents over-oxidation of the catalytic cysteine into sulfenic, sulfinic, or sulfonic acid forms. Whatever the explanation is, the reduction of this disulfide would require a dithiol-disulfide exchange reaction. The most likely possibility is that it involves the successive intervention of two glutathione molecules, but another possibility is that a thioredoxin participates to this reduction step. Indeed, *A. thaliana* DHAR3 was isolated at least in two previous studies aiming at identifying thioredoxin targets (Marchand et al., 2004, 2006).

Besides glutathionylated-mercaptoethanol which contains a sulfur-sulfur bond, other glutathionylated substrates used so far have carbon-sulfur bonds (Meux et al., 2011, 2013; Lam et al., 2012; Lallement et al., 2014). For instance, beyond their DHAR activity, fungal and bacterial GHRs characterized so far efficiently reduce glutathionylated (chlorinated) (hydro)quinones with *k*_cat_ around 10^3^ s^−1^ and *k*_cat_/*K*_m_ up to 10^6^ M^−1^.s^−1^ (Table 5) (Huang et al., 2008; Xun et al., 2010; Lam et al., 2012). However, there are some contrasting data in the literature. Some GHRs seem unable to catalyze the deglutathionylation of GSH conjugated-oxidized quinones and would be specific of glutathionylated reduced forms (Lam et al., 2012). On the other hand, using menadione as a substrate, a fungal GHR proved to deglutathionylate both forms with similar rates, but it is more efficient with the reduced forms because of a much better affinity (Meux et al., 2011). The latter observation points to the importance of the alcohol function for GHR recognition. Despite the above-mentioned discrepancy, it appears that GHRs are central to the regulation of the quinone redox state, likely preventing toxicity of quinones, either naturally present or found as environmental pollutants. Indeed, benzoquinones can covalently react with diverse macromolecules whereas hydroquinones, conjugated or not with glutathione, are prone to auto-oxidation forming reactive oxygen species. Since the major quinone forms found in the cells, ubiquinone and plastoquinone, are located into membranes and do not have electrophilic carbon groups that could be substituted by GSH, the question of the GHR physiological substrates is still open. Several other compounds often derived from lipids or fatty acids have alcohol functions and reactive electrophilic groups that might constitute possible substrates. As explained below, strategies aiming at identifying physiological substrates/ligands have been recently developed for other GSTs and they should be applied to GHRs. It is also possible that GHRs have protein substrates. For instance, it was proposed that the role of *S. cerevisiae* GTO1 could be related to the redox regulation of a Str3 cystathionine beta-lyase (Barreto et al., 2006). To date, the activity assays clearly separate GHRs from GSTLs and GSTOs which often catalyze the reduction of the same glutathionyl derivatives. GSTLs and GSTOs do not catalyze the deglutathionylation of glutathionylated quinones (Meux et al., 2013; Lallement et al., 2014). However, contrary to GHRs, GSTOs and GSTLs perform deglutathionylation of glutathionyl tetralone and/or acetophenone-derivatives with relatively good catalytic constants (*k*_cat_ around 10^4^ s^−1^) (Table 5) (Meux et al., 2013; Lallement et al., 2014) and they exhibit a weak esterase activity on the fluorescent probe chloromethyl fluorescein diacetate (CMFDA) (Meux et al., 2013; Lallement et al., 2014). This probe was initially used to identify tetralone as a GSTO substrate by competition experiments. Incidentally, one of the reported difference between GSTLs and GSTOs is that only GSTOs have the ability to remove the bound GSH molecule on glutathionyl tetralone (Meux et al., 2013; Lallement et al., 2014).

One of the major current challenge concerning Cys-GSTs and other GSTs is to identify relevant physiological substrates. One possibility to achieve this goal is to screen chemical libraries or cellular extracts by competition assays using fluorescent probes such as CFMDA or 8-anilino-1-naphthalenesulfonic acid (ANS). This has been successful in several cases both for Cys-GSTs (Son et al., 2010; Meux et al., 2013) and for those having a catalytic serine (Mathieu et al., 2012, 2013). Besides, Dixon and co-workers have identified several flavonoids derived from kaempferol which can bind tightly to GSTLs from Arabidopsis and wheat by ligand fishing approaches (Dixon and Edwards, 2010a). These approaches consist in isolating by affinity chromatographies and identifying natural physiological substrates from plants using *in vitro* and *in vivo* approaches. Both methods rely on the use of tagged proteins either by mixing them with crude or fractionated extracts or to secondary metabolite enriched-extracts, or by expressing them *in planta* in order to really trap physiological protein-substrate complexes. They proved to work also with Phi and Tau GSTs, the latter binding porphyrin intermediates and fatty acids (Dixon et al., 2008, 2011; Dixon and Edwards, 2009). It was then confirmed by enzymatic analyses that GSTLs from Arabidopsis, wheat and poplar can perform deglutathionylation of glutathionylated quercetin (Dixon and Edwards, 2010b; Lallement et al., 2014). However, the fact that the turnover numbers are quite low and similar to those obtained with other oxidoreductases (Grxs, Trxs, GSTOs, GHRs, and DHARs) from various organisms (Lallement et al., 2014) and that a quercetin derivative was also isolated from a ligand fishing experiment performed with a Phi GST (Dixon et al., 2011) raises the question of a specific role of GSTLs in quercetin recycling and in the maintenance of a reduced flavonoids pool. Overall, this may indicate that flavonoids are GST substrates, but it does not tell exactly which enzyme(s) is (are) really efficient *in vivo*.

From a biochemical point of view, mPGES-2 can hardly be compared with other Cys-GSTs. Indeed, although it has been shown that they do not display GSH transferase activity, none of the usual activities of Cys-GSTs were assayed (Watanabe et al., 1999). Since mammalian mPGES-2s have a defined role in prostanoid metabolism, all studies primarily investigated the PGH_2_ conversion into PGE_2_ (Watanabe et al., 1997). However, an issue was the observation that mPGES-2 activity was partially independent from glutathione and that DTT induced a 4-fold better efficiency of the proteins (Tanikawa et al., 2002). A recently solved structure of a heme-bound mPGES-2 allowed solving this discrepancy. Indeed, it seems that the isomerization activity is catalyzed by a heme-free enzyme, whereas heme-bound mPGES-2s can degrade PGH_2_ into hydroxyl heptadecatrienoic acid and malondialdehyde, instead of converting it to PGE_2_ (Jania et al., 2009; Takusagawa, 2013). This activity relies to the binding of a heme, which is stabilized by hydrogen bonds when a glutathione is present in the active site (Yamada and Takusagawa, 2007; Takusagawa, 2013). Hence, this may help explaining that the activity of isomerization is increased by adding DTT as it contributed to remove both GSH and heme from the active site. Overall, the current view in animals is that the physiological role of mPGES-2s is related to the degradation of PGH_2_ rather than to its isomerization into PGE_2_. This is also consistent with the fact that mPGES-1s also catalyze the GSH-dependent PGH_2_ isomerization into PGE_2_ with good efficiencies (Thoren et al., 2003). Since there is no PGH_2_ and PGE_2_ in plants, the physiological roles and substrates of mPGES-2 are unclear. Looking for possible related candidate molecules in plants, oxylipins might constitute such substrates. Indeed, these molecules, derived from the enzymatic and non-enzymatic peroxidation of fatty acids, exhibit a reactive carbonyl structure, which makes them highly reactive electrophilic species and they are formed at proximity of mPGES-2 localization (Farmer and Mueller, 2013). These compounds participate to numerous developmental processes and to stress response. It is for instance documented that the expression of some GSTs is induced by 12-oxo-phytodienoic acid (OPDA), a phytohormone precursor, and phytoprostane A1 (PPA_1_) (Mueller et al., 2008). Consequently, it was hypothesized that GSTs might reduce the reactive cyclopentenone ring to an unreactive ring. Hence, by glutathionylating or deglutathionylating these molecules, GSTs and possibly mPGES-2s could modulate the concerned signaling pathways. To conclude, although Cys-GSTs are encoded by multigenic families, there is a pressing need to perform reverse genetics by systematically generating single or multiple mutant lines or overexpressing lines to delineate the exact function of these proteins.

## Materials and methods

### Sequence retrieval, structural alignment and phylogenetic analyses

Sequences have been retrieved by iterative blastp analyses using a set of as variable GST sequences as possible either from the cyanobase (http://genome.microbedb.jp/cyanobase/) for cyanobacteria, from the jgi genome portal (http://genome.jgi.doe.gov/) for most algae and from the version 10 of the phytozome portal (http://phytozome.jgi.doe.gov/pz/portal.html) for terrestrial plants. When needed and possible, sequences have been completed and validated by analyzing the presence of ESTs using tblastn analyses against the NCBI protein databank. Sequences were then aligned with PROMALS3D (http://prodata.swmed.edu/promals3d/promals3d.php) (Pei et al., 2008) and alignment manually adjusted with Seaview software (Gouy et al., 2010). The phylogenetic tree was constructed with BioNJ (Gascuel, 1997) in Seaview and edited with Figtree software (http://tree.bio.ed.ac.uk/software/figtree/).

### Subcellular localization

The GST subcellular localization was defined based on the available literature, as well as from database mining using TAIR v10, and from the following prediction softwares, Predotar, TargetP, and Wolfpsort.

### Expression analyses

For developmental conditions, expression data from Gene Expression Map of Arabidopsis Development (Schmid et al., 2005) were retrieved using the eFP browser (Winter et al., 2007). Each gene was normalized to its maximum expression within the selected dataset. Further, the number of developmental conditions was reduced to eight classes in order to gain a better overview of the overall expression profile of each GST during Arabidopsis development. These classes were grouped together and their relative expression averaged as follows: Mature seeds (stages 8, 9, and 10 without siliques and dry seed), Developing seeds (stages 3, 4, and 5 with siliques), Pollen (mature), Flowers (stages 9, 10/11, 12, and 15), Senescing leaves, Leaves (rosette leaves 4, 6, 8, and 10), Shoot Apices (vegetative, transition, and inflorescence), and Roots (from seedlings and mature rosettes).

For perturbation conditions, expression data for each GST were obtained from Genevestigator V3 (Hruz et al., 2008). Only data with a *p*-value < 0.001 were included in the analysis. Perturbations in the resulting lists were grouped into 11 classes: Biotic stress (Bs), Chemicals (Che), Germination (Ge), Light (Li), N-starvation (-N), Fe-deficiency (-Fe), Salt stress (Sa), Hypoxia (Hy), Drought (Dr), Heat (He), and Cold (Co).

Data were analyzed using Open Office Calc (Apache), graphed using PRISM (GraphPad) and clustered using Multi-experiment Viewer (MeV).

## Author contributions

Pierre-Alexandre Lallement, Arnaud Hecker, and Nicolas Rouhier carried out the *in silico* genome analyses. Bastiaan Brouwer and Olivier Keech performed the transcriptome and subcellular prediction analyses. All authors participated to the writing of the manuscript, have read and approved the final manuscript.

### Conflict of interest statement

The authors declare that the research was conducted in the absence of any commercial or financial relationships that could be construed as a potential conflict of interest.
