# Supplementary material for: The still mysterious roles of cysteine-containing glutathione transferases in plants
Source: Front Pharmacol. 2014 Aug 20;5:192. doi: 10.3389/fphar.2014.00192 (PMC4138524; doi:10.3389/fphar.2014.00192)
Supplement: Supplementary file 1 [file Presentation1.PDF]

Supplementary material

Figure S1: Representative GST structures with atypical oligomerization
The structures are shown as cartoon. In **A** and **B**, the N-terminal domain (thioredoxin-like domain) and the C-terminal domain are respectively colored in cyan and in purple. In **C**, each subunit is colored. Glutathione (GSH) is represented as sticks. C2 (A and B) or C3 (C) symmetry axes are shown. Figures have been prepared with Pymol software.
A. GSTFuA dimer from *P. chrysosporium* (PDB code 4G19) in which each monomer interacts via the C-terminal domain of the other monomer. A β2'β2'' β-hairpin (colored in red) inserted between α2 and β3 prevents the formation of the classical dimer.
B. Dimeric GST (PDB code 4KF9) from *R. solanacearum* exhibiting a long C-terminal extension (colored in red) which extends the β-sheet structure of the protein and prevents the formation of the classical dimer.
C. Trimeric organization of TDR1 from *L. infantum* (PDB code 4AGS). TDR1 consists of a trimer of subunits each containing two linked glutathione S-transferase domains (domains I and II). The positions of the GST-like dimers between the domain I of one subunit and the domain II of the adjacent subunit are indicated by the dotted circles.

Figure S1

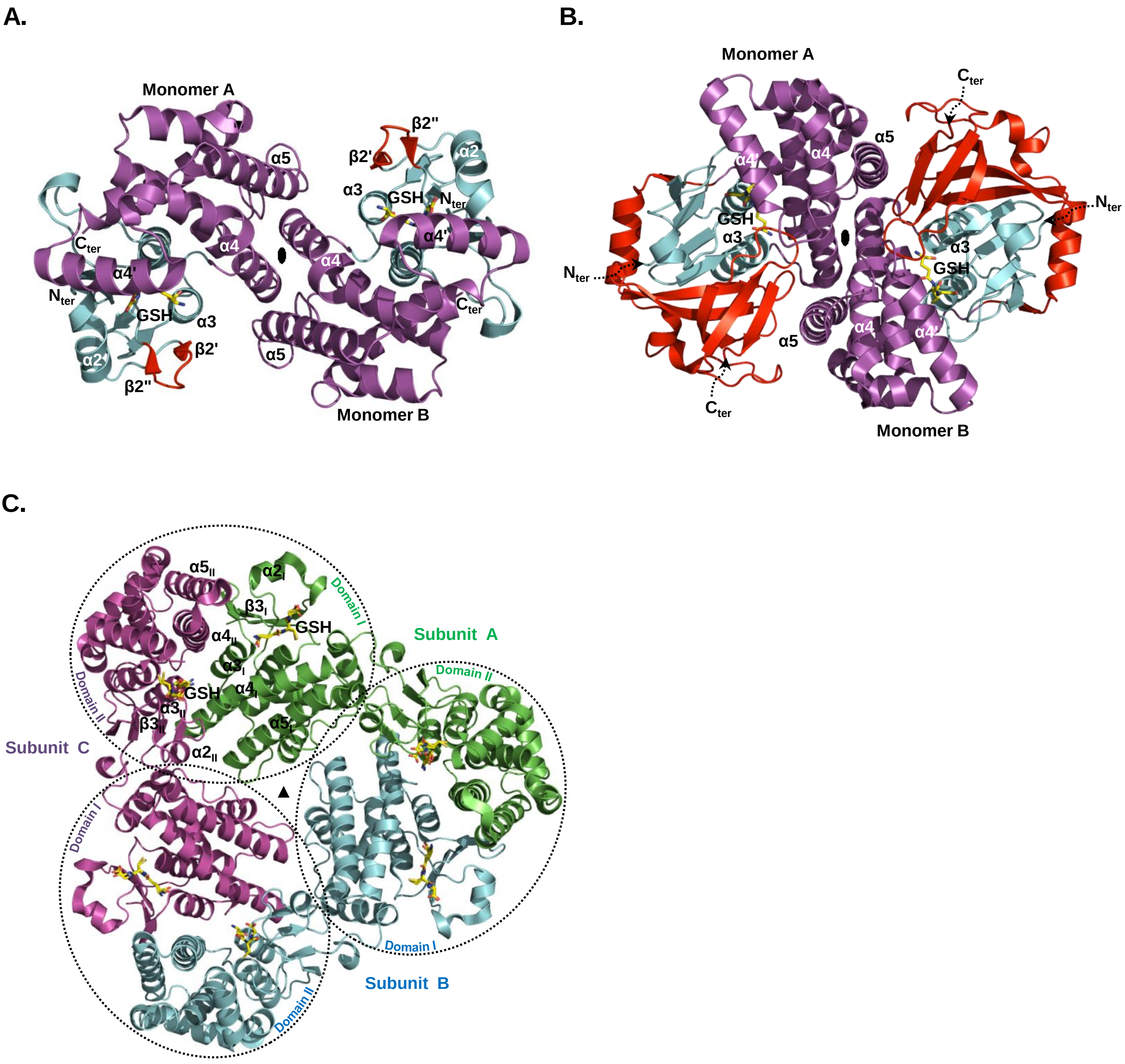
A.
Monomer A
Monomer B
α5
α4
α4'
α3
α2
β2'
β2''
GSH
N_{ter}
C_{ter}
B.
Monomer A
Monomer B
α5
α4
α4'
α3
GSH
N_{ter}
C_{ter}
C.
Subunit A
Subunit B
Subunit C
Domain I
Domain II
$α5_{II}$
$α2_{I}$
$β3_{I}$
GSH
$α4_{II}$
$α3_{I}$
$α4_{I}$
$α3_{II}$
$β3_{II}$
$α5_{I}$
$α2_{II}$

1. Sequences used for the phylogenetic analysis of GSTs (Cys-GSTs and other with serine) found in the selected photosynthetic organisms listed in the legend of Figure 1.

>AtDHAR1@AY039590
PDHLGDCPFSQRALLTLEEKSLYKIHLINLSDKPQWFLDISPQKVPVLKIVTDSDVIVGI
LEEKYPDPPLKPAEFASVGSNIFGTFGTFLKSKDSNDGSEHALLVELEALENHLKSHDGP
FIAGERVSAVDLSLAPKLYHLQVALGHFKSWSVPESFPHVHNYMKTLFSLDSFEKTKTEE
K
>AtDHAR2@AY140019
PDVLGDCPFSQRVLLTLEEKKLYKTHLINVSDKPQWFLDISPEKVPVVKLVADSDVIVGL
LEEKYPEPSLKPPEFASVGSKIFGAFVTFLKSKDANDGSEKALVDELEALENHLKTHSGP
FVAGEKITAVDLSLAPKLYHLEVALGHYKNWSVPESLTSVRNYAKALFSRESFENTKAKK
E
>AtDHAR3@AY065124
PNKLGYCPFCQKVLLTMEEKNVYDMKMVDLSNKPEWFLKISPEKVPVVKFVPDSDVITQA
LEEKYPEPPLAPPEKASVGSKIFSTFVGFLKSKDSGDGTEQVLLDELTTFNDYIKDNGPF
INGEKISAADLSLAPKLYHMKIALGHYKNWSVPDSLPFVKSYMENVFSRESFTNTRAETE
>AtDHAR4@NM@123018
PDVLGDCPFGQRILLTLEDKKLYKTHLIDVSLKPDWFLAISPKKLPLVKFDVADSDLIVG
IIEEKYPEPSLVPPEFASVGSKIIGAFVMFLTSKHANDGSDMALLDELEALDHHLKTHVG
PFVAGDKVTVVDLSLAPKLYHLETTLGHFMDWCVPESLTNVRDYMKVLFSLESFEKTKAA
KE
>AtGSTF1
TVKLYGMAYSTCTKRVYTTAKEIGVVKIVPVDLMKGEHKEPAYLDYHPFVIPVLEDEDGT
KIYESRAISRYLVAKYGKGSSLPSPKAYGLFEQAASVEYSSFDPPASSLAYERVFAGMRG
LKTNEELAKKYVDTLNAKMDGYERILSKQKYLAGNDFTLADLFHLPYGAMVAQLEPTVLD
SKPHVKAWWAASLRVIPGRLLRNSSKEFM
>AtGSTF2@AY039580
GIKVFGHPASIATRRVLIALHEKNLFELVHVELKDGEHKKEPFLSNPFQVPAFEDGDLKL
FESRAITQYIAHRYENQGTNLLSKNISQYAIMAIGMQVEDHQFDPVSKLAFEQIFKSIYG
LTTEAVVAEEEAKLAKVLDVYEARLKEFKYLAGETFTLTDLHHIPAIQYLLGTPTKKLFT
ERPRVNEWVAEITKRPASEKVQ
>AtGSTF3@BT024921
GIKVFGHPASTSTRRVLIALHEKNLFELVHVELKDGEHKKEPFLSNPFQVPAFEDGDLKL
FESRAITQYIAHRYENQGTNLLSKNIAQYAIMSIGIQVEAHQFDPVSKLAWEQVFKFNYG
LNTQAVVAEEEAKLAKVLDVYEARLKEFKYLAGETFTLTDLHHIPVIQYLLGTPTKKLFT
ERPRVNEWVAEITKRPASEKVL
>AtGSTF4@BT005712
GYKVHGDPFSTNTRRVLAVLHEKRLYEPITVKLQTGEHKTEPFLSNPFQVPVFEDGSVKL
YESRAITQYIAYVHSSRGTQLLSHETMATLTMWMEIEAHQFDPPSKLTWEQVIKPIYGLE
TQTIVKENEAILEKVLNIYEKRLEESRFLACNSFTLVDLHHLPNIQYLLGTPTKKLFEKR
SKVRKWVDEITSREAWKMACDQEKSWFNKPR
>AtGSTF5@NM_100175
EYKIYGYPYSTNTRRVLAVLHEKGLYDPITVNLIAGDQKKPSFLANPFQVPVFLDGGLKL
TESRAISEYIATVHKSRGTQLLSYKTMGTQRMWMAIESFEFDPLSTLTWEQSIKPMYGLK
TYKVVNETEAKLEKVLDIYEERLKNSSFLASNSFTMADLYHLPNIQYLMDTHTKRMFVNR
PSVRRWVAEITARPAWKRACDVKAWYHKKK
>AtGSTF6@AY050332
GIKVFGHPASTATRRVLIALHEKNVFEFVHVELKDGEHKKEPFILNPFKVPAFEDGDFKI
FESRAITQYIAHEFSDKGNNLLGKDMAIIAMGIEIESHEFDPVSKLVWEQVLKPLYGMTT
KTVVEEEEAKLAKVLDVYEHRLGESKYLASDHFTLVDLHTIPVIQYLLGTPTKKLFDERP
HVSAWVADITSRPSAQKVL
>AtGSTF7@AY062642
GIKVFGHPASTATRRVLIALHEKNLFEFVHIELKDGEHKKEPFIFNPFKVPAFEDGDFKL
FESRAITQYIAHFYSDKGNQLVSKDIAGIAMGIEIESHEFDPVSKLVWEQVLKPLYGMTT
KTVVEEEEAKLAKVLDVYEHRLGESKYLASDKFTLVDLHTIPVIQYLLGTPTKKLFDERP
HVSAWVADITSRPSAKKVL
>AtGSTF8@NM_180148
SIKVHGVPMSTATMRVLATLYEKDLFELIPVDMRAGAHKQEAHLANPFQIPALEDGDLTL
FESRAITQYLAEEYSEKGEKLICKKVKATTNVWLQVEGQQFDPNSKLAFERVFKGMFGMT

TPAAVQELEGKLQKVLDVYEARLAKSEFLAGDSFTLADLHHLPAIHYLLGTDSKVLFDSR
PKVSEWIKKISARPAWAKVIDLQKQ
>AtGSTF9@AF372905
VLKVYGPHFASPKRALVTLIEKGVFETIPVDLMKGEHKQPAYLAQPFTVPAVVDGDYKIF
ESRAVMRYVAEKYRSQGPDLLVEDRGQVEQWLDVEATTYHPPLNLTLHIMFASVMGFPSE
KLIKESEEKLAGVLDVYEAHLSKSKYLAGDFVSLADLAHLPFTDYLVGPIGKAYMIKDRK
HVSAWWDDISSRPAWKETVAKYSFPA
>AtGSTF10@AY128398
VLTIYAPLFASSKRAVVTLVEKGVFETVNVDLMKGEQRQPEYLAQPFKIPVLVDGDYKIF
ESRAIMRYIAEKYRSQGPDLLIEERGQVEQWLDVEATSYHPPLALTLNIVFAPLMGFPAE
KVIKESEEKLAEVLDVYEAQLSKNEYLAGDFVSLADLAHLPFTEYLVGPIGKAHLIKDRK
HVSAWWDKISSRAAWKEVSAKYSLPV
>AtGSTF11@AY099776
VVKVYGQIKAANPQRVLLCFLEKDIFEVIHVDLDKLEQKKPQHLLQPFQVPAIEDGYLKL
FESRAIARYYATKYADQGTDLLLEGRAIVDQWVEVENNYFYAVLPLVMNVVFKPKSGKPC
VALVEELKVKFDKVLDVYENRLATNRYLGGDEFTLADLSHMPGMRYIMNETSLSGLVTSR
ENLNRWWNEISARPAWKKLMELAAY
>AtGSTF12@AF288189
VVKLYGQVTAACPQRVLLCFLEKGIFEIIHIDLDTFEQKKPEHLLQPFQVPAIEDGDFKL
FESRAIARYYATKFADQGTNLLLEHRAIVDQWADVETYYFNVLQPLVINLIIKPRLGEKC
VVLVEDLKVKLGVVLDIYNNRLSSNRFLAGEEFTMADLTHMPAMGYLMSITDINQMVKAR
GSFNRWWEEISDRPSWKKLMVLAGH
>AtGSTF13@NM@116141
AMKLYGDEMSACVARVLLCLHEKNTFELVPVNLFACHHKLPSFLSNPFKVPALQDDDLTL
FESRAITAYIAEKHRDKGTLTRPKEAAIVKLWSEVEAHHFNPASAVIHQLIVVPLQGESP
AAIVEENLENLGKILDVYEERLGKTKYLAGDTYTLADLHHVPYTYYFMKTIHAGLINDRP
NVKAWWEDLCSRPAFLKVSPGLTVAPTTN
>AtGSTF14@AK176306
KMKLHCGFIWGNSAALFCINEKGLFELVFVDWLAGEAKTKTFLSNPFEVPVLEDGDLKLF
EPKAITRYLAEQYKDVGTNLLPKKRAIMSMWMEVDSNQFLPISTLIKELIINPYQGLATD
TAVQENKEKLSEVLNIYETRLGESPYLAGESFSLADLHHLAPIDYLLNTDEEELKNLIYS
RPNVAAWVEKMKMRPAWLKTVVMKNHIVDLMK
>AtGSTL1@NM_120356
TTRLYISYTCPFAQRVWITRNLKGLDEIKLVPIDLPNRPAWLKEVNPAKVPALEHTGESL
DLIKYVDSNFDGPSLYSAKREFGEELLKYVDETFVKTVFGSFKGDPVKETASAFDHVENA
LKKFDDGPFFLGELSLVDIAYIPFIERFQVFLDEVFKYEIIIGRPNLAAWIEQMNKMVAY
TQTKTDS
>AtGSTL2@AK118180
STRLYISYTCPFAQRAWIARNYKGLNKIELVPIDLKNRPAWYKEVYSAKVPALEHLGESL
DLIKYIDTNFEGPSLTLEKQVVADELLSYTDSFSKAVRSTLNGTDTNAADVAFDYIEQAL
SKFNEGPFFLGQFSLVDVAYAPFIERFRLILSDVMNVDITSGRPNLALWIQEMNKIEAYT
ETRQDP
>AtGSTL3@AY140069
TTRLYTSYVCPFAQRVWITRNFKGLEKIKLVPLDLGNRPAWYKEVYPEKVPALEHIGESL
DLIKYLDNTFEGPSLYHAKREFGDELLKYTDTFVKTMYVSLKGDPSKETAPVLDYLENAL
YKFDDGPFFLGQLSLVDIAYIPFIERFQTVLNELFKCDITAERPKLSAWIEEINKSDGYA
QTKMDP
>ATGHR@NP_193723.3
RYHLYISYACPWASRCLSYLKIKGLISFSSVHLNGAKSVRELYEIASPNTVPVLWDKKLK
NNESAEIIRMFNTEFNHIAGPDLSHLQAKIDETNEWIYNGINNGYRCGFAKKQGPYEEAV
EQVYEALDRCEEILGKHRYICGNTLTETDIRLFVTLIRFDEVCNKKLIREYPNLFNYTKD
IFQIPGMSSTVNMNHIKQHYYG
>ATGHR@NP_199315
RYHLYISYACPWACRCLSYLKIKGLITFSSVYLNGAKSVRELYEIASPNTVPVLWDKKLK
NNESSEIIRMFNTEFNGIAKPSLSHLRDVINETNGWVFNGINNGYKCGFARKQEPYNEAV
NQLYEAVDRCEEVLGKQRYICGNTFTEADIRLFVTLIRFDEVCNKRLLREYPNIFNYIKD
IYQIHGMSSTVNMEHIKQHYYG
>ATGHR@ABK28743
RYHLYISYACPWASRCLAILKLKGLISFSSVHINGAKSVRELYDIASSNTVPVLWDKKLK
NNESSEILRMFNTEFNHVAEPSLPNLRAIIDETNEWIHDGINNGYKCGFATNQETYDVEV
KRLYEALDRCEDILRKQRFLCGNTLTESDIRLFVTVIRFDEACDKRLVREYYHLFNYTKD

IYQIAGMSSTVKMDHIKQNYYG
>ATGHR@NP_568632
ELHLYVGLPCPWAHRTLIVRALKGLVPVSIAKANRCRNLKEVYKSRSGGTVPMLWDLRKK
CNESYDIIEFFNSGLNKLARDNLPELKEMIQGWNQIVYPKVNNGYRCGFAQSQEAYDGAV
NELFSTLDEIEDHLGSNRYLCGERLTLADVCLFTTLIRFDSVCTKKKLVEYPNLYGYLRE
IYQIPGVAATCDISAIMDGYYK
>AtGSTT1@AY054659
KLKVYADRMSQPSRAVIIFCKVNGIFDEVLISLAKRQQLSPEFKDINPLKVPAIVDGRLK
LFESHAILIYLSSAFPSVADWYLSKRAKIHSVLDWHHTNLRRGAAGYVLNSVLGPALGLP
LNPKAAAEAEQLLTKSLSTLETFWLKGNAKFLLGSNQPSIADLSLVCELMQLQVLDDKDR
LRLLSTHKKVEQWIENTKKMPHFDETHEILFKVKEGFQ
>AtGSTT2@AJ132398
KLKVYADKMSQPSRAVLIFCKVNEIFDEILISLGKRQQLSPEFKEINPMKVPAIVDGRLK
LFESHAILIYLSSAYASVVDWYLSKRAKIHSVLDWHHTNLRPGASGYVLNSVLAPALSLP
LNPKAAAEAENILTNSLSTLETFWLKGSAKFLLGGKQPSIADLSLVCELMQLQVLDDKDR
LRLLSPHKKVEQWIESTRKMPHSDEVHEVLFRAKDRFQ
>AtGSTU1@AF428387
SVKLLGFWASPFSRRVEMALKLKGVYEYLEEDLPNKTPLLLELNPLHKKVPVLVHNDLLE
SHLILEYIDQTWKNSPILPYEKAMARFWAKFIDDQILTLKAEKGREVAIEETRELLMFLE
KEVTKDFFGGKTIGFLDMIAGSMIPFLARLWKGPEEKFPELNRWIKNLEEVEAVRGCIPP
REKQIERMT
>AtGSTU2@AY094455
SVKLLGFWISPFSRRVEMALKLKGVYEYLEEDLPKKSTLLLELNPVHKKVPVLVHNDLSE
SHVILEYIDQTWNNNPILPYEKAMVRFWAKFVDEQILPVKAEKGIDVAIEEIREMLMFLE
KEVTKDFFGGKTIGFLDMVAGSMIPFLARAWECPEDTFPELNRWIKNLNEVEIVRECIPP
KEKHIERMK
>AtGSTU3@AK117612
GVKLIGSWASPFSRRVEMALKLKGVYDYLDEDYLVVKSPLLLQLNPVYKKVPVLVHNGLP
ESQLILEYIDQTWTNNPILPYDKAMARFWAKFVDEQVTMIKSEKRIDVAIEEVQELIMLL
ENQITKKLFGGETIGFLDMVVGSMIPFLARAWEGPEEKFPELNRWIKNLKEIEIVRECIP
DREKHIEHMM
>AtGSTU4@AF387004
DVKLLGFWASPFTRRVEMAFKLKGVYEYLEQDIVNKSPLLLQINPVYKKVPVLVYKGLSE
SHVILEYIDQIWKNNPILPYEKAMALFWAKFVDEQVGPVKAEKGVEVAIKEAQELFMFLE
KEVTKDFFGGKTIGFLDLVAGSMIPFLARGWEDPEEKFPELNRWIKNLKEIEIVRECIPP
REEQIEHMK
>AtGSTU5@AY062676
EVKLLGIWASPFSRRVEMALKLKGIYEYVEEILENKSPLLLALNPIHKKVPVLVHNGILE
SHVILEYIDETWPQNPILPYERSKARFFAKLVDEQIMNVADEKGREVLAEQVRELIMYLE
KELVKDYFGGKTVGFLDFVAGSLIPFLERGWEGTEEKFPEFKRWVRNLEKVEIVKDCVPP
REEHVEHMN
>AtGSTU6@AF288187
EVKLLGIWASPFSRRIEMALKLKGVYEYLEEDLENKSSLLLALSPIHKKIPVLVHNGIIE
SHVILEYIDETWKHNPILPFQRSKARVLAKLVDEKIVNVKTEKGREVLIEQTRELIMCLE
KELAKDYFGGKTVGFLDFVAGSMIPFLERAWEGTEKKFPEYNKWVKKLKEVEIVVDCIPL
REKHIEHMN
>AtGSTU7@AY045679
EVKLLGMWASPFSRRIEIALTLKGVYEFLEQDITNKSSLLLQLNPVHKMIPVLVHNGISE
SLVILEYIDETWRDNPILPYERTMARFWSKFVDEQIYVTKTGKERDAVVEATRDLLMFLE
KELVKDFLGGKSLGFVDIVATLVAFWLMRTEEIPVEKFPEIHRWVKNLLGNDVIKKCIPP
EDEHLKYIR
>AtGSTU8@AY086116
HVKLLGLWGSPFSKRVEMVLKLKGIYEYIEEDVYGNRSPMLLKYNPIHKKVPVLIHNGIA
ESLVIVEYIEDTWKTTHTILPYERAMARFWAKYVDEKVMLAPESEREKEVKEAYEGLKCL
EKELGKLFFGGETIGFVDIAADFIGYWLGIFQEATAEEFPKLQRWSEDFVGNNFIKEVLP
PKEKLVAVLK
>AtGSTU9@AK176211
KVILHGSFASPYSKRIELALRLKSIYQFVQEDLQNKSQTLLRYNPVHKKIPVLVHNGISE
SLFIIEYIDETWSNPHILPYRRSKVRFWANYIQLHLYDLEGEEQKKALTEVKEKLSVIEK
GLKSDTDGEPTVTNETMSLVDIVMCTLLSPYKAHEEVDPEIVPGVYGWINAINETSVVKD
LSPPYEQILEILR

>AtGSTU10@AK117614
KVILHGTWISTYSKRVEIALKLKGVYEYLEEDLQNKSESLIQLNPVHKKIPVLVHDGVAE
SLVILEYIDETWTNPRFFPYERAQVRFWVSYINQQVFEVEGEAQAKSVEEARKRFKVLDE
GLKPNKNIRRNDDVGLLEITIIATLGGYKAHREAGPVNTPTLYNWIERLQDLSVIKEVEV
PHDTLVTFIQ
>AtGSTU11@AK119143
YVKLLGAWPSPFVLRTRIALNLKNVYEYLEEEDTLSSESVLNYNPVHKQIPILIHGNIRE
SLNIVMYVDETWLSPPILPFDRAVARFWDVYIDEHCFTSKGEENINAAIAKLEQCMALLE
ETFQKGRGFFGGENIGFIDIGFGSMLGPLTVLEKFHPENTPGLFHWADRFYAHEAVKPVM
PDIEKLVQFAR
>AtGSTU12@BT010687
TVKLIGTWASPFAIRAQVALHLKSVHEYVEETDVLKGKSDLLIKSNPIHKKVPVLIHGDI
CESLNIVQYVDESWPSLSILPSERAFARFWAHFVDGKLFESKDDAARMTLAGNLMENLAA
LEEAFQKGGDFFGGGNIGFVDITVGAIVGPISVIEAFRPDTTPGLIQWAEKFRAHEAVKP
YMPTVAESIEFAK
>AtGSTU13@AY050343
TVKLIGSWSSPYSLRARVALHLKSVYEYLDEPDVLKEKSELLLKSNPIHKKVPVLLHGDI
SESLNVVQYVDEAWPSPSILAYDRASARFWAQYIDDKCFAAKDDEGKMAAVGKLMECLAI
LEETFQKGLGFFGGETIGYLDIACSALLGPISVIEAFRQETTPGLIKWAERFRAHEAVKP
YMPTVEEVVAFAK
>AtGSTU14@AF288178
TVKLIGCSDDPFSIRPRVALHLKSIYEYLEEPDDDLGEKSQLLLKSNPIHKKTPVLIHGD
ICESLNIVQYLDEAWPSPSILAYDRASARFWAQYIDDKCFEANNDEERIAATGKLTECLA
ILEETFQKGLGFFGGETIGYLDIACAALLGPISVIEMFREETTPGLIQWAVRFRAHEAVR
PYMPTVEEVTELVK
>AtGSTU15@AY084992
QVKLLGTWYSPVVIRAKIDLRLKSVYDYVEENLFGSKSELLLKSNPVYKKVPVLIHNTVC
VSLNIVEYIDETWNSSSSILPYDRALARFWSVFVDDKWLPTKSEEAKAKGMEEVEEGLLQ
LDAAFIKGKSFFGGETIGFIDICLGSFLVLLKAREKLDELKTPSLYRWANQFLSNEMVKN
VVPDIDKVAKFIE
>AtGSTU16@AF370480
EVKLLGVWYSPYAIRPKIALRLKSVYDYVEENLFGSKSELLLKSNPVHKKVPVLLHNNIV
ESLNIVEYIDETWNSSPSILPYDRALARFWSDFVDNKWFPAKSEDAKAKAMEEVEEGLLQ
LEDAFVKGKPFFGGEAIGFMDICFGSFVVLLKAREKFVESKTPSLCKWADRFLSDETVKN
VAPEIEKVAEFLQ
>AtGSTU17@AY091102
DVKLIGAWASPFVMRPRIALNLKSVPYEFLQETFGSKSELLLKSNPVHKKIPVLLHADVS
ESNIIVEYIDDTWSSSSILPYDRAMARFWAAYIDEKWFGGEEEKKAVIAQLEEGNAFLEK
AFIKGKPFFNGDNIGYLDIALGCFLAWLRVTELADEAKTPSLSKWAENFCNDPAVKPVMP
ETAKLAEFAK
>AtGSTU18@AY090957
DVKLIGSWASVYVMRARIALHLKSISYEFLQETYGSKSELLLKSNPVHKKMPVLIHADVC
ESNIIVHYIDEAWNSSSILPYDRAIARFWAAYIDDQWFQGDEEKKAAIAQVEERTKLLEK
AFNQGKPFFNGDHIGYLDIALGSFLGWWRVVELDDETKTPSLVKWAERFCDDPAVKPIMP
EITKLAEFAR
>AtGSTU19@AF385691
EVILLDFWPSMFGMRTRIALREKGVFEYREEDLRNKSPLLLQMNPIHKKIPVLIHNGVNE
SIIQVQYIDEVWSHKNILPYLRAQARFWADFIDKKLYDATKGEEQEAGKKDFIEILKTLE
SELGDKPYFSGDFGYVDIALIGFYTWFPAYEKFESEVPKLIAWVKKCLQRESVAKSLPPE
KVTEFVS
>AtGSTU20@AY136338
LPILLDYWPSMFGMRARVALREKGVFEYREEDFSNKSPLLLQSNPIHKKIPVLVHNGVCE
SLNVVQYVDEAWPEKNFFPYGRAQARFWADFVDKKFTDAKKGEEQEAGKKEFIEAVKILE
SELGDKPYFGGSFGYVDISLITFSSWFQAYEKFESESPKLIAWAKRCMEKESVSKSLPSE
KIVAYAA
>AtGSTU21@NM@106483
EVILLGFWPSMFGMRTMIALEEKGVYEYREEDVINNKSPLLLEMNPIHKTIPVLIHNGVL
ESLIQIQYIDEVWSDNNFLPYHRAQALFWADFIDKKEQLYVCTKGEELEAANKEFIEILK
TLQCELGEKPYFGGKFGFVDIVLIGFYSWFPAYQKFEPECLKLIAWGKRCMQRESVAKAL
PSEKVVGYVL
>AtGSTU22@AY086469

EVILLDFWPSPFGVRARIALREKGVFEYREENLRDKSPLLLQMNPVHKKIPVLIHNGVCE
SMNVVQYIDEVWSDKNILPYQRAQARFWVDFVDTKLFEPTKGEEQETAKKEYIEALKILE
TELGDKPYFGGTFGFVDIAMTGYYSWFEASXKLEPXCPTLMASAKRCLQRESVVQSLHSE
KILAFAY
>AtGSTU23@AY085813
EIILLDYWASMYGMRTRIALEEKKVYEYREEDLSNKSPLLLQMNPIHKKIPVLIHEGICE
SIIQVQYIDELWPDTNILPYQRAQARFWADYIDKKTYVPESGEKQEAAKIEFIEVLKTLD
SELGDKYYFGGEFGLVDIAFIGFYSWFRTYEEVVLEFPKLMAWAQRCLKRESVAKALPSD
KVLKSVS
>AtGSTU24@BT012184
EVILLDFWASMFGMRTRIALAEKRVYDHREEDLWNKSSLLLEMNPVHKKIPVLIHNGVCE
SLIQIEYIDETWPDNNLLPYKRAHAKFWADFIDKKVNVTVKGEEQEAAKELIEILKTLES
ELGDKKYFGDTFGYVDIALIGFHSWFAVYEKFESECSKLVAWAKRCLERESVAKALPSEK
VITFIS
>AtGSTU25@AK118907
EVILLDFWPSMFGMRTRIALEEKNVFDYREQDLWNKSPILLEMNPVHKKIPVLIHNGVCE
SLIQIEYIDEVWPSKTLLPYQRAQAKFWGDFIDKKVYASAKGEEHEAGKKEFIEILKTLE
SELGDKTYFGGTFGYVDIALIGFYSWFEAYEKFEAECPKLIAWGKRCVERESVAKSLPSE
KIIKFVP
>AtGSTU26@NM@101580
QVILLDYWPSMFGMRTKMALAEKGVYEYKETDPWVKTPLLIEMNPIHKKIPVLIHNGICE
SLIQLEYIDEVWSDASILPYQKSRARFWAEFIDKKFYDPTMGEEHAAVKKELLEHFKTLE
TELGDKPYYGGVFGYLDIALMGYYSWFKAMEKFETEFPILTTWTKRCLERESVVKALASD
RIIEYVY
>AtGSTU27@AF370274
EVVVLNFWPSMFGARVIMALEEKEIFEYKEEDVFGQKTDLLLQSNPVNKKIPVLIHNGVC
ESNIIVEYIDEVWKDDKLRLLPYQKSQCRFWADLIDKKVFDAKRGKEQEEAKQEFIEILK
VLERELGDKVYFGGNVSMVDLVLISYYPWFHTWETIEDHTPKLMDWIRKCLTRPAISKSL
PPLKIFDRVT
>AtGSTU28@NM@104246
KVVVLDFWASPYAMRTKVALREKGVFEVQEEDLWNKSELLLKSNPVHKKVPVLIHNNISE
SLIQVQYIDETWTDAAFLPQSRATARFWADYADKTISFEGGKKGEEQEKGKKEFLESLKV
LEAELGDKSYFGGTFGYVDITLVPFYSWFYALEKCEAECPKIVAWGKRCVERNSVAATLP
SEKVYQQVL
>AtGSTZ1@AY052332
KLKLYSYWRSSCAHRVRIALALKGLYEYIPVNLLKGDQFDSDFKKINPMTVPALVDGDVV
INDSFAIIMYLDEKYPEPPLLLHKRAVNYQAMSIVLSGIQPHNLAVIRYIEEKINVEEKT
AWVNNAITKGFTALEKLLVNCAGKHATGDEIYLADLFLAPQIHGAINRFQINMEPYPTLA
KCYESYNELPAFQNALPEKQPDAPSST
>AtGSTZ2@NM@126295
KLKLYSYWRSSCAHRVRIALTLKGLYEYIPVNLLKGDQSDSDFKKINPMTVPALVDGDVV
INDSFAIIMYLDDKYPEPPLLYHKRAVNYQATSIVMSGIQPHNMALFRYLEDKINAEEKT
AWITNAITKGFTALEKLLVSCAGKYATGDEVYLADLFLAPQIHAAFNRFHINMEPFPTLA
RFYESYNELPAFQNAVPEKQPDTPSTI
>AtTCHQD1@AY064034
MQLYHHPYSIDSQRVRLALEEKGIYTSYHVNPITGKHMDPSFFMNPNKLPVFRNGSHIIL
DTIEIIEYLERIAEVATFNREVVEWMRKIREWESKLFTLAHIPDNRRLYVSKFLRMVVIA
RMAESPDLASAYHRKLREAYDTEDKLKDPGALRRSKDHLLRLLDEVETKLEGTTYLAGNE
FSMADVMLIPVLARLSLLDLEEEYISSRKNLAEYWALVRRRPSYKKVIGRYFNGWRKYA
>gi|BAE44477|HvGSTU1
GLVLLDFWVSPFGQRVRIALAEKGLYEYVEEDLIAGKSDRLFRSNPVHKKIPVLLHDGVN
ESLIILNYLDDAFPDAPALLPYARAQARFWADYVDKKVYDCVKGEPQAQARAEMLEVLKT
LDGALGDKPFFGGKFGFVDAAFAPFTAWFHSYERYAEVAPKIAAWAKRCGERESVAKSLY
PEKVYDFIG
>gi|BAJ98279|HvGSTU2
EVVCIDFWANLFGMRVLIALRELGVFKYIEEDLRVCERSDLVLRMNPVHRMVPILIHHGI
CNSINILEYIDEVWGHRQETRLLPLHRAYARFWADFVDNKVCRTTTACFIISYQYLFDHL
YHFFFPAKYVSFTQWLVYSSFALMNYFT
>gi|BAJ90808|HvGSTU3
GELLLKSNPVHMKVPVLIHDGICESLAIVQYVDEVWAATTSILPYDRAAARFWAAYADSK
LLATEEERAEKVGDTLAAIGQLEEAFGNGKPFFAGDSVGYLDLVVGSQLLWFEVLRKMEA

CRVPFLAAWVKRFWETDTAKAVVPDVGTAAEYLK
>gi|BAJ95179|HvGSTU4
DLKLLGAWPSPFVTRVKLALALKGLSYEDVEENLSNKKIPVLIHNGVCESMIIVQYIDDV
FASTSLLPYERAVARFWVAYVDDKLVKTEEEKSEGKKQTFAAVEVLEGALRKGGDFFGGD
GVGLVDVALGGLLSWMKVTDVLDAVKTPLLAAWVERFSAIDAAKAALPDVGRLVEFAK
>gi|BAJ90384|HvGSTU5
KVKLFGMWASPYVLKVKWALSIKGVYEYLEEDLRNKSDDLLEHNPVHKKVPVLLYHGVAE
SDVIVEFVDEAWSHRGRILPYERAMARFWVRFVHDKLSPPAPGEDQEAARGASVEQLQVL
EELLAGGKEFFAGESVGLVDLSLGAMAYVVPMYEEITEERFPSLSAWMGRFLGSPPVKDH
PPPVERLIPRYR
>gi|BAJ99083|HvGSTU6
PVRLIGCFGSPVVHRAELALRLKGVYELVEEDLNNKSELLLTHNPVHKTVPVLLHGHIPE
SLVIVEYVDEAFPAPPLLPLARANARFWARFLEEECKKPDGEAQVKAARETKEKLTLLEA
QLPGKRFFAGDAIGFLDIAVGGVAHWMGVFEEMTEEAHPALCRWAREYRADETVRQCLPD
RARVLAALA
>gi|BAK02040|HvGSTU7
AVTCVDFWANGFGMRVRIALRELGVFDYVEEDLRMGERSDLVRRMNPVHRSVPILIHRGV
CGSLNILDYIDEVWSAPGERRLLPAGRADARFWADFIDHKVFATSKGEEKHVAKEELVEQ
LKRLEEVLGDKEHFSGEFGFLDMVLIPFSSMFRGYEQHEVECPKLMRWVKRCKERESVRA
VLPETKMYELHK
>gi|AK370747|HvGSTU8
EVTVLGHWGSPFVTRVRLALRLKGVRYEYVEEDLRSKSDLLLRCNPVHRAVPVLIHRGVC
ESQAILQYVDEAFGPTLLPHERAVARFWAAYFDDELGGTEEERAAWMKKVVDAADGLERG
LGGGGRKGCFFGGESVGYVDVVVGGTVSYVHAVARVQDEGRTPLLAAWLRRFGELDAAVE
LLQDVDRVVDYVR
>gi|BAK01946|HvGSTU9
EVTVLGHWGSPFVTRVRLALRLKGVRYEYVEEDLRSKSDLLLRCNPVHRAVPVLIHRGVC
ESQAILQYVDEAFGPTLLPHERAVARFWAAYFDDELGGTEEERAAWMKKVVDAADGLERG
LGDGGRKGCFFGGESVGYVDVVVGGAVSYVHAVARVQDEGRTPLLAAWLRRFGELDAAVE
LLQDVDRVVEYVR
>gi|BAJ96773|HvGSTU10
DVKVLGTAASMFAIRVRMALHAKGVSYEYLEQDLFHKGELLLASNPVRKAVPVLIHAGVC
ESLAIVEYIDEVWAGAASLLPYDRAVARFWAAYVDDKAVATEEDRAERLAAALAAVAPLE
DAFAGGKAFFAGDSIGYVDLALGCNLFWIEALRHMDAGRTPRLAAWAERFVETEAAKKAA
PPMESMLEEAE
>gi|BAJ93565|HvGSTU11
DLKLLGMWASPAVLRVRLALSIKGVSYEYVEEDFANKSELLLRSNPVHNMVPVLIHAGVC
ESQLIIQYLDEAFAGDPALLPYERAVARFWAAFVDDTLLKTEEEKAEGRKQVAAALKTLD
GALGGGKPFFGGDGPGYVDTVLGGLLAWVRSMDVIDSVTMPLLAAWADRFGALDAVEAVM
PDVNKIVEFSL
>gi|BAJ94524|HvGSTU12
EVKLLGMWASPFVLRIKLALSLKGVAYEYVEEDLKSKSELLLRSNPVLQKVPVLIHDGVC
ESSVILQYIDEAFAGFSLLPNGRAVARFWAAYIDGTLVKTEEEKAEGAKQVAAAVETLEG
ALRNGKPFFGGDTAGYVDVMLGGLLPWVHTSDKMDPATTPLLAAWADSFGSLGAVEAVMP
DVSKLIEFAM
>gi|BAJ94610|HvGSTU13
GLTLLGFWTSPFALRARFALNLKGIPYEYVEEDLFGERGKSQLLLASNPAHGKVPVLIHG
GVAESLVIVEYVDEAFPESRLLPHGRAAARFWAAYVDQKLLKTAEERARAAGEVVSVLEA
FEGELGKDFFGGDGVGLVDVVLGGFVGWLRASEAMDPARTPRLAAWAGRFGALDGVREIV
PDAAPLVEYNL
>gi|BAJ90106|HvGSTU14
ELTLVGYWSSPFALRARYALNLKGLPYAYVEEVGLFDGKSPLLLASNPVHKRVPVLIHNG
VPESQLIVQYVDEAFPASRFLPHERAVARFWASYVDGELLRTGEERAQAAGRVGAALETL
ERAFGKGKGFFGWDTVGLVDVVLGGFVGWLKTSEAMDAAATPLLAAWAERFRALHGVKEV
MPDPQRLLEYNL
>gi|BAJ99164|HvGSTU15
ELKLLGTWRGPFALRVRLALNFKGLSYEYQEEDLANKSDLLLESNPVNKKVPVLIHNGIC
ESLAILEYIDEVYRGTCLLPYQRAVARFWAAYIDQNLVKTDKEKHEGTKQTLAAVEKLEV
ALRSGKPFFGGDNVGYIDVVLGGMVAWMQGTEALDATKTPLLLAWMERFGGMEPAKAVLP
DIDRLVEFAK
>gi|BAJ90106|HvGSTU16

GLVLLNFWVSPFGQRCRIALAEKGLYEYVEENLMAGKSDRLLRSNPVHKKVPVLLHDGVN
ESLIIILNYLDDAFPDTPSLLPYERAQARFWADYVDKKVYDCLKGEPHAQARAEMVEILKN
LDGALGDKAFFGGTFGFVDAAFVPFTSWFRSYETYAEVAPRIAAWAKRCGERESVAESLY
PDKIYEFVG
>gi|BAJ98487|HvGSTU17
PVVVVGGWASPFVTRVCIALRLKGVEYEFLQEAVGRKSELLLRSNPVYRKMPVLLHAGVC
ESLVILQYVDEAFSAAPILPYRRAVHRFWAEYADAKLHMVGGDKVDAAEQVSAALRQLEE
AFVGGKPYFGGDDVGFLDIVVGSYIGWFGAAERIDQARTPRLAAWAVRFCAHEAVGELVP
DAARLVEFGE
>gi|BAK00368|HvGSTU18
ELQLLGAWMSPYVIRVKVALQMKGLSYDYIEQDLQHKSDLLLSSNPVHKKVPVLIHDGVC
ESLVVLEYVDEAWAGTPLLPYDRAVARFWATYVNDTFFTTAEQRAEAFENVLPQAEALER
AFRKGKAFFGGDAVGIVDLALGSFVVWVRVVDELDEARVPGLAAWAERFMAVDAVEEVMP
EAGKIMEHYK
>gi|BAJ90004|HvGSTU19
EPKLLGTFASPFVMRAKLALSFKGLSFEDVEEDLANKSELLLESNPAQKKVPVLIHNGVC
DSQVIVQYIDEAFSGNSLLPYERAVARFWAAYVDDKLLKTEEEKAEGLKQTLVAVETMEA
AFKNGKPFFGGDRVGYLDVTLGGLVVWVHAGAALDDAKSPLLAAWVERFGALDAAKAVLP
AVDRVVELAK
>gi|BAJ95534|HvGSTU20
DVKLLGMWASPYVLRVRLALSIKGIRYEYAEEDLRHKSELLLRSNPVHNKVPVLIHGGVC
ESLVILQYIDEAFGGAALLPHERAVARFWAAFIEDTLVKTEGEKVEGNKQATAALRTLEG
ALRMGKPFFGGDSAGYVDIVLGGLLAGVRAMEAMDPETMPLLAAWADNFGALDAVAAVMP
DVGRLVELFV
>gi|BAJ85757|HvGSTU21
EVKLLGMWASPFVVRAQLALRLKGVNYEYVEEELANKSDLFLRSNPVHKTVPVLIHDGIC
ESQVILQYIDEAFAGVSLLPYERAVARFWAAYVEDKLLTTDEERAEWTRQTVAAVDTLEE
GLREGKGFFGGDCVGYVDVLLGSIVPWVRATEKLDAGKAPLLAAWMERFSELDAAKAVFQ
DVDRVVEYAG
>gi|BAJ94042|HvGSTU22
DLKLLGAWASPFVTRVKLALSLKGLSFEDVEEDLGNKSELLLSSNPVHKKVPVLVHNGVC
ESVIILQYIDEAFAGISLLPYQRAVARFWAAYIDDKLVKTEEEKSEGINQTFAAVEMLEG
ALRKGEGYFGGETVGLVDVSLGSLLSWLNATEVMDPVKTPLLAAWMDRFSELDAAKAALP
EVDRVVEFAM
>gi|BAJ93446|HvGSTU23
PVRLVTAFGSPFAHRVEVALTLKGVYELLVEDLASKSDLLLAHNPVYQSVPVLLHGDVCD
SLVIVEYVDEAFHDDDGRRLLPYDRATARFWADFVANKCLKPDGEEQARLARETKEGLGV
LEAQLDGKRFFGGEALGFVDLAACTLAHWLGVLGEVEDGEYPALRRWAKEYTSHEVVRRS
LPDRDELVAYFT
>gi|BAJ94320|HvGSTU24
DLKLLGLSVSPFVVRVRMALHMKGLSYEYIEQDLFNKSELLLKSNPVEKKVPVLIHDGIL
DSSAIVQYIDEVWAAMPSILPYDRAASRFWAAYADDKLFTTKVERAEKVSETVAVLERLE
EAFANGKAFFAGDSVGYLDLAVGCHLHWLKAQRKMEAGRTPLLAGWAKRFAETDAAKEVV
PDTDVVMEYAK
>gi|BAK07331|HvGSTU25
PVKLISSFGSTFSHRAEVALRLKRVYELVVEDLRNKSQLLLTHNPVHKKVPVLLHGDRIS
ESLVIIEYVDEAFARPLLPHARATARFWAHFIDEKCMKSEPSEAQRRSVAEAKGSLALLE
EHLGRSFFGGDSVGLVDIAASLAHWLGVMEQITDAEFPTLCHWARRYASDEDVKSCLPER
GELIAMFS
>gi|BAK01145|HvGSTU26
DVKLLGSVMSPFAVRVRMALHAKGVSYEYLEQDLFHKGDLLLASNPVHNKVPVLIHAGVC
ESLAIVEYVNEVWADGASLLPYGRAVARFWAAYVDDKLFATEEDRAEKLDAALAVIGLME
DALADGKDFFAGDSVGYLDLALGCNLFWFKALREMDAGRTPGLAAWAERFEQTEAGKEVA
PPMKSMVEHAR
>gi|BAK01646|HvGSTU27
PVRLIGAFGSPVVHRAELALRLKGVYELILEDLNNKSELLLTHNPVHKTVPVLLHGHIPE
SLVIVEYVDEAFPAPPLLPLARANARFWARFLEEKFKKPDGKAQAASAKETKANLTLLEA
QLPGKRFFGGDAIGFLDIAVGGLAHWMGVVEEMTEEDHPTLCRWASAYRTNETVRQCLPD
RDRVLAALA
>gi|BAJ93711|HvGSTU28
RVKVLSTFGSIYGHRPEVALRLKGIYELLLGDLPNNKSELLLTHNPVHKLVPVLLHGDIC

ESLIIVEYIDEAFGGPPLLPCERATARFWAQFIDQKFARPGDKEEEEDFLKEAKENLLLL
ERQLKKKFFGGDSIGLVDIAASGLARWLETFEEITDEGFPALCRWAKEYADEERAKECLP
SKDELVAKFT
>gi|BAJ97078|HvGSTU29
AVRVIGAFDSPFSHRAEVALRLKGVYELILEELHNKSELLLTSNPVHKKVPVLLHGDVCE
SLIIVEYVDEAFDGPALLPYDRATARFWSRFVDDKCSTPDGEAQKGFIKEIKENFAILEA
QLEKRFFGGDTIGLVDIAACGFAHWLTVCEEVTADEFPRLCRWAKEYASDEKVRACLPDR
AQMLAHFT
>gi|BAK02670|HvGSTU30
GVKVFGMWASPMVIRVEWALRLKGVYEYVDEDLASKSDALLRRNPVTKKVPVLVHDGIVE
STIIVEYIDEAWKDGYPIMPHDRAQARFWARFADDKCNAATGKAQREVVREAQRCLKTLE
TALEKKFFGGDAIGYLDVVVGWYAYWLPVIEEVTDEELPLMKAWFDRFLAVDVVKDTLPP
RDKLLALNM
>gi|HvGSTU31|Predicted Protein
SVKLIGTLGSPFVHRAEAALRLKGVYELVLEDLQSKSDLLLKHNPIHQKVPVLVHGDICE
SLLIMEYVDEAFDGPPLLPYDRAMARFWAQFLEQKCSRPDAGEEQKGFVKETKENLALLE
GQLQKRFFGGDSIGYLDVAACGPAHWIYAFEEVGDENEFPALRRWGKEY
>gi|HvGSTU32|Predicted Protein
AVRVLGRWPSPFVIRVLIALGLKGVHELVEEAMGNKSELLLASNPVHKMIPVLLHHGVSE
SLIIVQYVHEAWSSHPALLPYARAAERFWAQYVDDKFPTARLGGDKDEAAVQVRAALQRL
EVALVGGKDYFGGDGVGYLDIALGSHLGWIRAVERIDEAKVPKLAAWADRFCAHPAVAGA
MPGVERFVEYSV
>gi|HvGSTU33|Predicted Protein
GVKLLGTVVSPFAVRVRMALHLKGVSYEDLEQDLFDKGELLLASNPVRKKVPVLIHAGVC
ESLAIVEYVDEVWAGAASLLPYDRAVARFWAAYVDDKVVTTEEERTERLDAALAAVRPLE
DAFDGGKAFFAGDSVGYLDLALGCHLFWFEALREMDAGRTPRLAAWAGRFLETETAKKAA
SPIESIVEYAG
>gi|HvGSTU34|Predicted Protein
ELKLLGVWTSPFVIRVRVVLNLKSLPYEYVEENLGSKSALLLGSNPVHQSVPVLLHGGVN
ESQVIVQYIDEVWAGVPSVLPYERAVARFWAAYVDDKVGSAWTCKTEEERAEAVSRAVAA
LETLEGALAGGKPFFGGDAIGFVDVVLGGYLGWFGAIDKIDPARTPLLARWEEWFRAADA
AKGVVPDADKMLDFLP
>gi|BAJ86777|HvGSTU35
DLKLLGLMVSPFVTRVRLALHMKGVGYEYIETDVLDKGELLLRYNPVHKKVPVLIHNGLC
ESQVIVQYVDEVWSAGAPILPYARATARFWAAYVDDKLFPTEAARAEKVDATLAALAQLE
VAAADGGRRPLFGGDSIGFLDLAVGCNMFWMEALRRMDAGRTPLLAAWAGRFAATEAATA
VVPDPDAAVAFAR
>gi|BAK03024|HvGSTU36
ELTLVGYWSSPFALRARYALNLKGLPYAYVEEVGLFDSKSPLLLASNPVHKRVPVLIHNG
VPESQLIVQYVDEAFPASRFLPHERAVARFWASYVDGELLRTGEERAQAAGRVGAALETL
ERAFGKGKGFFGGDTVGLVDVVLGGFVGWLKTSEAMDAAATPLLAAWAERFRALHGVKER
LLEYNL
>gi|CAJW010066194|HvGSTU37_Predicted CDS
EVKLLGTWASPFVLRAQLALSFKGVSFENVDEDLGSKSDLLLRSNPVHKAVPVLIHNGVC
ESLVIVQYVDEAFGGALLPHGRAVARFWAAFIEDKLVKTGEEKAEWMEQTLAAVDVLEGG
LKKGGSFFFGGDNVGYVDVVLGGAVPWVHATEALDGGRVPLLAVWLERFGALDKAQAVML
DAQRLVQHAK
>gi|CAJW010135660|HvGSTU38_Predicted Protein
ELKLLGSWSSPFVTRAKLALAIKGLSYENIDEDVHNKSDLLLTSNPVHKKIPVLIHNGVC
ESMIIVQYIDEAFAGTPILPHERAVARFWAAYVDDKLVKTGEDKAAEAMKEMLLAVQTLE
GALREGKGFFGGDSVGLVDIALGSMHSWLMAIEAMDPARTPLLAAWMERFGALDVARAVL
PDVGRVVELAK
>gi|CAJW010309720|HvGSTU39_Predicted Protein
ELKLLGFWASPFVHRARVALHLKGIGYEYAEEDLTSKSDLLLASNPVHAKVPVLLHGGVC
ESMLIVQYLDEAFPGSPALLPHDRAVARFWAAYADDHFFTTDEERAAATEAAAAALQKME
GAFGKGKAFFGGDAPGYVDIALGGYVAWMRAFHAVDAARTPLLAAWAERFAALDAAKEVI
PDVDRIAEFAK
>gi|CAJW010039737|HvGSTU40_Predicted Protein
GLVLLDFWRCRIALAEKGLYESLEQELLGDKSDLLLGSNPVHKKVPVLLHDGVCESLVIL
EYIEDAFPGAAPQLLPYERAQARFWADYVDKRVYSCLKGEGRAAARAEMLETLRTLDAEL
GDKAFFGGALGFVDVTLIPLTSWFYSYEKQEEECPRLAEWARRCGERESVAKVLTPEEVH

DFIG
>gi|CAJW010045730|HvGSTU41_Predicted Protein
PVKVIGTRFSAFSHRAEVALRLKGVYELIAEDLDNKSELLLRSNPVHGKVPVLLHGDRVC
ESLVIVEYVDEAFDPPILPAGRAAARFWAGFLGVNHCWRLADGDARAGFREEARARLALL
EAQLEGKRFFAGDGIGYVDVAASGLAYWLGAMEEVDAADFPALCRWAKEYTSSDVVKGCL
PDWDELVAGYA
>gi|CAJW011586888|HvGSTU42_Predicted Protein
EVKLLGVWDSPFVNRVQIVLNLKGISYEYVEEDLHNKGELLLASNPVHKKVPVLIHNGIP
ESQVIVQYIDEAWRGTPSLLPLERATARFWAAYVDDKVSITRRIAIPTSRSLLGSCVNVI
KLEGMDVRKTEEKMEAAVRAISAMETLEGAFEGGKPFFGGDGIGFVDVVLGSYLGWFVVI
EKMDSARTPALAAWAEHFRMADAAKGVLPEVDKVLEFLQ
>gi|CAJW010159558|HvGSTU43_Predicted Protein
ELKLLGTWRGPFALRVRLALNFKGLSYEYQEEDLANKSKLLLESNPVNKKVPVLIHNGIC
ESLAILEYVDEVYRGICLLPYQRARARFWAAYIDNKLVKTNKEKDEGRKQTLAAVEMLEG
ALGKGKTFFGGDNVGYIDVVLGGMVAWMLGTKALHATKTPLLLGWMESFGGLEPAKVVLP
EVDRLVEFAK
>gi|CAJW011559020|HvGSTU44_Predicted Protein
ELKLLGTWASAWAARVKIALHLKGLSYEYVEQDLDNKSDLLLTSNPVHKKVPVLIHNGIC
ESVVILEYIDEAYGTTSLLPYERAVARFWVDYIDQKLVDEEEEKTEGINQMLAAAHTLEG
ALKKGKPFFGGDSVGHVDIALGGLLAFLQGTEDLDKANTPLLLAWMDRFAELDAAKVALP
DVGKLVEFAK
>gi|CAJW012547630|HvGSTU45_Predicted Protein
EVTCVDFWCNEFGMRVRIALRELGVFEYIEEDLRVRERSDLVWRMNPVHRSIPILIHHGV
CGSVNIVEYIDDVWGQVTRRLLPADRAGARFWADFIDHKVFSTSKGEEKEAAKEELVEHL
KRLDRVLGDKCFFSGEFGFLDAVFIPFSSMFYGYHQHELECPKLTRWEKRCRERES
>gi|CAJW010115070|HvGSTU46_Predicted Protein
DVVLLDFWASPFGQRCRIALAEKGVYEYCEQDLEQKSELLLRSNPVHKKIPVLLHDGVCE
SLIILSYIDEAWPEVAPLLPYARAQAQFWADYIDNKIVDCTKGDAQEQAKKDMIGALETL
EAELGDKDYFGDAFGFVDVAFVTLTPWFYTYEKYEEHCPRIMAWAARCRERESVAKALTA
EKVYEIV
>gi|CAJW010138594|HvGSTU47_Predicted Protein
PAVRLIGWLSPYVHRAEVALRLKGVYELLNDDMASKSELLLTHNPVHNKVPVLLHGDIPE
SLVIVEYVDEAFPAPPLLPLARANARFWARFLDDKCWKAEPGEAQAAAAREAKEGLALLE
AQLPGRRFFGGDAIGLLDIAASGVARWLGVFEEMTEVEHPALCRWAREYAADETVRQCLP
DRDLVLAVLT
>gi|BAJ97078|HvGSTU48
PVRLLGSFGSPFTHRAEAALRLKGVYEFIQEDLHNKSELLLQHNPVHKKVPLLLHGADVA
ESLVIVEYVDEAFEPLLLPLARAAARFWAQFADDRCSRTPEGEARRGFVEEAKENLALME
AQLEGRRFFGGDSIGLLDIAASGLAWLPVLEEVMLREEDYPALCRWRGDYASDEAVKKCL
PSRDEMVAYYA
>gi|CAJW011584498|HvGSTU49_Predicted Protein
PVKLIGAFGSPFVHRAEVALRLKGVFELILEDLNNKSELLLKHNPIHKTVPVLLHGDRVL
ESLLIVEYVDEAFDPPLLTYGRAMARFWAQFIEQKCSRPDDGEAREGFVRETKANLALLE
ARLQGKRFFAGDAVGYLDLAACGLSHLLGVIEDVDAGEFPALRRWAEDYVSDATVSACLP
PREQLAAHFA
>gi|CAJW010050304|HvGSTU50_Predicted Protein
QLKLFGSWASSYTHRVQLAMRLKGLFEYAEEDLRNKSDALLRHNPVYNKVPVLVHDGLAE
SVIILQYLDDAFPARQLLAFDRAVARFWCHFGDDKVCTRAHAPLVMILLWLTCFLWNGST
GEEQEAAVHQVHENLALLEAELRKGRRFFGGDEVGFLDVVLGCGSYWLAVFEEV
>gi|BAK01391|HvGSTF1
PVKVFGPAMSTNVARVLVCLEEVGAYEVVDIDFKAMEHKSPEHLVNPFQIPAFQDGDLLL
FESRAIAKYVLRKYKTDEDLLLKEAAMVDVWTEVDAHTYNPASPIVYECLINPLGLPTQT
VVDESLEKLKKVLEVYEARLSQHKYLAGDFVSFADLNHFPYTFYFMATPHAALFDSYPHV
KAWWESLMARPAIKKLAAQMVPKKP
>gi|BAK00326|HvGSTF2
PPNSLRRTGTSDHNFHIESRAIARHVLRKYKPELLVPESAAAVDVWLEVEAHQHHPVVAI
TMQCLVAPLGNATQDVLDENLGKLRKVLEEYEARLSASKYLAGQSVSLADLSHFPMMRYF
METEFAALVEEHPHVKAWWEELKARPAARRVTEFMPPDFGLGK
>gi|BAK01090|HvGSTF3
GLQVFGQPASTDVARVLTCLFEKNLFELVRIDTFKREHKLPEFIKRDPTQVTFKHGDKTL
VDSRAICRYLCTQFPEDGNGIYSLERASIEQWLQAESQSFDAPSELVFHLAFAPQLNMIE

ARIAENERKLQQMLGVYDEILAKNQYLAGDEFTLADLSHLPASHYIAGSQRGRKLFTSKK
HVARWYDAISSRPSWKQVVKMQHEHPGTFE
>gi|BAK01441|HvGSTF4
AVKVYGVGASPFVATVLLCLEEMGAYELVPLDMAAREQRTEPYLSNPFKIPALEDGELTL
FESHAISRYVLRKYGGTSATDLLKELAMVDLWTEVEAHQYQPAVNVVRQCVILPFGGTRQ
AVVDEYVGKLEKVLDVYEARLSSSPYLAGDFFSLADLVHFGFTYCLVAGTEYASLLESRA
SVMAWWGRIMARPAVKKVAPLIHLGLKLSS
>gi|BAK06926|HvGSTF5
PVKLYGMTLSWNVTRCVAALEEAGVYDVVPIDFGAGEHKTPEHLTNPFQMPVLQDGDFYV
WESRAICKYTCRKNKELLLNESAMVDVWLEVEAHQYTAAEPIILECLVRPMGRATQKTVE
ENLVKFKKVLEVYEAHLTRCKYLAGDFLSLADLNHVSATACLAATPYASLFDAYPHVKAW
WSGLMARPSVQKIAALKKPYFHNSV
>gi|BAK01892|HvGSTF6
PMKVYGWAVSPWMARVLVCLEEAGAYEIVPMSRSGGDHRQPDHLTNPFEIPVLEDGDLTL
YQSRAIARYILRKYKPELLRLAESAMVDVWLDVEALQHEPIRPIVANCIVYPFGRSRQEI
VDEKIERLKKLLEVYESRLSDSKYLAGDFISLADLSHFSLMRYFMATEYADLLDAYPHVK
AWWAALLARPSVKKVMAGMPPDFGFGS
>gi|Q8LPD5|HvGSTF7
PVKLYGATLSWNVTRCVAALEEAGVYELVPINFSTGEHKSPDHLANPFQVPALQDGDLYV
FESRAICKYACRKNKELLIKESAMVDVWLEVEAHQYTAASPILFECLIHPMGGATQKVID
DNLVKIKNVLAVYEARLTKSKYLAGDFLSLADLNHASVTLCLAATLYASLFDAYPHVKAW
WTDLLTRPSVQKVAALMKP
>gi|BAJ87824|HvGSTF8
PVKVFGPAGSTNVARVLVCLEEVGAYELVDIDFPGKGHKRPDHLANPFQVPALQDGDLVL
FESRAIAKYVLRKYKSEQDLLLEEAAMVDIWTEVETHQYHPASPIVLECFIYPTGLLTQR
IVDESLEKAKKVLEIYEAHLSKHKYLAGDFVSFADLNHFACTFYLMDATPYASLFDSYPH
VKAWWEDLMSRPSMKKLGASMTTRV
>gi|AK369857|HvGSTF9
ALKVYGWAVSPFVARALLCLEEAGVYELVPMKREAGDHLLPDFLANPFQVPVLEDGDLTI
FESRAIARHVLRKYKPELLAPEAAAMVDVWLEVEAHQHHPVGAISIQCLLVPFGGTRQAV
VDENVVKLRKVLEVYEARLSASKYLAGESVSLADLSHFPLMHYFMQTEYAAMVEERPHVK
AWWEELKARPAARKVTEFMSPDFGLGK
>gi|BAJ85232|HvGSTF10
PVTVYGPAISPAVARVAACLLEKDVFQLEAVDMSKGEHKSPSFLKQPFQVPAFKDHLTTV
FESRAICRYICDQYSDRGNTLLAVGRAAIEQWIESEGQAFNPPLAIAFQLTFAPLMGMAM
AVVEQNEEKLAKVLDVYEGRLAESQYFAGDEFTLADLVHMPNTDLLVSKTGKAGLITERK
NLSRWWDEVSARPSWKKVVELQSAPRPPRS
>gi|AK367210|HvGSTF11
AVKVYGWAVSPFVARPLLCLEEAGVYELVPMSREAGDHRQPDFLANPFQVPVLEDGDLTL
FESRAIARHVLRKHKPELLPEAEATVDVWLEVEAHQYNPAGAIVVQCIILPLGGARQAVV
DENVGKLKKVLEVYEAQLSTSRYLAGDDISLADLSHFPFMRYFMETEYASLVEELPHVNA
WWEGLRARPAARKVTELMPPDLGLGK
>gi|BAJ91428|HvGSTF12
SRKLYGYALSSNSVRIAALLNEKGLYELVVVDDTKAPEFLANPLQVPAFQDGDDILFESR
AISRYIATKYRSSGTDLLASAKLEVWLEVESHHFYPAADLVYELRVRPRLGVPPPAVVDG
LAGKVADVLDVYDAHLAAGNRYLAGEQFTLADVNHIAQLFVMSRTPRAAELVAARPHVQA
WWDEISARPAWKKTVAALPLPPA
>gi|BAJ90215|HvGSTF13
PVKVFGHPMLTNVARVMLFLEEVGAYELVPVDFVAGEHKRPQHLQNPFQMPGFQDGDLVL
FESRAIGKYIIRKYGTAGDLLIEESAMVDMWTEVEDQQYYPASPVVFECIINPFIGAATQ
KVVDKSLERLKGVLGIYEARLEKSRYLAGDSISFADLNHIPFTFYFMTTRHASVFDDYPK
VKAWWGSLMARPAVQRVCKHMPSLFSCLL
>gi|BAJ90031|HvGSTF14
PMKLYGMMLSPNVTRVATVLNELGLFEFVSVDLRTGAHKQPDFLKNPFQIPALQDGDEVV
FESRAINRYIATKYGAAELLSAKLEVWLEVESHHFYPPRALVYELLIKPMLAPTAAEVNK
NAADLDKLLDVYEAHLAAGNKYLAGDAFTLADANHMSYLFMLTKSPKADLVASRPHVKAW
WDEISARPAWAKTVASIPLPPGV
>gi|BAK02558|HvGSTF15
AVKVYGWAMSPFVARALLCLEEAGVYELVPMSREAGDHRQPDFLANPFQVPVLEDGDLTI
FESRAVARHVLRKHKPELLPESAAMVDVWLEVEAHQHQTPGTIVMQCILTPFGCERQAAV
DENVGKLTKLFDVYEARLSASRYLAGDSLSLADLSHFPLMRYFMDTEHASLVDERPHVKA

WWEELKARPAAKRVTEFMPPNFGFGK
>gi|HvGSTF16|Predicted Protein
KVKVFGPARSTCVARVLVCLEEVGAYELVHVHLPAGDHKGPAHLATPFQVPAFQDGDLIL
FESRAISRYVLRKGADLLLGESAAVDAWLEAESHNFDRASAITFQCFVVPMGGTAQMVVG
ENLEKLKVALRVYEERLSRYRYLAGDFISLADLSHCPMAHYLLASPCASVLDAYPCVKAW
VDGIMDRPSVKKVMELMDAS
>gi|BAJ93656|HvGSTF17
GLQVFGQPASTDVARVLTCLFEKNLFDLVRIDTFKREHRLPEFIRRDPSQVTFKHGDKTL
VDPRAICRYLCTQFPNQGNNLYSLERASIEQWLQAEAQNFNPPSALVFQLAFAPVLEIPH
MVIAENERKLQQVLNVYDEILSKNEYLAGDEFTLADLCHLPDSQYIVSSERGMKLFTSRK
NVARWFDQISSRKTWEQVVKMQMEHPGAFE
>gi|HvGSTF18|Predicted Protein
PMKLYGWAVSPWMARVLVCLEESGAYELVPMSRNGGDHRRPEHLANPFEIPVLEDGDLTL
YQSRAIARHVLRKHKPELLGLEESAMVDVWVDVDAHQLEPVQPIVWNCIINPFGRDVQGL
VNESVKKLKKVLEVYEARLSSSKYLAGDFVSFADLTHFSFMRYFMATEHAIVLDAYPHVK
AWWEALLARSSVKKVMAGMPPDFGFGS
>gi|CAJW010214035|HvGSTF19_Predicted Protein
PVKVFGSAAFTNVARVLVCLEEVGAYEVVDIDFAAKEHKNPEHLANPFEIPAFQDGDLVL
FESRAIAKYILRKYRTDEDMLPEEAATVDVWAEVEAHQYSKAAPIVYECIVYPTGVKTQK
VVDESMEKLKKVLEIYEGRLSKHEYLAGSFISVADLNHFPFTLRFMETPYASLFDSFPCV
KAWWEKLMLRPSMKKLGADMQKRV
>gi|CAJW011565989|HvGSTF20_Predicted Protein
AVKVFGSAAFTNVTRVLVCLEEVGAYEIVQVDFHAREHKGSGHLAQPFQVPAFQDGDLVL
FQSRAISRYVLRKHKTDENLLPEESALVDVWMDVEALNFDTAHAVFYQHRVAPAGRTAEK
IIGDNIEKLKKVLDVYEARLTKHRYLAGDFLSLADLSHFPETHYFMGMPYAAVLDAYPRV
RAWLKELFARPAVKKVISLMAKDFQ
>gi|HvGSTF21|Predicted Protein
AVKVFGSPTSSEVARVLTCLFEKDVFQLIRVDSFRGPNRMPQYLKQPHEALTFEDGNVTL
VESRKILRHIADKYKRQGNDLIALERSSIEQWLQTEAQSFDVPADMIYSLAYLPRTMPVE
EMKQLFEKSSRDLSKVLDIYDQRLEEAEYLAGDKFTLADLSHMPNADRLASDERTARLIQ
SRRNVSRWWTAVSSRGSWVYVKSLQRPSSAEAP
>gi|BAJ97007|HvDHAR1
PDRLGDCPFTQRVLLTIEEKNLYELKLVDLANKPDWLFTINPEKVPIVKLVADSDVITQV
LEEKYPQPSLAPPEKASIGSKIFSTFIGFLKSKDTNDGTEQTLLSELTSFDSYLKDNGPF
INGGTISAADLSLAPKLYHMEIALGHYKNWSVPDALAHVKTYMKTIFSMDSFVNTRGLPE
>gi|BAJ90632|HvDHAR2
PDTLGDCPFSQRVLLTLEEKKVYEMKLIDVSNKPDWFLKINPEKVPVFNGGIADSDVITQ
VIEEKYPTPSLVPPEYASVGSKIFSTFVTFLKSKDASDGSEKALVDELQALEEHLKAHGP
YINGANVSAADLSLAPKLFHLQVALEHFKGWKVPETLTSVHAYTEALFSRESFVKTKPTK
E
>gi|HvEF1G1|Predicted Protein
SHKIFSYPNNQRVWKAQIAGNFTGLIEAPAFQIGVDNKTPEFAKFPLKVPALETPEGPLF
ESNAIARYAARYGGSSKIYEASLIDQWIDFSANEIELPASAWLYPIFEIVPFNAEATERA
KADIKKALELLNKHLTYRTYLVGERVSLADIVVSMALYRLYTMVLDITFRKNYGNVNRWF
LTCVNQPQFRTVLGETKLTEKMAV
>gi|HvEF1G2|Predicted Protein
ALVLHSGSGNKNAFKALIAAEYCGVVELPKNFEMGVSNKTPEFIMNPLKVPVLETPDGAV
FESNAIARYVARSKGDNLLWEYARVEQWMDFAATEVDPNIARWLYPRLGYAPFNAQSEEF
GIAGLKRALEALNTHLASNTFLVGHSVTLADIVMTCNLYHGFARIL
>gi|BAJ86721|HvGSTL1
TTRLYVAYHCPYAQRAWIARNYKGLDKIKIVAVDLADRPAWYKEVYPQKVPSLEHKGESL
DLVKYIDSNFDGPALLSAKKQFSEELLVYTDEFNKALYSSITSKGDVAEETVAALDKIEA
ALGKFSDGPFFLGQFSLVDIAYVPFIERFQIFFSGIKNYDITKDRPNIQKFIEEVNKIDA
YTQTKLDP
>gi|HvGSTL2|Predicted Protein
TTRLYLAYHCPYAQRAWITRNCKGLDKIKVVAIDLADRPAWFKEVYPDKVPVLEHKGESL
DLVKYIDSNFDGPALLSAKKQFAEELLAFSDGFNSAFFSCLRSKGDVSAEAAAAVDKIEA
ALGKFSDGPFFLGQFSLVDIAYVPFIERFQIFYSGIKKDDIAKGRPNLQKYIEEVNKVDA
YTQTKLDP
>gi|BAJ98512|TCHQD1
MQLYHHPYSLDSQKVRIALEEKGIYTSYHVNPLTGKNMNVAFFMNPSKLPVFQNGAHVIF

RAIDIIQYIDRLAVHLQPVNTEVLQWMQKVDGWNPKMFTLTHTPLKYRSFVSKFIRRVLI
ARMAEAPDLASMYHVKLRDAYETEDRLKDPETMRQSEEELSKLLDDVEAQLNKTKYLAGD
EFSPADSMFIPILARIALLDLHEEYISSRPRVLEYHTLVKQRPSYKVVIGKYFNGWKKYR
>gi|HvGSTT1|Predicted Protein
LLKVYADRLSQPSRAVIILCKANRIFEEVKVDLAKGQHRRQEFNKINPMQVPAIIDGRFK
LFESHAILRYLATVFPGVPDWYLFTRAKLESVLDWHHSNLRRGAASYVLHTALGPALGLT
PNPETAKEGEKLLSKSLGTIETVWLKGDAKFLNGNPQPSIADLSLVCEIMQLEVVGDERR
DTILGPHEKVRAWMENVKKNPHFDEVHQLIFKLKARLS
>gi|HvGSTZ1|Predicted Protein
KPILYGAWISSCSHRVRIALNLKGVYEYKAVNPRTDPDYEKINPIYIPALVDGDFVLSDS
LAIILYLEDKYPQHPLMIKMKALDLQIANIVCSSIQPLGYGVIGLHEGRLSSDESLEVVQ
RYIDKGFRAIEKLLDGCDSKYCVGDEVHLGDVFLAPQIHAAINRFQIDMTKYPILSRLHD
VYMEIPAFQAALPQNQQDAPSA
>gi|BAK05758|HvGSTZ2
KPVLYSAAISSCSYRVRIALTLKGVYEYRAVAWNDPDYEKINPIYVPALQDGDILVSDSL
AIILYLEDKYPLHPLLLKRKALNLQIANIVCSSIQPLCYAVVGLVNGKLGSDESLQIVHH
YIDKGFRAIEKLLEGCNSKFATGDEVQLADVFLAPQIHAGVTRFNIDMSKYPHLERFYKA
YMEIPAFQAARPENQPDAPSLL
>gi|HvGSTZ3|Predicted Protein
RPILYSYWRSSCSHRVRIALNLKGIYEYKAVNLLKGEQSDPEFMKLNPMFVPAWVNGDAV
IGDSYAIALYLEDKYPQRPLLLEKKALNIQIASIVCSGIQPLNLTLVRFIEQKVGTGESL
PWVQQQIDRGFTAVENMIKGCAGKFAMGDEVQLADVFLAPQIFAAVTRFQIDMSKYPTLA
RLHDQYMTHPAFEAALPDRQPDAPSSA
>gi|HvGSTZ4|Predicted Protein
KPVLYSAAISSCSYRVRIALTLKGVYEYRAVAWNDPDYEKINPIYVPALQDGDILVSDSL
AIILYLEDKYPLHPLLLKRKALNLQIANIVCSSIQPLCYAVVGLVNGKLGSDESLQIVHH
YIDKGFRAIEKLLEGCNSKFATGDEVQLADVFLAPQIHAGVTRFNIDMSKYPHLERFYKA
YMEIPAFQAARPENQPDAPSLL
>gi|HvGSTZ5|Predicted Protein
KPILYSKWFSSCSHRVRIALNLKGVFEYRATNPMTDPDYEKINPVYVPALVDGDFVVSDS
FAIIKYMEDKYPQCPLLLKKKALNLQIASIVCSSIQPLSYAVIVRTDIIFKFSQARIKHF
QLLCGNIHIRLRSFHGTMDANESLEMVEHYIDKGFRAIEKLLEGCDSKYATGDEVQMGDV
FLAPQIHAGVTRFQIDMSKYPILARLQDAYNEHPAFQAALPANQPDAPPSQ
>PtGSTU1
AVTLLDTRVSPFGMRVRIALEEKGVYEYSEQDLRNKGPMLLQMNPVYKKVPVLIHNGICE
SLIAVQYIDEVWNNKPLLPYQRAQSRLWADFVDNKVYFPKKGQELKNDLIESLKLLEGEL
GDKAYFEGKLGYVDIALIPFYSWFHGYETFEAECPKLIAWCKRCLQNESVSRSVAPQEVY
GFLV
>Pt-GSTU2
QVTLLDFWSSPFGMRVRIALAEKGVYEYSEQDLRNKSDLLLQMNPVHKKIPVLVHDGVCE
SLIIVQYIDEVWKDKALLPHERAQSRFWADFVDKKMYDFTKGEDQEAAKKDFIDSLKLLE
GELGDKPYFGGTLGFVDVALLPFYCWFYAYETIEADCPKLIAYCKRCLQKESVSKSLKPQ
KVYDFVV
>Pt-GSTU3
QVTLLDLWASPFGMRVRIALAEKAVYEYSEQDLWNKGALLLQMNPVHKRIPVLVHDGIFE
SLIIVQYIDEVWKDKALLPYERAQSRFWADFVDKKLYELTKGEDQEAAKKDFIDSLKLLE
GELGDKPYFGGTLGFVDVALLPFYCWFYAYETIEADCPKLIAYCKRCLEKESVSKSLEPQ
KVYDFVV
>Pt-GSTU4
QVTLLDLWASPFGMRVRIALAEKAVYEYSEQDLWNKGALLLQMNPVHKRIPVLVHDGIFE
SLIIVQYIDEVWKDKALLPYERAQSRFWADFVDKKVYDLTKGEDQEAAKKDFIDSLKLLE
GELGDKPYFGGTLGYVDVALLPFYCWFYAYETVEAVCPKLIAYCKRCLEKESVSKSLEPQ
KVSDFVV
>Pt-GSTU5
QVTLLDFWSSPFGMRVRIALAEKGVYEYSEQDLWNKGALYLQMNPVHKKIPVLVHDGICE
SLIIVQYIDEVWKDKALLPYERAQSRFWADFVDKKVNYFTKGEDQEAAKKDFIDSLKLLE
GELGDKPCFGGTLGYVDVALLPFYCWFYAYETIEADCPKLIAYCKRCLEKESVSKSLKPQ
KVYDFVV
>Pt-GSTU6
QVTLLDLWASPFGMRVRIALAEKAVYEYSEQDLWNKGDLLLQMNPVHKKIPVLVHDGICE
SLIIVQYIDEVWNDKALLPYERAQSRFWADFVDKKIHDVPKGEDQEAAKKDFIDSLKLLE

GELGDKPYFGGTLGYVDVALLPFYCWFYAYETIEADCPKLIAYCKRCLEKESVSKSVKPQ
KVYDFVV
>Pt-GSTU7
QVTLLNFWASPFGMRVRIALAEKGVYEYGEQDLRNKTALLLQMNPVHSKILVLVHNGACE
SLIIVQYINEAWKDKALLPYQGALSRFWADFVDKKIYDLTKREDQESAKKDFIDSLKLLE
GELGDKPYFGGTLGYVDVALLPFYCWFYAYETIEADCPKLIAYCKRCLQKESVSKSLEPQ
KVSDFVV
>Pt-GSTU8
QVTLLDFWSSPFGMRVRIALAEKGVYEYSEQDLRNKSDLLLQMNPVHKKIPVLVHDGVCE
SLIIVQYIDEVWKDKALLPHKRAQSRFWADFVDKKIYDFTNGEDQEAAKKDFIDSLKLLE
GELGDKPYFGGTLGYVDVALLPFYCWFYAYETIEADCPKLIACCKRCLQKESVSKSLKPQ
ILYDFAV
>Pt-GSTU9
QVTLLDFWASPFGMRVRIALAEKGVYEYSEQDLRNKSALLLQMNPVHKKIPVLVHDGICE
SLIIVQYIDDVWKDKALLPYQRAQSRFWADFVDKKLYDLTKGEDQEAAKKDFIDSLKLLE
GELGDKPYFGGTLGYVDVALLPFYCWFYAYETVEAVCPKLIAYCKRCLEKESVSKSLEPQ
KVSDFVV
>Pt-GSTU10
QVTLLDFWASPFGMRVRIALAEKGVYEYSEQDLRNKTALLLQMNPVHKKIPVLVHNGVCE
SLIIVQNIDEAWKDKALLPYQGVQSRFWADFVDKKIYALTKGEDQESAKKDFIDSLKLLE
RELGEKPYFGGTLGYVDVALLPFLCWFYAYETIEADCPKLIAYCKRCLQKESVSKSLEPQ
KVSDFVV
>Pt-GSTU11
RVKLFGALLSPFVYRVIWALKLKAIFEFIEEDLPNKSPLLLKYNPVHKKIPVLLHGDVCE
SMIIVEYIDEMWPQNPLLPYERALARFWVKFAEDKGTSKEEELEMTRKETLEMLQNVEEH
GLEKKFFGGDSVGMADIAFGSVVYWLEVVEEVEAHKFPRLHAWMKNFKQAPIIKENLPDR
DWLVTFFK
>Pt-GSTU12
RVKLLGALPSPFVYRVIWALKLKAIYEFIEEDLTNKSPLLLKYNPVHKKIPVLLHGDVCE
SMIIVEYIDEMWPQNPLLPYERALARFWVKFAEDKGTSKGEELENTRKETLEMLHNVEEH
ALEKKFFGGDSIGIADIAFGSVIYWLGLIEEVEAHKFPRLHAWMKNFKQAPIIKENLPDR
GWSVTFFK
>Pt-GSTU13
RVKLLGALPSPFVYRVIWALKLKGIYEFIEEDLTNKSPLLLKYNPVHKQIPVLLHGDVCE
SMIIVEYIDEMWPQNPLLPYERALARFWVKFAEDKGTSKGEELEKTRKETLEMLQNVEEH
GLEKKFFGGDSIGIVDIAFGSVIYWLELIEEVEAHKFPRLHAWIKNFKQAPIIKENLPDR
AWTVTFFK
>Pt-GSTU14
DVKLHSFWPSPYCYRVIWALKLKGIYEHVEEDLSNKSQALLQYNPIHKKVPVLVHGGIVE
SMVILEYIEETWPDHPLLAYERATARFWIQFGIDKGMATEEEKEKAAKEALEVLKILEEQ
CLDKKFFGGENIGMVDIAYGWLAHWFEASEEMEPSTLPRLHAWIQRLKEIPVIQENLPDR
ERLLVHFR
>Pt-GSTU15
RVTLLDFWSSSWAVRVKVALAEKGIYESREQNLIDKSSLLLEMNPVHKTIPVLIHNGICE
SHNIVQYIDEVWKDKSPLLPYQRSQARFWADYIDKKIYNNEKGEKQEEVKREFIEGLKTL
EGELGDKLYFGGSFGFVDVVLVPVTSWFYSLEICEAECPKITAWIKRCMEKESVSSSLPP
HKIYDFVL
>Pt-GSTU16
RVTLLDFWPSPWATRVKVALAEKGIYESREQNLIDKSPLLLEMNPVHKTIPVLIHNGICE
SHNIVQYIDEVWKDKSPLLPYQRSQARFWADYIDKKIYNNEKGEEQEEVKREFIEGLKTL
EGELGDKLYFGGSFGFVDVVLVPVTSWFYSLEICEAECPRFTAWIKRCMEKESVSSSLPP
HKVYDYVL
>Pt-GSTU17
RVTLLDFWSSSWAVRVKVALAEKGIYESREQNLIDKSSLLLEMNPVHKMIPVLIHNGICE
SLNIVQYIDEVWKDKSPLLPYQRSQARFWADYIDKKIYNNEKGEKQEEVKREFIEGLKTL
EGELGDKLYFGGSFGFVDVVLVPVTSWFYSLEICEAEFPMIAAWIKRCMEKESVSSSLPP
HKIYDFVL
>Pt-GSTU18
EVKVFRSWSSPFPLRVIWALRLKGVFDVICEDLFNKSPLLLQYNPVGKKVPVLVRNGICE
SLVILEYIEETWKQTPLLPYQKANARFWAKFSDDKVLQSEGKEQEEGILASLQNLRYLEE
ELRKKFFGGEAIGLADLALGWLAYYLNIFEEVDQESFPSLVAWMQDFANAPVVHGSWPDK

DKLAEKFV
>Pt-GSTU19
DVKLHGFWASPFSYRVIWALKLKGVFEYIEEDLANKSELLLKYNPVYKQIPVFVHGGIAE
SLVILEYIEETWPENPLLPCERAMARFWIQYGATKTAASGEELEKAAKEVVEVLRVLQEQ
GLDKKFFGGDSINLVDISYGLFVYWLAAIEDIEPSTLPRLHAWAQNFIEVPLIKENHPDN
DKLLLHMK
>Pt-GSTU20
DVKLHGSWVSPFNYRVIWALKLKGVFEHIVEDLTNKSELLLKYNPVYKKIPVLVHGGIAE
SLVILEYIEETWPENPLLPYERAMARFWIQYGATKTAASGEELEKAAKEVVEVLRVLEEQ
GLDKKFFGGDSINLVDISFGLFTCWLEAIEEAEPSTLPRLHAWAQNFIEVPLIKENIPDY
DKLLLHMK
>Pt-GSTU21
DVKLHGSWVSPFNYRVIWALKLKGVFEHIVEDLTNKSELLLKYNPVYKKIPVLVHGGIAE
SLVILEYIEETWPENPLLPYERAMARFWIQYGATKTAASGEELEKAAKEVVEVLRVLEEQ
GLDKKFFGGDSINLVDISFGLFTCWLEAIEEAEPSTLPRLHAWAQNFIEVPLIKENIPDY
DKLLLHMK
>Pt-GSTU22
DVKLIGAWPSPFVMRARIALNIKSLGYEFLEEKLGSKSQLLLESNPVHKKIPVLIHDGIC
ESLVIVEYIDEVWSSTILPYDRALARFWAAYLDEKWFKEEEARKALIEQAGEGVMMLEDA
FSKGKGFFGGDQIGYLDIAFGSFLGWLRTTEKMDETKTPSLLKWATSFSSHPAVKDVLPE
TEKLVEFAK
>Pt-GSTU23
QVKLLGFWASPFARRVAWALKLKGVYEYIEEEDIFNKSSQLIELNPVHKKVPVFVHGQIA
ESFVILQDCLKTWKQCPLMPHERAMIRFWTKFAEEKKILHRGEEKEKAAKLTVDVIEKIE
GELKKQFFGGESIGYLDIALGWMSYWLPVWEEVDPLQFPALTSWIKNFLEHPVIKDNLPP
RDRMIIYFL
>Pt-GSTU24
EVKVFRTWSSPFALRVIWALKLKGVFDTIYEDLSNKSPLLLQYNPIHKKVPVLVHNGICE
SLVILEYIDETWKQNPLLPHQQANARFWAKFGDDKVLQSEGKELEEGVLASLENLKYLEE
EIRKKFFGGETIGLADIALGWLAYYLDIFEEIDQEKFPSLAAWKQEFANAPIIHENWPDR
DKLVNKFV
>Pt-GSTU25
EVKLHGFWSSPFSCRVIWALKLKGVYEYIEEDLLNKSELLLKYNPIYKKIPVLVHDGIAE
SLVILEYIEETWPENPLLLYDRAMARFWIQYGATKCADASEEEKEKAAKEIMEVLKILED
QALDKKFFGGDNINLVDISYGPCAYWLPAMEEAEPSTLPKLHAWAKNFIEVPVIKENIPD
YDKMLAYMR
>Pt-GSTU26
EVKVIGSSKSLFCARVEWALKLKGVYEYLQEDIWNKSPLLLKHNPVHKKVPVLVHDDIAE
SLVILEYIDETWKDYPLLPYERAMARFWAKFAEEKCLMGEGEEKEKAIESALESFTFLEK
QIQKKFFSGDNIGYLDLVMGWIPLWLNVMEEADAQKFPFLHEWTQNFIEIPLIKECLPPR
DALVNYFI
>Pt-GSTU27
EVVLLDFEMSPFAARVRIALEEKGIYKSKVEDLSNKSSTLLKMNPVHQQIPVLIHNGICE
SMIIVQYIDEVWSHKPLLPYRRAHARFWADYIDKKIYPISEGEVKESSKKDLFQCFKILE
EELGDKQYFGDSFGYIDLALIPFYSFFYTFETLVAEFPKLVKWGERCLQKESVSKSLSQK
EIYEAAL
>Pt-GSTU28
EVVLLGFWASPFAMRVKIALAEREIYVSREQNLFNKSSLLLEMNPVYKKVPVLIHEGICE
SLIIIQYIDEVWKHKALFPCERAHARFWADYVDKHIFPNRKGERQEAAKKDLIESFKALE
GELGDKPYFGGSFGLLDIALIPFFNFFYAFETLEEECPEIVAWAKRCSQRETVSKSVVLQ
HKAYEFVL
>Pt-GSTU29
EVVLLGFWASPFAMRVKVALAEKEIYVSREQNLFNKSSLLLEMNPVYKKVPVLIHEGICE
SLIIIEYIDEVWKHKALFPCERAHARFWADYVDKHIFPNAKGERQEAAKKSLIESFKALE
GELGDKPYFGGSFGLIDIALIPFFSFFYAFETLEEECPEIVAWAKRCSQRETVSKSEVLQ
HKAYEFVL
>Pt-GSTU30
QVTLLDFWPSPFGMRVRLALAEKGVYEYSEEDLRNKSALLLQMNPVNKQIPVLVHNGVCE
SLIIVQYIDEVWKDSALLPYQRAQSRFWADFVDKKIYDLKKGEEQEAAKKDFIDSLKLME
GELGDKPYFGGTIGYVDIALVPFYSWFYAYETIEAECPKMIAYCKRCLQKETVSKALEPQ
KVYDFVL

>Pt-GSTU31
EVTLLDFWASPFGMRVKIALAEKGVYEYSEQDLRDKSALLLQMNPVYKKIPVLVHRGVCE
SLIIVQYIDDVWRGKTLLPYERAQSMFWADFIDKKIHDLTKGEEMEAAKKGFFECLELLE
GELGEKPYFGGTLGYVDIAFLPFCCGFSTYETIEAQCPKIIAWAKRCLQKDSVAKSLAPG
KVHELVM
>Pt-GSTU32
TLMLEFQAGAPSSVRVRIALAEKGVYDVYSKQNPRDEITKLLLQMNSVSKQLPTLIHNGV
CESLIIVQYVDDVWKGKALLPYQRAQSRFWADFIDKKVFEITKGEELEGAKKDFIECLKL
LEGEHGDKPYFGGNLGYVDVAFVPFYCWFYAYETCEAECPKIIAWAKRCMQKKSVSKSLE
PKKVYEFVL
>Pt-GSTU33
EVTLLDFWASPFGIRVRIALAEKGVYEYSEQNLRDKSALLLQMNPVYKKIPTLIHNGVCE
SLIIVQYVDDAWKGKALLPYQRAQSRFWADFIDKKVFEITKGEELEGAKKDFIECLKLLE
GELGDKPYFGGNLGYVDVAFVPFYCWFYAYETCEAECPKIIAWAKRCMQKESVSKSLEPK
KVYEFVL
>Pt-GSTU34
DLLLLDFWVSPFCMRVKIALAEKGLYESKEEDLFGGKSELLLKSNPVYQKVPVLLHNGLN
ESAIIVGYIDEKWPSPLLAYGRSQARFWADYIDKKVIDASTGEAVEVAKKDFIEVLKVLE
EALGEKTFFGGTFGFVDIVAIPMASWFYASEKFEAECPKLSAWIKRSMQRESVAKVLPPE
KVYDFVV
>Pt-GSTU35
DLLLLDFWVSPFCMRVKIALAEKGLYESKEEDLFGGKSELLLKSNPVYQKVPVLLHNGLN
ESAIIVGYIDEKWPSPLLAYGRSQARFWADYIDKKVFDASTGEAVEVAKKDFIEVLKVLE
EALGEKKFFGGTFGFVDIVAIPMASWFYASEKFEAECPKLSAWIKRSMQRESVAKALPPE
KVYDFVV
>Pt-GSTU36
EVKLYGFWPSPFSHRIIWALKLKGVYEYIEEDLSNKSESLLKYNPVYKKIPVLVHGGIAE
SLVILEYIEETWPENPLLPYERAMARFWIQYGVIKGASSGEELEKAAKELAEWLKILEEQ
GLDKKFFGGESINLVDIAYGALGYWFAAMEEAEPSTFPRLHAWAKNFVELPVVKENIPAY
DKMLAYRT
>Pt-GSTU37
EVKLYGFWPSPFSHRIIWALKLKGVYEYIEEDLSNKSESLLKYNQVYKKIPVLVHGGIAE
SLVILEYIEETWPDNPLLPYERAMARFWIQSGVIKGAASGEELEKAAKEMSEWLKILEEQ
GLDKKFFGGESINLVDISHGALGYWFAALEEAEPSTFPRLHAWAKNFVELPVVKENIPAY
DKMLAYVT
>Pt-GSTU38
EVKLYGFWPSPFSHRIIWALKLKGVYEYIEEDLSNKSESLLKYNPVYKKIPVLVHGGIAE
SLVILEYIEETWPENPLLPYERAMARFWIQYGVIKGASSGEELEKAAKELAEWLKILEEQ
GLDKKFFGGESINLVDIAYGALGYWFAAMEEAEPATLPRLHAWAKNFVELPVVKENIPGY
DKMLAYRT
>Pt-GSTU39
EVTLLNFWASPFGMRVKIALAEKEVYEYSEQDLMNGKSDLLLQMNPVYKKIPVLVHRGVC
ESLIIVQYIDDVWRDKALLPHERAQSMFWADFIDKKINDLTKGEELEAAKKGFFELKLLE
GELGEKPYFGGTLGYVDIAFLPFCCGFSTYETIEAQCPKIIAWAKRCLQKESVAKSLAPG
KLHELVL
>Pt-GSTU40
EVVLLDLKASPFAARVRIALEEKGIYKSQVEDLSNKSSTLLKMNPVHQQIPVLIHNGICE
SMVIVQYIDEAWSHKPLLPYRRAHARFWADYIDKKIYPISEGEVKESSKKDLIQCFKILE
EQLGDKLYFGDSFGYIDLALIPFYSFFYTFETLVAEFPKLVEWGERCLQKESVSKSLSQK
EVYEVIL
>Pt-GSTU41
GVKLFKTWSSPFGIRIVWALKVKGVFEQIDEDLVNKSPLLLLYNPVHKKIPVLVHDGVVE
SLIILEYIEGTWKQNPLFPLERAAARFWAKFGDDKVMPSGREEEECAFAPAFENLKFLEE
ELKKQFFGGERIGIVDIAFGWLANLVPVFEEIDEERFPLLHAWMQEFSKAPVIADCRPPH
EKLVNKFR
>Pt-GSTU42
MADEVTLLFWASSEQDLSNGKSDLLLQMNPVYKKIPVLVHRGVCESLIILQYIDDVWRDK
ALLPYERAQSMFWADFIDKKVITFQQKEKNRRQRRVSLNALSILEGELGEKPYFGGTLGY
GILPLLPFCCGFSTYETIEAQCPKIIAWANGCLQKESVSKSLAPGKVHELLS
>Pt-GSTU43
GVKLFGLSPSPFSQRVIWALKLKGIYEYVEDDLSNKSNLLLQYNPIYKKIPVLVHDGIAE

SMVILEYIDNTWPENPLLPHERSLVRFWAKFIDERLKPVGEQQEKAINDNLQTLRIIEEH
GLEQKFLSGDRIGLADIALGWIIHTLAAMEEIQADTFPLLHAWMKNFREIPVIKDNLPSH
DQILDYFK
>Pt-GSTU44
EVKLLGAWPSTFSYRVLWALKLKGVYEFVEENLSNKSELLLQYNPVHKKIPVLIHGGIAE
STIILEYIEETWPQNPLLPYERAMARFWTKFGEDKSPTVGEGQEKATKEFKELLGIIEEL
GLDKKFFGGDKVGMTDIAFGWIAGWLQPMEEAEPGSFPRLHLWTQNFKEVAVIKENLPDY
DEMLAYFK
>Pt-GSTU45
EVKLLGAWGSPFSRRVEMALKLKGVYEYIDEDLANKSPLLLKYNPIHKKVPVLLHNGMAE
SLVILEYIDETWKSNPILPYDKAMARFWAKFIDEKCMPAKENEREKAIEEAIQHLKTLEN
ELKKKFFGGETIGLVDIVANFIGFWLGAAQEANKERFPVLCKWIDEYANCSVVKENLPPR
DKLIAFLR
>Pt-GSTU46
AVKLIGSYTSLFCTRVEWALKLKGVYEYVEDDVFNKSPLLLKHNPVHKKVPVLVHDDIPE
SLVILQYIDETWNDNNLLSYERAMAHFWAKFTEEKCLMEEGEEKERAIESAQESFAFLEK
LIQKKFFSGDKIGYLDLAMGWIPLCLDIIEEVDAEKFPSLLEWAQNFTEIPLIKERLPPR
DALFDYFH
>Pt-GSTU47
EVKLLGTWPSPFSRVIWALKLKGIYEYIEEDLSNKSPLLLQCNPVHKKIPVLIHGGICES
MVILEYLEETWPQIPLMPYERARARFWVKFVEDKGVASGEEQEKAVKDSLEMLKTIEEHA
LKKRFFCGDKISLVDIAYGWIAQWLEVLEEVEPQKFPRLHTWIKNFKDEPIIKENLPGHD
EMLVYFK
>Pt-GSTU48
MWVWLQGRVIKVLTALKLKGIYESFEEDLSNKSPLLLQCNPVHKKIPVLIHGGICESMVI
LEYMEETWPQIPLMPYERARARFWVKFVEDKGVASGEEQEKAVKDSLEMLKTIEEHALKK
RFFCGDKISLVDIAYGWIAQWLAVLEEVEPQKFPRLHTWIKNFKDEPIIKENLPGHDEML
VYFK
>PtGSTU49
AVTLLDTWARPFGMRVRIALEEKGVYEYTEQDLRNKSPLLLQMNPVYKKVPVLLHNGICE
SLIAVEYVDEMWNNKSLLHPKRAQSRFWSDFADKKVYRSEKGEEKEAAFIEFIESLKLLE
GELGDKPYFEGKLGYVDVALIPFYCWFHGYETFEECPKLIAWCKRCMQKESVSKSLAPQK
IYIFMA
>Pt-GSTU50
EVKLLGTWPSPFSYRVIWALKLKGIYEYIEEDLSNKSPLLLQCNPVHKKIPVLIHGGICE
SLVILEYMEETWPQIPLMPYERARARFWVKFVEDKGVASGEEQEKAVKDSLEMLKTIEEH
ALKKRFFCGDKISLVDIAYGWIAQWLEVLEEVEPQKFPRLHTWIKNFKDEPIIKENLPGH
DEMLVYFK
>Pt-GSTU51
DVKLIGAWPSPFVMRPRIALNIKSAGYEFLEETLGSKSQLLLESNPVHKKIPVLIHDGIC
ESLVIVEYIDEVWSSTILPYDRALARFWAAYLDEKGFKEEEARKALIEQAGEGVMMLEDA
FSKGKGFFGGDQIGYLDIAFGSFLGWLRAIEKMDETKTPSLLKWATSFSSHPAVKDVLPE
TQKLVEFAK
>Pt-GSTU52
QVKLLGFWASPFARRVEWALKLKGVYEYIEEDIFNKSSLLMELNPVHKKVPVLVHGRIAE
SFVILEFIDETWKQCPLMPYERAKTRFWAKFAEEKILDLGEEKEKAIKLAVEAIEKIEGE
LKKHFFGGENIGYLDIAMGWMSYWLPVWEEVNPLQFPATTSWMNKFLDHPVIKENLPPRD
KMIIYFQ
>Pt-GSTU53
EVKLLGTWPSPFSYRVIWALKLKGIYEYIEEDLSNKSPLLLQCNPVHKKIPVLIHGGICE
SLVILEYMEETWPQIPLMPYERARARFWVKFVEDKGVASGEEQEKAVKDSLEMLKTIEEH
ALKKRFFCGDKISLVDIAYGWIAQWLAVLEEVEPQKFPRLHTWIKNFKDEPIIKENLPGH
DEMLVYFK
>Pt-GSTU54
QVTLLDLWASPFGMRVRIALAEKGVYEYSEQDLWNKGALLLQMNPVHKKIPVLVHDGICE
SLIIVQYIDEVSKDKALLPYERAQSRFWADFVDKKLYELTKGEDQEAAKKDFIDSLKLLE
GELGDKPYFGGTLGYVDVALLPFYCWFYAYETIEADCPKLIAYCKRCLEKESVSKSLEPQ
KVYDFVV
>Pt-GSTU55
EVVLLDLKLSPFAARVRIALEEKGIYKSKVEDLSNKSSTLLKMNPVHQKIPVLIHNGICE
SMVIVQYIDEVWSHKPLLPYRRAHARFWADYIDKKIYPISEGEVKESSKKDLIQCFKILE

EQLGDKLYFGDSFGYIDLALIPFYSFFYTFETLVAEFPKLVEWGERCLQKESVSKSLSQK
EVYEVIL
>Pt-GSTU56
GIKLLDSWASPAAMKVRIALAEKGIYESMEEDLPHKSPLLLEMNPVHKRIPVLIHNGICE
SMIIVEYIDEVWNDRSLLVQERTRARFWVHLIDKKIYSLSSSDNKKAAAKDLIEFFKVIE
GELGDKPYFGGGFGFVDVALVPFYGYFYTYETFAIECPKLVEWGKRCLQKESVSKNLPPY
KAYEFVM
>Pt-GSTU57
KVKLYGMWASTYVKRVEVALRAKGICEYIEEDLSNKSQALLQYDPVHNEVPVLVHNGITE
SSIILEYIDETWKQPRLLPYQRAKVRFWASFIQQQLFEGGGEAQEKAIGELLEKMNIFEE
EMKPNGVSVIEVQNLGLLDILVGAVFSPYKAQEEVDPEKNPLILSWVTAWNQLTTVQELL
PPHDKIVGLLQ
>Pt-GSTU58
QVTLLDFWASPVGMRVRIALAEKGVYEYKEQSLRNKSVLLLEMNPVHRKIPVLIHNGICE
SLNIVQYIDETWKQSLLPYERTQSMFWADFVDQKVCKAEGEEQEAAKKDFIDCLKLLEGE
LGAKPYFGGTLGFVDVALVPFYCWFHAYETMEAECPKLIAWCKRCMQKDGVSKSLAPRKI
YNYAM
>Pt-GSTF1
PVTIYGPPLSTAVSRVLATLIEKDVFHLVPIDLSKGEQKKPEYLKQPFQVPAFKDESITL
FESRAICRYICDKYADKGNSLYILSKANIDQWVETDGQTFGPPGDLVHDLLFSSVPEALI
KKNVDKLAKVLDIYEQKLGQTRFLAGDEFSFADLSHLPNGDYLVNSTDKGYLFTSRKNVN
RWWTEISNRESWKKVLEMRKNA
>Pt-GSTF2
PVKVYGPPLSTAVSRVLVTLLEKDVFQIIPVDMSKGEHKKPDYLKQPFQVPAFQDESISL
FESRSICRYVCEKYADRGDGLYPLERASIDQWVEAEGQSFGPSGALVFQLAFAPRMNIPQ
GVIKQNEEKLGKVLDIYEQRLGESRFLAGDEFTFADLSHLPNGDYLVNATDKGHLFTSRE
NVGRWWNEISDRESWKKVIEMRKSG
>Pt-GSTF3
ALKLYGAPMSTCTSRVLTCLHEKDLFELVIVDLFAGEHKQPPFLANPFQIPALEDDDLTL
FESRAITSYVAERFKETGYLIRIKEAALVKVWTEVESQQFHPAAPIIFQFLVAPLQGNLP
QTIIDTNLEKLGKVLDIYEAKLTSTKYLAGDFYSLADLHHLPYAYYLMKTPAASVVNERP
HVKAWWEDISSRPAFKKVAEGMNFGEK
>Pt-GSTF7
VLKLYGAPMSTCTSRVLTCLHEKNLFELVPVDLFAGEHKQPPFLANPFQIPALEEDDLTL
FESRAITSYLAEKFKGTGYLIRLKEAASVKVWTEVESHRYNPAAPIVFQFMVAPLRGNSP
QTIIDDNVEKLGKVLDIYEAKLSSTKYLAGDFYSLADLHHLPYTYYLMKTPAASVVNERP
HVKAWWEDISSRPAFKKVAEGMNFVKK
>Pt-GSTF9
MSTCTARVLLCLSEKGLYDWCRSTRCRREPISNNLIHLPFQIPAFEDTFALLSESRAICK
YLLRSSSFTESSIVDEWMEAEAHRFIIGIIVIPAFQLVPEKVIGTELEKLGKVLDVYEER
LSKCRYLAGHYTMADMNHIPCLFYFMKTPYATAVTSRSPSK
>Pt-GSTF8
VVKVYGPAMAVCPQRVMACLLEKGVFDLVHVDLDSGEQKLPEFLLQPFQVPVVEDGDFKL
FESRAIIRYYAAKYEDRGPNLLLEEKALVDQWLEIEAHNFNDLFNIVFQVVILPRIGQQG
SELVRTYEEKLEKVLDVYEQRLSKSKYLAGDSFTLADLSHLPATRYLVNEAGLGHLVKDR
KKLNAWWEDISSRPAWKKLINLAGF
>Pt-GSTF5
PLKLHGSVLSTNTQRVLATLYEKEVFELVNVNLGAGEHKQEPHISNPFQVPAAVDGDLKL
FESRAISQYVAHQYASKGTQLGNGYATILVWQEVESHQFDPSSKLVWEQVFKPVFGLPTA
ALVAETEVTLGKVLDVYEARLSQSKYLASDSFTLADLHHLPNIQALLGTPSKKLFDSRPH
VSAWVASITGRPAWGKVLALLPK
>Pt-GSTF6
TLKLHGTPISTNTQRVLATLYEKEVFELVNVNLGAGEHKQEPHISNPFQVPAAVDGDLKL
FESRAISQYVAHQYASKGTQLGNGYATILVWQEVESHQFDPSSKLVWEQVFKPVFGLPTA
ALVAETEVTLGKVLDVYEARLSQSKYLASDSFTLADLHHLPNIQALLGTPSKKLFDSRPH
VSAWVASITGRPAWGKVLALLPK
>Pt-GSTF4
IIKVHGSTLSTAAQRVFACLYEKELFEFIPVNMAVGEHKKEPFLANPFQVPAFEQGDLKL
FESRAITQYIAHGYADKGTPLVKQMATLSVWMEVEAHQFDPVSKLNWELVFKPMFGIPTN
AAVEENEAKLGKVLDVYESRLAQSKYLGGDIFTLADLHHLPNISCAMRTHVKKLFDSRPH
VSAWVADITSRPAWSKVVAMNK

>Pt-TCHQD1
MQLYHHPYSLDSQKVRLALEEKGIYTSNHVNPITGKNMDASFFINQSKLPVFQNGSHIIF
DTIEIIQYIERLGGGSFSSREVVEWMCKIQEWNPKYFTLSHVPEKYRISVSKFIRQVIIA
RVLKRSKEHLVRLLDEVETKLKETAYLAGEEFSMADVMLIPVLARLVLLKLEDEYISSRP
NIAAYWVLMQQRPSYKKVIGKYFNGWRRYK
>Pt-EF1B¦Ã2
ALVLYAGKTNKNAYKALIAAEYSGVVKLAENFEMGVTNKTPEFLMNPLKVPVLETPDGPI
FESNAIARYVTRLKADNPLYDYAHIEQWMDFAATEIDAGISRWLYPRMGFQPYLPPAEEA
AISALKRALGALNLHLASNTYLVGHSVTLADIILTCNLFLGFAHVMTKSFTSEFPHVERY
FWTMINQPNVKKVMGEVKQAEVVVP
>Pt-EF1B¦Ã3
ALILHAGKTNKNAYKTLIAAEYSGVVKLAENFEMRVTNKTPEFLMNPIKVPVLETPEGPV
FESNAIARYVTRLKADNHLYEYARIEQWIDFAATEIDAGISKWLYPRLGYQPYLPPAEEA
AIFALKRALGALNLHLTSNTYLVGHSVTLADIIMTCNLYTGFSYVMTKSFTSEFPHVERY
FWTMVNQPNVKKVVGEVKQAESVLP
>Pt-EF1B¦Ã1
ALVLHAGSTNKNALKTLIAAEYSGVVELVKNFEMGVSNKTPEFLMNPIKVPVLETPDGPI
FESNAIARYVTRLKADNPLYEYARIEQWIDFATLEIDANILRWFIPRIGFAVYLPPAEEA
AIAALKRALTALNTHLSTSTYLVGHSLTLADIVLTCNLTLGFSRLLTKTFTSEFPHVERY
FWTMVNQPNFRKILGEVKQTESVPP
>Pt-GSTZ1
KLVLYNFSHSSCSWRVRFALNLKGLYEYKAVNLAKGEQFRTEFEQLNPLYVPVLVDGDVV
VSDSLAILLYLEEKYPQRALLPRRKALNLQVASIVCSSIQPLMLALVKRIEEKVGPEEGL
LWAQSSIEKGFFALEQLVKDFATRFATGEALYMADVFLAPQIATVVMRFNIDMSNFPILS
RVYESYKTVPEFRASSPEAQPDAGS
>Pt-GSTZ2
KLKLYSYWRSSCSQRVRIALNLKGLYEYIPVNLLKGEHFSPDFLKLNPLYVPALVDGEIV
ISDSFAILMYLEEKYPQHPLLLQKKALNYQAANVVCSSIQPLNLAVLKYIKEKVGPDEVI
PWVQSHINKGFAALEKLLKDSAGKYATGNEVSMADLFIEPQIHGAIKRFNVDMTQFPLLS
RLHVAYSELPAFQNAMPENQPDASPSS
>Pt-GSTT1
KLKVYADRMSQPSRAVLIFCKVNRIFEEVRVDISKRQHLTPEFKEINPMKLPAIVDGRFK
LFESHAILIYLACVFPGVADRYLFKRAKINSVLDWHHSNLRHGAAEYVKNTTLAPVLGLP
LDPQAAAEAEKVLFSSLSKIESVWLKGSGRFLLGGNQPSIADLSLVCELMQLEVLDEKDC
SRILCPYKKVQQWMEDTKNRPHFDEVHQILFKAKVKLQ
>Pt-GSTT2
ELKVYVDRLSQPSRAIVIFCKVNKIFEEVGIELLKGQHLTPEFKEINPMKVPAIVVDGKF
KLFESHAILIFLASAFPGVADWYLYRRAEIHSILDWHHSNLRRGSVEFIQNTLLAPFFGR
PLNPQAAAEGEKVLSSSLSKIEALWLKESGQFLLGSSQPSIVDVCLVCEIMQLEFTDETD
RNCILGPHKKIQQWIEDTKNKPHFDEVHQALFAAKVKLQ
>Pt-GSTL2
LPRLYTCYTCPFAHRVWITRNFKGLDEIKLVPLILQNRPAWYSEVYPPKVPSLEHTGESL
DLIKYLESNFQGPSLLPAKKEFAEELFSYTDTFNRTVFTSFKGDPAKEAGPAFDHLENAL
HKFGDGPFFLGQEFSLVDIAYIPFVERFCIFLSEVFKYDITAGRPKLAAWIEELNKIEAY
KQTKTDP
>Pt-GSTL3
SLRLYTCYTCPFAQRVWITRNFKGLDEIKLVPLILQNRPAWYPEVYPPKVPSLEHTGESL
DLIKYLESNFEGPSLLPAKKEFAEELFSYTDKFNGTVYTAFKGDLAKSGPAFDYLENALH
KFDDGPFFLGKCCQVDIAYIPFVERLNIFLLEVFKYDIAAGRQKLAAWIEEVNKIEAYKQ
TKTDP
>Pt-GSTL1
QQRLYISYTCPYAQRVWITRNCKGLDKIKLVPIDLQDRPAWYKEVYPPKVPSLEHKGESL
DLIKYIDSHFDGPSLFPAKKEFAEDLFSYTGSFSKANNSTFKGEADEAGAAFDYIETALS
KFDDGPFFLGQFSLVDIAYAPFIERFQPALLEFKKYDITAGRPKLAAWIEEMNKIEAYNQ
TRREP
>Pt-DHAR2
PDILGDCPFSQRALLTLEEKKIYKSHLINLSDKPQWFLEVNPEKVPVVKFVSDSDVIVGI
LEEKYPEPSLAPPEFASVGSKIFPSFVKFLKSKDPNDGTEQALLEELEALDDHLKAHGPF
IAGEKITAVDLSLAPKLYHLEVALAHFKNWTIPDKLTHVLNYIKLLFSRESFEKTKAAKE
>Pt-DHAR3
PNILGDCPFCQRVLLSLEEKKIYKSHLIDLGDKPQWFLEISPEKVPVVKIVADSDVIVGI

LEEKNPEPPLAPPEFASVGSKIFPSFVKFLKSKDPNDGTEQALLEELKALDEHLKVHGPF
IAGEKITAVDLSLAPKLYHLEVALGHFRNWTIPDNLTHVLNYIKLLFSRESFKKTRAAEE
>Pt-DHAR1
PDKLGDCPFCQRVLLTLEEKNLYDMKFVDLGNKPEWFLKLNPDKVPVIKFVSDSDVITQA
LEEKFPDPPLAPPEKASVGSKIFSTFIGFLKSKDPGDGTEQALLDELSAFNDHIKENGPF
INGEKVSAADLALGPKLYHLEIALGHYKNWSVPESLPYIKSYLKEIFSRDSFVNTRALPE
>Ec-GRX2
MKLYIYDHCPYCLKARMIFGLKNIVELHVLLNDDAETPTMVGQQVPILQKDMPESMDIVH
YVDKLDGKPLLTRSPAIEEWLRKVNGYANKLLLPRFAKSADEFSTPAARKYFVDKKEASA
GNFADLLAHSDGLIKNISDDLRALDKLIVKPNAVNGELSEDDIQLFPLLRNLTLVAGINW
PSRVADYRDNMAKQTQINLLSSMA
>Pt-GHR1
RYHLYVSYACPWASRCLAYLKIKGLIAFTSVPLNGARSIRELYELASTNTVPVLWDKKLK
NNESSEIIRMFNTEFNDIAEAALSHLQARIDETNEWVYNGINNGYKCGFARKQGPYEEAA
IQLYEALDKCEEILGRQRYICGNTLSEADIKLFVTLIRFDEVCNKKLLRDYPNMFNYTKD
IFQIPGMSSTVNMQHIKRHYYG
>Pt-GHR2
TLHLYVGLPCPWAHRTLIVRALKGLVPVSIANANGCRNLKGVYGLRSGGTVPMLWDVEKK
CNESYDIIEFFNSGLNGLARPGLKELKGKIGEWNGLIYPNVNNGYRCGFAQSQDAYDSAV
NGLFTTLEAVEDHLTTSRYLCGDTLTLADVCLFTTLIRFDIVCTKKKLIEYPNLHGYMRD
IYQMPKVAETCNFSAIMDGYYK
>PpGSTI1@KC119478
FYRDNSSWCPYCQRVWLQLEEKKIYQVEKINMRCYGDKPAWFTKMVPSLLPVIELITESM
DIMILIEKRFPEFNPLLGPELAAVNSLLGLERRLAGAWMNRLRSSWPDMGAFENTMDKVN
SALQTFGGPYFLGSKFSLVDAVYAPFLERTAASMPYWPGVKVRGNDRWNAVNLWFDAMDS
RPSYQAMKSDDF
>PpGSTZ1 @KC119480
AVTLWGFYASSCTWRVRLALGLKGIYKYKALNISNGEHKTEEFRKISPLYVPAVEVDGGT
IADSLAIIMYFEEKYPDKKPLLLLKRATVRQVVYLIASNIQPLNLGTLKMIEAQFGADAR
PKWAQDHIIIGFTALEQLLQNVAGKYTVGDELTLADVVLVPQIGNAKRFQVDLTQFPIID
RIGKALLELPEVQASLPANQPDAPK
>PpTCHQD1@KC119470
MQFYHHPLSMNSQKVRLALEEKNIYTAFRVNPLKARNLDAEFFQNPNTLPMLKIGAHVLC
ESLPILQHIDKLNEPLKDYRERAEEWIKKIDEWDAKPFTLTHVPDRMIRFFGKFKRRVLI
ARMAKNPDLANKYHSKLNSMHAMEEQLKDTQAVDSNRKELVLMLDDAEHQLAGTEFMAGD
VFSVADAAFIPVLARIELLKLSEEYLRPRPKLLDYWERMKCRPSYKKVIGGYSSQLKQLK
>PpTCHQD2@KC119471
LAIFYNYALAFNPAKSRLALEEKNIYVETKIDLFNGQSLEPWYLLNPSSAPTLVVGDEKI
TESVEIIRWADRQGAPLDRAFVDEWLNKVDAWDGNLFAAANSPAGALKYSTEYKFKVAEA
NAKRNPDMAELYKEKISTLKKNYIDEPNDEAVCDANRQQLRVLLDEAETRLATNKFLAGP
AYSAADAIFTPVIYRIYLLKKDGEYLSSRPNIKRYYEEIKKRPSYKKVFSVSDSALASAG
>PpTCHQD3@KC119472
SIIFYNYPLAFNPAKAKLALEEKGIYTEKKIDLFNGQSLEPWYLLNPASSPTLVVGDEKI
VESADIIRWADSQGAPLDRAFVKEWLEKVDAWDGNLFAAANSSSSAIKFGTKHKIKVAEA
NMKRNPDMAELYKKKIATMEAQLAEPDNREAVEQNLRQLAGLLDEAEARLSTNKFLAGDS
YSAADCIFTPVIYRLFMVKKDKEFLEPRPNIQRYYDELKKRPSYKKVFGVADSGFCTAQ
>PpTCHQD4@KC119473
IPEFYNYPLAFNPAKVKLALEEKSLYTEKHIDIFNGQSLTPGYMLNPHWSPTLVTKEEIL
TESLDIVKWADRQGSSLDRTFVSEWLTKVNAWDGNLFAMFNSPARMFRYVTKFKIKTAES
YAQRFPDLADVYRKKVVSMKQVLREVDDAETVNANMAQLVALLDEAEMRLRNFKFLAGNQ
YSVADVIFTPVVYRLFSTKKDQEYLNSRPHIRTYYQDLKTRSSYKKVFGISDTSLGTMW
>PpTCHQD5@KC119474
SVILYNYSLAFNPAKAKLALEEKGIYTEEKIDLFNGQSLEPWYLLNPASSPTLVVGSEKI
VESADIIRWADKQGIPLDRAFVNEWLQKVDAWDGNLFAEGNSSSGAVKLSTEYKIKVAEA
NATRYPDLAELYKKKLVSLKKSIERPSNSDAREKNLKQLSDLLDEAEARLSSTKFLAGDA
YSAADIIFTPVLFRVYQVKKDAELLDSRPNIKLYYEELKKRPSFKPVFAAAESGLLTAL
>PpDHAR1@KC119456
PDKFGDCPFSHRVVLTLAEKKVYDMKLIDVSNKPQWFLDINPEKVPVIKDVADSDVITQL
LEEKYPEPCLKPEDKASAGARIFPNFAAFLKSKDPNDGTEAALLAELKSLDEHLKSNKPF
IAGEAVTAADLALAPKLHHLTVALGHYKKWSIPEDLTNVLSYVEAVHSLESFKKTKPADE
>PpDHAR2@KC119457

SPSKERGDCPFSQRIYIELEEKKLYTATYIEEGENKPDWFMEKNPKLMPVLRDIQDSDKI
AEHLEKKYPEVSLAPKEYKQIGLNIFQAFTTYLKSKNADDQSKQELLKELAALDQHLQTK
GPYIAGENPTDSDYALIPKLHHMRVSLAHYMGFKIPSEHKALHKYIKLLESRPSFQKTNS
PDD
>PpDHAR3@KC119458
PCKLGDCPFSQRVLITCELKNIYDVKFVDLDRKPEWFLRINPERVPVIKIIPDSDIIVDV
LEKSYPYPPLSCRNITCRGQNIFPAGMAFFKSKPRCDGTESQFVCELDHMNHHLCNEGPY
IAGQYVTSADIALAPQLYVLQTALAYYKNWTNFEQFYPALNLFMKVYSHKYHLKTHARPS
>PpGSTL1@KC119459
TTRLYFSSRCPYAQRVWVAVKYKGLEIECVEISLSDKPTWYKEVYPVKVPALEHTGESMD
LLTYLDDHFGGPKLAESKKQAAAELLQYADTFNKLGFTGLSMKSTPDEIAAAVAPAFDFL
ENALAKFSSEGPLFLGNFGLVDIVYAPFIERFEIAFGGIRNYDIRAGRPRLAKWIEAMDN
VEAYSSTKVPR
>PpGSTF1@KC119460
AIIVHGAGFSTCTQRTLTTLAELNVYKLEFVDMMKGVHKSPEFLKQPFQIPVLEDGDIQI
FESRAIIRYLAEKFEGQGTPLLVKDKALVSQWLEVESQNYNPPAQIVYQRVFSSMFGSSC
EDVVTEQVAKLEKVLDVYEAHLSNNKYLAGDFFSLADLSHLPYTYLLVNAAGKSDLITSR
PHVSAWWNDISSRPTFQKVLGMNEFNKKE
>PpGSTF2@KC119461
VMIVYGDIGLNPYRAIQPLFRSSGVLKLVTVSLKAGHFLLPSYKAQPFLIPCLEDGDLIV
FESRAIARYLAEKYEGQGTPLLLKERAIINQWAESEAQNFQPAGPMVREAFVAAFGIRPV
EEVVAAGMKKLDQVLDVYEAHLAKGTKYLAGDDFSLADLFHTVYMNWMKSARPELLEKRP
HLSAWIHDITTRPAFLRCLQLDWENASPIQ
>PpGSTF3@KC119462
AMILYGGPTPNVIRSILPLFEAEVLKLVRVALQTAQHRQPIYTTQPFQIPFFEDGDVKIF
ESRAIARYIAEKYEGQGTPLLLKERAIIYQWIESEGQNFHPCGPVVREMFVAPREKRPVE
EVISTCLAKLNNVLNVYEAHLAKGSKFIAGESFSLADAFHTPYMNWVKNVKPELLENRPH
VSAWVEAITSRPAFQKCLQLDWENAAALE
>PpGSTF4@KC119463
VMILYGDKALNVYRAIQPLFEAQVLKLVTVSLKAGQFRLPSYKAQPFLIPFLEDGDFTVF
ESRAIARYLAEKYEGQNTPLLLKERATINQWAESEAQNFHPAAPMVRELFLAPFEKRPVE
EVMETGMNKLDKVLDVYEVHLAKGTKYMAGDNFSMADLFHTVYMNWMKSRWPQLLEKRPH
LSAWIHDITTRPAFLRCLQLDWENASPIE
>PpGSTF5@KC119464
VMKLYGMKNIFNVQRAIVPLFEAGTLELVHVNLFTHDNDKPNFLAKHPFLIPYLEDGDVK
IYESRAIARYIAEKYEGQGANMLKERAAINQWVETEAHVLYPPLAPMLKELYVAGALNRP
LDEELVASCTAALVKVLDVYEAHLVKGSKYLTGDEYNFADACHTPYLYQVKIMKAEVLRH
HPYVWAYTDAITERPGFQKLLQLDWDNAPSLE
>PpGSTF6@KC119465
VMIVYGDIGLYRPIQPLFEAQVRGSSKLVTVSLKAGHFLLPSYKAKQPFLIPCLEDADLT
VFESRAIARYLAEKYEGQGTLLLKERAIINQWADSEAQNFQPATGPMVREAFVAAFGIRP
VDEEVVAAGMKKLDQVLDVCEAHLAKGTKYLAGDDFSLADLFHTVYMNWMKSARPELLEK
RPHLSAWIHDITTRPAFLRCLQLDWENASPIQ
>PpGSTF7@KC119466
APKLYGTYYSLATAKAIAVMLEKEVFELVRVSVKDGEHRKPEYLAQPFLVPLLEDEDLKL
FESGAIMRYIADKYEAQGTKLYKAERALVEQWMEVEVGTWGAHQVLVRELIFTPMLQKKP
QQTVEDTKVKLQKVMDTYEAHLTKHQYLAGDFVSIADLVHLPVSYLILNEYGMPGLLSSR
PRVAAWWEAITSRPSWKEIVKNSGADWESWV
>PpGSTF8@KC119467
ATKLYGTYYSSATARAIVVMLEKEVFELISTSILLGEHKKPEYMTQPFLVPLLEDEDLKL
FESGAIMRYIADKYEAQGTKLYKAERALVEQWMEVESGTFSALRTLVKELIYGPGLFKIA
QKAVEDATEKLNKVLDVYEAQLTKHQYLAGDFVSIADLGHLPLSNLYFNVLGKRELLSSR
PRVAAWWGAILSRASWKKIVSFAGADYESWV
>PpGSTF9@KC119468
KLILHGVASSTCTGRVLVALLEKGVFELKVVNVRKGEGKTPEHKAQPFLIPVLQDGSLTL
FESRAIIRYIANKFEGQGTPIYPVEKALVEQWLEVEGQNFNAAKECLKYAAASPVQEKLA
QAMEKFSKVLDIYEARLSDTQFLAGDFFSLADLSHLTTGWKLFDKYKQGVVCFEGRPHVK
AWWAAISSRPAWKRVLEMI
>PpGSTF10@KC119469
GIKVYGIATYNCQRVTLALLEFGVFELVKVYLKAGEHDAPSWTSTPFQVPVFECGDFHLY
ESRAIVRYIARKYKGQGPSLFLEEQAIVDQWSEAEAYQFSFSLPWSLEYYVGRREERPLE

GVLKPFYDKLATFFDICESQLSKTKYLAGDFYSVVDLNIAPSLLRVMTLKPELITTRKHV
KRWYDSLASRPAFRKMLQADEHWAFALN
>PpEF1B?1@KC119481
GLVIHSQKVNKNSYKSLIAAEYVGVVEIVPDFQMGVTNKSPEFLMNPIKVPVLQTPEGPI
FESNAMARYVANKKDVGLVEKALVEQWIDFATMEIDVNAGGWVYPRLGFGLYNEEVEASK
ISNLKRALTFLNAHLASRTYLVGESITLADIVLTCNMIVLVKLAATKEFTSEFPHVERYF
WTLVNQPNFKKIIGEVSQAAQPLG
>PpEF1B?2@KC119482
GLVLHSPKVNKNAYKTLIAAEYVGVVETTPDFQMGVTNKTPEFLLNPIKVPVLQTPDGPI
FESNAMARYVANKKDVGLTEKALVEQWIDFSSLEVDANIGRWVYPRLGYFAFIEEVEAFA
ISNLKRALTCLNGHLASRTYLVGESVTLADIVLTCNLATLFKVAATKEFTSEYPHVERYF
WTLVNQPNFKKIVGEVAQAVKPLG
>PpEF1B?3@KC119483
EQKLHANKLNKNACKSLIAAEYVGFLETTPGFELTDTISPEYLMNPMKAPVLETPEGSIS
ESNAIAHYVAGLKNVGLFEKALVDQWIDFGSLEIDTNALRWAYPRMGLAFFSEIEASSIN
NLKRALTALNAHLASRTYLVGESVTLADIVLSCNLSLPFYVAMTKEFTADYPHVERYFWT
LINQPNFKKVFGEFTQTAKPLG
>PpEF1B?4@KC119484
GLKLYANPVNKNAYKALIAAEYVGVIEFTEITDWSTTKSPQYLMNPMKVPVLETPEGSIF
ESNAIARYVAGLKDVGLLNKAQIDQWIDFAALEIDINARAWVLPHLGLGFFNEEVEAFII
NNLKRALTTLNSYLASRTYLVGESVTLADIVLICNLSFIWRRAATKEFTAEYPHVERYFW
TLINQPNFKKVFGEFTQADKPLG
>PpUre2p1@KC119479
KIQLYSMATPNGQKVSIALEEMELYEPHTINIFKNDQFTPEFIINPNKIPAIVDPNGPVF
ESGAILLYLAEKSGKFLLKWETIQWVFFQMGGIGPMFGQFGHFFKYAKDKCQHPYPVERY
TNEAKRLLGVLEKRLEGREFLIDGGYSIADIAIFPWVGCLDTGYGGREKVGLDNFPNVMA
WKTRCLERPKTAKGMTVCKIS
>PpGSTT1@KC119475
NLKLYADLMSQPSRAVLLFCRVNNIVDQQLINIQKGDHKTPEFKAINPLKVPCIVDGRFK
LAESHAIMEYLAATRPGVADWYVQRRSKINSVLDWHHLNLRRGSMTLVFHKVLAPIVGVK
ADPRAAAEGLSILQQSLQVLETVWLQEGYPFLAGGRQPSIADLSLVCEIMQLQVLGEEEV
AALLSSKERVTAWIAAVKKSPHFDEVHKIIFRAAANVS
>PpGSTT2@KC119476
PLTLYVDLLPQPARAVSLFCRVNKIHEEVHVEVTKGERTPRFKKINPMVPTIVDDGFKLH
ESHAILRYLATTRDVADWYPKRRALIDSVVDWHHLHLRRNAFLLVMHRVISLLPGIPKGI
YPEHDEAVAKESKTGLDHALDYIDTVLLKGPNGFLENAEEVSIADLSLVCEIKQLLVPLC
PLSVSCTSLWLASRHVLGKRSSSQPFPGLSSY
>PpGSTT3@KC119477
GLKLYVDLGSQPARAVTIFCRVNKIAHEIGVDLRKLENRTPEFRKINPMLVPCIDDHGFK
LHESHAIMRYLATTHNVADWYPQKRALVDAALDWHHLNLRKYGSQLVINRVLVKVPAHKN
FFPNLGEETRKSLADDASKTLPQILDALELMLEHGKFLHNADLVSIADLSLCCELTQLQL
LDKDDHKNMLEHRKRITTWMADVKDNPEFTDVHQAIFNYSKHVE
>PpGSTH1@KC119485
KVKLVGDVTCPYTQRVRIALLEKGVVEASLVMPDDLRGCKGEDLGISPDKFPVFQHGTGS
VDSILAYVEENFENSLLLEKEVAQWVSYIRDHFSNVIEDALNGDPYAQDSLEARLNESLA
HLDSGLELHHKEGKHFLGSKFTLVDVYLIPFLSLMDLITYIRGFAINPSYSRLCAYKSAM
CTFKCYKPVQVGT
>PpGSTH2@KC119486
KVKLVGDITCPYTQRVRIALLEKSVAETSLVMPEDLMGGKGDNLAESPDKFPVFQHGTGS
VDSILTYIEENFENPLLLEKDVAQWVSYIRDIFSNVIEDALNGDPYAQDSLEARLNESLA
CLDDGLELHHKEGKHFLGSNFTLVDVYLIPFLSLMDLVTYIRGFEINSSFTRLSAYKSAM
CTFKCYKPVQMGT
>PpGSTH3@KC119487
RVKLVGDVTCPYTQRVRIALLHKNIVNASLVMPDDLRSYRAENLSTSPDKYPVFQHGTGS
VDAMLAYIEETFENPPLLLEKEVARWVSYIRDTFTVNVLDILNGDPYAQDSLEARLNESL
ARLDSGIKPQGKHFLGSKFTLVDVYLIPFLSLVDLVTYVRGFTIDSSYSRLLSYKFSMCK
FKCYKPVQVGV
>PpGSTH4@KC119488
RVQLIGDMLCPFTLRVQIALQFKGVVDPTWLTPVDLTNPKLVASPNKYPVLHYGSGSTDA
MLDYIEETFEDPTLIVETEVMQWVAFIRDELTPIVGQLLDGSPLVQQELESKLESCFMKL
DSGIWEHGKQGRFFFGNQFTLVDVYLIPILLLVDVAKFFRGIEISTLHSHLLAYSRAMHS

FPNYAPVRVNT
>PpGSTH5@KC119489
GVELIGDLLCPFTLRVLIALQFKGIVNPTWLTPADLKNPNRIIGSPNKYPVLQYGIGSTG
TMLDYIEETFQDPPLIIRDEVMKWVAFIRDEFTPILVQLIDGSHLEQHKMTLNLESAFAE
LNNGKCEHGKHGRYFLGNQFTLVDVYLIPSLLLVDVAKFFRGITIGTVHSHLLSYSQAMH
SFSNYAPVRLDL
>PpGSTH6@KC119490
RVQLIGDVLCLFTLRVLIALQFKGVVDATWLTPADLTNPKVVTSPDKYPVLKYGVHSTDV
MLEYIEETFQDPTLIIRSEVMNWVAFIRDEFTPIVGQLVDGSPLVQQELRPNLESAFAKL
DSGKLVHGKQGRFFFGNHFTLVDVYLIPALLLVDVAKFFRGIEIGAAHPHLLSYSLGLHS
FPNYAPVRVDL
>PpGSTH7@KC119491
RVQLIGDMLCPFTLRVQIALQFKGVVDPTWLTPADLTNPKLVASPTKYPVLHYESGSTDA
MLDYIEETFEVPTLIVEAEVMQWVAFIRDELTPIVGQLLDGSPLVQQELEPKLESCFMKL
DSAIWEHGKQGRFFFGNQFTFVDVYLIPILLLVDVAKFFRGIEISTLHSHLLAYSRAMHS
FPNYSPVRMNT
>PpGSTH8@KC119492
RVQLIGDKFCPFTLRVLIALQFKGIVDAAWLTPEDLRNPNRIIASPNKYPVLQNGISSTS
AMLDYIEETFQYPTLIIKGEVIKWASFIRDEFTPTLAQLIDGNPVVQQRMTQDLKSAFAE
LNNGKRQHGKQGRFFSGSQFTLVDVYLIPTLLQVDVAKFFRGVSIDTVNSHLLSYSRAMH
SFPNYAPVRVDM
>PtaGSTU1
QVKVLNLWASPFGLRVLVGLEEKGVYEYQEENLPSKSELLLKMNPIHKKIPVLIHNDVLE
SLIIVEYIDEAWPNTNPFMAYERARARFWADFVDKKIYDNCKGEAQEEAKRNMLEYLGLL
EGALDGGIKPYFGGKFGYMDIAFIPFASWFQAWEVMLETQFPRLHEWVNACMERESVKKV
LPPEKVAEFAM
>PtaGSTU2
QVKLLGATLSPFVVRVRIALALKGIYEFIQEDLETKSELLLQSNPVYKQIPVLVHNGVCE
SMIIVQYIEEAWDNKPNLMPYDRAIARFWAAFIDDKLFPSQGEQLQKAVEDSVTNFLLIE
EALRAGKAYFGGDEIGLIDIALGGMSTFIKALEKADPKKMPLLTAWMDRFCKSDGVKEVM
PDPVKHAEYLC
>PtaGSTU3
QVKLLGVTLSPFVVRVCIALALKGIYELIQEHFMHPKSELLLKSNPVHKKVPVLIHNGVC
ESMIIVQYIEEAWGNKPNLMPYERAIARFWAAFVDDKLFPSPGEQLQKAVEESVTNFLLI
EEALRAGKVYFGGDEIGSIDIALGGLSSFIKSVEKAGAEKMPLLSAWMDRFCKIDVVKEV
MPDPAEYIS
>PtaGSTU4
QVKLLGATLSPFVVRVRIALALKGIYEFIQEHSMHLKSELLLKSNPVHKKVPVLIHNGVC
ESMIIVQYIEEAWCNKPNLMPYDRANARFWAAFVDDKLFPCQGEQLQKAVEDSVTNILLI
EEALRAGKAYFGGDQIGLIDIALGGLSAFIKSLEKADPEKMPLLTAWMDRFFKSDGVKEV
MPDVAKQAEFIS
>PtaGSTU5
QVKLLGATLSPFVVRVRIALALKGIYEFIQESMHPKSELLVKSNPVHKKIPVLIHNGVCE
SMIIVQYIEEGWGNKPNLMPYDRAIARFWAAFVDDKLFPCQGEQLQKAVEDSVTNFLLIE
EALRAGKAYFGGDQIGLIDIALGGLSAFIKGLEKADPEKMPLLSAWMDRFFKSDGVKEVM
PDVTKQVEFIS
>PtaGSTU6
QVKLLGVTLSPFVVRVCIALALKGIYEFIQVNFMHPKSELLLKSNPVHKKVPVLIHNGVC
ESMIIVQYIEEAWENKPNLMPYYRAIARFWVAFVDDKLFPSQGEQLQKEVEVTVTNFLLI
EEALRAGKPYFGGDEIGLIDIALGGLSAFITGVEKADAEKMPLLSAWLDRFCQLDVVKEA
MPDPAEYIS
>PtaGSTU7
QVKLLGATLSPFVVRVRIALALKGIYEFIQETMQSKSELLLKSNPVHKKIPVLIHNGVCE
STVIVQYIEEAWGSKPNLMPYDRAVARFWAAFVDDKIFPSQGEQLQKAVEESVTNFLLIE
EALRAGKAYFGGDQIGLVDIALGGLSAFIKGLEKADPEKMPLLSAWMDRFCKSDGVREVM
PDFTKQAEFIS
>PtaGSTU8
QVKLLGASISPFVARVRIALALKGIYQFVEENLQNKSELLLQSNPIHKKIPVLIHNGVCE
SMIIVQYIEEAWGNKPNLTPYDRAIARFWAAFVDDKLVPCQGEQQEKAIQESVANFLILE
EALRSGKLYFGGDEIGFVDIALGGLSGFVKALQKADEEKMPLLSAWMDRFCEANGVKEVM
PDPAKYWNLYP

>PtaGSTU9
QVKLLGALWSPFVLRVRIALALKGIYEFIEDNLQPKSELLLKSNPVHKRIPVLIHNGVCE
SMIIVQYIEEAWNKPILMPYDRATARFWAAFIDDKLVPSQGEQQQKALEESVANLVVLEE
ALRSGKLYFGGDEIGFVDIALAGVSAFVKALEKILIDMPLLSAWIDRICEDNRVKEITLD
LAKVQESIA
>PtaGSTU10
QVKLLGGSTSPYVLRVRIALALKGIYEYIEENTQNKSELLLKSNPVHKKIPVLIHNGVCE
SMIIVQYIDETWDTRPFLMPYDRAIARFWAAFIDDKLLPCLGEQCQKAVEDTVANFLILE
EALRSGKAYFGGDGIGFVDIALGGTLAFVRALEKGDAEKMPLLSAWMDRFCEADGVKEVM
PDPVKLLEFIC
>PtaGSTU11
QVKLLGGRSSPFMLRVRIALALKGIYEHIKQTMNHTSELLLKSNPVHKKIPVLIHNCVCE
STIIVQYINETWDTRPFIMPYDRTIALWSAFIDDKLLPCQGEQYQKAAEEMVANFLIPEE
ALRSRKEYFRGDGIGFVDIALGGLLAFLKSLEKGGAEKMPLLSAWMDRFCEADGVKEVMP
DPVKLLEFIC
>PtaGSTU12
QVKLLGGRSSPFVLRVRIALALKGIYEHIEETMNPKSELLLKSNPVHKKIPVLVHNGICE
SMIIVQYIDETWDTRPFLMPYDRAIARFWAAFIDDKLLPCQGEQCQKAVEETVANFLILE
EALRLGKAYFGGDGIGFVDIALGGMLAFVNALEKADAEKMPLLSAWMDRFCEADGVKEVM
PDPVKLLEFIC
>PtaGSTU13
QVKLLGAAVSPFTARVRMALALKGIYEFIEEDIHNRSELLLKSNPVHKKIPVLIHSGVCE
SMIIVQYIEEAWGNKPNLMPYDRAIARFWVAFIEDKFAQSQGEQQQKAAEESVANFLLLE
EALSGKAYFGGDEIGLVDISLGGLLVFIKMLEKVDAERMPVLSAWVDRFSEADGVKDVLP
DRAKIFEFIS
>PtaGSTU14
QVKVLSLWASPFGLRVLVGLEEKGVYEYQEENLASKSELLLKMNPIHKKIPVLIHNDVLE
SLVILEYIDEAWPNTNPFMAYERARARFWADFVDKKIFDNCKGEAQEEAKRNMLEYLGLL
EGALDGGIKAYFGGKFGYMDIAFIPFASWFQAWEVMLETQFPRLHEWVNACMERESVKKV
LPPEKVAECAM
>PtaGSTU15
QVKLLGVIHSPFVVRVRIALALKGIYEFIEEEVLNNKSELLLQSNPVHKKIPVLIHNGVC
ESMIIVQYIEEAWGNKFNFMPYDRAIARFWAAFIDDKLTPSVGEEQQKAVEESVANLLLL
EEALSGKAYFGGDEIGLVDIALGGLLVGVQTIERVDTLKMPLLSTWAHKFCKAEEVKEVL
TDPAKLFELLS
>PtaGSTU16
QVKLLGVIQSPFVVRVRIALALKGIYEFIEEDICSNKSQLLVQSNPVHKKVPVLIHKGVC
ESIIIVQYIEEAWESKPFLMPYDRAIARFWAAFIDDKVIPSQGEEQQKAVEESVANLLLL
EEALRTGKAYFGGDGIGLVDIALGGPLVLIQTVEKVDTEKTPLLSAWADQFCKADKVKKA
LPDGAELFVLLS
>PtaGSTU17
GVKLLLIRSSPFAMSCALALRQKGVFEEVPENLSARSDLLLRSNPVYKQVPVLIHNGVSQ
SLVILEYIEEAWPPSDPSLLAYDRSLSRFWADFINKKFIETFGEEHATARKEIVEQFITL
EEGMRSVGPYFLGQKMSLADLALVPLVPWFTSFEALGPEQCGRMHEWLNAMREDPDVVAS
VPTADWLLNYTL
>PtaGSTU18
QVKLLGVTRSPFVARVRIALALKGIYEFIEEDIPNNKSELLLQSNPVHKKIPVLIHNGVC
ESMIIVQYIEEAWDSKPFLMPYDRAIARFWAAFIDDKVIPSQGEEQQKAGEESVENLLLL
EEVLRTGKAYFGGDGIGLVEIALGALLVLIQTVEKVDTQKSPLLSAWADQFCKADKVKNA
LPDRAELFVFLS
>PtaGSTU19
QVKLLGVIRSPFVVRVRIALALKGIYEFIEEDTPNNKSELLLQSNPVHKKIPVLIHNGVC
ESMIIVQYIEEAWDSKPFLMPYDRAIARFWAAFIDDKVIPSQGEEQQKTVEESVANLPLL
EEVLRTGKAYFGGDGIGLVDIALGGLLVLIQTVEKVDTQKTPLLSAWADQSCKADKVKNA
FPDRAELFVFLA
>PtaGSTU20
QVKLLGVIRSPFVVRVRIALALKGICEFIEEDVLNNKSELLLQSNPVHKKIPVLIHNGVC
ESMIIVQYIEEAWDSKPFLMPYDRAVARFWAAFIDDKVFPSQGEEQQRAGEESVENLLLL
EEVLRTGKAYFGGDGIGLVDIALGGFLVLTQTVEKVDTQKTPLLSAWADQFCKADKVKNA
FPDRAELFAFLS
>PtaGSTU21

QVKLLGWRRSPYVTRVRIALALKGIYEFIQEDSENKSQLLLQSNPIHKKVPVFFHNGLSE
SIIILEYIEEAWQTKPNLMPYDRALARFWAAFLDDKLLPSQGEEQQKAVKESLANFSLLE
KALRSGKAYFGGDEIGLVDIALGGALVHIKNLEKVDPVKLPLLSAWMAQFSETDELKQIL
PDPADVFEYLS
>PtaGSTU22
QVKLLGWLRSPCVTRVRIALALKGIYQFIEDDAENKSQLLLQSNPVHKKVPVLIHNGVCE
SMIIVQYIDETWDTKPNLLPYDRAIARFWAAFVDDKLASSQGEKQQKAVEESLANFLLLD
DALRSGNAYFGGGEIGFADIALGGGLVSLKAIQKVDPQKMPHLCAWIDRFFEIDLVKGIL
PDPVKVLERIS
>PtaGSTU23
QVKLLGWLRSPFVTRVRIALALKGIYQFIEDDAENKSQLLLQSNPVHKKVPVFIHNGVCE
SMIIVQYIDETWDTKPNLLPYDRAIARFWAAFVDDKLVLSQGEKQQKAVEESLANFLLLD
DALRSGNAYFRGVEIGFADIALGALLVTIKAIQKVDPQKMPHLCAWIDRFSEIDLVKGIM
PDPVKVLERVS
>PtaGSTU24
RVKVVKTWESPFAMRVLIALEEKGIYELQEENTRYKSQLPLQMDPIHKQIPVFLHNGLAE
SLIILQYIDEAWPSASPKRFMPYDRAIARFWAAFMDKKIYEATKGEAQEEAKRELVESLM
LIEGALKGGKTYFGGTLGFLDIAFIPLTGWFYTYETLLEDKCPCLKAWMKACMERDSVNR
LLPPEKILDYAI
>PtaGSTU25
QVKVLSQWPSGFCMRVLIGLEEKGVYEYQEENVLACKSELLLQTNPAHKGVPVLIHEGIC
ESVIILQYIDEVWPGSNSFMPYDRALGRFWADFMDTKFLENCTGEAQEEGTRYMLECLGL
LEGELSGGKPYFAGDQFGFLDIVLIPNSPWFYALETLWDGEFPRLQGWMKRCLERESVKK
ILQDPLKVSEYAI
>PtaGSTU26
EVKLLLIRSSPFAMSCALALREKGIFEEVHENSLPPKTDRLLRSNPVYKQVPVLIHNGVS
QSLVILEYIEEAWPPSDPSLLAYDRSLSRFWADFINKKFIETFGEEHATARKEIVEQFIT
MEEGMRSEGPYFLGDQMSLADVALVPLVPWLPSFEALGPEQCGRMHKWLDAMRELPNVVA
SVPPADWLSQYTA
>PtaGSTF1
AVKLYGPLASTATSRALACLLEKQVYQLIPVDLKKREQKKPGFLAQPFQVPVLQDGALTL
FESRAIVRYMAEKYAAQGTGLLLAERAVIEQWMEVESQTYNPPSFLVFQLAFAQLWGIPQ
TEIAKNEKKFSNALDVYEKRLSESKYLAGDEFSIADLSHLPNTEYIVKATGKAELITSRK
YVNAWWENISSRDSWKKIREMSEAGSKK
>PtaGSTF2
LIKVHGHHFSTATGMVLCCLNEKQVYDFVLVDLPTGAHKKPQYLANPFVVPTVQDEDLTL
FESRAIVKYLAKKFKGQGTELLLAEQALVEQWCQVEGQSFYPPSTILFQTVLFPLRGGTT
EAVVEINIGKLNRVLDIYEERLSKSKYLAGDFFSLADLQHLPWTQYLVTACKKGDLISSR
KHVSAWWEDISSRPAWKKVAEKMPK
>PtaGSTF3
AIKVHGFHASTATNMVLCCLNEKQVYDFVLIDLLTGAHKKPQYLANPFVVPTIQDGDLTL
FESRAILRYLAQKFKGQGTDLLLSEEALVYQWCEVEGQSFNPPSTIVSQILVVPRRGGST
EAAVEMNIEKLGKVLDIYEERLSKSKYLAGDFFSLADLQHLPHTHYLVSACGKGDLISSR
KHVNAWWEDISSRPTWQKVAENMKVN
>PtaGSTF4
TIKVLGYSGSITTRTVLCCLKEKRVYELVVVDPAAGDHLQPRYLANPFVVPTIQDGDLTL
FESRGILKYLAKKYKGQGTELYLSEEALVEQWCEVEGQSYNSPLAILSQIVFVPKKGGTT
EAVVELNVEKVGKLLDIYEERLSKSKYLAGDFFSLADLQHLATTHYLVTVCGKGDLISSR
KHVKAWWKDISSRPTWKKVTEKAP
>PtaGSTF5
TIKVLGYPLSTATRLVLCCLEEKQVYELVVVDAAAGAHKQPQYLANPFLVPTIQDGDLTL
FESRAIVKYLAKKYKGQGTELFLSEEALVEQWCEVEGQSYNSPLTILSQILFVPKKGGTT
EALVEMNVEKLVKVLDIYEERLSKSKYLAGDFFSLADLQHLPTTHYLVTDCGKGDLISSR
KHVKAWWEDISSRPAWKKVSEKGP
>PtaGSTF6
TIKVHGHHLSTATRLVLCCLEEKQVYELVVVDLAAGAHKKPQYLASPFVVPTIQDGDLTL
FESRAIVKYLAKKYKGQGTELFLSEEALVEQWCEVEGQSYNSPSAILFQILFVPMRGGTT
EAVVEMNVEKLVKVLDIYEERLSKSKYLAGDFFSLADLQHLPTTHYLVTACGKGDLISSR
KHVKAWWEDISCRPAWKKVSEKSS
>PtaGSTF7
KIKVHGLPFSSCTARVLACLNEKQVYELIPVNARAGDHKKPQYLSNPFLFPALEDGCLTL

FESRAIIKYLAKKYEGQGCNLLVEEEAVVEQWCEVESHHFNPPYALVSQIIINPLKGGTT
EAVVESNAEKLSRVLDVYEDRLSKSKYLAGDWFSLADLQHMPFLHYLVNDVGKATLISSR
KHVNAWWEDICSRPAWKKIAVHMKFPSK
>PtaGSTT1
KLRVYYVDRMSQPSRAVLIFCKVNKIFEEHLVNLGKKEHKQPEFRAINPLLVPAIHDGGFK
LFESHAILKYLACAYPSVPDWYLSKRAKIDSVLDWHHSNLRRGSAGLVLNRALAPALGLP
LNPQAASEAEILLKSSLSILETVWLSGNGTFLAGGYQPSVADLSLACEVMQLELLDKRDV
DILLGPHEKVRKWLEKVKESPHFEEVHEKIYKVSKRFR
>PtaGSTZ1
RLKLYSFWRSSCAWRVRIALNLKGLYEYKAVNLRQGEQFSEEFTKLNPIFVPTLVDGDII
VADSLAILLYLEDKFPGHPLLLQSKAISLQAAVLIGSNIQPLNMGVLNLIQEKLGPKEHQ
AWPKHFIEKGFTALEKLLKDVAGKYSVGDKLTLADIFLVPQVYNARSYNVDMSKFPTLNR
IDQALAELPEFQAAVPERQPDAKA
>PtaGSTZ2
RLKLYSYWRSSCSWRVRIALNLKGLYEYKAVNIVQGEQFSEEYTKLNPLFVPTLVDGDTI
VSDSLAILLYLEDKFPEHPLLHHLKAISLQAASIIGSNIQPLNLVILNLIEEKLGAEERL
AWPKPFIERGFTALEKLLKDVAGKYSVGDQLTLADIFLVPQVFGARRFNVDMSKFPTLNR
IDKELAELPEFQAALPARQPDAKA
>PtaGSTL1
TTRLYISVACPYAQRVWSARNIKGLQIQLVPIDLQDRPAWYKEVYPPKVPAIEHTGESLD
LLEYLENNFEGPKLFPAKKEAANELLKYTDTFTKNSFIALTKPDSETAQEAGPALDYLEN
ALGKFSDGPFLLGQFSVVDIAYGPFVERFHVAFPALKNYDITAGRPKLSKWIQELHKIEG
YAKTVSDP
>PtaGSTL2
TTRLYICVKCPYAQRAWSARNIKGLQIQIVPIDLQDRPVWYKEVYPPKVPALEHTGESMD
LLEYLDNNFEGPKLFPAEKEAANELLKHTDAFTKTVFVALTKPDSEAAQEAGPALDYLEN
ALGKFSDGPFLLGQFSVADIAYGPFVERFQVAFPALKNYDITAGRPKLLKWIQELHKIEG
YAKTTVADP
>PtaGSTL3
TIRLYINVLCPYAQRAWSARNIKGLEIQIVSIDLQDRPAWYKEVYPPKVPALEHTGESIA
LLEYLENNFEGPKLFPAKTEAATELLKHTDTFTQNLFGALTKPEPKAAQEAGPALDYLEN
ALGKFADGPFFLGQLSVVDIAYGPFFERFQVVFPALKNSAGRPKLLKWIQELHKIEGYAK
TVADP
>PtaDHAR1
PDKLGDCPFSQRVLLTLEEKQVYNMKFIDTSNKPDWFLQTNPEKVPVIKIIPDSDVITQI
LEEKYPEPPLAPPEKATVGSRIFSTFIGFLKSKDPNDGTEQALLNELRAFNEYLKDNGPF
INGEKISAADLSLAPKLYHLKVALGHFKKWSVPEEFTYVQNYMKALFSRESFQRTNAPDE
>PtaDHAR2
SPSKERGDCPFSQRVFLELEELGLYTATYIQEGPNKPHWFMVKNPSLMPVLRDIQDSDKI
AEYLDKHCAENTLKPVEFRDVGSKIFPIFTKWLQSKDPGSPCIYEFVEELVRFDRHLQKH
GPYIAGERPTDSDFALAPKLRHARVALAHFMDFQFPQELGALQNYMNRMESRESFEKTNY
PDE
>PtaTCHQD1
MQLYQHPYSLDSQKVRLALEEKGLYWPYSINPLKAKNLDSRIFMNPTKLPVFLNGQHIIL
DTLSILQYIDSIHEPLDREKMLLWMRSIDSWNPKLFTLSHVPQKYRLFFSRFQRRAAIAR
MAECPDLAGKYHLKLHSAYATEEELKDKELINSSNELLIKILDAAEDQLSSTDFLAGGAF
SMADAMFIPILARINLLNLEEEYIGSRSHLLDYWNRVKKRPSYHAVIGKYFSGWRKYK
>PtaEF1B?1
ALTLHAGSTNKNAFKALISAEYVGVVSLAPNFEMGGTKSPEFLMNPIKVPVLETPEGAVF
ESNAIARYVARLKGVNSLFDYAHVEQWIDFSTLEIDINITRWFYPRLGFGVYLPPAEEAT
IAGLKRSLAALNSHLATNTYLVGHSITLADIIMTCNLYLGFTSCMTKDFTKEFPHVERYF
WTMVNQPKFKKVMGEVKQTDAVPP
>PtaEF1B?2
ALTLHASSINKNGFKARITAEYAGVLNLAPNFVMGESNKSPEFLMNPIKIPVLETPEGAV
FESNVIARYVARLKGDNTLFDYAHVEQWIDFSSIEIDANVMRWYFPRIGSGVYLPPAEEA
AISDLKIGLLALNSYLATNTYLVGHSITLADIIVTCNLYQGFTRCMTKDFTKEFPHVERY
FWTMVNQPKFKKILGEVKQADAVPP
>OsGSTF1@NM_193840
PVKVFGPAQSTNVARVLLCLEEVGAYEVVNVDFTVMEHKSPEHLKNPFQIPAFQDGDLYL
FESRAIGKYILRKYKTREDLLLREAAMVDVWTEVETHQYNSASPIVYECIINPAGIPTQK
VVDESAEKLKKVLEVYEARLSQSTYLAGDFVSFADLNHFPYTFYFMGTPYASLFDSYPHV

KAWWERLMARPSVKKLAAVMAPQGA
>OsGSTF2@AF062403
PMKLYGSTLSWNVTRCVAVLEEAGAYEIVPLDFSKGEHKAPDHLANPFQVPALQDGDLFL
WESRAICKYVCRKNKELLLKESAMVDVWLEVESNQYTPANPILFQCLIRPMGAPPEKVVE
ENLEKLKKVLEVYEARLTKCKYLAGDYISVADLSHVAGTVCLGATPHASVLDAYPHVKAW
WTDLMARPSSQKVASLMKPPA
>OsGSTF3@AF309384
PVTVYGPMISPAVARVAACLLEKDVFQVEPVDMSKGEHKSPSFLKQPFQVPAFKDSLTTV
FESRAICRYICDQYADSGNTLMAVGRAAIEKWIEAEGQSFNPPLAMAFQLAFAPFMGRAM
AVVEQNEAKLVKVLDVYEQWLGENQYFAGDEFSLADLVHMPNTDLLVRKTNKAGLFTERK
NLAKWWDEVSARPSWKKVVELQNVPRPS
>OsGSTF4@AF309383
KLRVYGMALSANVVRVATVLNEKGLFDLVPVDLRTAAHKQPHFLANPFQIPVLQDGDEVL
YESRAINRYIATKYKAEGADLLASPAKLEVWLEVESHHFYPASGLVFQLLIKPLLGATTA
AVDEHAAALAQVLDVYDAHLAGSRYLAGNRFSLADANHMSYLLFLSKTPMAELVAFRPHV
KAWWDDISSRPAWKKTAAAIPFPPAA
>OsGSTF5@AF309382
MKVYGWVVSPWMARVLVALEEAGAYEVVPMSRSGGDHRRPEHLANPFEIPVLEDGDLTLY
QSRAIARYIFRKYKPEFLGLLEESAMVDVWLDVEAHQHEAARPILWHCIINKFGRDRQGV
VDESVRKLEKVLGVYEARLSGSRYLAGDRISLADLSHFSNMRYFMATEYAGVVDAYPHVK
AWWEALLARPTVQKVMAGMPPDFGFGS
>OsGSTF6@NM_197602
AVKVFGSPSSAEVARVLACLFEKDVFQLIRVDSFRGSKRMPQYLKQPHEALTFEDGNVTL
VESRKIIRHIADKYKNQGNDLIALERSSIEQWLQTEAQSFDVPADVVYSLAYLPAATTQP
VEEMKQLFEKSSKELSKVLDIYEQRLEEAEYLAGDKFTLADLSHLPNADRLAADPRTLRM
LQSRRNVSRWWADVSGRESWKQVKSLNRPPSAEAP
>OsGSTF7@NM_193830
PVKVFGRAISTNVSRVLVCLEEVGAYELVTVDFLAGEQNSPEHVENPFKIPALQDGDLVL
FESRAIAKYILRKYKSSKDLLIREAALVDVWTEVEAHQYYPASPIVFECIIFPIGVPTQQ
VVHESLEKLKKVLETYEARLSGSRYLAGDFLSFADLNHFPFTFYFMATPCASLFDAYPHV
KAWWEGLMSRPSIKKISANMPTKF
>OsGSTF8@NM_193836
PVKVFGPAMSTNVARVLVCLEEVGVYELVNIDFKAMEHKSPEHLKNPFQMPAFQDGDLLL
FESRAVGRYILRKYKTSENLLLTEAAMVDIGIEVEIHQYYPVSSIVYECLFNPAGVPTQK
VVDNSLEKLKKVLEVYEARLSQNTYLAGNFLSFVDLSHFPFTFYFMATPYASLLDKYPHV
KAWWDGLAARPSIKKVTAAMVLPLKA
>OsGSTF9@NM_193841
PVKVFGPAKSTAVARVLVCLEEVGAYELVGIHIPAGEQKSPAHLANPFQVPAFQDGDLIL
FGKISLGENLVSPLRKSADLLLSQSAIMVDVWLEVESQTFDTASAITFQCLTIPTGGIAD
KIVEENLGKLKKALEVYEARSCRFRYLAGDFISLADLSHFPMTHYLLATPHASVLDAYPH
VKSWINDLMKRPAVKRVRELMEA
>OsGSTF10@NM_193842
PAKVYGPAMSTNVMRILVCLEEVGAYEVVPVDMSTGEHKRPPHISNPFQVPAFEDGDLTL
FESRAISKYILRKHGDLLLSESAMVDVWLEVESSHFDGASPIIFQCFIVPMGGATIGVVN
ESLEKLKKALEVYEAQLSKSKYLAGDFISLADISHFPTVYYLLASAHASVLEAYPRVKAW
IDDVMQRPSVKKVTEALKMPSA
>OsGSTF11@NM_193851
PVKVFGSAPFTNVARVLLCLEEVGAYEIVDVDFGDREHKGPDHLANPFQVPAFQDGDLML
FESRAICRYILRKHRATDENLLPSESAVVDAWLDVEALRYEPSHAVFVQRRVVPAGGEPE
RVIAESVARLRETLAVYEARLEATRGYLAGGEVSLADLSHFPYTRYFMEMPYEVPVFGAY
PRVTAWWERLLTRPSVRKVAAMMSGGEG
>OsGSTF12@NM_193860
AMKVYGLPMSTNVARVLVCLEEAGEYEVVPIDFSIAEHKSPEHTSNPFQVPALQDGDLIL
FESRAISKYVLRKNNELLLSDAAKVDVWLEAESHHFDEPSVVIYQCLILPVGGQTAKVVE
ENLEKLKKTFQVYEERLCKFRYLAGDFLSLADLSHFPTAYYLLATPHAAMLDEFPLVKAW
IDGMLARPSVKKVIEMMKATA
>OsGSTF14@AC099399-OJ1006F06.9
SVKVFGSPTSAEVARVLMCLFEKDVFQLVRVDAYRGTQRMPQYLKQPLEALTFEDDNLTL
SESRGILRHIAHKYARQGNDLIALERASIEQWLQTEAQSFDVPAEMVYSLAFLPPNMPKQ
ANGNKQQQKEEEMRKVFEKSKKDLEKLLDIYEQRLEEAAYLAGDKFTIADLSHLPNADRL
ASDPRSRRMFEARKNVSRWWNNISSRESWEYVKSLQRPPSAAHA

>OsGSTF15@AY533123-OJ1006F06.5
GLQVFGQPASTDVARVLTCLFEKNLFELIRIDTFKKEHKLPEFIKRDPTQVTFKHGDKTL
VDSRAICRYLSTQFPDDGNTIYSLERASIEQWLQAEAQSFDAPSELVFHLAFAPQLNIPE
ARIAENERKLQQMLNVYDEILAKNKYLAGDEFTLADLSHLPNSHYIVNARSPRGKKLFTS
KKHVARWYEEISNRASWKQVVKMQSEHPGAFE
>OsGSTF16@OJ1006F06.6
GLQVFGQPASTDVARVLTCLFEKDLFELVCIDTFKREHKLPEFIKRVRYYLFSRAICRYV
CTQFPEGNTLYSLERASIEQWLQAEAQNFSPPSALVFHLAFAPHLNIPHAVIAENEKKLQ
QVLNVYDEILSKNEYLAGDEFTLADLSHLPNSHYIVSSERGRKLFTGRKNVARWYDQISK
RETWKQVVKMQREHPGAFE
>OsGSTT1@AF402793
LLKVYADRRSQPSRAIIIFCRVNRIFEEVTVDLFKREHLSPEFKKINPMQVPAIVDGRFR
LFESHAILRYLATVFPGVADWYLFTRAKLEAILDWHHSNLRRGAATFILNTVLAPSLGLP
SSPQAAKEAEKVLFRSLGLIESMWLKGNAKFLLGNPQLSIADLSLVCEIMQLEVLGDSER
DRILGPHEKIRSWVQNVKKSPHFDEVHELIFKMKERMA
>OsGSTT2@AY541762
LKSLYGLKQHGGGEGVILCLYVNESYVEKILNRFGYIDSKPSPTYDPSLLLRKNKRIARN
QGSLMYLASATRPDISAVRFTSSPGDDHWRALERVMRYLKATFILNTVLAPSLGLPSSPQ
AAKEAEKVLFRLIESMWLKGNAKFLLGNPQLSMADLSLVCEIMQLEVLSDSERDRILGPH
EKIRGWVQNVKKSPHFDEVHELILKVKTRTA
>OsGSTU1@AF050102
ELVLLDFWVSPFGQRCRIAMAEKGLFEYREEDLGNKSDLLLRSNPVHRKIPVLLHAGVSE
SLVILQYLDDAFPGTPHLLPAFARATARFWADYVDRKLYDCLKGEPHAAAGREMAEILRT
LEAELGDREFFGGRLGFVDVALVPFTACSTATERCEEVAPRLAAWARRRGRIDSVVKHLP
PEKVYDFVG
>OsGSTU2@OSJNBa0034L04.3
ELKLLGTWASPYVSRVKLALHLKGLSYEYVVEEDHFNNKSELLLSSNPVHKKVPVMIHNG
ICESLIIMEYLDEAFPDTPLLLHDRAVARFWAAYIDDKLVKTGEEKAEGMRHMLAAVDAL
EAAMEKGKPFFGGDAVGFLDVALGGLLSWLHGTEELDAAKTPLLSAWARRFGEMDAAKVA
LPDVCKLVEFAK
>OsGSTU3@AF309379
ELKLLGMWASPFALRAKLALSFKGLSYEYVEEDLKNKSELLLTSNPVHKKVPVLIHNGVC
ESQVIVQYIDEAFPDAPLLPYDRAVARFWAAYIDDKLLKTEQEKAAAMKETFAAVANLEA
AFKKGKPFFGGDAVGYVDVTLGAVIGFVRVGEAVDASRSPLLDAWLDRFAALDAAKAVLP
DTGRLAEYAK
>OsGSTU4@AF309378
ELMLLGKWPSPFVTRVELALGLKGLSYEYVKQDLVNKSELLLASNPVHKKIPVLIHNGVC
ESSIIVQYIDEAFPDAAALLPYERAVARFWVAYVDDKFVKTEEEKAEGMKQLLAAVETLE
GALKKGKPFFGGDTVGIVDVALGGLISWVKATEVLDEEKAPLLAAWAQRFGELDVAEKVL
PDVDGVVEFAK
>OsGSTU5@AF309377
EVVLLDLWVSPFGQRCRIALAEKGVYEYSEQSLADKSDLLLRSNPVHKKVPVLLHAGVCE
SLVILEYIDETWPPEPEPRLLPYARARARFWADYVDKKLFDCLAGDAAHEQAKRDMAEAL
GTLEAELGEGDYFGGAFGYLDVVLVPFVAWFHAYERLAEICPRLVAWGERCKGRDSVAKT
LTPEKVYEFAL
>OsGSTU6@AF309376
ELKLLGVWSSPYAIRVRVVLNLKSLPYEYVEENLGDKSDLLLASNPVHKSVPVLLHAGVN
ESQVIVQYIDEVWPGGPSVMPYERAVARFWAAYVDDKVRPAWLSKTEEERAAAVAQAVAA
LETLEGAFGKGKPFFGGDGVGFVDVVLGGYLGWFTAIDKLDPARTPALAAWEERFRATDA
AKGVVPDADKLLEFRQ
>OsGSTU7@AF402794
ELKLLGLWASPYVLRAKFALSFKGLSYENVEEDLHNKSELLLSSNPVHKKVPVLIHNGIC
ESQIIVEYVDEAFPDASLLPYDRAVARFWAAYINDKFMLTEKKKAKAVKQMLAAIENLET
AFKKGKPFFGGDTAGYLDVTLGAVVGWARAGEVLDATRSPLLAAWMERFVALDAVKAVLP
DNAELIEYGK
>OsGSTU8@AF402795
ELKLLGHWSSAYVTRVKLALHLKGVSYEYVEEDLRNKSDLLLASNPVHKTVPVLIHNGIR
ESQIIVQYIDEVFSGASILPYERAVARFWAAYIDDKLLKTEEERAAWMKQMFVAVDVLEG
GLKKGKGCFFGGDSVGYVDVVLGGAVSFVHANDMIDAARTPLLAAWLERFGELDAAKAVL
QDVDRAVEHTK
>OsGSTU9@AF402796

ELKLLGMWASPYVSRAKLALQLKGVSYEYIEEDLGNKSDLFLRSNPVHKTVPVLIHNGIC
ESSIIVQYIDESFPSSSLLPYDRAVARFWAAYIDDKLAKTEEERDELMKQTLAAVDVLEG
GLKKGKGCFFGGDSVGYVDVVLGGLVSWVHASDKLDAAKAPLLAAWLGRFGELDAAKAVL
QDVDKVVEYAK
>OsGSTU10@AE016959
ELKLLGMWTSAFVLRVRFVLNLKSLPYEFVEENLGDKSDLLLASNPVNKTVPVLLHAGVN
ESQVILQYIDEAWPDRPAVLPYERAVARFWAAYVDDKVRLAWLSETEEERAAAVAQADAA
LETLEGALRGGKPFFGGDGVGLVDVVLGGYLGWFTAIKKLDPARTPALAAWEDLFRATDA
ARGVLPDADKMLEFRQ
>OsGSTU11@AF402798
ELKLLGTWASPFVQRVRLALNLKGLYEFIEEEIGGGKSELLLSNPVHKVPVLLHICESQV
IVQYLDDRARVRGAEGLLRRGQAGVRRRGARRVRGLDARVRHGGRRGARRRAHPAAGGVG
GARRGARRRQGRHPRRRTRRRALEEEVDTVTICVVLWCLYRQNKCGTKVVIISEHSCRFL
LQFKCLCCFCLDCGDVRIYGS
>OsGSTU12@AF402799
ELKLLGMWASPYVLRVKLALSLKGLDYEYVEEDLKNKSELLLSSNPVNKKVPVLIHNGVC
ESQIILQYLDEAFPDATLLPHERAVARFWAAFCDDTIAKTEEEKAEGEKKVVEALEKMEV
GLSKGKPFFGGDTVGYVDIVLGSFLAWVRAGDAMDPATTPLLAAWAERFVELDVAKAAMP
EVDKLIELAM
>OsGSTU13@AF402800
DVKVLGVVVSPFAIRVRIALNIKGVSYEYVEEDIFNKSELLLTSNPVHKKVPVLIHGGIS
ESLVIVQYVDEVWAAAPSVLPYDRAVARFWAAYVDNNMFATEEERAAKAEETLAALAQLE
KAFACAGGKAFFGGDSIGYVDLALGSNLHWFEALRRLDAGKTPLLAAWAKRFVEAEAAKG
VVPDAGVAVELGK
>OsGSTU14@AF402801
QLKLLGLWVSPYTHRVKLALSFKGLSYEYVEEDLSNKSELLLSTNPVHKKVPVLIHNGIC
ESQVIVQYLDEEFPNSSLLSYDRAIARFWAAYINDKLMKTEEERAEALKQTLEAVANLET
AFKKGKPFFGGDTVGYLDVSLGAMIGWMRAGEALDATRSPLLNAWMERFAALDAAKAAMP
DNNKLVEFVR
>OsGSTU15@AF402802
AVRVLGGWASPFTNRVVVALKLKGVHEMLQETVGKKSELLLRSNPVHKKFPVLLHHSLPE
SLVIVEYIDEVWPASNGPAILPHGRAVERFWARYVDDKILPGSVAGDKDQTAGEMSTTLQ
RLEEAFVQGKEYFGGDSIGYLDIALGSFLGWIKAVEKINETKLPILAVWADRFCAHPAVV
DVVPDADKLVEFTV
>OsGSTU16@P0017D10.7
ELKLFGSWASSYTHRVQLALRLKALFVYAEEDLGNKSEALLRLNPVHKKVPVLVHRGLAE
SVIILQYLDDAWPERPLLPFDRALARFWCHFADDKLLTGREQEAAVQQVHDNLALLEAEL
RKGRRFFGGDQVGLLDVVLGCGSYWLAVFEEVDADAFPLFHAWLRDFEAQEEVKETIPSV
DRLLEYAR
>OsGSTU17@AF402804
GVKVFGMWASPMAIRVEWALRLKGVYEYVDEDLANKSEALLRHNPVTKKVPVLVHDGLAE
STVIVEYIDEAWKHGYPIMPFDRAQARFWARFAEEKCNAATGEEQRKLVHEAQQCLKTLE
TALEKKFFGGDAFGYLDIVTGWFAYWLPVIEEATDEALPLMKAWFDRVLAVDAVKAVLPP
RDKLVALNK
>OsGSTU18@AF402805
ELKLLGLWASPYVLRAKFALSFKGLSYENVEEDLHNKSELLLSSNPVHKKVPVLIHNGIC
ESQIIVEYVDEAFPDASLLPYDRAVARFWAAYINDKFMLTEEEKAEAVKQMLAAIENLET
AFKKGKPFFGGDTAGYLDVTLGAVVGWARAGEVLDATRSPLLAAWMERFVALDAVKAVLP
DNAELIEYGK
>OsGSTU19@NM_197460
ELKLLGMWASPFALRAKLALSFKGLSYDYVEEDFKNKSDVLLSSNPVHKKVPVLIHKGIC
ESQVIVQYIDEVFPDATLLPHDRAVARFWAAYIDEKLFKTEEEKAEAVKQTFAVVEKLEG
ALSKGKPFFGGDTVGYVDVVLGGFVAWVHAIEEVDAAKTPLLAAWLERFDELDAVKEVMP
DIGRLVELAK
>OsGSTU20@NM_197461
DLKMLGVYVSPFPLRVKLALSFKGLSFEYVEEDLHNKSDLLVSSNPVHKRTPVVIHNGIS
ESMVIVQYLDEAFPGAALLPLDRAVARFWASYVDDKLFKTEEEKVEGRKQTFAVAETLEG
ALRKGKPFFGGDAVGYVDVALGGFVPWVHAMEELDAAKTPLLAAWLERVGELEAYKAVMP
DAGMMIEFKK
>OsGSTU21@NM_197444
AVRVLGSWTSPFVMRVVVALKLKGVEYELLQETRGKKSELLLRSNPVHKKIPVLLHHGLA

ESLIIVEYIDEVWPASDGPAILPYCRAVERFWAQYIDDKPDDFVSSGDSVERNRGRRGTV
VEMSTALKHLEEAFVQGKKYFGGDKIGYLDIALGSFLGWIKAVEKFDEAKVPNLAAWADR
FCAHPAVVDAMPDADKLVEFAV
>OsGSTU22@NM_197445
ELKLLGMWASPFALRAKLALSFKGLSYDYVEEDFKNKSELLLSSNPVHKKVPVLIHNGIC
ESQVIVQYIDEVFPDATLLPHDRAVARFWASYIDEKLFKTEEEKAEGVKQTFAVAEKLEG
ALSKGKPFFGGDTVGYVDVVLGGFVAWVHAIEEVDAAKTPLLAAWLERFDELDAAKEAMP
DIGRLVELEK
>OsGSTU23@NM_197496
DVKVLGLVMSPFAIRVCIALKLKGVSYEYIEEDLANKSELLLSSNPVHKKIPVLIHGGVS
ESLVIVQYVDEAWAPSPSILPYDRAVARFWAAYVDDKMVATEEERAAKADETLAAMAQLE
KAFAAKNGKPFFGGDTVGYVDLALGCNLHFLEAIRRLDAGKTPLLAAWAERFVEVEAAKG
VVPDADDAVEFAR
>OsGSTU24@NM_197473
ELKLLGMWASPYVLRVKFALSLKGLSYEYVEEDLMNKSDLLLSSNPVNKKVPVLIHNGVC
ESQVILQYLDEAFPGATLLPHERAVARFWAAFNDDTLVKTEEERAEGEKKVVEALEKMEV
GLRKGKPFFGGDTVGYLDVVLGGFLAWVRATDVMDPATTPLLAAWAERFVELDAAKAVMP
DMDKMIEFGK
>OsGSTU25@NM_197490
ELKLLGFWASPYVCRVKLALHLKGLIYDYVKEDVFTNKSELLLSCNPVHAKVPVLIHNGI
CESQVIVQYIDEVFPDATLLPHDRAAARFWAAYIDDKLLKTDEEKAERMKQTLAVVDALE
TAMEKGNAFFGGDTVGYVDVALGGLLSWLHGTEELDAAKTPLLSAWARRFGELDAANAAL
PDVGRLVEFCK
>OsGSTU26@NM_197493
ELKLLGTWFSPFVSRVKFVFHLKGLSYENIEEDLKNKSELLLKSNPAIKKVPVLFHNGLC
ESMIIVEYIDETFAGVSVVAYERAVARFWVSYIDNKLVKSMEEKAEGLKQIFVAVMVLEE
AFKKGRPFFGGDNAGIVDIALGSQLGWVRASQALDPAKTPLLAAWAERFLALDAAKASMP
EFGRLIEYAK
>OsGSTU27@NM_197492
ELKLLATWFSPFASRVKFVFHLKGLSYENIEEDLKNKSELLLKSNPVIKKVPVLLHNGLC
ESMVIVEYLDETFAAVSVVPYERAVARFWVSYIDNKLVKTKEEKAEGLKQMFEATAVMEV
AFRKGRPFFGGDAVGIVDVALGSQLGWLRASETLDPAKTPLLLAWAERFLALDAAKASMP
ESGRLLAYAK
>OsGSTU28@NM_197495
ELKLLGTWPSPFVVRVRLALGLKGLSYEYVEQDIRDKSELLVVSNPVHKKVPVLIHGGVC
ESQIIVQYIDEAFPGASLLPHERAVARFWATYIDDEFAAKEEEEKDEAAAQVFAALETLE
EAMKGKVFFGGDSAGYVDVALGGFLGWIKAAEALDGARTPLLAAWAARFSALEAAKEAIP
SVERLREFHG
>OsGSTU29@NM_197474
ELKLLGMWTSPFALRVKLALSFKGLSYEYAEEDLSNKSELLLSSNPVHKKVPVLIHNGIC
ESQVIVQYIDEAFPGAPLLPYERAVARFWAAYIDDKLLKTEEEKAEALKETFTAVANLEA
AFEKGKPFFGGDAVGYLDVTLGALVSWVHAGEALDATRSPLLDAWVDRFAALDAAMAVLP
DAGTLAEYAK
>OsGSTU30@NM_197486
ELKLLGHWASAYVTRVKLALHLKGVSYEYVEEDLRNKSDLLLASNPVHKTVPVLIHNGIR
ESQIIVQYIDEAFSGASLLPHERAVARFWTAYIEDKLVKTEEERAAWMKQMFVAVEALEG
GLKKGKGCFFGGDSVGYVDVVLGGGVSFVHANDVIDAAKTPLLAEWLGRFGELDAAKAVL
QDVDRAVEYTK
>OsGSTU31@AY533124-CI241065
AVRVVGGWASPFMNRVVVALKLKGVHEMLQETVGKKSELLLRSNPVHKKIPVLLHHGIAE
SLIIYIDEVWPASNGPSILPYGRAVERFWAKYIDDKIPPGSVEEDKDKAAGEMSTALQHL
EEAFVQGKQYFGGDNIGYLDIALGSFLGWIRAVEKINETKVPNLAAWADRFCAHPAVVDV
VPDADRLVEFTV
>OsGSTU32@NM_197036
MRVMVALRLKGVYELLQETMGKKSELLLASNPVHKKIPVLLHRGISESLVIVQYVDEVWP
PPSILPPYAAAIHRFWGQYIDDMVSTLRIRILGTVPGDKDEASDEMTTALLYLEEAFVKG
KQYFGDDSIGYLDIALGSHLGWIRAVERIGGAKVPNLAAWADRFCGHPAVVDVMPDVDIL
VEFTA
>OsGSTU33@NM_196067
ELKLLGSTNPSPFVTRVELALALRGLTYDLVAVDLDRKTDLLLAANPVHAKVPVLIHRGV
CESRVILEYIDDAFPFPAPLLPLARAAARFWAAHVDDEFVSTEGERAEGMARMAAAVGAL

EGALAEGKPFFGGDAPGLVDVTLGSVIPRTRANEALDAARTPLLAAWAERFGELDAARKV
LPAVGDVVEYLE
>OsGSTU34@NM_196328
GVRLLGGRMSPFTMRARMALALRGVYELVEEALHPRKSGRLLAANPAYGRIPVLLLPGGV
CESAVIAQYVDDAWGGAGAAILPYERAMHRFWTAYIDDKFWPAPTPEARATATASTRAAL
KLLEEAFANGGAFFSGGSPGLLDVALGCFLPALWACENLDDDATPLLRAWSARLAATPAA
MAVMPETEEVVAFTR
>OsGSTU35@NM_196091
ELKLLGTWPSPFVTRVELALALKGLSQDLVGMSDLLLASNPVHKKVPVLIHNGICESRII
LEYIDEVFPVDALLPYDWAVARFWAAYIDDKAMCPFAITHAMADNVHAKTEEEKAEGIKQ
ILAAVETLEGALKKEKPFFGGGTVGLVDIMLGAHIPGVRATEVLNAAITPLLASWTERFG
ELDAPKKVLPDVDGMVEYVK
>OsGSTU38@AY541763
EVKLYGAWGSAHAAMARNALELKGVYEYVEEDLERKSETLLLLNPAHAKVPVLVVVDDDL
AESLVILEYVDEVWPQAPRLPPPRARAAARFWARFFHGEVSPLSRAAVLAPTPEERAEAV
REMKARMAGFERDFPSSVVGGPFVHGATPGLLDVILGSCAAGTRAISAMAGEEVVEPDAL
PHVHASMAAFDERVAGFGT
>OsGSTU39@NM_192151
ELRLLGTWSSPWVIRVRVALGMKGLSYEYTEEDLSSKSDHLLRSNPVHEKVPVLIHGGVC
ESLVVLEYIDETWGATPQLLPYDRATARFWTNYVNDTFFTAAEQRAEAFKNVVPRVEALE
RAFGKGKAFFGGDDAGLVDVALGSHLVWIKVVDEVDEAKFPGLAAWAERFLAVDAVRQVM
PDAGDVLKQYK
>OsGSTU40@NM_192150
ELQLLGTWYSPYAMRAKIALGLKGLSYEYIEQDLFGKSELLLKSNPVHKKVPVLIHAGVC
ESRVVLEYVDEAWPGAPLLPHDRATARFWATYFDSTFFTTAEQRAEAFMNAVPQVEVLER
AFVKGKAFFGGDAVGLVDVVVGGFVVWFKVVDEVDEAKFPGLAAWAERFLAVDAVREAMP
DAGKLLEHYK
>OsGSTZ1@AF309381
KPVLYSEWMSSCSYRVRIALNLKGIYEYRAVTRGDPDYGKINPIYVPALVDGDFTISDSL
AIILYLEDKYPQHPLLLKKKALNMQIANIVCSSIQPLCYAVIGLADGKMSANESLQIVQH
YTDKGFRAIEKLLEGCRSKYATGDEVQLADVFLAPQIHAGITRFQIDMSKYPILARFYKA
YMELPAFQAAVPENQPDAPSS
>OsGSTZ2@AF402792
KPILYGAWISSCSHRIRIVLNLKGVYEYKSVNPRTDPDYEKINPIYIPALVDGDLVVSDS
LAIALYLEDKYPQHALLLKKKALNLQIANIVCSSIQPLGYAVIGLHEGKLSPDESLQIVQ
HYIDKGFKAIEKLLEGSNFKYATGDEVQLGDVFLAPQIHAGINRFQIDITKYPNLARLHD
TYMEIPAFQAALPKNQPDAPSC
>OsGSTZ3@AK073086
KLGLYSYWRSSCSHRVRIALNLKGLYEYKAVNLLKGEHSDPEFMKVNPMFVPALVDGDAV
IGDSYAIALYLEDKYPEHPLLLKMKALNLQIASIVCSGIQPLNLTVLVRTDLHRFIEKKV
GTGESIPWTQQQIDRGFAAAENLVKGCAGKYATGDEVRLADVFLAPQIYAAVTRFQINML
NYPTLARLHEEYMKHPAFQAALPDRQPDAPSST
>OsGSTZ3@AY538745
KLGLYSYWRSSCSHRVRIALNLKGLYEYKAVNLLKGEHSDPEFMKVNPMFVPALVDGDAV
IGDSYAIALYLEDKYPEHPLLLKMKALNLQIASIVCSGIQPLNLTVLRFIEKKVGTGESI
PWTQQQIDRGFADLVAAENLVKGCAGKYATGDEVRLADVFLAPQIYAAVTRFQINMLNYP
TLARLHEEYMKHPAFQAALPDRQPDAPSST
>XP_004229524.1_SlGSTU1
EVKIYRNWSSPYGLRVVWALDIKGIYENIFEDLSQKSPQLLQYNPVHKKIPVLVHKGICE
SLVILEYIDETWKETTPLLPYEKAIARFWAKFVDDKLLPSRYEAKKEALVPAMQNLEFIE
EQLKKKFFGGESIGYVDLALGWMAYLLDVFEEVDADKFPLLSGWTKNFCDAPAIKQHLPP
RDKLVTKFQ
>Solyc01g081260.1.1_SlGSTU2
EVKLYRTWSSRFSLRIIWALHIKGIYEAIFEDLSHKSPQLLKYNHVHKKFPVLVHNDICE
SLVILEYIDETWKETSHLLPYEKAMARFWAKFVDDKFKNLGPKQNFIIN
>Solyc01g081270.2.1_SlGSTU3
EVKLYRTWSSPFGLRIVWALHIKGIYEAIFEDLSQKSPQLLQYNPVHKKIPVLVHKGICE
SLVILEYIDETWKETAPLLPYEKAMARFWAKFVEDKLLPSGYDAKKEAFVPAVQNLEIIE
EQLKKKFFGGESIGYVDLVLGWMAYLLDVFEEVDADKFPLLSGWMKNFCDAPAIKQHLPP
RDKLVTKFQ
>Solyc01g081310.2.1_SlGSTU4

KVKLLGYWASPFALKVHWALKLKGIYEYQEEDLSNKSPLLLQYNPVHKKIPVLVHNGIAE
SLVILEYIEETWKHNPLLPYERAKARFWAKFVDDKCVPGGVEQQKIAKEARENLKILEDE
LGKHFFGDAKIGFMDVTSAWIICWAQIVEEVDAEEMPSLVSWFQNVLEAPILKECTPPKD
KLLEHNK
>Solyc01g086680.2.1_SlGSTU5
EVKVHGIFAGPFNKRVELALKLKGVYEYIEEDRSNKSDELVKYNPIYKQVPVLVHNGICE
SIIILEYIDDTWENNIPLLPYQRSMARFLAKLIDEKLMGAKGEEREKGCDETFEVLKYLD
NELQKKFFGGDSIGFVDIVASYIALWFGAIQEATEQKFPKLSKWIDEFLCCRIVKENLPN
REVLVPLYK
>Solyc01g099590.2.1_SlGSTU6
DVKLLGTKESIFTQRIIWALKLKGIYEFIEQDFSSRSSPLLVKLNPVYNKVPVIVHDGLA
ESLVILEYIEETWPLINPLFPFQRASTRFWARFVDGKFYEASGETKAEGVESVVEGLHLL
EGQIIGKKFFGGEKIGYLDIITGWIAYWFQYIEEIDSTKYPCLHAWINNFIQLPIIKQSL
PTPDVVKSVFR
>Solyc02g081240.1.1_SlGSTU7
ELRLLDFWASPFCMRVKIALSEKGVYESQQEDLFGGKSDMLLKSNPIYEKVPVLLDNGIV
ESNNIVYYIEDKYPSTNLLAYGRSRARFWADFIDKKIFEGSKGEELEIAKKDFIEILKKL
EGAMGDKDFFGGNFGYVDVIAIAMTSWFHAYEVFEQECPKFGCWMKRCLERESVSSVLPP
EKIYQCVV
>Solyc03g116120.1.1_SlGSTU8
KVTLHGMWLSPYVKRVELALKVKGIFEYIEEDLSNKSPLILKYNPIHKKVPILVHNGVNE
SFVIVEYIDETWKNPQLLPYERSKVHFWAAYIQQVMESMEDQKQACNEFHQKFRLLEDGM
KPTIENRNIGLIDIWIVVAFGMCKAQEEADPEKVPLIHSRVNSLLELPLLRETVPDHDKA
VSFLR
>Solyc03g116130.1.1_SlGSTU9
RVTLHGMWISTYAKKVELALKIKGIFDYVEEDLSNKSSLLLKYNPIHKKVPLLLHRGLSE
SLVILEYIDETWNNLPLLLPYERATVRLWASYCLQISDTMARDVEGGAFDELFEILKVME
EGMKPGGRSKICAENLGLLDIIIVCSLATYKAAEEVDPEKNPFVYSWVTTLLELPLVKET
LPPHDKLVSRLD
>Solyc05g006730.2.1_SlGSTU10
NLKILGAWPSPYVMRPRIALNIKCLAYDFLEEQFGTKSELLLKSNPIYKKIPVLIHDGIC
ESLIIVQYIDENWTNFSILPYDRAIARFWAFYIDDKWFQDEDAKKAAMETVIEGLVLLED
VFKKGKKFFGGDKIGYLDIALGCFLGWLKVNEKLDESRTPSLYQWAKDFCVDSVVKDVMP
ETDQLVQAAK
>Solyc05g006740.2.1_SlGSTU11
MRPRIALNVKSVCYDFLEEQLSSKSDLLLISNPVYKKIPVLIHDGICESLNIVQYIDEKW
TNSSILPYDRAIARFWACYIDDKWFQGEDAIKTALEPVFDGLVLLEDAFKKGKKFFGGDK
IGYVDIALGCFLGWMRVIEKMDEAKTPGLYNWAEDFCADSSVKDVMPETNKLAEAAK
>Solyc05g006750.2.1_SlGSTU12
SVKLLGTWACPYVYRVEIALKMKSIYEFIQERVFNKSELLLKSNPVYKKIPVLFHDEICE
SLVILQYIDEAWLNPAILPYDRAIARFWAAYIDEKWYPLEGKEAKAAMVEKISEGTLLLE
EAFIKGKSYFGGDSIGYVDIVFGSLLGWVKVIEIVDETKTPSLAEWDEKFCSHNVVKDII
PETEKLVEIYH
>Solyc05g026210.1.1_SlGSTU13
IIKVLGTPASPFANRVSIALNVKSVYEFVQEDMSNKSELLLKSNPVYKKIPVLILGEICE
SLVIVQYIDETWTNPSVLPLDRAITRFWVAYIDCKWLPLQGEEAILEVQEKLQQALVPLE
EAFVKGKSFFGGENIGYIDIALWCILGWIKAIKIMNVTKAPELVNWGNRFLEDKCVKGAM
LEPEKLVEIVK
>Solyc05g026220.1.1_SlGSTU14
MNIFKVNGWTCTPPLQLSLKIKSIYEFIQEHILNKSELLLKSNPVNKKIPILFHDEICES
LVILQNIDEPWLNPSILPYNRSIARFWAAYIDDKEYRNEGKEAKAVVVDKMSEGNMLLEE
TFIKGKSFFSGDSIGYVDIVLGSLLGWVR
>Solyc06g069040.2.1_SlGSTU15
QVKLIGSSGSLFCTRVEWALKLKGVYEYIQEDLLNKSELLIKSNPVHKKIPVLLHDDVVE
SLLILEYIDETWKGYPLLPHERATARFWAKFVDDKCVIGEGEAKTKAIESIQELYAFIEK
QIEKKFFGGEQIGYLDLVMGWKTLWLSAMEEVDPEKFPSLHQWAENFKQIPIINECMPQQ
ETLVNYFQ
>Solyc07g021460.1.1_SlGSTU16
MKEENTLHGMWAKSTNICESSVIIEYIDETWKNESPLFPYQRIKVRFWASYIHQVYDCML
KVFRGKEALEGFYAKLSVLEDGINNFSLGITSNMNNIGMLDIMIVITLGAYRVQEEVEEE
NSLLYSWVTTLIELPIVKGITPPHEKVVSFL

>Solyc07g049330.1.1_SlGSTU17
MTGKGEDQEEAKELIEIFKTLEGELGDKTYFGGKLGFVDVTCANFEAECPKLVAWAKRCM
EIENVSNSLTPHKIYGYVL
>Solyc07g056420.2.1_SlGSTU18
EVILLDFWPSMYGMRLRIALAEKEIYEYRDEDLRNKSPLLLQMNPIHKKIPVLIHNGICE
SIIGVEYIDEVWKDKALLPYERAQARFWADYIDKKLYATATGDEQEAGKKDFVEILKVLE
GALGEKPYYGGNFGFGDIALIGFYCWFHAYEVYEAECPNLVAWAKRCMQRDSVAKTLPQH
KIIEFVK
>Solyc07g056430.2.1_SlGSTU19
MNPIHKKIPVLIHNGICESIIGVEYIDEVWKDKALLPYERAQARFWADYIDKKLYRSTKG
EEQEAGKKDFIEVKVLEGALGEKPYFGGNFGFVDIALIGFYSWFHSYETYEAECPKFVYH
QF
>Solyc07g056440.2.1_SlGSTU20
ELILLDFWASMFGMRLRIALAEKGIYEYKEEEGLISNKSALLLEMNPIHKKVPVLIHNGI
CESIIGVEYIEEVWKDKALLPYERAQARFWVDYIDKKLYVSTKGEEQEAGKKDFIEVLKV
LEGELGEKPYFGGNFGFVDIALIGFYSWFYAYETYEAECPKFVAWAKRCMQRDSVAKSLP
QHKVLEFIQ
>Solyc07g056450.2.1_SlGSTU21
EVILLDFWPSMFGMRLRIALAEKEVYEYKEEDVWNKSPLLLEMNPIYKKVPVLIHNGICE
SIIGVEYIEEVWKDKALLPYERAQARFWADYINKKCETYEAECPKLVAWAKRCMQRDCGQ
VFA
>Solyc07g056460.2.1_SlGSTU22
EVIVLGFWPSMFGLRLRIALAEKEVFEYREEDLKNKSPLLLQMNPIHRKIPVLIHNGICE
SIIGVEYIEEVWKDKALLPYERTQARFWADYIDKKFYWPTKGEEQEIAKKDFIECLKVLE
GVLGDKPYFGGNFGFVDIALIGFYCWFSAYETYEAEFPKFFAWAKRCMQRDSVAKSSPQH
KVLEFVK
>Solyc07g056470.2.1_SlGSTU23
EVILLDFWPSMFGLRLRIALAEKEVYEYREEDLPNKSPLLLQMNPIHKKIPVLIHNGICE
SIIGVEYIDEVWKDKALLPYERAQARFWADYIDKKFYWATKGEELDAAKEEFIVCLKVLE
GALGDKPYFGGNFGFVDIALIGFYCWFSAYETYEAESPKFVAWAKRCMQRDSVAKSSPQH
KVLEFVK
>Solyc07g056480.2.1_SlGSTU24
EVILLDFWPSMFGMRLRIALAEKEIYEYRDEDLRNKSPLLLQMNPIHKKIPVLIHNGICE
SIIGVEYIDEVWKDKAFLPYERAQARFWADYIDKKLYDSTKGEEQETAKKDFIECLKVLE
GALGEKPYFGGNFGCVDIALIGYYSWFYAYESYEAECPKFVAWAKNCMLRDSVAKSLPQH
KVCEFVK
>Solyc07g056490.2.1_SlGSTU25
EVVLLGTYVSMFAVRVKIALAEKGIYEYKEENLVNKSPLLLQMNPIHKKIPVLIHNGICE
SLIIVEYIDEVWNDKSPLLPYKRAQARFWADYVDKKIYDGTKVEEQEAANKEFIECLKVL
EGELGDKPYFDGSFGFVDLALIPYYSWFPAYEKFEPECPKFVAWANRCMQKENVSKYLSP
DKIYDFVV
>Solyc07g056500.2.1_SlGSTU26
EVVLLDTFVSVFGMRVRIALAEKGIYEYKEEDLMNKSQLLLQMNPIHKKIPVLIHNGICE
SLIIVEYIDEVWKDKSPLMPYKRAHARFWADYIGKKIYDGSKVEEHKTANKDFIECLKVL
EGELGDKPYFDGNFGLVDMAFIPYYSWFPVYKKLEAECPKFVAWAKRCMQKESVSKTLVP
DKIYEFIV
>Solyc07g056510.2.1_SlGSTU27
EVVLLDLWVSPFGMRVRIALKEKGIYESKEENLSNKSSLLLKMNPIHKQIPVLIHNGICE
SLIIVQYIDEVWKDKALLPYERAHAKFWADYVDKKIYSTTKGEAQEAAKKELIHHFKLLE
KELGDKTFFGGQFGLVDIALIPFYSWFYALETCIHECPKLVEWAKRCMERESVSTSLPQY
KVYDFIL
>Solyc08g062570.1.1_SlGSTU28
KVTLHGMWTSPYVKRVERALKVKGIYEYVEEDLMNKSELLLTYNPIHKKVPILVHNGICE
SSVIIEYIDETWKNESPLFPYQRAKVRFWASYIHQVLLLYIHIKQTNPYTIFLIDLYIS
>Solyc09g011490.2.1_SlGSTU29
DVKLLGLWYSPASHKVEWALKLKGVYEFIEENLQNKSPLLLESNPVHKKIPILIHNGICE
SMIILEYIDETFEGPSILPYDRALARFWAKFLDDKVGAKGEEQEKGKKEVCEMLNVLDNE
LKKKFFVGDKFGYADMAANFVGYWLGVFQEATSEKFSNFCVWRDEYVNCSQVKEYLPPRN
DLLAFVE
>Solyc09g011500.2.1_SlGSTU30
DVKLLGLWYSFFSHRVEWALKIKGVYEIIEEDLQNKSPLLLQSNPIHKKIPVLIHNGICE

SMIILEYIDETFEGPYILPYDRALARFWAKFLDDKVGAKGEEREKGKEEACEMLKVLDNE
LKKKFFVGDKFGFADIAANLVGYWLGIFQEATSEKYPNFCAWRDEYMNCSQVKEYLPPRN
DELLAFF
>Solyc09g011510.2.1_SlGSTU31
MVILEYIDETFEGPSILPYDRALARFWAKFLDDKVVTKGEENEKAKEEVYEMLKILDNEL
KKKFFVGDKFGIADIVANLVGLWLGVFQEGTSEKFPNFCSWRDEYVNCSQVKEYLPRRDD
LLAFFQ
>Solyc09g011520.2.1_SlGSTU32
QVKLLGFWYSPFTHRVEWALKIKGVYEYIEEDRYNKSPLLLESNPIYKKVPVLIHNGICD
SIVILEYIDEIFEGPSILPHERALARFWAKFLDDKVGAKGEEQEKGKKEVCEMLKVLDNE
LKKKLFVGDKLGFADMVANLVGLWMSVFEEATNENFPNFCAWRNTYISCNQVKEYLPLRI
DELLAFY
>Solyc09g011530.1.1_SlGSTU33
MLKVLDNDFKDKKLFVGDKFGFVDIVANLVELWMGVFQEATNENLPSRIDELLVFYQAYI
RHSSYNFCFS
>Solyc09g011540.2.1_SlGSTU34
EVKLLGVSGSSYSRRVEWALRVKGVYEFIEEDLQNKSPLLLESNPVLKKIPVLIHNGICE
SMVIVEYIDETFEGPSILPYDRAIARFWATFLDGMCLDAKREEKEKNIQEEAYEMLKIVD
NELKKKFFSGDKIGFVDVAANYIPFWVEIVEEATSEKFPNLCAWIDKYLKCSEVQENLPD
RDMMLSFFK
>Solyc09g011550.2.1_SlGSTU35
DVKLLGLWYSPYSHRVEWALKIKGVYEFIEEDLRNKSPLLLESNPIYKKIPVLIHNGICE
SMVIVEYIDETFEGPSILPYDRAIARFWAKFFDEKGSSKGEEQEKAKEELHEMLKVVDNE
LKKKYFVVDKFGFVDIVANVVALWLGVLEEATNEKYPNFYAWRDEYINCSENKKYLPSRN
ELLAKFK
>Solyc09g011560.2.1_SlGSTU36
EVKLLGLWYSPFCHRVEWALKVKGVFEFIEENLQNKSPLLLESNPIHKKIPVLIHNGICE
SMVIVEYIDETFEGPSILPYDRVIARFWVKFFEDKGSAKSEKAKEEVCEMLKILDNELKK
KFFVGDKFGFADIAANFLALWMGILEEATKEKYPNFYAWRDEYINGNKEYLPSRDELLAF
FK
>Solyc09g011570.2.1_SlGSTU37
DVKLLGLWYSPFSHRVEWALKFKGVYEFIEQDLQNKSPILLESNPIYKKVPVLIHNGICE
SIVILEYIDEVFEGPSILPYNRALARFWVKFFEDKGPSKGEEQEKAKEEVFEMLRILDNE
LKKKFFVGDKFGFVDIVANAGALWLGVLEEVTKEKFPNFCVWRDEYCTQNKEYLPSRDEL
LIRFK
>Solyc09g011580.2.1_SlGSTU38
DIKLLGLWYSPFSKRVEWALKTKGVYEYIEDDLQNKSLLLLQSNPIHKKVPVLIHNGICE
SSVILEYIDETFEGPSILPYDRALARFWAKFFEDKWPSKGEEQEKGKEEVNEMLKILDNE
LKKKFFVGNNFGFVDVVANAVALWFGVLEEVTSEKFPNFCDWRDEYYIQNKEYLPSRDEL
FAHYQ
>Solyc09g011590.2.1_SlGSTU39
EVKLLGLSYSPFNHRVEWALKIKGVYEFIEEDLQNKSSLLLESNPIHKKIPVLIHNGICE
SMVILEYIDEAFEGPSILPYDRALARFWAKYVDDKGSAKGEEQEKAKEEAYEMLKILDNE
FKKKYFVGDKFGFADIVANGAALYLGILEEVTSEKFPNFCAWRDEYCIQNKEYFPSRDEL
LIRYR
>Solyc09g011600.2.1_SlGSTU40
DVKLIGLWYSPFSRRVEWALKIKGVYEYIEDDLHNKSLLLLQSNPIHKAVPVLIHNGLCE
SSVILEYIDETFEGPSILPYDRSLARFWAKFFDDKGLAKGEEQEKAKEEVYDMLKVLDNE
LKKKIFVGEKFGFVDIVANAAALWLGVLEEATREKYPNFCDWRDEYCTQNKKYLPPRDEL
LAHYQ
>Solyc09g011610.2.1_SlGSTU41
MVIVEYIDKTFEGPSIIPYDCAIARFWAKFLDDKMPPKGEEQERAKEEAYEILKILDNEL
KQEVLCW
>Solyc09g011620.1.1_SlGSTU42
DVKLLGLWYSPFSHRVEWALKIKGVYEFIEQDLQNKSPLLLESNPIHKKIPVLIHNGICE
SMVIVEYIDETFEGPSILPYDRALARFWVKFLEDQIAAKGEEQEREKKAACEMLKILENE
LKKKFFVGDKFGLADIAANVLAIWLGVFEEATSENYPNLYGWRNEYCNQNKEYLPSRDEL
LIHFQ
>Solyc09g011630.2.1_SlGSTU43
GVKLLGISLSPFSRRVEWALKIKGVYEFVEEDLHNKSPVLLELNPIHKKIPVLIHNGICE
SMVIVEYIDETFEGPSILPYDRAIARFWAKFFDDKCMPVSGEESNKAKEELGDLIKILEN

ELKKNFFVGDKFGFADMAGNLMAYWMGIVEEATSEKFPIFCNWRNEYVNCSTIKEYLPPR
DEILAHFK
>Solyc09g011640.2.1_SlGSTU44
KIKLLGVSLSPFTHRVEWALKIKGVYELIVEDPQNKSPLLLEYNPIHKKIPVLIHNGICE
SMVIVEYIDETFEGPSILPYDRATARFWAKFLDDKCLPTNEEEKEKAKEECGELLKILDN
ELKKEFFVGDKIGFVDIAANALAFWMGIIEEAKNEKFPNYYTWRDNYINCSQVKKYLPSR
DELFSHFQ
>Solyc09g011650.2.1_SlGSTU45
MLGMKQEVYTQGIYEFIEAQRPIKKCPNIIKYNPIYKKVPVFLHKGIPESLVILEYIDEN
WKDTSLLPYQRAIARFWAKFIDEKCLPESNYEVKVKAMGELQELLKLLENELMNKIFFGG
ENKVGYMEIVSILITYWLGVMQEANKKEFPNICGWADKVISFSFMKENLPPREKLLAIYK
>Solyc09g063150.2.1_SlGSTU46
QVKLFGAFPSPFSHRIIWALKHKNIYEYIEEDLSNKSQHLLTYNPIYKMIPILVHNEIVE
STIILEYIEETWPQNPLFPYEKAKARFWIKFGEDKNSEIGEEQVKATENAKKILKIIEEQ
GLDKKFFSGDTIGLIDIAFGWLAFWLEVIQEAEPNNFPHLQSWINNFKQVAIIKENIPNR
NAMLDYFK
>Solyc09g091130.2.1_SlGSTU47
DLKLHGSWASPYSLRIIWALKLKGLYEYIEEDLANKSDLLLKYNPIFKKIPILVHDGICE
SMIILEYLDQIWPNQYPLLSYQRALARFWVNYFEQKSVSKGEEQEKAVKDSLEMLKIIEE
NAFKNNIFFIGGKIGIVDISFGWICHWLKIIEDVEENSFPNLQNWMKKFKEVPLIKESLP
NHQKLFLPFK
>Solyc09g091140.2.1_SlGSTU48
EVKLHGTSYNLFTYRVIWALKLKGIFEYIEEEHSNNGSLIMKYNPVFKRFPILFHGEISE
SMVIIEYIEDTWPQNPLLPLDRSIARFWVKFAGDKGACVGEKQEKAIKETMEMLKIIEEQ
AFEENIFFGGEKIGIVDLAFGVIPHWLEIIEDIEPNLFPNLLNWVQNFKEEQIIKENLPN
YEEMFVFLK
>Solyc10g007620.1.1_SlGSTU49
EVILLDFWPSMYGMRVRVALAEKCVFEYKEQNMIEKSPILLEMNPIYKKIPVLIHNGICE
SLNVVQYIDEVWKNKVFLPYEKYQAMFWADYVEKVFDTEKGGEKQTRKGNYIDTLRMLEG
IIGDKLYFGGKFGYLDICLIGICSWFYTYEKFEVETPKIIAWMKRCMKRESVYKNVVPLK
VYDFAL
>Solyc10g007640.2.1_SlGSTU50
EVILLDFWPSMFGMRVRVALAEKAIYEYKEEDLFTSKSPLLVKMNPIHKKIPVLIHNGVC
ESFVVVEYIDEVWKDKALLPYDRSQARFWASYTDKLYDFVKREEFAEGKKDFIDPLKLLE
EALGDKPYFGGSFGFVDIALIGFYSWFYTYETIEAECPKIAAWGKRCMKRESVSKSLASR
KIYEVVI
>Solyc10g084960.1.1_SlGSTU51
RVKLLGVYGSPASQRVEWALKIKGVYEFITEDLQNKSPLLLKSNPVYKKIPVLLHNDIAE
SLVIIEYIDEAFEGPSILPYDRAIARFWVKFLDEKCLPAQGDEQEKDKEEAYEVLKVIDN
ELKKKFFGGDNIGFVDVVANFVGFWIGIVEEATSENFPNFCAWRDEYLNCDRVKENMPSR
EMLLGYFK
>Solyc12g011300.1.1_SlGSTU52
EVILLDFWCSMYGMRARIALEEKGVYEYKEEDLKNKSPLLLQMNPIHKKIPVLIHNGICE
SLVIIQYIDDVWKDIGLLIPYDKAQAWFWSDYMDNTVHEYTKGEEQEQAIKDFLGGLKLL
EGVLGDKPYFGGNFGFLDVSLIGYYSWFLAYETFELECPKLISWVKRCMERESVSKALPS
KKVCEFVL
>Solyc12g011310.1.1_SlGSTU53
EVVLLSAYVSMFGMRVRIALHEKGIYEYKEEDLSNKSELLLQMNPIHKKIPVLIHNGICE
SLIIVEYIDEVWKDKSPLMPYKRAQARFWADFIDKKVYDSTKGEDQEAAKKEFIEYLKLL
EGELGDKTYFNGNFGFVDLALIPFYSWFPTFEKFEKECPKFVAWANKCIYKDSVSKSLAS
NKVYEYVL
>Solyc12g011320.1.1_SlGSTU54
EVVLLDYWASPFGTMARIALVEKGVFIHKFEDLSNKSPLLLEMNPVHHKIPVLVHKGICE
SNIIIQYIDEIWKNNSLLPYQRAKARFLVDFINKKVHGSGQIEEQENGKKELVECSKFLE
EELGDKLYFGGVFGFVDIALVPFYNWFIVFKTFIEIQCPKLVMWGERCLNRDSVSKSLPS
NQVYQAYL
>Solyc12g062730.1.1_SlGSTU55
KVTLHGMWANPYVKRVELALKVKGIYEYVEEYLMNKSELLLTYNPIHKNTWKNSPLFQYQ
RAKVRFWASYIHQVYDCMKEALKRFYAKLSVLEDGINLGITSNMNNIGMLDIMIVITLGA
YKVQEEVEEENTPLLYSWVTTLIDLPIVKGITPPHDKVVSFLQ
>Solyc12g097080.1.1_SlGSTU56

IVKLIGTPFSFFTYRVIWALKLKGIYEYIDEDMSKKSSLLVKYNPIHKKVPVLIHGDICE
SMVIVEYINETWKLNPLLSYERATSRFWAKYIEEKSHSTGEKQQNAIKESLEMFKTIEEN
ALENNILFGGENIGFVDIAFGGYSLWMEIIEEINPHNFPRINNWIKKFKEVQTIKDNLPN
RDEMFVYMK
>Solyc05g013950.1.1_SlDHAR1
MAVLRFPSLFYLGLLLRGSKIFSTFVSFMKSKDSSDGTEQALLDELKALEEHLKVHGPYV
DGKNVCSVHMILAPKLYHLEVALGHFKKWSVPESLSHVRNYMNDFLGSGANFPVGNVSC
>Solyc05g054760.2.1_SlDHAR2
PDVLGDCPFSQRVLLTLEEKKVYKKHLINVSDKPKWFLEVNPEKVPVINFIPDSDVIVGI
IEEKYPNPSLIPPEFASVGSKIFPTFVSFLKSKDSSDSTEQALLDELKALEEHLKAHGPY
INGQNVCSVDMSLAPKLYHLEVALGHFKKWSVPESLSHVRNYMKLLFERESFQKTKAEEK
>Solyc06g075520.2.1_SlDHAR3
MESKDSSDCTEQALFDELKALEEHLKAHGPYVNGQNVCSVDMSLAPKLYHLKVALGHFKK
WSVTESLTHVRNYMKDFVGNMSCFLF
>Solyc09g056180.2.1_SlDHAR4
MKSKDSSDCTEHALFDELKALEEHLKAHGPYVNGQNVCSVDMSLAPKLCHLEVALGNFKK
WSVTESLSHVRNYMKNDIKSAFWSMLLCYIE
>Solyc11g011250.1.1_SlDHAR5
PNKLGDCPFTQRVLLTLEEKHLYDMKFVDLSNKPDWFLKISPEKVPLIKLVPDSDVISQA
LEEKFPKPPLTPPEKASVGSKIFPKFVAFLKSKDSGDGTEQALLDELTAFNDYLKENGPF
INGNEVSAADLSLGPKLYHLEIALGNYKNWSIPDSLSYMKSYMKSIFSRESFINTRALKE
>Solyc11g039930.1.1_SlDHAR6
MKSKDSSDCTVQALFDELKALEEHLKAHGPYVNGQNVYSVDMSLAPKLYHLEVALEHFKK
WSVTECLSHVRNYMKLDPFLLPVVQGSI
>Solyc02g081340.2.1_SlGSTF1
VVKVYGSAMAACPQRVMVCLIELGVYELIHVDLDSLQQKKPDFLLQPFQVPVIEEGDFRL
FESRAIIRYYAAKYEDKGKKLTLEEKALVDQWLEVESNNYNDLYNMVLQLLVFPKMGHKS
LIVVQKCANNLEKVFDIYEQRLSKSKYLAGDFFSLADLSHLPSLRFLMNEGGFAHLVTQR
KYLHDWYLDISSRPSWSKVLDFMNLKKLEML
>Solyc06g009020.2.1_SlGSTF2
AIKVHGPMMSPAVMRVVATLKEKDLFELVPVNMQAGDHKKEPFISNPFQVPAFEDGDLKL
FESRAITQYIAHTYADKGNQLLPKKMAVMSVWMEVEAQKFDPISKLGFEIVIKPMLGMVT
DAVVAENEEKLGKLLDVYESRLKESKYLGGESFTLADLHHAPSLHYLSGSKVKSLFDARP
HVSAWVADILARPAWSKTIELSKQ
>Solyc06g009040.2.1_SlGSTF3
AIKVHGPMLSPAVVRVVAMLKEKNLFELVHVDLQNGDQKKEPFISNPFQVPAFEDGDLKL
FESRAITQYIAHTYADKGNQLLPKKMAIMYVWIEVEAQRFEPVSKLCYEIVIKPLLDMVT
DAIVAENEEKLSKLLDVYESRLKDSKYLGGDSFTLADLNHAPALHYLMGTKVKSLFNARP
HVGAWVANILARPAWAKSLELTK
>Solyc09g074850.2.1_SlGSTF4
MRVISCLIEKDLFEFVFVDMAKEEHKRHPFLSNPFQVPAFEDGDLKLFESRAITQYIAQV
YASNGIQLIPMKMAIMSVWMEVEGQKFEPPSKLTWELVIKPMIGLGSDVIVKESEEQLSK
VLDIYETRLTESKYLGGDSFTLVDLHHIPNIYHLMNTKAKALFDSRPRVSVWCADILARP
AWVKGLEKMQK
>Solyc12g094430.1.1_SlGSTF5
PVKVYGPTLSTAVSRVLACLLEKNVFHLIPVNMAKGEHKKPAYLKQPFQVPAYQDEDITL
FESRSINRYICDKYGSQGNGLYPLEKASIDQWIEAEGQSFNPPSVLVFQLAFAPRMKLKE
NLIRQNEEKLKKVLDVYEKRLGDSQYLAGDEFTLADLSHLPNIQYLVNGTDRAELITSRE
NVGRWWGEISNRESWKKVVEMQTSPPPS
>Solyc04g009530.2.1_SlGSTL1
TPKLYISYSCPYAQRTWIARNCKALEEIKLVPIDLKNRPDWYKEVYPAKVPSLEHKGESM
DLIRYIDSNFEGPSLFPSKREFAEELFSYFDSFYKAVISSLKEDKINDAIAAFDSIETAL
SKFVDGSFFLGSLSLVDIAYAPFIERFQPFLLEVKNYDITTGRTKLAAWIKEMNQIEGYT
VTKRDP
>Solyc09g007150.2.1_SlGSTL2
TTRLYINYQCPYSQRVWITRNVKGLDKINLVPIDLQNMPDWYKEVYPQKVPSLEHIGESL
DLVKYVDSNFEGPSLLPEKRKFAEELIAYSDIFVPEVYKSFFRDAQTLAGAQFDYLEKAL
DKFDDGPFFLGQFSQVDIAYVPFIERFQIFMEKGINYDITSARPKLAKLIEEMNKLDGYK
QTKVLDP
>Solyc10g084400.1.1_SlGSTL3
TTRLYISYVCPFAQRPWIARNFKGLDKIELVPIDLQNRPVWYKEVYPQKVPSLEHIGESL

DLVKYIDSNFEGPFLLPEKQKFAEELIAYSDTFLKEIYANFKGDIEKHAGPQFDYLEKAL
DKFDDGPFFLGQFSQVDIVYAPFVERFQIFLKEGLNYDITSGRPKLAKWTEELNKLDSYI
QTKADP
>Solyc12g044520.1.1_SlGSTL4
TTRLYINYQCPYSQRVWITRNVKGLDMIKLVPIDLQNRPDWYKEVYPKKVPSLEHTGESL
VLVKYVDCNFEGPSFLQEKRKFAEELIAYSDTTFVPEVYRSFAKDARTLAGAQFDYLEKA
LHKFDDGPFFLGQFSQVDIIYAPFVERFHVFMPEGFNYDITTGRPKLAKWTEEMNNLDGY
KQTKVLEQ
>Solyc12g044530.1.1_SlGSTL5
TTRLYINYQCPYSQRVWITRNVKGLDMIKLVPIDLQNRPDWYKEVYPKKVPSLEHTGESL
VLVKYVDCNFEGPSFLQEKRKFVEELIAYSDTTFVPEVYKSFAKDARTQAGVQFDYLEKA
LHKFDDGPFFLGQLSQVDIIYAPFVERFHVFMPEGFNYDITTGRPKLAKWIEEMNNLDGY
KQTKVLEQ
>Solyc04g050560.2.1_SlGSTT1
MLDWHHSNLRHGTAGYIFDTVLAPAFGLTLNPQAAAEAEKVLLASLANIESVWIQKKGRL
LLGSGQPSIADLSLVRELMELEAKHKHLKPRQRICSVN
>Solyc08g080900.2.1_SlGSTT2
TLKLYVDRMSQPSRAVIIFCKLNGIFEEIHINLSKRQQLSPEFKEINPMQVPAIIDGRFK
LFESHAILRYLACAFPGIADWYLYKRAKVDSVLDWHHSNLRRGAAGYIFNTVLAPAFGLP
LNPQAAAEAEKVLLASLAKVESVWLQRKGRFLLGSGQPSIADLSLVCEIMELEILDEKDR
ERIIGPYKRVLKWIDDTKNEPHFQEVHVILFKAKEKFH
>Solyc08g080910.2.1_SlGSTT3
TLKLYVDRMSQACREVIIFCKLNGIFEEVHIDLSKRQQLSPEYREINPIQIPAIMDGRFK
LSESHAILKYLACAFPRIADWYLYKRAKVESVLDWHRTNFPRGPGSYTFYSVLAPTVGLP
LNTKAAARTEKMFIACLATIESVWLQKKGRFLLGSDQPSIADLSLACEIMQLEILDEKDR
ERILGPFKRVLKWLDDTKNAPHFEEVQSTLAGYKEKVQ
>Solyc12g056250.1.1_SlGSTT4
SLKVYVDRLSQPSRAILIFCKLNGIFEEVNIDLAKGQHRTPEYQEVNIMQVPAIVHDTFK
LFESHAILRYLASAFPETADWYLQKRANVECVLDWHHANLRRGSAGYVFNTILAPAFGLP
LNPQAAAEGKNLLSASLATIDTYWLQKDGSFLLGNSQPSLADLSLVCEIMQLQFLDEKDR
EGLLSPHKNVLKWIDDVKSAPYFDEIHATLFKVSEIFQ
>Solyc01g091330.2.1_SlGSTZ1
KIVLYSFWQSSCSWRVRFALNLKGLYEYRAVNLGKGEQFTSEFDKLNPLYVPVLVDGDVV
ISDSYAILLYLEEKYHQRPLLPQLRALNLQAASIVSSNMQPLMLSVLRYMEERVGPEEKQ
LWAKFHIQKGFGALEKLLTGSAGKYATGDEVYMADVFLAPQIAVATKRFDIDMSEFPTLR
KIYDSCEALPEFQASLPERQPDASP
>Solyc01g102660.2.1_SlGSTZ2
KLQLYSYWRSSCAFRVRIALNLKGLYEYKAVNLLKGEQRDPEYLKLNPLYVPTLVDGDAV
IADSFAILMYLEEKYPQRALLCQKRAINYQAANIVSANIQPLNLAVLKYIQEKIGPDETT
PWVQGHITKGFEALEKLLKDYAGKYATGDEVYMADLFLAPQIHAAIKRFEVDMNQFPTLL
RVFEAYQELPAFQDAMPEKQPDAIHHL
>Solyc11g028090.1.1_SlEF1Bg2
MDLFLKMLSHVRPCLIFFHLSSHHIAKYSFDTDFTNSRSYQINAQQSSFGSSLFEYSEEA
AVSALKRALGTLNTHLASTKYLVEHLITLADNIVVCNLSIGFRMIMTKSFTKEIPRVERY
FWTVVNQQNFSKILGKVKQAKSILA
>Solyc04g057890.2.1_SlTCHQD
MQLYHHPFSLNSQKVRLTLEEKGIYTSHHVNPLTGKNMDAFFFMNPSKVPVFQNGSHIIY
DTIEIIQYIERIAEKVNLSSREVIGWMHKIQEWDSMYFTLFHVPEKYRLCVSKFLRRVII
ARMAESPDLASAYHCKLRQAYDTDDKLKNADVLRRSENHLVRLLDEVELKLGETSYLAGE
EFSLADVMLIPLLARIELLNLENEYINSRPNIADYWVLVKQRPSYKKVIGKYFDGWRRRK
>POPTR_0005s26400.1
MRRVNHLATLSRAFSGAASHQRLVQGAAMISTYAASNSQCFASRIAESFRISNPPVARGV
TGTMFFSVAASSLAQEAQAKEAPPVEKLMPKDVVLYQYEACPFCNKVKAFLDYYNIPYKV
VEVNPINKKEIKWSDYKKVPILKIDGEQMVDSSDIVDKLFQRIHPDNSVTDSDEERQWRG
WVDNHLVHVLSPNIYRSVSEALESFDYITTHGNFSFTERLVAKYAGATAMYFVSKKLKKR
HNITDERAALYGAAETWVDALKGRQYLGGLKPNLADLAVFGVLRPIRYLKSGKDMVEHTR
IGEWYSRMENAVGEPSRIKA
>POPTR_0012s04410.1 (eugene3.00120387)
MRRASTLASSVLSRTLTPTLHEGGALSTINHRFLLVALYSTTSNTGSSHSRRIFNPFSSS
LGVAGALVSAAAAASLSQDVLAKEPPRAELVPKEVVLYQYEACPFCNKVKAYLDYYDIPY
KVVEVNPISKKEIKWSDYKKVPILTVDGEQLVDSSAIIDKLRNKIHGKEIVESASDKDDD

EEIKWRRWVDNHLVHVLSPNIYRNTSEALESFDYITSNGNFSFTEKITVKYAGAAAMYFV
SKKLKKKYNITDERAALYEAAETWVDALNGREFLGGSKPNLADLAVFGVLRPIRYLRSGR
DMVEQTRIGDWYTRMENAVGESARIKA
>POPTR_0015s04620.1|POPTR_0015s04610.1
MRRASTLASSVLSRTLSSTLHDCSGLSTTSAATINHRFLHAALFSTTTSTGSSSTRRIFN
PFSTYLGVAGALVSAAAAASLSQEVLAKEPPPAELVPKEVVLYQYEACPFCNKVKAYLDY
YDIPYKVVEVNPISKKEIKWSDYKKVPILLVDGEQLVDSSAIIDKLGNKIHGKEIVDSAS
DKDDDEEKKWRRWVDNHLVHVLSPNIYRNTSEALESFDYITSNGNFSFTERITVKYAGAA
AMYFVSKNLKKKYNITDERAALYEAVETWVDALNGREFLGGSKPNLADLAVFGVLRPIRY
LRSGRDMVEQTRIGDWYTRMENAVGESSRMKA
>LOC_Os04g17050.1
MRSIRAAQALASRSLLLSSRALHGDAASTAAAAAGGGRLGVQPSPPSQASSSSSSRAMPA
GIAGAVSFSLTFATMAAAEAKERPPMDLLPQNVVLYQYQACPFCNKVRAFLDYHDIPYKV
VEVNPLSKKEIKWSEYKKVPILMVDGEQLVDSSDIINILQQRVRPDDKATNEEEEKWRRW
VDEHLVHVLSPNIYRTTSEALESFDYISKHGNFSFTERFAVKYAGAAAMYMVSKKLKKKY
NITDARASLYDAANTWMEALDGRDFLGGSKPNLADLAVFGVLRPIRYLTAGKDMVEHTQI
GDWYQRMEDAIGEPSRIQE
>At-mPGES2@NP_199030.1
MRRVTGLAARTISSSVAINSRLTQSMAITTISSSEPISRRFGGLPEIKTPSFAGGVAGVV
FFSAAAVSSLGQEVHAKEMAQKFNPKEVVLYQYEACPFCNKVKAFLDYNKIPYKVVEVNP
ISKKEIKWSDYKKVPILTVDGEQMVDSSVIIDSLFQKMHPEISKSEDDEETKWRKWVDNH
LVHLLSPNIYRNTSEALESFEYITTHGNFSFTERLVAKYAGATAMYFVSKKLKKKYNITD
ERAALYDAAETWVDALKERPYLGGSKPNLGDLAVFGVLRPIRYLRSGKDMVDNTRIGEWY
SRMENTVGEPSRIKE
>Sly-mPGES2@XP_004238374.1
MRRVSRIAALYRAVDGAAAMEVPQHRMSTAAQFSTSSNKSSTRSNWLFNNLLTDLSARTS
AHAVAGTMLFSVAATTLTEEVHAKEVVPPELRPKDLVLYQYEACPFCNKVKAFLDYYDLP
YKIIEVNPISKKELKWSDYKKVPVVLVDGEQMVNSSGTYYHQYIYSYYADIIDKLYEKVR
SGDSTFDADEESKWRKWVDDHLVHMLSPNIYRNTSEALESFDYITSHGNFSFTERITAKY
AGAAAMYFVSKKLKKKYNITDERAALYEAAETWVDALKGRDFLGGSKPNLADLAVYGVLR
PIRYLKSGRDMVENTRIGDWYSRMESEVGVSARIQA
>Hvu-mPGES2@BAK04530.1
MRSLRAAQTLLASRSFLLSSRALHAAAASPAAAGRWGNAPPPPPTPTPCPGPSSRAGIAG
AVSFSLTFATVAVAEVQAKERLPSDLLPRNVVLYQYQACPFCNKVRAFLDYHDIPYKVVE
VNPLSKKEIKWSEYKKVPILTVDGEHLVDSTDIINILQHRISPDDEVTNEEETKWRKWVD
EHLVHVLSPNIYRTTSEALESFDYIAKHGNFSYTERFAVKYAGAAAMYFVAKKLKKKYNI
TDERASLYDAANTWTEALNGRNFLGGPKPNLADLAAFGVLRPIRYLQSGKDMVEHTQIGE
WYQRMEDAVGEPSRIPEGQYQE
>Phpat.022G051000.1
MAAVRVMAQRIARARRSGLAAAAQQYGRVIGTSEGDDAVLRVGTRAAGMEKAMAVVEEEQ
QQGARNWRRWSSGSWIAAGAASLSFAASTMTVAYGKERVTDRFSPKEVVLYQYDACPFCN
KVKAFLDYHDIAYKVVEVNPVGKKEIKWSDYKKVPILVVDGEALNDSTAIITELTRRIQG
GNAKDLAQKIGSDEEEKWRSWVDEHLVHLLSPNIYRTPREALQAFDYLTTNGNFSSIERA
TGKYVGATAMYFIGKRLKKRHNIIDARASLYEAAEEWVAALNGRSFMGGSKPNLADLAVF
GVLRPIRHLDTGKDLLASTQIGEWYMRMEDAVGETARLPEEPLMGTIDSSMKV
>Phpat.019G052100.1
ASLPCGRRPCPLKLNEEWELLVRQRAERMLGVDCLRFHHCLARCTEIHCCHCGEKHIFVI
GARSIVVISGRIGTEAAGTMMSVRAVAHRCARARSGAQAVQRCAGVVWTSEGHDDVRRFG
MQAAGVAKGNSSGLDRRTWSEEDARGRSWNARWIGAGSFSLSFATSTIGVAYGKERVADR
FAPKDVVLYQYETCPFCNKVKAFLDYHDIAYRVVEVNPMGKKEIKWSDYQKVPILVVDGE
ALKDSTAIITELTRRIEGGNANAPALKPESDEEERWRRWVDEHLVHLLSPNIYRTPRESL
QALDYLTTSGNFSMMERATGKYFGAAAMYIIGKRLKKRHNIVDERISLYDAVEEWVKALD
GRQFMGGSKPNLADLAVFGVLRPIKSLDTGRDMLASTKIQEWYSRMEDTVGATARLQEES
LMGSIDNSVKV
>AT2G19080.1
MEGDQETNVYTLVARKPSFDLPTACPNCLPAYIYLKLAQLPFELAFNSTFPDSDELPYFE
SDTYVAYNNEDGGVIEKLKKDGIVNLDSQLQSLSDYLSLKALIVSWLEEALTYEIWVGTE
GISTSKIYYSDLPWVISKVLFYKQTYLAKNRLGITKENAEQREKQIYKRASEAYEALSTR
LGEQKFLFEDRPSSLDAFLLSHILFIIQALPVTSVLRCKLLEHSNLVRYAEKLKSEFLEA
SSSSPSPPLHSFPSSFPRKSSKPKSKPKVEKTEEEKKFKKRARFFLAAQFLAVVIYVSVM
GGGSSDELEYEDEDD

>Solyc07g009320.2.1
MEEANEREKLTLVTRKSCFGLPTSCPNCLPVYVYLKFSATPFDLDFNLNNPDSDQIPYVE
SGTYVAYNNEKGGVIRSLSEDGFVDLDSQVRGIPEWISAKAMVDSWLADATIYELWVGSD
GTSAHKIYYSDLPWPLGKILYLKQVHVVKQILGITKENAERREEEIYRNANDAFSALSTR
LGEQAYFFENRPTSLDAVFLGHALFTLYALPETSVLRSKLLEHDNLVRYTEKYKSELVDS
SGSSSSGTHSQSDPSSSVPRRPSQWSSKPKSKPKREKTEEEKKFRRRAKYFLVTQLVAVL
VFLSLLGGSDAAEVELDEDDDGADYD
>Potri.006G077100.1
MQESQERAEYTLVARKPSFGLPTGCPICLPVYIHLKFASFPFRLDFNNTFPDSDQIPYIE
SGTYVAFNDENGGLIERLREDGIVDLDAAFCSLPEWISMKAMVCTWLAEAVMYELWVGSD
GTSARAIYYSDLPWLIGKALFMKQVYVVKQRFGITKENAERKEAEIYKRAKIAYGALSTT
LGDQTFLFERPSSLDAYLFGHVLFTLQALPESSVLRLALSEHGNLIRYAEKLKSEYLEGG
SSSSVPQFHSEASSTSTRRPSNSSSKTKKQPKREKTEEEKTFRRRAKYFLVTQLVAVLVF
LSVMGGYDFSEVEVDDDEGFSYD
>Potri.T161600.1
MQESQERAEYTLVARKPSFGLPTGCPICLPLFIYLKFSNFPFHLVFNNTFPDSDQIPYSE
SGTYVAYNDENGGVIKSLKEDGIVDLDTDFSSLPEWISMKAMVSTWLADAIMYELWVGSD
GTSARTIYHSGLPWLIGKALLMKQVHVVKQRLGITKENAERREAEIYKRAKIAYGALSTT
LGDHTFLFERPSSLDAYFLGHVLFTLQAFPESSMLQSALLEHGNLIRYAEKLKTDFMEAG
SSSSVPQFLSDASSTSTRRSKPKKQPKRERTEEEKTFRRRARYF
>Phpat.012G046000.1
MMDMVSSTSSAWAESSSGSADLVLVTRPPAFGLPTACPACLPAYLYLRLAGARFDVHVTA
VEPDSEDLPSVEYGENVGFASENGGVVEFLREEKIVDLDAGLNERERAELETCKAMMQSW
VADASAYEVWMRDNSRQCKTVYFSELPWGLVQALDWKQRLAVMQRLEITPENTMTRTEEL
YKKASNAYSALSILLSDQKYFFNDRPTSLDALVLGHLLFHLRVPLEVSTLKEAILKYQNL
VDYAEHWSKYLLGEEGNSIDSSFRPKTPHSSHASPPRQGPKERDNPEKERREKDIFFKKR
AKYFVIAQVTAVLMYVVFAGYGVDDDGEDDGGDDDD
>Phpat.004G037800.1
MESSGDSVDLVLVTRPAGFGMPTVCPACLPVYLYLRLAAVSFREQVSAVEPDSVDLPCVE
YGENVGFSSENGGVIEFLRKEKIADLDADLSDSEKAELETCKAMMESWVADASAFEVWTR
DNDRQCKVVYFSELPWGLVQALDWKQRLAVMQRLGITPENTVARSEELFRKASNAYSALS
VLLSDRKFFFNNRPTSLDALVLGHLIFHLRVPFEISTLKGEILKYQNLVDYAESWGKQLL
DKQAILANPAFRPKAPPSPPLRPTKLGSNEREEPAKKARSESDILLKKRAKYFLIAQFLA
VLMYIFIAGYEVDDEDLDVDDD
>AK366355 MLOC_6040 POPSEQ: contig: morex_contig_136998 chr: 7 cM: 41.9971671388102 BLAST2GO: 938020 at2g19080
MASATTAAAEWEAAARRTLVARKPGFGLPTACPTCLPVLLYLRMSQVPFDIHVDSRFPDA
DHIPYVEFGECVAFNNENGGVIEYLREEKIVDLTSKHPSVSYSDVLPTKAMISTWLADAL
QYELWVANDGAHWSIARDIYFSDLPWPIGKVLYWKKIREVKQLLDITKLNAAEKEEEIYR
KATAAYDALSTKLGDQSFLFDDSPTDVDALLLGHVLFVLNALPATSMLRSYLQNYDNLVK
LAEDIKVQLVGVDSSAAGSASSDPPSSSTPRKTASSGQSYKPKPKAKKERTEEEKKFRRR
TKYFLAAQLISVLVFLSIMGGVDSSELDDDYELEYED
>LOC_Os06g07160.1
MASAAAAAAAEWEEAERKVLVARKAAFGLPTACPTCLPVLLYLRMCNVPFDIHVDSSFPD
ADHIPYVEFGECVAFNNEKGGVIEYLKEEKIVDLNSKHPSVSYSDVLSTKAMVMTWLSDA
LQYELWLASDGSIPHDIYFSDLSWPIGKILYWKKTREVKQQLGITKLNAAEKEEEIYQKA
NAAYDALSTRLGDQIFLFDNSPTDVDALFLGHALFVLNVLPDTSVLRSCLQKYDNLVNFT
KHLKVQLLEADSDSSATGLGSTDPSSSSTPRKRASSGRSYKPKPRAKKERTEEEKKFRRK
AKYFLATQLVAVLLFLSLMGGADSSELDDEDGVDYED

2. Sequences listed in Table 1 and used for the phylogenetic analyses of Cys-GSTs

DHAR class

>Aquca_002_01303
MSSATSIIPPTAFVALSSTLKYRVNKFSQNNNNNIKNFFFTVRRNSSRARRSLTITSMSTTTSSDPLEVCVKASI
TSPGKLGDCPFTQRVLLTLEEKHLPYEMKLVDLTNKPEWFLKISPEGKVPVIKLEEKWIADSDVITQSLDEKYPD
PSLTAPPEKASVGSKIFSTFVGFLKSKDPSDGTEQALLNELTVFNDYIKENGPFINGKDVSAPDLALGPKLYHLE
IALSYYKNWSIPDSLSYTKSYMKTIFSRESFVNTRALKEDVIAGWRSKVMG
>Aquca_027_00233

MALEVCTKAAVGVPDALGDCPFTQRVLLTLEEKKVPYKLHLIDVSNKPQWFMEANPEGKVPCVKFDGKWTPDSDV
ITQILEEKYPEPSLVTPPEYSSVGSNIFSSFVKFLKSKDASDGSEQALVDELKALEDHLKEHGPYVNGENVSAVD
LSLAPKLYHLVVALGHFKEWSIPKDLTYVNSYVELLFARESFLKTKPEKKEYVIAGWAPKVNG
>472181
MALEICVKAAVGAPDHLGDCPFSQRALLTLEEKNLTYKIHLINLSDKPQWFLDISPQGKVPVLKIDDKWVTDSDV
IVGILEEKYPDPPLKTPPEFASVGSNIFSTFGTFLKSKDSNDGSEQALLHELEALENHLKSHDGPFIAGERVSAV
DLSLAPKLYHLQVALGHFKSWSVPASLPHVHNYMKDLFSLDSFEKTKTEEKYVISGWAPKVNP
>476670
MALDICVKVAVGAPDVLGDCPFSQRVLLTLEEKKLPYKTHLINVSDKPQWFLDISPEGKVPVVKLDGKWVADSDV
IVGLLEEKYPEPSLKTPPEFASVGSKIFGAFVTFLKSKDANDGSEKALVDELEALENHLKTHSGPFVAGEKVTAV
DLSLAPKLYHLEVALGHYKNWSVPESLTNVRNYAKALFSRESFEKTKAKKEIVVAGWESKVKA
>AT5G16710-DHAR3
MISLRFQPSTTAGVLSASVSRAGFIKRCGSTKPGRVGRFVTMATAASPLEICVKASITTPNKLGDCPFCQKVLLT
MEEKNVPYDMKMVDLSNKPEWFLKISPEGKVPVVKFDEKWVPDSDVITQALEEKYPEPPLATPPEKASVGSKIFS
TFVGFLKSKDSGDGTEQVLLDELTTFNDYIKDNGPFINGEKISAADLSLAPKLYHMKIALGHYKNWSVPDSLPFV
KSYMENVFSRESFTNTRAETEDVIAGWRPKVMG
>AT1G75270-DHAR2
MALDICVKVAVGAPDVLGDCPFSQRVLLTLEEKKLPYKTHLINVSDKPQWFLDISPEGKVPVVKLDGKWVADSDV
IVGLLEEKYPEPSLKTPPEFASVGSKIFGAFVTFLKSKDANDGSEKALVDELEALENHLKTHSGPFVAGEKITAV
DLSLAPKLYHLEVALGHYKNWSVPESLTSVRNYAKALFSRESFENTKAKKEIVVAGWESKVNA
>AT1G19570-DHAR1
MALEICVKAAVGAPDHLGDCPFSQRALLTLEEKSLTYKIHLINLSDKPQWFLDISPQGKVPVLKIDDKWVTDSDV
IVGILEEKYPDPPLKTPAEFASVGSNIFGTFGTFLKSKDSNDGSEHALLVELEALENHLKSHDGPFIAGERVSAV
DLSLAPKLYHLQVALGHFKSWSVPESFPHVHNYMKTLFSLDSFEKTKTEEKYVISGWAPKVNP
>Bostr.7128s0558
MALEICVKAAVGAPDHLGDCPFSQRVLLTLEEKNLTYKIHLINLSDKPQWFLEISPQGKVPVLKIDNKWVTDSDV
IVGILEEKYPDPPLKTPPEFASVGSNIFGTFGTFLKSKDSNDGSEQALLHELEALENHLKSHNGPFIAGERVSAV
DLSLAPKLYHLQVALGHFKSWSVPASLPHVHNYMKALFSLDSFEKTKTEEKYVISGWAPKVNP
>Bostr.3288s0176
MALDICVKVAVGAPDVLGDCPFSQRVLLTLEEKKLPYKIHLISVSDKPQWFLDISPEGKVPVLKLDGKWVTDSDV
IVGLLEEKYPEPSLKTPPEFASVGSKIFGAFVTFLKSKDANDGSEKALVDELEALENHLKTHSGPFVAGEKVTAV
DLSLAPKLYHLEFALGHYKNWSVPESLTNVRNYAKALFARESFEKTKAKKEFVVAGWESKVNA
>Bostr.2618s0009
MISLRFQPCTAGVLSASVSRAGFIKRCGSTKPGRTVRFGTMAMAASPLEICVKASITTPNKLGDCPFCQRVLLTM
EEKHVPYDMKMVDLSNKPEWFLKISPEGKVPVVKFDEKWVPDSDVITQVLEDKYPEPPLATPPEKASVGSKIFST
FIGFLKSKDSGDGTEQVLLDELSTFNDYIKENGPFINGEKISEADLSLAPKLYHMKIALGHYKNWSVPDSLSFVK
SYMENIFSRESFANTRAQTEDVIAGWRPKVMG
>Bradi1g45010
MAVLLRTTTCATASTTGSSTLLATTFRQGRRLSRPRALPTRRAFTARASAEPLEVCAKASITVPDRLGDCPFTQR
VLLTIEEKHLAYDLKLVDLANKPDWFLKINPEGKVPIVKLEEKWVADSDVITQALEEKYPQPSLAIPPERASTGS
KIFSTFIGFLKSKDPNDGTEQAILSELTSFNSYLEDNGPFINGGTVSAADLSLGPKLYHMEIALGHYKTWSVPDA
LAHVTTYMKTIFSRDSFVKTRALPEDVIAGWRPKVMG
>Bradi2g37480
MTVEVCVKAAVGHPDTLGDCPFSQRVLLTLEEKKVPYEMKLIDVSNKPEWFLKINPEGKVPVFNSGDGKWIADSD
VITQIIEEKYPTPSLVTPPEYSSVGSKIFSTFIAFLKSKDASDGTEKALLDELQALEEHLKAHGPYINGENVSAA
DLSLGPKLFHLQVSLEHFKGWKIPETLTGVHAYTEALFSRESFAKTKPAKEHLIAGWAPKVNP
>Brara.F01386
MALEVCVKAAVGAPDALGDCPFSQRVLLTLEEKNLPYKMHLINISDKPQWFLAISPEGKVPVLKNDDKWVSDSDV
ITGILEEKYPEPSLKTPPEFASVGSKIFGTFVTFLKSKDSSDGSEKALLDELEALETHLKTHDGPFIAGGKVSAV
DLSLAPKLYHLKVALGHYKSWSVPESLPHVHGYMKALFSLDSFEKTKTEERYVIAGWEHKVNP
>Brara.B02202
MAALEICVKGAVGAPDVLGDCPFSQRVLLTLEEKKLPYKIHLANVSDKPQWFLDISPEGKVPVMKLDGKWVADSD
VIVGILEEKYPEPSLKTPPEFASVGSKIFGSFVTFLTSKDPSDGSDKALLNELESLENHLKTRPGPFVAGEKLTA
VDLSLAPKLYHLEIALGHYKKWSVPESLTNVRSYTNALFSRDSFEKTKAKKEFVVAGWASKVNK
>Brara.H02367
MALEVCVKAAVGAPDVLGDCPFSQRVLLTLEEKSLPYKMHLINLSDKPKWFLDINPGGKVPVLKIDGKWVPDSDV
IVSLLEKKYPEPSLKTPPKFASVGSKIMSTFVAFLTTKDSSDGPLLHELEALENHLKSHDGPFIAGEKVSAVDLS
LAPKLYHLEVALGHFKSWSVPGSLTHVHNYMHAVFSLHSFEKTKAEEKYVIAGWAPKVHY
>Brara.J01859
MISLRFQPSTAGVLSAPVGRAGLIKRFGLTKPRRTVRLGTVAMAAAPLEICVKASITTPNKLGDCPFCQRVLLTM
EEKHVPYDMKMVDLSNKPEWFLKINAEGKVPVVKFDEKWVPDSDVITHALEDKYPEPPLATPPEKASVGSKIFST

FIGFLKSKDPKDGTEQVLLDELSTFNDYLKENGPYINGEKISAADLSLAPKLYHMKIALGHFKNWSVPDSLPFLK
SYMENVFSRESYKNTEAQIEDVIAGWRPKVMG
>Brara.B00674
MIRLRFQPSTVGVLSASVNRAWLTRRYGTNEPGSTRGFGTSAMAASPLEICVKASVTTPNKLGDCPFCQRVLLTM
EEKHVPYDMKLVDLINKPEWFLKISPDGKVPLVKFNEKWVLDSDVITQSLEEKYPEPPLTTPPEKASVGSKIFST
FIGFLKSKDQGDGTEQALLNELSTFNDYLKENGPFINGEKISAVDLSLGPKLHHMKIALGHYKDWSVPDSLSFLK
SYMENVFSRESFAKTQAQAEDVIAGWRPKVMA
>Cagra.1961s0047
MALEICVKAAVGAPDHFGDCPFSHRAVLTLEEKNLTYKMHLINLSEKPQWFLDISPQGKVPVLKIDNKWVSDSDV
IVGLLDEKYPDPPLKTPPEFASVGSNIFGTFGTFLKSKDSNDSSEHALLHELEALENHLKSHEGPFIAGERVSAV
DLSLAPKLYHLQVALGHFKSWSVPSSLPHVHNYMNTLFSLPSFEKTKPEEKYVISGWAPKVNP
>Cagra.0402s0003
MALDICVKVAVGAPDVLGDCPFSQRVLLTLEEKELPYKIHLINVSEKPQWFLDISPEGKVPLMKLDGKWVADSDV
IVGLLEEKFPEPSLKTPPEFASVGSKIFGAFVTFLKSKDANDGSEKALVDELEALDNHLKTHSGPFVAGEKVTAV
DLSLAPKLYHLEVALGHYKKWSVPESLTNVRNYANALFARESFEKTKAKNEFVVAGWESKVNA
>Cagra.0434s0037
MISLRFQPCTAGVLSASVSRAGYIKQCGSTKPGRTFRFGTMAMAASPPLEICVKASITTPNKLGDCPFCQRVLLT
MEEKHVPYDMKMVDLSNKPEWFLKISPEGKVPVVKFDEKWVPDSDVITQALEDKYPEPPLATPPEKASVGSKIFS
TFIGFLKSKDSGDGTEQVFLDELSTFNDYIKDNGPFINGEKISAADLSLAPKLYHMKIALGHFKNWSVPDSLSFV
KSYMENVFSRESFTNTQAQTEDVIAGWRPKVMG
>Carubv10001758m.g
MISLRIQPCTAGVLSASVSRAGYIKQCGSTKPGRTFRFGTMAMAASPPLEICVKASITTPNKLGDCPFCQRVLLT
MEEKHVPYDMKMVDLSNKPEWFLKISPEGKVPVVKFDEKWVPDSDVITQALEDKYPEPPLATPPEKASVGSKIFS
TFIGFLKSKDSGDGTEQVFLDELSTFNDYIKDNGPFINGEKISAADLSLAPKLYHMKIALGHFKNWSVPDSLSFV
KSYMENVFSRESFTNTQAQTEDVIAGWRPKVMG
>Carubv10020944m.g
MALDICVKVAVGAPDVLGDCPFSQRVLLTLEEKELPYKIHLINVSEKPQWFLDISPEGKVPLMKLDGKWVADSDV
IVGLLEEKFPEPSLKTPPEFASVGSKIFGAFVTFLKSKDANDGSEKALVDELEALDNHLKTHSGPFVAGEKVTAV
DLSLAPKLYHLEVALGHYKKWSVPESLTNVRNYANALFARESFEKTKAKNEFVVAGWESKVNA
>Carubv10010286m.g
MALEICVKAAVGAPDHFGDCPFSHRAVLTLEEKNLTYKMHLINLSEKPQWFLDISPQGKVPVLKIDNKWVSDSDV
IVGLLDEKYPDPPLKTPPEFASVGSNIFGTFGTFLKSKDSNDSSEHALLHELEALENHLKSHEGPFIAGERVSAV
DLSLAPKLYHLQVALGHFKSWSVPSSLPHVHNYMNTLFSLPSFEKTKPEEKYVISGWAPKVNP
>evm.TU.supercontig_116.44
MGLPWKRCHHNLLTASAFSLASTHTNKARENVDRQIQPNAASVVCSTIKSLGFNIRLPQNCGISRRSHWTRQRTK
SFTVAMATPPLEVCVKASITIPNRLGDCPFSQRVLLTMEEKHLPYDMKLVDLRNKPEWFLKISPEGQVPVIKLDE
KWVSDSDVITQSLEEKYPEPPLQTPPEKASVGSKIFSTFIGFLKSKDPSDGTEQSLLNELSSFDAYIKENGPFIN
GEKISAADLSLGPKLYHLEIALGHYKKWSVPDSLSFVKSYMEKFFSMDSFIKTRAVPEDVIEGWRPKVMG
>evm.TU.supercontig_1.329
MAVEVCVKAATAAPNVLGDCPFCQRVLLTLEEKKVPYKTHLVNLSEKPQWFLEISPEGKVPVAKFDDKWVPDSDV
IVGMIEEKFPEPSLISPPEVASVGSKIFWAFFTFLKSKDPSDGSEQALINELKALDEHLAGHGPFIAGEKITAVD
LSLAPKLYHLEVALGHFKKWTVPESLTHLRGYTKLVFSRESFVKTNAAKEYVIAGWAPKVEA
>Cre10.g456750
MATPVTTIYVKGDPAKNKLLDCPFCHRVLLAYEAKKLPYKMEYIDFDNKPAWLLEASGGKVPVIKEGPDAPYMPD
SDVIVVHLEKQHPEPSLQSSVPAEIGAKLFPNFRAILIGPAAEVADKVAALEEQLAGMDDYLRQHEAQGPLFGGQ
HLNGTDCSLAPKLYHAVVALKHFKGWELPARFTALHKYLAALKALPEWQHVDYGTEAIIAGWERHIKHAAAGTGH
H
>jgi|ChlNC64A_1|143262|IGS.gm_5_00591
MASAAEPKYDIAVKAVGGKLADCPFCHRSLLTLEEKHVPYTKTFIDFANKPQWLLDVNPAGSVPVMKELATGEWI
VDSGTIQDYLEEKFPDPPLGTAEDSPQIGLDVLGKFGAFIKSSPEEAAEKEADLVECLRGLDAHLTAHGPFIGGA
APCATDCAVMPRLYHMQVGTKYFRDWEMPAELAALRQYMDTFMSRDSWKNTYYSPEMVIAGWEAHGIKKLVPAQ
>Ciclev10012501m.g
MSTLRIQPSTTSAISSTIKNLHLGFSFRLPCKNYGVSKRPRNLSVMAVATDPLEVCVKASSTSPNRLGDCPFCQR
VLLTIEEKHLPYDMKLVDLGNKPEWFLKISPEGKVPVIKLDEKWLPDSDVITQSLEEKYPDPPLGTPPEKASVGS
KIFSTFIGFLKSKDPSDGSQQALLNELNSFNDYIKENGPFINGGKVSAADLSLGPKLYHLEIALGHYKNWSVADS
LPHVKSYMKTIFSMDSFIKTRALPDDVIVGWRPKVFG
>Ciclev10032931m.g corrigée
MAVEICVKAAVGAPDILGDCPFSQRALLTLEEKKVPYKRHLINISDKPQWFMEISPEGKVPVVKFDDKWVADSDV
IVRIVEEKYPEPSLTNPPEFASLGSKIFPSFVNFLKSKDPNDGTEQALLEELKALDEHLKTHGGPFIAGEKVTAV
DLSLAPKLYHLQVALEHFKQWTVPESLAHVHGYMKKLFALESFQKTKAEKQYVIAGWAPKVNA
>orange1.1g040329m.g

MAVATDSLEVCVKASSASPNRLGDCPFCQRVLLTIEEKHLPYDMKLVDLGNKPEWFLKISPEGKVPVIKLDEKWL
PDSDVITQSLEEKYPDPPLRTPPEKASVGSKIFSMFIGFLKSKDPSDGSEQALLNELNSFNDYIKENGPFIIGGK
VSAADLSLGPKFYHLEIALGHYKNWSVPDSLPHVKSYMKTIFSMDSFIKTRALPDDVIVGWRPKVFG
>orange1.1g027907m.g
MSMKFLFEIFVDLCYPFFFSGSLGPFSQRALLTLEEKKVPYKRHLINISDKPQWFMEISPEGKVPVVKFDDKWVA
DSDVIVRIIEEKYPEPSLTNPPEFASLGSKIFPSFVNFLKSKDPNDGTEQALLEELKALDEHLKTHGGPFIAGEK
VTAVDLSLAPKLYHLQVALEHFKQWTVPESLAHVHGYTKKLFALESFQKTKAEKQYVIAGWVPKVNA
>estExt_Genewise1Plus.C_190281
MINSASRAIVRPVVGPTKAQIKNVPSLVKSSPTTRRGALRLCVAMATSPIIEAYVKGNPEAHERGDCPFSHRALL
TLAEKHVPYKEEYIDFSNKPKWLFDINPKGSVPIIHDLEEDSWIPDSAAIVDYLEERFPEPPLGKHDAPPHVGET
LFPSFVAALKAKKGTDEESKKIGTLVQSLEEINNYLKDNKKEYLGGARPNQADLGLAPKLYHVEHAMREYKGWEI
PDELTALKEYNQRIRSRESWKQTYYPPEKVVQGWKAHLEA
>fgenesh1_pg.22_#_14
MLKMHSSFVAFRYVKGSPEKGELGDCPFSHRTMLTLEEKGVPYNKLLLDELNMPEWIAEVTEGKSTIPFATELET
GKWLYDSDKIVPYLEDKFPERKLGKPDEVPQVGANLFPAFMEYVKTTDKADEDKKREALIAELKSIDSELKKSEG
PYVGGQDVNAADLKLGPQLKHVIIGSKAVKQWELPKELTAIHTFMEAIQKRESWKNTYYTEKYVADGWHKKLETM
GVK
>Cucsa.165630
MALEAAVKAAVGAPDEIGDCPFSQRVLLTLEEKKLPYKLHLINLSDKPSWFLKVSPEGKVPVVKFDDEWVPDSDV
IVETLEKKYPEPSLVTPPQFSSVGSKIFSAFTKFLKSKDPKDHSEQNLLEELKALDEHLKAHGPYVAGEKVTAVD
LSLAPKLYHVDVALGHFKKWCIPKDLACLISYKELLFARESFVKTKATPEHVIAGWEPKVNPA
>Eucgr.J01595
MSSAKITPTASVLSSTIKNLGFGLHLPSYTVLPSNFKQRYGCSQRRLCIMAKSLASNPLEVCAKASVTIPNKLGD
CPFCQRVLMTMEEKQLPYDLKLVDLGNKPEWFLKINSEGKVPVVKLDENWVADSDVITQSLEEKYPDPPLKTPAE
KASIGSKIFSTFIGFLKSKDSSDGTEQALLNELSSFNDHIKENGPFINGKEISAADLSLGPKLHHMEIALGHYKN
WSVPDTLPFVKSYMQSVFSRDSFVKTRALKEDVIAGWRPKVMG
>Eucgr.G02764
MALEICVKAAAGAPDILGDCPFCQRVMLTLEEKKVPYKTHLINFSEKPQWLLEVNPEGKVPLIKIDDKWIADSDV
IVGILEEKYPEPPLTPPPEFASVGSKIFISFVKFVKSKDPSDGTEQALLDELKALDEHLKAHGPYIAGEKITAVD
LSLGPKLFHLEVALGHFKKWTVPESFTHVHSYTKLLFARESFVKTKPAKEHVVAGWAPKVNGA
>Eucgr.G02889
MDPSSIKEGKPTKMIKRKRFTLPRRGEEGKPTKMIKIEGLEICVKAADGAPDFLGDCPFSQWVMLTLKEKKVPYK
THLINLSEKPQWLLEVNPEGKPLIKIDDKWIADSDVIVGILEEKYPEPPLTPPPEFASVGSKIFISFVEFVKSKD
PSDGTEQALLGELKALDEHLKAHGPYIAGKKITSVDLSVAPKLFHLEVALGHFKKWTVPESFTHFHSYTKLLFAR
ESFVKTKPAKEHVVAGWAPKVNEA
>Thhalv10014453m.g
MISLRLQPSTAGVLSASVSRAGFIKRCGSTKPGRIGRFRTMAMAATPLEICVKASITTPNKLGDCPFCQRVLLTM
EEKHVPYDMRMVDLSNKPEWFLQISPEGKVPVVKFDEKWVPDSDVITQSLEEKYPEPPLATPPEKASVGSKIFST
FIGFLKSKDPGDGTEQVLLDELSTFNDYIKENGPFINGEKISAADLSLGPKLYHMKIALGHFKNWSVPDSLSFVK
SYMENVFSRESFAKTRGQTEDVIAGWKPKVMG
>Thhalv10008761m.g
MALEVCVKAAVGAPDKLGDCPFSQRVLLTLEEKSLPYKIHLINISDKPQWFLDISPQGKVPVLKIDGKWVSDSDV
IVGILEDKYPEPSLKTPPRFASVGSQIFSTFVAFLKSKDSHDRTEHALLHELEALENHLKTHDGPFIAGERVTAV
DLSLAPKLYHLEVALGHFKSWSVPGSLPHVHNYMKALFSLDSFEKTKTEEKYVISGWAPKVHP
>Thhalv10019864m.g
MALDICVKVAVGAPDVLGDCPFSQRVLLTLEEKKLPYKTHLISVSDKPQWFLDISPEGKVPVVKLDGKWVADSDV
IVGLLEEKYPEPSLKTPPEFSSVGSKIFGAFVTFLTSKDANDGSEKALVDALEALENHLKTHPGPFVAGESVTAV
DLSLAPKLYHLMVALGHYKNWSAPESLTSVRNYAKALFNRESFEKTKAKKEFVVAGWASKVNK
>gene25477-v1.0-hybrid
MFLRLVLTRKPIRLCVPRVKHFEIRLDLICREVSTICSSISGRHRVSFSALLTLQGNNKMSTTTAARIHPTAAAL
SSTIKHHLLLRPSHRRHVVFRNNTSFSRRPMLTMASAPTPLQVCVKSSITVPNKLGDCPFCQRVLLTLEEKHLPY
ELKLVDLANKPDWFLKINPDGKVPVVNLDDKWIADSDIITQILEDKYPEPPLTTPPDKASVGSKIFSTFIGFLKS
KDPKDGTEQALINELSTFNDYLKLNGPFVNGDQVSSVDLSLAPKLYHMEIALGHYKSWSVPDSLPFLKSYIKSIF
SRDSFTKTSALKEDVIAGWRPKVLG
>gene23208-v1.0-hybrid
MALEVAAKAAAGAPELLGDCPFTQRVLLTLEEKKVPYKLHLINLADKPKWFTEVNPEGKVPVVKFDDKWVSDSDV
LVGILEEKYPEPCLQTPPEFASVGSKIFGSFVTFLKSKDPSDGSEQALLNELKALDDHLKAHGPYIAGEKVTAAD
LSLAPKLYHLKVALGHFKKWTVPKALTHYHKYTELLFSRESFAKTIPEDKYIIAGWEPKVNPK
>Glyma.18G040100
MSAVRVQVSACAVSATVNHLRLRQNAVVSFRKKKPLTLRVVSMSSVPPSQPFEIAVKASVTTPNRLGDCPFCQRV
LLTLEEKHLPYDPKLVDLTNKPEWFLKVNPDGKVPVIKFDEKWVPDSDVITQTLEEKYPSPPLVTPPERATAGSK

IFSTFIGFLKSKDPNDGTEQALLSELSSFNDYIKENGPFINGSEISAADLSLGPKLYHLEIALGHYKKWTVPDSL
TSLKSYMKAIFSRESFVKTSAQPQDVIEGWRPKVEG
>Glyma.11G216400
MSTVRVQVSACVFSATVNNLCLRQNAVVSFRKKKLLRLVSMSSVPPSQPFEIAVKASVTTPNRLGDCPFCQRVLL
TLEEKHLPYDPKLVDLTNKPEWFLKVNPDGKVPVIKFDEKWVPDSDIITQTLEEKYPSPPLLTPPEKATAGSKIF
STFIGFLKSKDPNDGTEQALLSELSSFSDYIKENGPFINGSEISAADLSLGPKLYHLEIALGHYKKWTVPDSLTS
LKSYMKVIFSRESFVKTSAQPQDVIEGWRPKVEG
>Glyma.20G240300
MALEVAVKAAVGAPNVLGDCPFSQRVLLTLEEKKIPYKLHLIDLSNKPEWFLGVNPEGKVPVVLFDGKWVADSDV
IVGILEEKYPEPSLITPPEFASVGSKIFGSFVSFLKSKDTNDGTEQALVAELSALDEHLKTHGPYIAGEKVTAVD
LSLAPKLYHLVVALGHFKNWNIPESLVHVHNYTKLLFSRESFEKTKPPKEEYVIAGWAPKVNA
>Glyma.10G291100
MALEVAVKAAVGAPNVLGDCPFSQRVLLTLEEKKIPYKLHLIDLSSKPEWFLGVNPEGKVPVVLFDGKWVADSDV
IVGILEEKYPEPSLVTPPEFASVGSKIFGSFVSFLKSKDTNDGTEQTLVAELSALDEHLKAHGPYIAGEKVTAVD
LSLAPKLYHLVVALSHFKNWNIPESLTHVHNYTKLLFSRESFEKTKPPKVEYVISGWAPKVNA
>Gorai.001G089300
MSTAVALSSSLKHNLALGIRFPQNPSFYRRNGPKVSTFRTFTVAMAAINTTPLEICVKASVSTPNKLGDCPFCQR
VLLTMEEKHLPYEMKLVDLSNKPEWFLQISPEGKVPVVKFDEKWVPDSDVIAQSLEEKYPNPPLVTPQEKALVGS
KIFSTFIGFLKSKDPSGGTEQALLDELSSFNDYIKENGPFINGVKISAADLSLAPKLYHLEIALGHYKKWSIPDT
LPYTKSYMKTIFSMDSFMKTRASPEDVIAGWRPKVMG
>Gorai.012G068600
MALEICVKAAVGAPNVLGDCPFSQRALLTLEEKKISYNMHLINISDKPQWFLEISPEGKVPVVKFDDKWVADSDV
IVGILEEKYPQPCLKTPPQFASVGSNIFGTFVTFLKSKDANDGSEQALLNELKALDEHLKAHGPFIAGEKISAVD
LALGPKLYHLEVALGHFKKWTVPESLTNVRNYMKSIFSRESFVKTRAAKEYVIAGWAPKVNA
>Gorai.011G246200
MALEICVKAAAGAPDILGDCPFCQRVLLTLEEKKVPYKLNLVNLSDKPQWFLEISPEGKVPVVKFDDKWVADSDV
IVGIIEDKFPEPSLKTPPEFAHVGSKIFGTFISFLKSKDANNGSEQDLVNELKALDEHLKGHGPFIAGEKITAVD
LGLGPKLYHLEITLGHFKKWTVPESLTYVHNYMKSIFGRESFVKTKAAKEHVIEGWAPKVNA
>Lus10009135.g
MSTARIQPAVAAVAASALSSTVKYSSFTFNCPSSTVFARRPSIRRTLTFAMSTSVSNPLEICVKASITQPTKLGD
CPFCQRVLLTMEEKRVPYDMKLVDLNNKPEWFLRLSPEGKVPVVNLEDKWVPDSDVITQSLEEKFPDPPLATPPE
KSSVGSKIFSTFIGFLKSKDPADGTEQALLSELSSFDDYIKQNGPYINGEKVSAADLSLGPKLYHLEIALVHYKN
WSVPESLPHVKSYMQTIFSMDSFVKTRALQEDVIAGWRPKANCPGQVPFTLVHGTVCCCTCICIYVDDTGVGSGD
LTFQGEWRSFSGWFAEMAQVLQWEGFFERRMAFGSVDLLIGPALNRFCRLNSTAIRKEYCNEA
>Lus10028510.g
MRAAFAAVGQFFWIGLSSPVMVCLKWWSMELLTLLSVFMSNPKLETSVLSICLSISTRHFTIPYGFGAAVRRSSI
RRTLTFAMSTSVSNPLEICVKASITQPTKLGDCPFCQRVLLTMEEKRVPYDIKLVDLTNKPEWFLKLSPEGKVPV
VNLEDKWVPDSDVITQSLEEKFPDPPLATPAEKSSVGSKIFSTFIGFLKSKDPADGTEQALLSELSSFDDYIKQN
GPYINGEKVSAADLSLGPKLYHLEIALGHYKNWSVPESLPHVKSYMQTIFSMDSFVKTRALQDDVIADVDDTGVG
SGDLMFQGEWFAEMAQVLQLEGFFERRMTFGSVDLLMGPALNRFCRLNSTLLEMVVVGSVVGVHTGGGRDRLLTG
CIPSQFEHYLQSPPLLFDIRTIRVGGWNCKIRHVRREANLTRMFLPSWVMRLAKGKCGSPILPRKSCESINIINK
>Lus10023442.g
MVLKLEIAVKGAVGAPHLQGDCPFSQRVQLTLEEKNVSYNPHFINIADKPKWFLEINPGGKVPVVKFDEKWVSDS
DVIVGILEEKYPEPSLATPPEFASVGLKIFDSFVSFLKSKDSSDGTEEALVNQLKALNDHLKTHSPFIAGEKISA
VDLSLAPKLYHLETALAHFKKWSIPEELTHVHSYLKALFSRESFQKTKAEKQYVIAGWAPKVNP
>Lus10040320.g
MALKLEIAVKGAVGAPHLQGDCPFSQRVQLTLEEKNVPYNPHFINIADKPKWFLEINPGGKVPVVKFDEKWVSDS
DVIVGILEEKYPEPSLVTPPEFASVGSKIFDSFVNFLKSKDSSDGTEEALVNQLKALNEHLKTYSPFIAGEKISA
VDLSLAPKLYHLETALAHFKKWSIPEELTHVHSYLKALFSRESFQKTKAEKQYVIAGWAPKKTRVNKKDMAVEIA
VKAAAGAPDLQGDCPFCQRVQLTLEEKKVAYKLHLVNLSDKPQWFLDISPEGKVPVVKFDDKWVADSDVIVGILE
EKYPEPSLVTPDEFSSVGSKIFISFINFLKSKDLSDGTEQALVEQLKALDEHLKAHGPFIAGEKITGVDLSLAPK
LYHLETALGHFKKWSIPQDLTHVHSYLKTLFTRESFEKTRPEKEYVIAGWAPKDANYEFVDEDADTSNCFIFWPE
PNNYIHASFRAKMADDGPGKSKLFFAEATFRKDRLGD
>Lus10023441.g
MAVEIAVKAAAGAPDLQGDCPFCQRVQLTLEEKKVAYKLHLVNLSDKPQWFLDISPEGKVPVVKFDDKWVADSDV
IVGILEEKYPEPSLVTPDEFSSVGSKIFISFINFLKSKDSSDGAEQALVEQLKALDEHLKAHGPFIAGEKITGVD
LSLAPKLYHLETALGHFKKWSIPQDLTHVHSYLKTLFTRESFEKTKPEKEYVIAGWAPKVNA
>MDP0000156763
MLTTAKIHPAASAVLSSSIKHHLRPPPNAVVFRTNPNSLRRRGTTRTLTVAMVAPLDVCAKASTTVPNKLGDCPF
CQRVLLTLEEKHLPYDLKLVDLGNKPEWFLKINSEGKVPVVKLDEKWVADSDIITQALEEKYPEPXLATPPEKAS
VGSKIFSTFIGFLKSKDPKDGTEQALLNELSSFNDYLKENGPFINGKEVSAADLSLGPKLYHLEIALGHFKDWSI

PDSLPYVKSYMKSIFSLDSFVKTSALKEDVIAGWRPKVYTLTLPVLYLNQVNHFCIIDLVYWKSHCQMREYTEFI
DSGGKKKNKKVIERV
>MDP0000240690
MSTTARIHPTASAVLSXTIKHHLRPPPNAVVFRTNPNSLRRRGTTRTLTVAMAAPLDVCAKASVTVPNKLGDCPF
CQRVLLTLEEKHLPYDLKLVDLGNKPEWFLKXYPEGKVPVVKLDEKWVADSDIITQALEEKYPDPPLATPPEKAS
VGSKIFSTFIGFLKSKDAKDGTEEALLNELKSFDDYLKENGPFINGKVVSAADFSLGPKLYHLEIALGHFKDWSI
PDSLPYVKSYMKSIFSLDSFVKTSALKEDVIAGWRPKVLG
>MDP0000175246
MLTTAKIHPAASAVLSSSIKHHLRPPPNAVVFRTNPNSLRRRGTTRTLTVAMVAPLDVCAKASTTVPNKLGDCPF
CQRVLLTLEEKHLPYDLKLVDLGNKPEWFLKINSEGKVPVVKLDEKWVADSDIITQALEEKYPEPXLATPPEKAS
VGSKIFSTFIGFLKSKDPKDGTEQALLNELSSFNDYLKENGPFINGKEVSAADLSLGPKLYHLEIALGHFKDWSI
PDSLPYVKSYMKSIFSLDSFVKTSALKEDVIAVYWKSHCQMREYTEFIDSGGKKKNKVIERV
>MDP0000530903
MSTTARIHPTASAVLSXTIKHHLRPPPNAVVFRTNPNSLRRRGTTRTLTVAMAAPLDVCAKASVTVPNKLGDCPF
CQRVLLTLEEKHLPYDLKLVDLGNKPEWFLKXYPEGKVPVVKLDEKWVADSDIITQALEEKYPDPPLATPPEKAS
VGSKIFSTFIGFLKSKDAKDGTEEALLNELKSFDDYLKENGPFINGKVVSAADFSLGPKLYHLEIALGHFKDWSI
PDSLPYVKSYMKRQPKIPTTIRRQPPLWTRFRRKPDKISSEADQFERDFLGRIMRVKRQKRHRKSVRFYTACYGF
RQPYKVLCDVTFVHHLVTNRITPADKAISNVLGAPVLLFTTNCAIAELKQHGPLLGPSYSQSLEAANSLITARCD
HESHVSAEDCILDVVGQSNSEHFFVATQHVDLRRKLLKHALTETSFHLQIPGVPAIYALRTALLLESPSDAQHQF
VKASEEQRLHMTDLEYKLLKKKKNIPNSLKINDPPGEEDGSGDQNMEAQAVAKRDXARIGLGVKDKVQFKRKRAK
ERKDGDATLRSRKKRSRSRKGKKTXEADG
>MDP0000311865
MALEVAAKAAAGAPDLLGDCPFCQRVTLTLEEKKVPYKLHLINLSDKPKWFTEVNPEGKVPVVKFDDKWVPDSDV
IVGIIEEKYPEPSLKTPPEICFCSKDPGDGSEQALLTELKALDEHLKAHGPYIAGEKVTAADLSLAPKLYHLKVA
LGHFKKWTVPADLAHYHKYTELLFSRESFVKTAPADEKYVIAGWEPKWWRLILEPNSLWARVIKARYFCKKGARA
LWAWSNLLTGRELLAGGSHWQIMGGGLSVASGSIFRFLGSSPACSSFDSEKFVDNYLAVRVKDFGALRSMVGPRE
AMGFAGVVARTAIGSFTAAVRFSLMAHSSLVAESLAILRGYELGATLGFSSVIIESDSLQAISCLNGSLENGSWE
AFPILARAQRYATLLSPSMASMATLFVNTPYLSHSFPKPSSAHFAHVLKLSTHLGSKSTRSLAKEEVESRDSLKE
LLVESRDVVVSEEKE
>MDP0000316839
MALEVAAKAAAGAPDLLGDCPFCQRVTLTLEEKKVPYKLHLINLSDKPKWFTEVNPEGKVPVVKFDDKWVPDSDV
IVGIIEEKYPEPSLKTPPEFASVVSNETTGPYIAGEKVTAADLSLAPKLYHLKVALGHFKKWTVPADLTHYHKYT
ELLFSRESFAKTAPAEEKYVIAGWKPKVFLPFVVEDIKVLIPSITEVPFTHIRRQANGPAHRLARLGLSVSQCCE
WLGSPPILVSYWIVLCPE
>MDP0000146156
MALEVAVKAAFGAPHLLGDCPFSQRVLLTLEEKKVPYKLHLINLSDKPSXILLCFEIFIPKIHAGIDGNMFTEVN
PEGKVPVVKFDDKWVADFDVIVRIIEENYPEPSLKIPSKFASVYDLSLSLFLILLRKYIFVVSSKRLFLLLENGA
DANYRNYCGQTALMQACRYGHWEVVQTLLLFRCNVGLCRGNAMSVFKDFFQIFRYVVCTLSPTIHQSILSCLDIP
LARYLWILDRIFSR
>MDP0000942136
MALEVAVKAAFGAPHLLGDCPFSQRVLLTLEEKKVPYKLHLINLSDKPSAILLCFEIFIPKIHAGIDGNMFTEVN
PEGKVPVVKFDDKWVADFDVIVRIIEENYPEPSLKIPSKFASVLFLLLENGADANYRNYCGQTALMQACRYGHWE
VVQTLLLFRCNVGLCRGNAHVCLLRISSRYLDMWSAL
>cassava4.1_013773m.g
MSTTRLQPTTAFAASAACALSSTVKNHLGFTLRLPRNNSGFRRHSVTRRNLSVFAMSAASDPLEVCVKASLTVSN
RLGDCPFCQRVLLTMEEKHLPYDMKLVDLANKPEWFLKLSPEGKVPVIKLEDKWVPDSDVIAQSLEEKFPDPPLG
TPPEKASVGSKIFSTFIGFLKSKDASDGTEQALLNELSALNDYIKTNGPFINGEKVSASDLSLGPKLYHLEIALG
HYKQWSVPESLPHVKSYMKAIFTLDSFVKTRALPEDVIAGWRPKVMG
>cassava4.1_016102m.g
MALEICVKAAVGAPDVIGDCPFSQRALLTLEEKKLSYKLHLINLSDKPQWFLEISPEGKVPVVKFDDKWVPDSDV
IVGTLEEKYPEPSLVTPPEYASVGSKIFPSFVKFLKSKDANDGSEQALLGELEALEEHLKAHGPFIAGEKITAVD
LSLAPKLYHLEIALGHFKKWKVPENLPHVHKYMELLFSRESFQKTKAAKEHVILGWEPKVNA
>Medtr3g066060
MSTVRIQVSACALSATVNHLRYRPNYAVSTSSFNNHFSIKPLKVSMSSAPPSEPLEVAVKASLTTPNKIGDCPFS
QRVLLTLEEKHLPYEPKLVDLRNKPEWFLEISPEGKVPVINFDGKWVADSDLITQTLEEKYPSPPLVTPPEKATA
GSKIFSTFIGFLKSKDPNDGTEQALLNELSSFNDYLKENGPFINGKDISAADLSLGPKLYHLEIALGHYKKWTVP
DSLTFLKSYLKEIFSRESFINTRAQPEDVIEGWRPKVEG
>Medtr1g115500
MALEVAVKAAVGAPTILGDCPFSQRVLLTLEEKKIPHNIHLINLTDKPQWFLEVNPEGKVPVVKFDGKWVPDSDV
IVGILEDKYPEPSLVSPAQFSSVGSNIFASFSSFLKSKDSNDGTEQALLAELNALDEHLKANGPFVAGEKVTAVD
LSLAPKLYHLVVALRHFKSWTIPESLAKVHNYIKLLFSRESFEKTKAAEEYIIAGWAPKVNA

>mgv1a012701m.g
MCRGGHSGRVGLFGLFGSGYSGFLQKKNYPNPTRDNRVPGCRVPGYFGSGPFTQRVLLTLEEKRLPYDLKLVNLN
NKPEWFLSISPEGKVPIIKLGEKWIPDSDVITQALEEKFPEPSLATPPEKATVGSKIFSTFIGFLKSKDSSDGTE
QALLNELTAFNDYLQENGPFINGELISAADLSLAPKLYHLEIALGHYKKWSIPDSLTYVNSYMKTIFSKDSFVNT
RAEPKDVIEGWRPKVEG
>mgv1a013716m.g
MAVEICVKAAAGAPDILGDCPFSQRVQLTLEEKKVHYKLHLINTDDKPQWFLEVNPEGKVPVIKFDDKWIADSDV
IVGIIEEKYPNPSLSPPTEVSSVGSKVFPSFVKFLKSKDSTDGSEQALIDELKALDEHLSTKGPYVNGENICAVD
LSLAPKLYHLDVALGHFKKWSVPESLTHVHNYMKLLFSRESFQKTKAAKEFVIAGWAPKVNA
>LOC_Os06g12630
MAVLLRTTTSATTATSGGSSSATALLATTFRRGGRRLLLLPATRGSAPRRAALLTARASAEPLEVCAKASLTVPD
RLGDCPFTQRVLLTIEEKHLPYDIKLVDLANKPDWFLKISPEGKVPIVKLEEQWVADSDVITQAIEEKYPEPSLA
TPPEKASVGSKIFSTFIGFLKSKDPNDGTEQALLSELTSFDSYLKDNGPFINGETISAADLSLAPKLYHMEIALG
HYKNWSVPDSLSHVKKYMKTIFSMDSFVKTIALQEDVIAGWRPKVMG
>LOC_Os05g02530
MGRHVRMTITLPSPSRIPKPPEHGGTASWAPHVILTPSVSGELPLPINPASPISPVISPPIARRRRNPKSSLPVR
EKRQVVVAAMGVEVCVKAAVGHPDTLGDCPFSQRVLLTLEEKKVPYEMKLIDVQNKPDWFLKISPEGKVPVFNGG
DGKWIPDSDVITQVIEEKYPTPSLVTPPEYASVGSKIFSCFTTFLKSKDPNDGSEKALLTELQALEEHLKAHGPF
INGQNISAADLSLAPKLYHLQVALEHFKGWKIPEDLTNVHAYTEALFSRESFIKTKAAKEHLIAGWAPKVNA
>Pavirv00044899m.g
MTVEVCVKAAAGAPDTLGDCPFSQRVLLTLEEKKVPYEMKLVDLGNKPEWFLKISPEGKVPVFNSGDDKWIADSD
VITQVIEEKFPTPSLVTPPEYASVGSKIFPSFVKFLKSKDASDGSEKALLDELQALDEHLKAHGPYINGENVSAA
DLNLGPKLFHLQVALEHFKGWKIPENLTSVHAYTKALFSRESFVKTKPAKEHLIAGWAPKVNA
>Pavirv00033843m.g
MTVEVCVKAAVGAPDTLGDCPFSQRVLLTLEEKKVPYEMKLVDLSNKPEWFLKISPEGKVPVFNSGDGNWIADSD
VITQVIEEKFPTPSLVTPPEYASVGSKIFPSFVKFLKSKDASDGSEKALLDELQALDDHLKAHGPYINGENVSAA
DLSLGSKLFHLQVALEHFKGWKIPENLTSVHAYTKALFSRESFVKTKPAKEHLIAGWAPKVNA
>Pavirv00014947m.g
MAVEISYEEFLAGPFTQRVLLTIEQKHLPYDLKLVDLANKPDWLFEINPEGKVPIVKVEDKWIADSDVITQALEE
KYPEPSLATPADKASIGSKIFSTFIGFLKSKDPSDGTERALLDELTSFDTYLKDNGPFINGGAVSAADLSLGPKL
YHMEIALGHYKSWSVPDSLSHVKQYMKSIFSMDSFVKTQALQEDVIAGWRPKVMG
>Phvul.001G230400
MSTARVQVSACALSTAVNHLRLRPNAVVSFTNHFRRKSLRVVSMSSVPPSQPFEIAVKASLTTPNRLGDCPFCQR
VLLTLEEKHLPYEPKLVDFTNIPEWFRTVNPDGKVPVIKFDEKWVPDSDVITQTLEEKYPSPPLVTPPEKATVGS
KIFSTFIGFLKSKDPNDGTEQALLSELSSFNDYIKENGPFINGNEISAADLSLGPKLYHLEIALGHYKKWTVPDS
LTSLKSYTKAIFLRESFIKTSAQPQDVIEGWRPKVEG
>Phvul.007G009800
MAVEVAVKAAVGAPTVLGDCPFSQRVLLTLEEKKIPYKLHLIDLSNKPEWFLGVNPEGKVPVALFDGKWVSDSDV
IVGILEEKYPEISLITPPEFATVGSKIFGSFVTFLKSKDPNDGTEQALLAELSALDEHLKANGPYVAGEKVTAVD
LSLAPKLYHLVVVLGHFKKWSVPESLAHVHNYTKLLFSRESFEKTKPPKEEYVIAGWAPKVNG
>Pp1s12_401V6
MRALCGVPSCASLSSHRLASPLLAYRACEFALGSSPGLRLEFVGRAVGLQSRSIGRRCIVAMAAAAAEAAPTEVF
VKAAVGHPDKFGDCPFSHRVVLTLAEKKVPYDMKLIDVSNKPQWFLDINPEGKVPVIKDEGKFVADSDVITQLLE
EKYPEPCLKTPEDKASAGARIFPNFAAFLKSKDPNDGTEAALLAELKSLDEHLKSNKPFIAGEAVTAADLALAPK
LHHLTVALGHYKKWSIPEDLTNVLSYVEAVHSLESFKKTKPADEFIIAGWAKFFV
>Pp1s241_65V6
MASRCQRLKVYVKAATGNPCKLGDCPFSQRVLITCELKNIAYDVKFVDLDRKPEWFLRINPEGRVPVIKINGDYI
PDSDIIVDVLEKSYPYPPLSTCRNITCRGQNIFPAGMAFFKSKNPRCDGTESQFVCELDHMNHHLCNEGPYIAGQ
YVTSADIALAPQLYVLQTALAYYKNWTNFEQFYPALNLFMKVYSHKYHLKTHARPSSQPNKKPPPH
>Pp1s271_8V6
MPLPVAYEGRSPLGICMPRNVCSFLFGPRFNVHHSPPPTSRTNSNWVQRYSEIINMLRQQALLTRAYATLCPSFN
SITKFRPIVQVPSTGLASKVPFQGTKVSVPKSYVTSKGRAYAMAANAGLDLEAFGKAKSGSGSPSKERGDCPFSQ
RIYIELEEKKLPYTATYIEEGENKPDWFMEKNPKGLMPVLRDGDEWIQDSDKIAEHLEKKYPEVSLATPKEYKQI
GLNIFQAFTTYLKSKNADDQSKQELLKELAALDQHLQTKGPYIAGENPTDSDYALIPKLHHMRVSLAHYMGFKIP
SEHKALHKYIKLLESRPSFQKTNSPDDMIIEGWQKKFGLPDRVVEPASA
>Potri.017G125100
MSTARIQPTVTAACVLSSTIKHHFGLTSLRLPTNHSAAFRRQRIKRNLTVSMSATSVSEPLEICVKASLTVPDKL
GDCPFCQRVLLTLEEKNLPYDMKFVDLGNKPEWFLKLNPDGKVPVIKFEENWVSDSDVITQALEEKFPDPPLAIP
PEKASVGSKIFSTFIGFLKSKDPGDGTEQALLDELSAFNDHIKENGPFINGEKVSAADLALGPKLYHLEIALGHY
KNWSVPESLPYIKSYLKEIFSRDSFVNTRALPEDVIAGWRPKVMG
>Potri.008G049300

MALEICVKAAVGAPNILGDCPFCQRVLLSLEEKKIPYKSYLINLGDKPQWFLEISPEGKVPVVKIDDKWVADSDV
IVGILEEKNPEPPLATPPEFASVGSKIFPSFVKFLKSKDPNDGTEQALLEELKALDGHLKVHGPFIAGEKITAVD
LSLAPKLYHLEVALGHFKNWTIPDNLTHVLNYIKLLFSRESFKKTRAAEEHVIAGWEPKVNA
>Potri.010G211600
MVLEIAVKAAVGAPDILGDCPFSQRALLTLEEKKIPYKSHLINLSDKPQWFLEVNPEGKVPVVKFDDKWVSDSDV
IVGILEEKYPEPSLATPPEFASVGSKIFPSFVKFLKSKDPNDGTEQALLEELKALDDHLKAHGPFIAGEKITAVD
LSLAPKLYHLEVALAHFKNWTIPDKLTHVLNYIKLLFSHESFEKTKAAKEHIIAGWEPKVNA
>ppa010038m.g
MSTTARIHPAASTVLSSTIKHHLLRPPPNAVVFRTNPNSVRRRGTIRTLTVAAAAPFEVCAKASVTVPNKLGDCP
FCQRVLLTLEEKNLPYDLKLVDLANKPEWFLKINPEGKVPVINLNEKWVADSDVITQALEEKYPDPPLAAPPEKA
SVGSKIFSTFIGFLKSKDPKDGTEQALLNELSSFNDYLKENGPFINGKEVSAVDFSLGPKLYHLEIALGHFKDWS
VPDSLPYVKSYMKSIFSLDSFVKTSSLKEDVIAGWRPKVLG
>ppa011390m.g
MALEIAVKAAVGAPDVLGDCPFCQRVLLTLEEKKVPYKFHLISFSDKPKWFTEVNPEGKVPVVKFDDKWVADSDV
IVGIIEEKYPEPSLKTPPEFASVGSKIFGSFVTFVKSKDPSDGSEQALVNELKALDEHLKAHGPYIAGEKITAAD
LSLAPKLFHLKVALGHFKKWTVPEDLTSYYKYTELLFSKESFVKTKTEEKYVIAGWESKVNP
>28524.t000001
MSTARIQSMPAAAVAAATCAFSCTMMKHHHLRFPRNNAVLRGRNRARKIAMSAVSDPLEVCVKASITVPNKLGDC
PFCQRVLLTMEEKHVPYEMKLVDLSNKPEWFLKISPEGKVPVIKFEEKWVPDSDFITQSLEEKFPDPPLGIPPEK
ASVGSKIFSTFIGFLKSKDASDGTEQALLNELSAFNDYIKENGPYINGEKVSAADLALGPKLYHLEVALGHYKNW
SIPESLPYFRSYMKAIFSLDSFIKTRALPEDVIAGWRPKSWGNVSPLPL
>29939.t000007
MTLEICVKAAAGAPDVLGDCPFCQRVQLTLEEKKVPYKLNLINLSDKPQWFLEISPEGKVPVIKIDDKWVADSDV
IVGILEKKYPEPSLVTPPEFASVGSKIFPTFIKFLKSKDPNDGSEQALLDELKALDEHLKTHGPFIAGEKITALD
LSLAPKLYHLEVTLGHFKKWTVPEDLTHVKNYLMLFSRESFQNSKASKEHMIAGWEPKVNA
>29939.t000003
MALEICVKAAVGAPDTIGDCPFSQRALLTLEEKKIPYKCNLINLSDKPQWFLQISSEGKVPVLKVDDKWVPDSDV
IVGLLEEKYPVPSLVTPPEFASVGSKIFPAFVKFLKSKDANDGSEQALLEELKALDEHLKAHGPYVAAEKITAVD
LSLAPKLYHLEVALGHFKKWTVPSDLTYVNNYIRLLFSRESFQKTKASEEHVIAGWEPKVNA
>271409
MAVATTEAVEVLVKAANGDPSRLGDCPFSQRVLLTLEEKGIPYNSKFVDMENKPAWFLEANPEGKVPVIKDDGKW
VADSDVITQLIDTKFPSPSLVTPPEKSSVGSKIFSSFVKFLKSKDPSDGSEAALLEELKALDEYLAKNGPFVNGS
NISAVDLSLAPKLYHLKIALGHYKQWSVPENLTNLNSYMEALFKRESFQKTMAPAEVVVKGWAKHLSA
>139875
MASQDVDLEVFGKAATGTGSPSNQRGDCPFSQRVYMVLEEKHLPYKATYVEEGPNKPDWFMQHNPSGLMPVLRDA
ADWIQDSDKIFEHVENKFKEPSLKTPDEFKSVGAGIFPAFTNWLKSKDRNAPAKQEFINELTALEEHLKKHGPYI
AGKNPTDSDFALAPKLRHARVALKHFIDFVFPSNLQHVAKYIELMETRPSFKKTDSPDEMIIAGWQTKFDLPDKI
DKNSGQEKKTAVTPLV
>Si008384m.g
MAILLRGTSAAAAATAGPSSTLLATTFRRARGCGRLLPAAPRLRRAFAARASAQPLEVCAKESITVPGRLGDCPF
TQRVLLTIEEKHLPYDLKLVDLANKPDWLFEMNPEGKVPIVKLEDKWIADSDVITQALEEKYPEPPLATPLDKAS
VGSKIFSTFIGFLKSKDPSDGTEQALLDELTSFDSYLKDNGPFINGGTISAADLSLGPKLYHMEIALGHYKNWSV
PDSLSHVKQYMKSIFSMDSFVKTRALPEDVIAGWRPKVMG
>Si023201m.g
MAVEVCVKAAVGAPDSLGDCPFSQRVLLTLEEKKVTYEMKLIDLSNKPEWFLKISPEGKVPVFNGGDGKWIADSD
VITQVIEEKFPTPSLVTPPEYASVGSKIFPSFVKFLKSKDASDGSEKALLDELQALDEHLKAHGPYINGENVSAA
DLSLGPKLFHLQIALEHFKGWKIPENLTSVHAYTQALFSRESFVKTKPTKEHLIAGWAPKVNA
>Solyc11g011250.1
MSTAKITPSAASFATSIKHLAGIQLPRCQSTIFTSNSTKFRAPRRGFTVSMAASIETPLEVCVKQSITTPNKLGD
CPFTQRVLLTLEEKHLPYDMKFVDLSNKPDWFLKISPEGKVPLIKLDEKWVPDSDVISQALEEKFPKPPLTTPPE
KASVGSKIFPKFVAFLKSKDSGDGTEQALLDELTAFNDYLKENGPFINGNEVSAADLSLGPKLYHLEIALGNYKN
WSIPDSLSYMKSYMKSIFSRESFINTRALKEDVIEGWRPKVMG
>Solyc05g054760.2
MVVEVCVKAAVGAPDVLGDCPFSQRVLLTLEEKKVTYKKHLINVSDKPKWFLEVNPEGKVPVINFGDKWIPDSDV
IVGIIEEKYPNPSLIAPPEFASVGSKIFPTFVSFLKSKDSSDSTEQALLDELKALEEHLKAHGPYINGQNVCSVD
MSLAPKLYHLEVALGHFKKWSVPESLSHVRNYMKLLFERESFQKTKAEEKYVIAGWAPKV
>PGSC0003DMG400015691
MSTVKITPSAASFATSIKHLAGIQLPRLQNTIFTSNSTKFRAPRRAFTVSMAASLDTPLEVCVKQSITTPNKLGD
CPFTQRVLLTLEEKHLPYDMKFVDLSNKPDWFLKISPEGKVPLIKLDEKWVPDSDVITQALEEKFPEPPLTTPPE
KASIGSKIFPKFVAFLKSKDPTDGTEQALLDELTAFNDYLKENGPFINGNEVSAADLSLGPKLYHLEISLGHYKN
WSIPDSLSYVKSYMKSIFSRESFINTRALKEDVIEGWRPKVMG

>PGSC0003DMG400023416
MAVEVCVKAAVGAPDVLGDCPFSQRVLLTLEEKKVTYKKHLINVSDKPKWFLEVNPEGKVPVINFGDKWIPDSDV
IVGIIEEKYPNPSLIAPPEFASVGSKLFPTFVSFLKSKDSSDGTEQALLDELKALEEHLKAHGPYANGQNVCSVD
MSLAPKLYHLEVALGHFKKWSVPESLSHVRNYMKLLFERESFQKTKAEEKYVIAGWAPKV
>Sb10g008310
MAVLLRGTSAATSTVGPSSALLATTFRRSRGRLLPRAAPQRRLYVARASAQPLEVCAKESITVPGRLGDCPFTQR
VLLTIEEKHLPYELKLVDLANKPDWLFEINPEGKVPIVKLEEKWIGDSDVITQTLEEKYPEPPLATPPEKASVGS
KIFSTFIGFLKSKDPSDGTEEALLNELTSFDSHLKDNGPFINGGTISAADLSLGPKLYHMEIALGHYKNWSVPDS
LSHVKTYMKSIFSTDSFVKTQALPEDVIAGWRPKVMG
>Sb09g001700
MAAVEVCVKAAAGKPDTLGDCPFSQRVLLTLEEKKVPYEVKLVDLGNKPEWFLEINPEGKVPVLKGDDGKCIADS
DVITQVIEEKFPTPSLVTPPEYASVGSKIFPAFVKFLKSKDASDGSEKALLDELQALDEHLKAHGPYINGDNVSA
ADLSLAPKLFHLQVALEHFKGWKIPENLTNVHAYTKALFSRESFVKTKPSEEHVIAGWAPKVNA
>Sb09g001690
MAAGHKNTTTATLLVLLLSLMLSLTVSVARLSIATAVEVCVKAAVGAPDKLGDCPFSQRVLLTLEEKKVPYQLKL
IDLSNKPGWFLKISPEGKVPVYNGGDGKWIPDSDVITQVIEKKYPTPSLITPPEYASVGSKIFPSFVKFLMSKNA
KDGSEKALLHELQALELHLKAHGRPYISAKNVSAVDLSLAPKLFHLVVALEHFKHWKVPESLSNVHAYTKALFSR
ESFIKTKPTKEHLIAGWVAHGARVSPRAHGALLVPGGHPRLI
>Thecc1EG019159
MMSTASALSCSLKHHLAFGLRFPQNHALCRPNGARITTSRSLAVTMAATSTTPLEVCVKASVTTPNKLGDCPFCQ
RVLLTFEEKHLPYDMKLVDLANKPEWFLQISPEGKVPVAKLDEKWVPDSDVITQSLEEKYPDPPLVTPPEKASVG
SKIFSTFIGFLKSKDPTDGTEQALLNELSSFNDYIKENGPFINGEKISAADLSLGPKLYHLEIALGHYKKWSVPD
TLPYVKSYMETIFSMDSFVKTRASPDDVIAGWRPKVMG
>Thecc1EG042968
MALEICVKAAAGAPDVLGDCPFCQRVVLTLEEKKVPYKMHLVNLSDKPRWFLEISPEGKVPVVKFDDKWVPDSDV
IVGILEEKYPEPSLKTPPEFASVGSKIFGTFITFLKSRDANDGSEQALLNELKALDEHLKGQGPFIAGEKITAID
LSLGPKLYHLEIALGHFKKWTIPESLTCVHGYLKLIFSQESFVKTSVAKEFVITGWAPKVNA
>GSVIVG01030734001
MSSVRIPPTASAVSSTIKHLGYNPRFFTSHGGAKGVKRVGGERRKYSITMSSPLEVCVKASVIIPNKLGDCPFSQ
RILLTLEEKHLPYEMKLVDLTNKPEWFLKISPGGTVPVMKLDEKWIADSDVIAQSLEEKYPDPPLGTPPEKASVG
SKIFPAFIGFLKSKDPSDGTEQTLLNELASFDDYIKENGPFINGKDISAVDLSLGPKLYHLEIALGHYKKWTVPD
SLPFVKSYMKNIFSMESFVKTRGLPEDVIAGWRPKVMS
>GSVIVG01005966001
MSFEVCVKAAAGDPEILGDCPFSQRVLLTLEEKKVPYKMHLINVNEKPQWFLEMNPEGKVPVIKVDDKWVPDSDV
ITGVLEEKHPSPPLAPPPEHSSVGSKIFPAFVKFLKSKDPNDGSEQALLDELKALDDHLKDHGPYINGENICAVD
LSLAPKLYHLQVALGHYKNWTIPESLSHVHNYMKLLFSRESFEKTKPAPDHVVAGWAPKVNA
>jgi|Volca1|105142|estExt_fgenesh4_pg.C_240097
MANTVIYVKGDPANKTLGDCPFCHRALLTFERKKVPYTLDYIDFANKPSWLQDVSGGKVPVIKEDGQPYMPDSDV
IVVHLEEKYPEPSMKSSVPPEIGAKLFPAFRGVLMGPPEELADKQALLISELKAMNDYLEAHQAEGPLFGGHQIN
ATDAAVAPKLYHAVVALKHFKGWELPPEFAAVRRYMAAIQQLPEWKKTDYGEAMIIKGWERHMAHH
>GRMZM5G826194
MAVLLRSTSAAASTAGPSSALLATTFRRSRGCLLPRAAPQRRLYVARASAQPLEVCAKESITIPGRLGDCPFTQR
VLLTIEEKHLPYDLKLVDLANKPDWLFEINPEGKVPIVKLEEKWIGDSDVITQALEEKYPEPPLATPPEKASVGS
KIFSTFIGFLKSKDPSDGTEEALLNELTSFDSYLKDNVHWCRDPDQRGLHRAGPALRGRARRQRNDKDFATSRER
SPCHPWRRIERGALAKPPFSPAGQRASGDSIILRRRLFPYPLSSR
>GRMZM5G855672
MAAVEVCVKAAAGNPDTLGDCPFSQRVLLTLEEKKVPYEVKLVDLGNKPEWFLNISPEGKVPLFNGGDGKCIADS
DVITQVIEEKFPTPSLVTPPEYASVGSKIFPAFVKFLKSKDASDGSEKALLDELQALDDHLKAHGPYINGENVSA
TDLSLGPKLFHLQIALEHFKGWKIPENLTNVHAYTKALFSRESFVKTKPSEEHVIAGWAPKVNA
>GRMZM2G035502
MAAVEVCVKAATGKPDTLGDCPFSQRVLLTLEEKKVPYEVKLVDLDNKPEWFLKISPEGKVPVFNGGDGECIADS
DVITQTIEEKFPTPSLVTPVEYASVGSKIFPAFITFLKSKDASDGSEKALLDELQALDEHLKAHGPYINGENVSA
ADLSLGPKLFHLQVALEHFKGWKIPENLTNVHAYTKALFSRESFVKTKPSEEHVIAGWAPKVNA
>GRMZM2G005710
MAIGHKNTTKVALLVLLLSLMLSLTASVARNLQWRLSVATAVEVCVKAAVGAPDKLGDCPFSQRVLLTLEEKKVP
YRMRLIDLSNKPGWFLKISPEGKVPVYNSGDGKWIANSDVITQVIEEKYPAPSLATPPEYASVGSKIFPSFVKFL
MSKDASDDGSEEALVRELQALEEHLKAHGRPYISGERVTAADLSLAPKLFHLVVALEHFKGWKVPESMSSVHAYT
QAMFSRESFIKTKPTKEHLIAGWAAKVPKPPNLVVPCWLI

GHR class

>Aquca_030_00355
MICNLSKPHLWNKKICSFKNTFRMARSALDEMTDSGAFNRTPSTFRNLISRDPKSPFPAESGRYHLYISYACPWA
SRCISYLKIKGLDKSISVTVVKPIWERTKESDDHMGWVFPASSTEEPGAEPDLLNGARSIRDLYELASANYSGKF
TVPVLWDKKLKTIVNNESSEIMRMLNTEFSDIAENPALDLYPAHLQAQIDEVNEWVYDGINNGVYKCGFAKKQEP
YEEAATKLYETLDRCEVILSKQRYICGNSLTEADIRLFVTLIRFDEVYAVHFKCNKKLLREYPNLFNYTKDIFQI
PGMSDTVNMQHIKKHYYGSHPSINPFGIIPLGPDIDFSSPHDRQKFSS
>Aquca_034_00272
MATTTALLNHTFLLPTKNNKINTTLHKTNSRTCRVIPRMSLQPQPQPQPPNQDLLTSITKLLWGKSLPPQLLIST
VRTTWNATWHLMMKQLAPSDPSGSYTRPISKFRANFKSYSISDLKPGTLHLYVGLPCPWAHRTLLVRALKGLEEM
IPVSIASPSLDGSWEFRQTIGGHDNDVYLPGLDKANGRKILKEVYGLHRGGYDGRSTVPMLWDTKKNEVLCNESY
DIIEFFNGLESNDSNLDLAPPSLKGKIDEWNQIIYPNVNNGVYRCGFAQSQEAYDTAVNGLFTTMDMIDDHLASS
RYLCGDVITLADVCLFTTLIRFDLVYNVLFKCTKKKLLEYSNLYAYMRDIYQIPKVSATCNFEAIMDGYYKILFP
LNPGSIQPAMPSACDNKFLSKPHNREILSAMDKRLEAGIL
>329576
MSYSTIISNTSFLAFASKFTTRGSRLQCTLSMARSAVDETSDSGAFQRTASTFRNFVSRDSNSQFPAESGRYHLY
ISYACPWASRCLSYLKIKGLDDAISFSSVKPIWGRTKETDEHMGWVFPSSDTEVQGADPDHLNGAKSVRELYEIA
SPNYTGKYTVPVLWDKKLKTVVNNESAEIIRMFNTEFNHIAGNPDLDLYPSHLQAKIDETNEWIYNGINNGVYRC
GFAKKQGPYEEAVEQVYEALDRCEEILGKHRYICGNTLTETDIRLFVTLIRFDEVIDSSSYFQTKKKKHTICEQI
CNVETLMQVYAVHFKCNKKLIREYPNLFNYTKDIFQIPGMSSTVNMNHIKQHYYGSHPSINPFGIIPHGPNIDYT
SPHDRHRFSK
>915666
MARSGVDETSESGAFVRTASTFRNFVSKDPHSQFPAESGRYHLYISYACPWACRCLSYLKIKGLDEAISFSSVHA
IWGRTKETDDHRGWVFPDSDTELPGAEPDFLNGAKSVRELYEIASPNYEGKYTVPILWDKKLKTVVNNESSEIIR
MFNTEFNGIAKNPSLDLYPSHLRDTIDETNEWVFNGINNGVYKCGFARKQEPYNEAVNQLYEAVDRCEEILRKQR
YICGNTFTEADIRLFATLIRFDEVYSVHFKCNKRLLREYPNIFNYIKDIYQIQGMSSTVNMEHIKQHYYGSHPTI
NPFGIIPHGPNIDYSSLHDRDRFSS
>884156
MILCTSMANEKSDFTRTATSFRNFVSQKSDSQFPAESGRYHLYISYACPWASRCLAILKFKGLEKAISFSSVQPL
FKKTKESDEHMGWVFPDSDTEVLGAERDHLNGAKSVRELYDIASSNYTGKYTVPVLWDKKLKTIVNNESSEILRM
FNTEFNHVAENPSLDLYPPNLRAIIDETNEWIHDEINNGVYKCGFAKNQETYDVAVKQLYDALDRCEEILRNQRF
LCGNTLTEPDIRLFVTLIRFDEAYAVIFKCDKRLVREYYNLFNYTKDIYQIAGMSSTVKMDHIKQNYYGSFPSLN
PLEIIAPGPNIDYSLPHDRHRFSSESDYARLELLESASFVCEMSALLIEGSL
>494596
MANCFAPQLTFPSFAPRHVSPRMSHQSPKPSTSTTNKSIFTSATKLLWGPSLPPGLLISTARTAWTTVWQLMMTQ
LAPSDSSGSYTRPTSKFRLDPTQFPSAASSELHLYVGLPCPWAHRTVIVRALKGLNDAVTVSIASPGQDGSWEFK
DNNIPIKDKDKLIPSLDKANRCRNLKEVYKSRTGGYDGRCTVPMLWDSRKKEVVCNESYDIIEFFNSGLNELARN
ADLDLSPPELKEMIQDWNQIVYPKVNNGVYRCGFAQSQEAYDGAVNELFSTLDEIEDHLGSNRYLCGERLTLADV
CLFTTLIRFDPVYNVLFKCTKKKLVEYPNLYGYLRDMYQIPGVAATCDISAIMDGYYKTLFPLNASGIQPAISSS
GDQESLLRPHNRDLVGKAVEAQLAV
>AT4G19880
MSYSTIISNTSFLSLASKFTTRGSRLQCTVSMARSAVDETSDSGAFQRTASTFRNFVSKDSNSQFPAESGRYHLY
ISYACPWASRCLSYLKIKGLDDAISFSSVKPIWGRTKETDEHMGWVFPGSDTEVPGADPDHLNGAKSVRELYEIA
SPNYTGKYTVPVLWDKKLKTVVNNESAEIIRMFNTEFNHIAGNPDLDLYPSHLQAKIDETNEWIYNGINNGVYRC
GFAKKQGPYEEAVEQVYEALDRCEEILGKHRYICGNTLTETDIRLFVTLIRFDEVYAVHFKCNKKLIREYPNLFN
YTKDIFQIPGMSSTVNMNHIKQHYYGSHPSINPFGIIPHGPNIDYTSPHDRHRFSK
>AT5G45020
MARSGVDETSESGAFVRTASTFRNFVSQDPDSQFPAESGRYHLYISYACPWACRCLSYLKIKGLDEAITFSSVHA
IWGRTKETDDHRGWVFPDSDTELPGAEPDYLNGAKSVRELYEIASPNYEGKYTVPVLWDKKLKTVVNNESSEIIR
MFNTEFNGIAKTPSLDLYPSHLRDVINETNGWVFNGINNGVYKCGFARKQEPYNEAVNQLYEAVDRCEEVLGKQR
YICGNTFTEADIRLFVTLIRFDEVYAVHFKCNKRLLREYPNIFNYIKDIYQIHGMSSTVNMEHIKQHYYGSHPTI
NPFGIIPHGPNIDYSSPHDRDRFSS
>AT5G44990
MATPMENENPNFARTATSFRNFVSKDPDSQFPAESGRYHLYISYACPWASRCLAILKLKGLDKAISFSSVQPLWR
NTKENDEHMGWVFPDSDTEVLGAERDHINGAKSVRELYDIASSNYTGKYTVPVLWDKKLKTIVNNESSEILRMFN
TEFNHVAENPSLDLYPPNLRAIIDETNEWIHDGINNGVYKCGFATNQETYDVEVKRLYEALDRCEDILRKQRFLC
GNTLTESDIRLFVTVIRFDEAYAVIFKCDKRLVREYYHLFNYTKDIYQIAGMSSTVKMDHIKQNYYGSFPSINPL
EIIAHGPNIDYSLPHDRHRFSLESDYTRLELFESASFVCELKLIEIFDSL
>AT5G44000
MANCFAPQLTFPSFSPRHFSPRMSHQSPKPSTSTTTSIFTSATKLLWGPSLPPGLLISTARTAWTTVWQLMMTQL
APSDSSGSYTRPTSKFRLDPTQFTSAASSELHLYVGLPCPWAHRTLIVRALKGLNDAVPVSIASPGQDGSWEFKN
NNIPIKDKDKLIPSLDKANRCRNLKEVYKSRSGGYDGRCTVPMLWDLRKKDVVCNESYDIIEFFNSGLNKLARND

NLDLSPPELKEMIQGWNQIVYPKVNNGVYRCGFAQSQEAYDGAVNELFSTLDEIEDHLGSNRYLCGERLTLADVC
LFTTLIRFDSVYNILFKCTKKKLVEYPNLYGYLREIYQIPGVAATCDISAIMDGYYKTLFPLNASGIQPAISSSG
DQDSLWRPHNRDLVGKAIEAQLSV
>Bostr.30275s0459
MSYSTIIGNNSLLSLSRKFSTRGSRLQCTLSMARSAVDETSDSGAFQRTASTFRNFVSRDTNSQFPAESGRYHLY
ISYACPWASRCLSYLKIKGLDDAISFSSVKPIWGRTKETDEHMGWVFPGSDTEVQGADPDHLNGAKSVRELYEIA
SPNYTGKYTVPVLWDKKLKTVVNNESAEIIRMFNTEFNHVAGNPDLDLYPSHLQSKIDETNEWIYNGINNGVYRC
GFAKKEGPYEEAVEQVYEALDRCEEILGKHRYICGNTLTETDIRLFVTLIRFDEVYAVHFKCNKKLMREYPNLFN
YTKDIFQIPGMSSTVNVNHIKQHYYGSHPSINPFGIIPHGPNINYSSPHDRHRFSK
>Bostr.29514s0007
MARSGVDETSESGAFVRTASTFRNFVSQDPHSQFPAESGRYHLYISYACPWACRCLSYLKIKGLDDAISFSSVHT
IWGRTKDTDDHRGWVFPDSDTELPGAEPDYLNGAKTVRELYEIASPNYEGKYTVPILWDKKLKTVVNNESSEIIR
MFNTEFNGIAKNPSLDLYPSHLRDRIDETDEWVFNGINNGVYKCGFARKQEPYDEAVNQLYEALDRCEEILGKQR
YICGNTFTEADIRLFVTLIRFDEVYAVHFKCNKRLLREYPNIFNYIKDIYQIQGMSSTVNMEHIKQHYYGSHPTI
NPFGIIPHGPNIDYSSPHDRNRFSS
>Bostr.29514s0008
MAATSFRNFISQNPDSQFPAKSGRYHLYISYACPWASRCLIVLKLKGLDKAISFSSVQPLWRKTKESDEHMGWVF
PDSDTEVLGADPDHLNGAKSIRELYDIASSNYTRKYTVPVLWDKKLKTIVSNESSEILRMFNTEFNHVAENPSVD
LYPPSLQTIIDDTNEWIHDKINNGVYKCGFATNQETYDVAMRQLFNALDSCEDILGKQRFLCGNTLTESDIRLFV
TLIRFDEAYAVIFKCNKRLVREYYNLFNYTKDIYQIAGLSSTVKMDHIKQNYYGSFPSINPTEIIAHGPNIDYSL
PHDRHRFSSEIALRREYTSLEYARPFCELGLLLIEVFSRYLF
>Bostr.3148s0060
MANCFAPQLSFPSFTPRHGSPKMSHQSPKPSMSTTTSIFTSATKLLWGPSLPPGLLISTARTAWTTVWQLMMTQL
APSDSSGSYTRPTSKFRLDPTQFPFAASPELHLYVGLPCPWAHRTLIVRALKGLNDAVPVSIASPGQDGSWEFKD
NNIPVKDKDQLIPSLDKVNRCRNLKEVYKSRSGGYDGRCTVPMLWDSRKKEVVCNESYDIIEFFNSGLNELARNA
NLDLSPPELKEKIQDWNQIVYPKVNNGVYRCGFAQSQEAYDGAVNELFSTLDEIEDHLGSHRYLCGERLTLADVC
LFTTLIRFDPVYNILFKCTKKKLVEYPNLYGYLRDIYQIPGVAATCDFPAIMDGYYKTLFPLNASGIQPAISSSG
DQDSLLRPHYRDLVSKAVEAQLAV
>Bradi3g10950
MLGLVPSSSSYLLYRRLAAVRTFSAAAGSVNMAARSALDEVTDSGAFDRSPSTFRNFVSRDSSARFPAVPGRYHL
YVSYACPWASRCLAYLKLKGLDNAISFTSVKPIFERTRETDDHLGWVFPATGDEDPGADPDPFNGVKTVRELYEI
ASTSYTGKPTVPVLWDKQLKTVVNNESSEIIRMFNSEFNEIAENPGLDLNPPHLQASIDEVNDLVYDAINNGVYK
CGFAKKQEPYDEAVRNLYEALDKCEEILSKQRYMCGNQLTEADIRLFVTLIRFDEVYAVHFKCNKKLLREYPNLF
NYTKDIYQIPGISSTVNMEHIRKHYYGSHPSINPYRIVPAGPNIDYNATHDREKYSL
>Bradi3g55150
MWSPPPPLPLQLQLRQSPPTLPLFSHGRRLSRIAASQEDPLTALTRVLWGRALPPSQLVLAVRHGWTTAWRLLMR
QLAPSDPSTGAFTRTPSRFPAVVAAPSPTASLHLYVGLPCPWAHRALLVRALLGLHRRLPVSVAVPGDDGAWSFT
AASPDRLYGKCRLRDVYGAGTGEFEGRASVPMLWDADRRQVLCNESIEIAKFLCTLAGDDGDLDLYPPELRGEID
RWYGIIYPSVNNGVYRCGFAQSQQAYDAAAGELFAALDTLEEHLSRSRYLCSGDGLTLADVCLFTTLIRFDLVYN
PLFRCTRRKLVEYPSLHAYTREIYQMPGVADTCDMDAIAEGYFGTLFPLNPGGIQPVVPASCGREALMEPHGREA
LPSSAPAAAGRQLGAASSVS
>Bra012530
MSFSTILSHNSFLSLATKFTTRGSRLQCTIAMARSAVDETSDSGAFQRTASTFRNFVSRDSNSQFPAESGRYHLY
ISYACPWASRCISYLKIKGLDDAISFSSVKPIWGRTKESDEHMGWVFPDSDNEVEGAEPDHLNGAKSVRELYEIA
SPNYTGKYTVPVLWDKKLKTVVNNESAEIIRMFNTEFNHIARNPDLDLYPSHLQAKIDEANEWIYSGINNGVYRC
GFAKKQEPYEEAVQQVYEALDRCEEILGKHRYICGNTLTETDIRLFVTLIRFDEVYAVHFKCNKKLLREYPNLFN
YTKDIFQVPGMSSTVNMNHIKQHYYGSHPSINPFGIIPHGPNIDYSSPHDRHRFSK
>Bra039529
MARSGVDETSESGAFVRTASTFRSFVSQDPASQFPPESGRYHLYISYACPWACRCLSFLMLKGLDQAITFSSCHT
IWGRTKETDDHRGWVFPGSNSELPGAEPDYLNGAKTVRELYEIASPNYMGKYTVPILWDKKLKTVVNNESSEIIR
MLNTEFNGVAKNPSLDLYPSHLKEAIDETNEWVFNGINNGVYRCGFARKQEPYDEAVNQVFEAVDRCEALLGKQR
YICGNTFTEADVRLFVTLIRFDEVYAVHFKCNKRLLREYPNIFNYIKDVYQINGMSSTVKMDHIKQHYYGSHPTL
NPFGIIPHGPNIDYSSPHDRDRFSS
>Bra025096
MAASSANENSDKPRRTVTSFRNFVSQNHDSQFPAESGRYHLYISYACPWASRCLAVLKLKGLDKAITFTSVQPLW
RNTKENDEHMGWVFPDSDKEVPGAERDHINGAKSVRELYDIASLNYTGKYTVPVLWDKKLKTIVNNESSEILRMF
STEFNHFAENPCLDLYPPNLRSLINETNEWVHHGINNGVYKCGSATTQTAYDEAVKQLFDALDKCEEILQKQRFL
CGNTLTEPDIRLFVTLIRFDEAYTVIFKCNKRLIREYSNLFNYTKDIYQIPGLSSTVKMDHVKQNYYGSFPSINP
LGIVAHGPNIDYSLSHDRHRFS
>Bra033711

MSVCCASAQLTFPSHTTTPRRVSARMSNQPPKPTTTTTSIFTSATKLLWGPSLPPGLLISTARTAWSTVWHLMMT
QLAPSDSSGSYTRPTSQFRLNPTQFPSAASSELHLYVGLPCPWAHRTLIVRALKGLDDAVSVSVASPGQDGSWEF
KDNTIPIRDKDKLIPGLDKANRCRNLKEVYKSRTGGYDGRCTVPMLWDSRKKDVVCNESYDIIEFFNTGLNELAK
NPSLDLTPPELKGKIETWNRIVYPKVNNGVYRCGFAQSQEAYDRAVNELFSTLDEIEDHLGSNRYLCGERLTLAD
VCLFTTLIRFDPVYNILFKCTKKKLVEYPNLYGYLRDIYQIPGVAATCDIPAIMDGYYKTLFPLNASGIQPAISL
SGHQDSLLIPHNRDSVGKAFVAQNAV
>Cagra.2489s0006
MSYSTIISNNSLLSLATKFSTRGSRLQLQCTLSMARSAVDETSDSGAFQRTASTFRNFVSKDSNSQFPAESGRYH
LYISYACPWASRCLSYLKIKGLDDAISFSSVKPIWGRTKETDEHMGWVFPGSDSEVQGADPDHLNGAKSVRELYE
IASPNYTGKYTVPVLWDKKLKTVVNNESAEIIRMFNTEFNHIAGNPDLDLYPSHLQAKIDETNEWIYNGINNGVY
RCGFAKKQEPYEEAVEQVYEALDRCEEILGKHRYICGNTLTETDIRLFVTLIRFDEVYAVHFKCNKKLLREYPNL
FNYTKDIFQVPGMSSTVNMNHIKHHYYGSHPSINPFGIVPQGPNIDYTSPHDRHRFSK
>Cagra.1562s0038
MARSGVDETSESGAFVRTASTFRNFVSQDPHSQFPAESGRYHLYISYACPWACRCLSFLKIKGLDEAISFSSVHA
IWGRTKETDDHRGWVFPGSNTELPGAEPDYLNGAKTVRELYEIVSPNYEGKYTVPILWDKKLKTVVNNESSEIIR
MFNTEFNGIAKNPSLDLYPSHLRDRIDETNEWVFNGINNGVYKCGFARKQEPYNEAVNQLYEAVDRCEEILGKQR
YVCGNTFTEADIRLFVTFIRFDEVYAVHFKCNKRLLREYPNIFNYIKEIYQIQGMSSTVNMEHIKQHYYGSHPTV
NPFGIIPHGPNIDYSAPHDRDRFSS
>Cagra.1562s0037
MENLIFNQLGSCPSSVKTSELGSDSFEIKSASAMDMSITCENSDFPRDATSFRNFVSQKPDSIFPAKAGRYHLYI
SYACPWASRCLAILILKGLEKAISFSSVQPLWRRTKESDEHMGWVFPDSDTEVLGADPDRLNGAKSIRELYDIAS
PNYTGKYTVPVLWDKKLKTIVNNESSEIIRMFNTKFNHVAENPSLDLYPPNLQTIIDDTNEWIHDDINNGVYKCG
FATNQETYDVAVKALFHALDRCEEILRKQRFLCGNTLTETDIRLFVTLIRFDEVYAVIFKCGKRLVREYYNLFNY
TKDIYQMAGLSSTVKMDHIKQNYYGSFPSLNPLKIIPLGTNINYSWPHDRHRFSSEIPLRRDCARRELEHASLVC
ELSLILLEIFGRLF
>Cagra.13578s0004
MSHQSPKPSTSTTTSIFTSATKLLWGPSLPPGLLVTTARTAWTTVWQLMMTQLAPSDSSGSYTRPTSKFRLDPTQ
FPSVAASSELHLYVGLPCPWAHRTLIVRALKGLNDAVPVSIASPGQDGSWEFKDNNIPIKDKDQLIPSLDKANRC
RNLKEVYKSRSGGYEGRCTVPMLWDSRKKDVVCNESYDIIEFFNSGLNEVARNADLDLSPPELKEKIQDWNQIVY
PKVNNGVYRCGFAQSQEAYDGAVNELFSTLDEIEDHLGSNRYLCGERLTLADVCLFTTLIRFDPVYNILFKCTKK
KLVEYPNLYGYLRDIYQIPGVAATCDFPAIMDGYYKTLFPLNASGIQPAISSSGDQDSLLRPHCRELVSKAVEAQ
LTV
>Carubv10026527m.g
MENLIFNQLGSCPSSVKTSDIGSAPFEIKSASAMDMSNSDFARTATSFRNFVSQKPDSQFPAKAGRYHLYISYAC
PWASRCLAILILKGLEKAISFSSVQPLWRKTKESDEHMGWVFPDSDTEVLGADPDRLNGAKSIRELYDIASPNYT
GKYTVPVLWDKKLKTIVNNESSEIIRMFNTKFNHVAENPSLDLYPPNLQTIIDDTNEWIHDDINNGVYKCGFATN
QETYDVAVKALFHALDRCEETLRKQRFLCGNTLTETDIRLFVTLIRFDEVYAVIFKCGKRLVREYYNLFNYTKDI
YQIAGLSSTVKMDHIKQNYYGSFPSLNPLEIIPLGTNIDYSWPHDRNRFSSEIPLRRDCARRELEHASLVCELSL
ILLEIFGRLF
>Carubv10026474m
MTQLAPSDSSGSYTRPTSKFRLDPTQFPSVAASSELHLYVGLPCPWAHRTLIVRALKGLNDAVPVSIASPGQDGS
WEFKDNNIPIKDKDQLIPSLDKANRCRNLKEVYKSRSGGYEGRCTVPMLWDSRKKDVVCNESYDIIEFFNSGLNE
VARNADLDLSPPELKEKIQDWNQIVYPKVNNGVYRCGFAQSQEAYDGAVNELFSTLDEIEDHLGSNRYLCGERLT
LADVCLFTTLIRFDPVYNILFKCTKKKLVEYPNLYGYLRDIYQIPGVAATCDFPAIMDGYYKTLFPLNASGIQPA
ISSSGDQDSLLRPHCREFVSKAVEAQLTV*
>evm.TU.supercontig_135.12
MARSALDETSDSGAFIRTASTFRNFISRHPDSQFPAESGRYHLYVSYACPWASRCLAYLKLKGLDKSISFTVALQ
RMLTWICILSFAILVDETNEWVYNGINNGVYKCGFARKQEPYEEAMKQLYETLDKCEQILSKQQYICGNMLTEAD
IRLFVTLIRFDEVYAVHFKCNKKLLREYPNLFDHTKEIFQIPGMSSTVNMEHIKKHYYGSHPSINPFGVVPLGPD
IDYAAPHDRDRFAK
>Cre12.g538100
MAAATGKARTALDETSKTGEFKRTDAGFRNQIAPGTRFEPEAGRYHLYVSLACPWACRCLAVLHMKGLTDAIGVS
VTHPTWQRTRPEDPADEHTGWVFRAPGDAPLSSATGFGAFPCTGCVPDSINGATTVRQLYDMANDTTGKYSVPVL
WDKKEKTIVNNESSEIIRMFNTAFNDVAKNPGLDLYPEALRAAIDDLNAWTYPAINNGVYRCGFATSQAAYEAAF
EELFSALDRVEGILSRQRYLAGEVLTEADVRLFQTLIRFDEVYVVYFKTNKRFLREYPNIAGYVRELYQESGMKP
AVDMYHIKTHYFTSHPKLNYYAVVPRGGEAWWEQPHDRAAKFPAAGKL
>Cre03.g154950
MATDKPVAFRDFVTADGRFKPEAGRYHLYVCLGCPFACRCLAVLYMKGLEHVIGVSVTHPTFQRTRPDDHEDTHC
GWVFAKPTDPPLTATNGKGAFPCTGCVPDTVNGVRFVRDLYEMAGAKKVTFSVPVLWDKKEKTIVNNESPEIMRM
LNSAFNALAKHPEVDLYPEALQSTIDDTAAWVGPHINMAVYRCGFAPDQAAYDAGFKDLFAGLDRAESVLGGRRY

LTGDTLTEVDIKLFMTLIRFDEAYFVCYKTNKKLIRDYPNLCNYVRDLYHVPGIGRTVDLYHIKAAYFTCKPDMN
PNVIIPGGGDAWWAQPHDRADKFGGKREGGGLAGWLQKLPVWAPHAAASAAAALVAGAALGSSRRR
>Cre14.g611517
MQHRVVCALPPPSNPTGLAKGFSLLEWTNQVLPQGQLVSGVKEGWKMAWMAMMRELAPQSKDGAYTRPTYAFGGR
LGEAEFPAEAGRYHLYLGNACPWCHRVALAAVLRGWFRPQQQQQQQQLGPAAAAAAAVAGRQLGPAAAAAAAAVA
AAAGPQPLMSYTRLEDDPTRARRGGWVFGAADPDPVFGAADLWQVYDTVSPGFRGRCTAPLLVDRSKRAAVCNES
ADIVRGLDAANLPGATTAVQLRPPALAAQIDVLNARIYSAINNGVYRSGFATTQAAYDAVQAELWGALDEMEGRL
SASRFLLGDKLTECDVWLFPTIVRLDAMYGPLFKCSRRRIFGCSSPAAARPAAAGTAVARGHGAAAAAVAAAAVG
CGGDYPHLAGWARDIWQLQVPGSLVQISDTLDLDAARRSYFSSLFPLNPGGIVPAGPTAADLQLGSRAPGRGGSS
VLEEVCHLRRAAE
>IGS.gm_5_00114
MATAASRRQPPPPAAARTALDEMGKGGEFRRKDSAFRSWIKKGGEFEPEAGRYHLYVSLACPWASRCVALLHMKG
LEDVIGLSVTHPTWQRTRPDQDEHCGWAFASPDGPPLSSSTGHGSFTCEGCIPDPINGAQFVRDLYEKSSDTSGK
YSVPVLWDKKKGCIANNESSEIIRMLNSEFNDLAGNPQLDLYPADLAAAIDEANSWIYPAINNGVYRCGFATSQE
AYETAFGELFAALDRCEDILSRQRYIAGDRITEADIRLFHTLIRFDPVYVVVYFKTNGKFIRECPNLSNYTRDIFQ
TPGVSKSVNIRHIKVHYFSSHPKLNYYAIMPVGSESDPWWEKPHDRAERFGQKS
>estExt_fgenesh3_pg.C_30211
MAAAVCAVQPLHSGSLQARGRRPVVSSVVICQQQGGGAPKSGFRVLERTGALVPQGLLVKTAKYGWRTAWQTLMT
ELAPQSKDGTYQRPSYSFQGRVGSPEFPLEPGRYHLYVGNACPWCHRVLLALVVSGLEGHISFSRAVDDPERASR
GGWVFDGKDPVFGCRDLREVYDLLSPGFSGRCTAPLLVDRKARRAMCNESAFIARNFAELAMPPGAPPGAGSGAA
GGGGGAASGGGGGAVDLYPQALRGEIDRWNDKIYDTVNNGVYKCGFSTSQAGFTRAEAALFETLAELEAVLSRQR
FVCGDRFTEADLRLFPTIVRFDAVYATLFKCCRRRVADHPHLQAWLRDVQQLELPGSRMQIRDCFDLDDARRSYF
QQLFPLNPGGIVPSGPTAADLGLDALTNYPNAAYNNARCEPVSSNHRLLDTWVAAARRHGVPHHKVVPWVRRKLG
PAVVVLRFRGDGQLGCSVPCVLCQRELQRFDLSVHCYLGGDGAPGERQWFRGRLSDVGAPTPVLTAGQRRTLNLE
PSPSQPKKPRDSHPPALKNGGRKKKKQPAPS
>Ciclev10015774m.g
MIFLFSKTYFAKTSFILSLFPFKHSRQMARSAIDEVSETGSFTRTASTFRSFISRDPNSQFPAESGRYHLYISYA
CPWASRCLAYLKIKGLEKAISFTSVKPIWEQTKETDEHRGWVFPATDTEEPGAEPDPLNGAKTIRDLYELASTNY
SGKFTVPVLWDKKLKTIVNNESAEIIRMVNTEFNDIAENASLDLYPSDQRDQIDGTNEWIYNGINNGVYRCGFAT
KQGPYDEAVRQLYEALDKCEEILGKQRYICGNRLTEADIRLFVTLIRFDEVYAVHFKCNKKLLREYPNLFNYTKD
IYQIPSMSSTVNMQHIKRHYYGSHPSINPYGIIPLGPDIDYSSPHDREIFSA*
>Ciclev10020368m.g
MICTSSHLSSQLLIISPHKTKPTIHYKCHVSPRMSLDQQRPPSSSSSSTSPTRLLTAVTKLLWGPSLPPGLLVST
VRTSWNAAWQLMMSQLAPSDSSGSYTRPASKFFLINNPPSTANLHLYVGLPCPWAHRTLIVRALKGLEDAVPVSI
AGPGQDGSWEFTNNRNPSRDKDIPVPGLDNENGCKNLKEVYKLRKGGYSGRATVPMLWDVDNKDVACNESYDIIQ
FFNSGLNRSARNPDLDLAPVELKGKIEEWNKIIYPNVNNGVYRCGFAQSQEAYDTAVNDLFSKLDMIDDHLGGSR
YLCGDTLTLADVCLFTTLIRFDLVYNVLFKCTKKKLLEYPNLHGYMREIYQIPEVAATCNLTAIMDGYYKILFPL
NPGSIRPVMPSGCEHEVLLRPHNRESLPSVDRNTPVYIS*
>orange1.1g019639m.g
MIFLFSKTFFAKTSFILSLFPFKHSRQMARSAIDEVSETGSFTRTASTFRRFISRDPNSQFPAESGRYHLYISYA
CPWASRCLAYLKIKGLEKAISFTSVKPIWEQTKETDEHRGWVFPATNTEEPGAEPDPLNGAKTIRDLYELASTNY
SGKFTVPVLWDKKLKTIVNNESAEIIRMFNTEFNDIAENASLDLHPSDQRDQIDGTNEWIYNGINNGVYRCGFAT
KQGPYDEILGKQRYICGNRLTEADIRLFVTLIRFDEVYAVHFKCNKKLLREYPNLFNYTKDIYQIPSMSSTVNMQ
HIKRHYYGSHPSINPYGIIPLGPDIDYSSPHDREKFSA
>orange1.1g015033m.g
MICTSSHLSSQLLIISPHKTKPTIHYKCHVSPRMSLDQQRPPSSSSSSTSPTRLLTAVTKLLWGPSLPPGLLVST
VRTSWNAAWQLMMSQLAPSDSSGSYTRPASKFFLRNNPPSTANLHLYVGLPCPWAHRTLIVRALKGLEDAVPVSI
AGPGQDGSWEFTNNRNPSRDKDIPVPGLDNENGCKNLKEVYKLRKGGYSGRATVPMLWDVDNKDVACNESYDIIQ
FFNSGLNRSARNPDLDLAPVELKGKIEEWNKIIYPNVNNGVYRCGFAQSQEAYDTAVNDLFSKLDMIDDHLGGSR
YLCGDTLTLADVCLFTTLIRFDLVYNVLFKCTKKKLLEYPNLHGYMREIYQIPEVAATCNLTAIMDGYYKILFPL
NPGSIRPVMPSGCEHEVLLRPHNRESLPSVDRNTPVYIS
>e_gw1.15.129.1
MQARTALEETENDKGEFKRKDATFRKWIGKDKDFPAEAGRYHLYISWACPWANRCAATIYLKASARHLQLCMQGL
EDVVGLSVVHPTWQKTKPDDPNDEHTGWTFAAPDDPPFTSVTGFGSFPPHECIPDTVNGAKYVRDLYEISNDTSG
KDPESKYSVPVLWDKKTKTIVNNESAEIVRMFNSEFNDFAKNQDLDLYPEALQKEIDSINEWVYPTINNGVYRCG
FAIKQEAYNEAFKELFDSLDRCEEILGKQRYIAGNQLTEADLRLFQTLIRFDEVYVVVYFKTNKYFIREKPNLTNY
VRDLYQIPGIGKAVHMYHIKTHYLTSHPRLNYYAIVPAGQTGPDGSDKGWWTEPHDRDRFADKATA
>fgenesh1_pm.2_#_147
MGWQFAWETMMKELAPQTKDGSYARPTYNLKGVIGSPELPAESGRYHLYVGNACPWCHRVLLALIVHGLLPHVSY
TMAVDDPERASRGGWVFDQAEPVFNAKDLREVYDAASPGYRGRCTAPLLVDKRTRKLVCNESSDIVRMLNSVQLP
GCTPFDLYPAQLSREIDAINDIVHNKINNGVYKSGFATTQSAYARAQQELYGALDLVEQRLSEHRFLVGDRFTEA

DLRLYPTVVRYDGMYATLFKCCRKRISDYPNLSAWLRDVYQITVQDASQMQISTSFDLDEARRSYFTSLFPLNPG
GIVPVGPTIDDLALHRDAGRGSHSAESVFNFQAMPSAVAA
>Cucsa.003910
MARSALDEMSTTGAFVRTASTFRNLISRDGSSQFPSESGRYHLYISYACPWASRCLAYLKLKGLEKAISFTSVKP
IWERTKDSDDHMGWVFPSSDTEEPGAEPDPLNGVRSLRELYELASTNYSGKYTVPVLWDMKLKTIVNNESSEIIR
MLNTEFNDIAENPHLDLYPTHLQAQIDETNDWIYRGINNGVYKCGFARQQQPYDQAVKELYEALDRCEEILSKQR
YLCGNTLTEADIRLFVTIIRFDEVYAVHFKCNKKLLREYSNLFNYTKDIYQTKGVGSSVNMEHIKKHYYGSHPTI
NPFGIIPLGPNIDYSSPHDRDRFSS
>Cucsa.140740
MPTASASIFFPSPPPYPPPAILTKNPSRCHVIPRMSLNQSPSTGPKRSSSASSSSSSLLASITNLLWGPSLPPGL
LIATVRTAWETTWQLMMAQLAPSDTSGTYTRPISQFRATKISANNLHLYVGLPCPWAHRTLIVRALKGLENAVPV
SIAGPGSDGSWEFREKPKAENETLNPGLDKANGCRTLKEVYRMKRGGYNGRSTVPMLWDAEKKEVLCNESFDIIE
IFNSGLNELAENPELDLSPPLLKRKIEEWNSIIYPNVNNGVYRCGFAQSQKAYDKAVEDLFSTLDLLDDHLGHSR
YLCGENLTLADVCLFTTLIRFDLVYNVLFKCTKKKLLEYDNLHGYMRDIYQIPKVSATCNFTAIMDGYYKTLFPL
NPGSIRPTIPTSCYHEALSVPSERRSLPFLGRIEQVSA
>Eucgr.D01044
MAGPMNMIASRFKPKFLASALVPKSNRFSCCTRIPSKNGSQMARSALDEMSDSGAFLRTASTFRNFISRDPSSQF
PAESGRYHLYISYACPWASRCLAYLKIKGLDKAISFTSVKPRWERTKESDEHMGWVFPSSDTEEPGAEPDSLNGA
KSIRELYELASTNYSGKFTVPVLWDKKLKTIVSNESSEIIRMVNTEFNNIAENAALDLYPPHLQTLIDETNEWIY
DAINNGVYKCGFARKQGPYDEAMKKLFDALDKCEEILGKQRYMCGNTLSETDIRLFVTLIRFDEVYVVHFKCNKK
QLHQYPNLFNYTKDIFQVPGMSSTVNMEHIKRHYYGSHPSINPFGIIPLGPNIDYSSPHDRERFS
>Eucgr.E01874
MEIYRSIWSQDLGHRKSEGDNGTMSLSALPRSCLFPPATRSCKQRCSAFSPSPSKLSIRMARSSIQEVSESGAFV
RTPSAFHNFISGDPDSPFPAESGRYHLYVSFACPWACRCLAYLKIKGLNKIIGHTVVKSKWGRTKDGDTHIGWVF
PASNADEPGAEPDPIIGAGSVRELYETASPIYSGKYSVPVLWDKELKTIVNNESSEIIRMLNSEFNGLAENPNLD
LYPPHLQAQIEEANGWIYDCINNGVYKCGFAKQQAPYEEAVENLYKALDKCEQILSNQRFICGNLLTETDIRLFV
TLIRFDEVYAVLFKCNRKLIREYPNLFNYTKDIFQIPGMSSTVNMDHIKLNYYGSHPSMDPLGIIPIGPNIDYFS
SHDRGRFTLSKQ
>Eucgr.K01434
MSSSACSACCRPSSSSSPAISASTTPFPSTSKFKPVLRTKPHATIPKMSLDPRDFHSPAPQPPPPSSSSSLLAAV
AALLWGPSLPPGLLVSSVRSAWTAAWRLMMSQLAPSDPSGRYARPASRFRLAGPSGLPGPRRPGALHLYVGLPCP
WAHRALVVRALKGLEDAVPVSVASPGVDGSWEFRDPPGPERGDLAPGRDRVNGCRNLREVYRRRRGGYSGRCTVP
MLWDVDRSDVACNESYDIIRVLNSGLNGLARHPEVDLAPPELEAEIEEWNRVIYPGVNNGVYRCGFAQSQEGYDA
AVTELFRTLDMLDDHLSRSRYLCGDKLTLADVCLFTTLVRFDPVYNVLFKCTKKKLVEYPSLHGYMRDIYQVPKV
AATCDFDAIMDGYYRFLFPLNPGNIKPIMPSAFAGDALFEPHGRERLSSQSRGTLVSS
>Thhalv10027346m.g
MARSAVDETSDSGAFQRTASTFRNFVSRDSNSQFPAESGRYHLYISYACPWASRCLSYLKVKGLDDAISFSSVKP
IWGRTKESDEHMGWIFPDSDTEVPGADTDHLNGAKSVRELYEIASPNYTGKYTVLWDKKLKTVVNNESAEIIRMF
NTEFNHIAGNPELDLYPSHLQAKIDETNEWVYDGINNGVYRCGFAKKQGPYEEAVKQVYEALDRCEEILGKHRYI
CGNTLTEADIRFFVTLIRFDEVYAVHFKCNKKLLREYPNLFNYTKDIFQVPGMSSTVNMNHIKQHYYGSHPSINP
FGIIPHGPNIDYSSPHDRHRFSR*
>Thhalv10000959m.g
MARSGVDETSESGAFVRTASTFRNFVSQDPDSQFPAESGRYHLYISYACPWACRCLSYLKIKGLDEAISFSSVHT
IWGRTKETDDHRGWVFPDSDTELPGAEPDYLNGAKTVRDLYEIASPNYEGKYTVPILWDKKLKTVVNNESSEIIR
MFNTEFNSIAKNPSLDLYPSHLRDTIDETNEWVFNGINNGVYRCGFARKQEPYDEAVNQLYEAVDRCEAILGKQR
YICGNTFTEADIRLFVTLIRFDEVYAVHFKCNKRLLREYPNIFNYIKDIYQINGMSSTVKMDHIKQHYYGSHPTI
NPFGIIPQGPNIDYSSPHDRDRFSS*
>Thhalv10003220m.g
MAVYRAPPQLSFPSFTTTTTSFSPRHVSPIMSNQSPKSSTSTSIFTSATKLLWGPSLPPGLLISTARTAWTTVWQ
LMMTQLAPSDSSGSYTRPTSQFRLNPTQFSSAASSELHLYVGLPCPWAHRTLIVRALKGLNDAVSVSVASPGQDG
SWEFKDYNSIPIQDKDKVIPSLDKANRCRNLKEVYKSRTGGYDGRCTVPMLWDSRKKEVICNESYDIIVFFNSGL
NEFARNPELDLSPPELKEKIENWNRIVYPKVNNGVYRCGFAQSQEAYDQAVNEIFSTLDEIEENLSGSRYLCGEK
LTLADVCLFTTLFRFDPVYNILFKCTKKKLVEYPNLYGYLRDIYQIPGVAATCDLPAIMDGYYKTLFPLNASGIG
PAISSSGDQDSLLRPHNRDLVGKVVEAEYAV*
>gene20040-v1.0-hybrid
MARSALDEVSQSGAFVRSASEFRNFISRDPNSQFPAEAGRYHLYISYACPWASRCLAYLNIKGLQKAISFTSVKP
IWERTKESDEHMGWVFAASDKELAGAEPDPLNGAKSVRELYEIASTKYTGKYTVPVLWDKKLGTIVSNESSEIIR
MFNTEFNSIAENPALDLYPTHLQSQINQTNEWIYDKINNGVYKCGFAKKQEPYDEAVKQLYEALDRCEEILSKQR
YLCGNTLSEADIRLFVTLIRFDEVYVVHFKCNKKLLREYPNLFNYTKDIFQVPGMSSTVQMDHIKQHYYGSHPSI
NPFGIVPSGPNIDYSSPHDRYKFSA
>gene08323-v1.0-hybrid

MNCSSASLLSSSFLPAPPPNTNTSSSFKLPQSKPNRRHVTAKMSLNKPPDPTSLLTSVTNLLWGPSLPPGLLIST
VRSAWNTTWRLMMSQLAPSDPSLAYTRPPSRFRATQIPRQNRTSLHLYVGLPCPWAHRALIVRSLKGLESAVPVS
IASPGNDGSWEFKTTRDPDPDTLDPTRDKANGYKTLKQVYKSRPGGYDGRSTVPMLWDSNNKEVVCNESYDIIEL
FNSGLNEMAHNPGLDLSPPSLKPKIEEWNSIIYPNVNNGVYRCGFAQSQEAYDVAVNDLFNTLDKLEDHLSRFRY
LCGDELTLADVCLFTTLIRFDLAYNVLFKCTRRKLVEYPNLHAYMRDIYQIPKVAETCNFQLIMEGYYKTLFPLN
PGSIQPIIPVGSEHQELLRPHNRDSLCLKGARESASTLSVS
>Glyma20g02490
MLCLRLPLLRPKSTFPPSDFCYNYYQYKHIVGMARFSLDEISDTGAFVRSASTFRNWISRDPNSLFPPESGRYHL
YVSYACPWASRCLAYLNIKGLDKAISFSAVKPIFERTRESDEYKGWIFPESETEVPGAEPDRFNGAKTIRELYEI
ASENYAGKYTVPVLWDKKLKTIVNNESSEIIRMFNTEFNNIAENPTLDLYPTDLKAQIDDTNEWIYDSINNGVYK
CGFAKKQEPYNEAARQLYGALDKCEDILSKQRYICGNTLTEADIRLFVTLIRFDEVYAVHFKCNKKLLREYPNLF
NYTKDIFQIPGISSTVNMEHIKLHYYGSHPSINPFGIVPVGPNIDYSAPHDRERFSA
>Glyma10g04570
MASCTCSSSLLLVNPPPSTSRHHAIRRNLRMSLNNNDSKLNPSSLLNSVTKLLWGQSLPPGLLVATVRTAWNSTW
HLMMSQLAPSDSSGGYSRPASKFRFSSTTPSQGSSLHLYVGLACPWAHRTLIVRALKGLEDAVPVSVASPGLDGS
WEFKRAGGPDTNTIAPSLDRANGCNTLKEVYRLRRGGYDGRSTVPMLWDKGSKDVVCNESYDIIHLFNSELNSVA
QNPGLDLSPPQLKKQTEEWYQIIYPNVNNGVYRCGFAQSQEAYDRAVNDLFSTLDKLEDHLANSRYLCGDTLTLV
DICLFTTLIRFDLAYNVLFKCTKKKLCEYTNLHAYMRDIYQIPKVAATCNFTEIMDGYYKILFPLNPGSIRPAMP
STSEHESLCRPHGRESLSSATLAFVK*
>Gorai.009G151800
MRTASTFRNFISRDPSSQFPPESGRYHLYVSYACPWASRCLAYLKVKGLDKAIGFTSVKPIWERTKETDEHMGWV
FPTSDTEEPGAAPNPFNGAKSIRELYELASTNYTGKYTVPVLWDKKFKTIVSNESAEIIRMLNTEFNDIAENPAV
DLYPPHLRAEIDKTNEWIYSGINNGVYKCGFARKQEPYDEAVKQLYDALDQCEEILSKQRYLCGNILTEADIRLF
VTLIRFDEVYVVHFKCNKKLLREYPNLYNYTKDIYQIPGMSSTVNVQHIKRHYYGSHPSINPFGIIPVGPNSDYS
SPHDRARFTA
>Gorai.001G181000
MHCISLPSSHLLLHSPAKTTLKLACHVSPRMFFNDSATNNNSGSTNGFSLIKNVSKLLWGASLPPGILISTVRTA
WTSTWQIMMSQLAPSDPSGAYTRPFSKFRLNPTAATTKLHLYVGLPCPWAHRTLIVRALKGLEEAVPVSVAGHGL
DGSWEFKDVPDKDNDILVPTMDGVNRCRNLKEVYRLRKGGYDGRATVPMLWDVEKKEVVCNESYDIIEFFNSGLN
ELAQHPGLDLSPVELKEEIEEWNGVIYPNVNNGVYRCGFAQSQEAYDVAVSGLFSTLDRIDDHLGSSRYLCGDRL
TLADICLFTTLIRFDLVYNVLFKCTKKKLLEYTNLHAYMRDIYQIPKVATTCNFLEIMDGYYRMLFPLNPGSIRP
VMPSVCEHEFLSRPSKRESMSSVGKRVQHVL
>Lus10038348.g
MARSAIDELSQSGAFMRSASTFRNFISRDSSSQFPPESGRYHLYVSYACPWASRCLAYLKIKGLDSHISFTSVKP
IWGRTKDSDEHMGWVFPSSDTEEPGADPDFLNGAKTVRDLYELASTNYAGKYTVPVLWDKKLKTIVNNESAEIIR
MFNTEFNDIAENPALDLYPAELRSQIDAANEWIYSGINNGVYKCGFARKQAPYEEAIKELYECLDKCEEILSKQR
YMCGDTLTETDIRLFVTLIRFDEVYAVHFKCNKKLLREFPNVLNYTKEIYQIPGMSSTVNMAHIKRHYYGSHPSI
NPFGIIPLGPDVDYSSPHDRDRF
>Lus10036209.g
MARSAIDELSQSGAFMRSASTFRNFISRDSSSQFPPESGRYHLYVSYACPWASRCLAYLKIKGLDSHISFTSVKP
IWGRTKDSDEHMGWVFPSSDTEEPGADPDFLNGAKTVRDLYELASTNYAGKYTVPVLWDKKLKTIVNNESAEIIR
MFNTEFNDIAENPALDLYPAELRSQIDAANEWIYSGINNGVYKCGFARKQAPYEEAIKELYECLDKCEEILSKQR
YMCGDTLTETDIRLFVTLIRFDEVYAVHFKCNKKLLREYPNVLNYTKEIYQIPGMSSTVNMAHIKRHYYGSHPSI
NPFGIIPLGPDFDYSSPHDRDRF*
>Lus10014304.g
MASSTQNELSNSGTATTFRNFITQDPNSQFPPESGRYHLYVSYACPWASRCLAYLSITGLINHISFTSVKPLWGR
TKDSVDEHMGWVFPNSETDEPGSEPDPLNGAKSIRDLYDLASTNYTGKYTVPVLWDKKLSTIVNNESAEIIRMLN
AGFNDIVTLDLYPPHLRDRIDETNSWIDSGINSGVYKCGFARKQSQYEEAIRDLYESLDNCEAILNKQRYICGDK
LTEADIRLFVTLIRFDEVYAVNFKCSKKLLREYPNLFNYTKDVYQIPGMSSTVNMAHIKLFYYGSHPSMNPSGII
PVGPDVDYAAPHDRARF*
>Lus10027233.g
MYLTGGANLLTSYRSPVTSSSGGARISLNRTSQPLNPTASLLTSVTKLLWGPSLPPGLLISTVRTGWSTAWNLMM
SQLAPSDSSGNYTRPPSKFRLSPSPDPRFSGNLQLYVGLPCPWAHRTLIVRALKGLQDAIPVSIAAPGQDGSWEF
SSGENSVLGDVLVPGKDSSNGCRTLKEVYNRRRGGYSGRATVPMLWDSARKEVVCNESYDIIEFFNSGLNDLAGN
PELDLSPESLKGEIDEWNRLIYPNINNGVYRCGFAQSQEAYDRSVNDLFEVLEKVEEHLQASRYLCGERLTLADV
CLFTTLIRFDPVYNVLFKCTKKKLVEYPNLHGYMRDIYQMPKVAVTCNFGAIMDGYYRTLFPLNPGGICPAVPLG
SEIQNLSKPHNRESLSSSDSKSRGVLC*
>MDP0000146135
MAQTTEISKSGAFLRTASVFHNFISRDPNSQFPAEPGRYHLYISYGCPWASRCLAYLKIKGLEKAISFTSVKPTW
GRTKESDEHMGWLFPASDAEVAGAEPDPLNGAKTIRELYELASTQYSGKYTVPVLWDKKLKTIVNNESAEIIRMF
NTEFNDMAENASMDLYPPHLQSQIDQTNEWIYDKINNGVYKCGFARNQEPYEEAVKELFEALDKCEEILSKQRYL

CGNTLSEADIRLFVTLIRFDEAYAVNFKCNKKLLREYPNLFNYTKDIFQVPGVSSSVNMEHIRRGYYGLLAVNPS
GIVPIGPNIDYSSPHDRDRFST
>MDP0000269900
MAQTTEISQSGAFLRTASVFRNFISRDPNSQFLAEPGRYHLYISYGCPWASRCLAYLKIKGLEKAISFTSVRPTW
GRTKETDEHMGWVFPASDTEVVGAEPDPLNGAKSIRELYELASSQYTGKYTVPVLWDKKLKTIVNNESAEIIRMF
NTEFNDTAENASLDLYPSHLQSQIDQTNEWIYDKINNGVYKCGFARKQEAYDEAVKELFEALDKCEEILSKQRYL
CGNTLSEADIRLFVTLIRFDEAYAVNFKCNKKLLREYPNLFNYTKDIFQVPGVSSSVNMDHIKRGYYTMAAINPS
GIIPIGSNIDYSSPHDRDRFST
>MDP0000261468
MAQPMEISQSGAFVRAPSVFRNFISXDPNSQFPAEPGRYHLYISYLCPWACRCLAYLKLKGLEKAISFTSVKPKW
ERTKESDEHMGWVFPASDTEVAGAEPDPLNGAKTIRELYDLASTHYTGKYTVPVLWDKKLKTIVNNESSEIIRMF
NTEFNDIAENASLDLYPPHLQSQIDQTNEWIYNMINNGVYKCGFARKQEPYDEAVKELFEALDKCEEILSKQRYL
CGNTMSEADIRLFVTLIRFDEAYAVNFKCNKKLLREYPNLFNYTKDIXQVPGVSSTVNIDHIKRGYYNIAAINPF
GIIPVGPHIDYSSPHDRDRFST
>MDP0000240930
MAQTTEISQSGAFLRTASVFRNFISRDPNSQFLAEPGRYHLYISYGCPWASRCLAYLKIKGLEKAISFTSVRPTW
GRTKETDEHMGWVFPASDTEVVGAEPDPLNGAKSIRELYELASSQYTGKYTVPVLWDKKLKTIVNNESAEIIRMF
NTEFNDTAENASLDLYPSHLQSQIDQTNEWIYDKINNGVYKCGFARKQEAYDEVYFASCNYECAGSDVYILFKDY
LCHLKFIGLSFSSQAVKELFEALDKCEEILSKQRYLCGNTLSEADIRLFVTLIRFDEAYAVNFKCNKKLLREYPN
LFNYTKDIFQVPGVSSSVNMDHIKRGYYTMAAINPSGIIPIGSNIDYSSPHDRDRFST
>MDP0000866259
MAQTTEISQSGAFLRTASVFRNFHFTGPEFTVIPAEPGRYHLYISYGCPWASRCLAYLKIKGLEKAISFTSVKPM
WGRTKESDEHMGLLFPASDAEVAGAEPDPLNGAKSIRELYELASTQYSGKYTVPVLWDQKLKTIVNNESAEIIRM
FNTEFNDMAENASMDLYPPHLQSQIDQTNEWIYDMINNGVYKCGFARKQEPYDEAVKELFEALDKCEEILSKQRY
LCGNTLSEADIRLFVTLIRFDEAYAVNFKCNKKLLREYPNLFNYTKDIFQVPGVSSSVNMEHIRRGYYGLLAVNP
SGIVPIGPNIDYSSPHDRDRFST
>cassava4.1_008745m.g
MYCTSSLCSHFPIFSTTSSATAAKAKGRCVVYPRMSMNQTPQSPIPKKLLTTITNLLWGPSLPPGLLISTVRTAW
NSAWQIMMSQLAPSDSSGGYTRPASKFRLSNSQYSRRNPTTLHLYVGLPCPWAHRTLIVRALKGLEDAIPVSIAA
PGQDGSWEFQNTPNTDKYILVPSKDEANGRKTLKEVYGITRGGYSGRATVPMLWDSERKEVVCNESYDIIEFFNS
GLNGLAQNPDLDLSPNSLKGKIEEWNQLIYPNVNNGVYRCGFAQSQEAYDRAVDELFTTLDKIDNHLGTSRFLCG
DTITLADVCLFTTLIRFDPVYNVLFKCTKKKVVEYPNLHGYMRDIYQMPKVAETCNFSAIMDGYYKILFPLNPGG
IRPTMPSGCEGEILYAAHNRESVSSVNKNTQVYVS
>Medtr4g084040
MFCLRSVVLHKSSFTLSLFSHNRHYKHIVEMVRSSLDEISDSGAFTRSASTFRQFVSKDPNSQFPPESGRYHLYI
SYACPWACRCLAYLKIKGLDKAISFSSVKPIWGRTKESDEYMGWIFPESNTEVLGAEPDPLNGAKSVRELYEIAS
TNYSGKFTVPILWDKKLKTIVNNESSEIIRMFNTEFNDIAENPILDLYPSELQAQIDETNEWIYPNINNGVYRCG
FAKKQEPYIDAARQLYEALDKCEDILSKQRYICGNKLTEADIRLFVTLIRFDEVYAVHFKCNKKLIREYPNIFNY
TKDIFQIPGISSTVNMEHIKLHYYGSHPSINPFGIVPMGPNIDYFAPHDRERFST
>MicpuC2.estExt_fgenesh1_pm.C_60070
MQVHLTGFLEHNAAAGRYHLYVSYACPWASRCLSVLGLKGIGRDVVSVSSVHPTWAKTRPDDVDDAHCGWRFWRE
DDDDDATVSNPAGVGAFPGGAKYNCTADDLHGVTYVRDLYDMVNAPKGTRFTVPILWDKKTNSIVSNESADIIRD
LYTQFDAFAKNPDLDLYPEALRDEIDAVNEWVYHGINNGGAYEEAVNELFRCLDRAEEILSKRRYIAGDVLTEAD
VRLFPTLVRFDEVYVVVYFKCNRKFIHQYPNLHNYVLDVYQTPGMRESVDMWHIKTHYFTSHPGLNQHAVVPVGPG
VDLDAPHDRARFEKKA
>MicpuC2.fgenesh1_pm.C_scaffold_5000099
MMTELAPSDDAGAYVRPSYGFDDTLSNDPTAKHPAMCGRYVVYVGNACPWCHRVTLTIALKGLSDAIAVVKMTDD
AERASRGGWVFEQFPASARDPVFGARDLREVYDAAVMSKGPAGAPYRGRCTAPLLLDAARKTPVCNESADIVRAL
NDVDFVGVGQGVGQGVGQSDAEEGVVELRPAALLDEIDAWGEEVYRGLNNGVYRCGFATSQAAYERAATDVKTTL
LKVEKRLSESRFLCGDKITEADVRLFPTIVRFDAVYATLFKCSNVRVADLPCVSGFMKDVYALPGVRETVDLAGY
RSSYFGQLFPLNPGGIVPIGPTEADLGLGEDANRGTGDVFHRR
>e_gw2.07.270.1
MTPGETAVADVSKDGAFVRKDAIFRNRITPDGPYTPESGRYHLYVSYACPWACRTLSLLHLKGLTQHVTVSSVHP
TWQRTRPDDPDDKHAGWCFVGTDGASVSGPSGVGSFPGGPSYNCTPDHLFESTFVRDVYDRAMGGAAVGTRFTVP
ILWDKQTNTIVSNESSEIIRDLNSQFNAFADNKELDLYPEALRPAIDEVNEWVYHGINNGVYKCGFATSQGAYDA
AVTELFRCLDRCEDILGKRRYIAGDVLTEADVRLFPTLVRFDEVYVVVYFKTNKKFIHQYPNLWNYVKDVYQTPGM
RHSVNMWHIKTHYFTSHPVLNANAIVPIGPDLNLDEPHDRHRFGAAK
>e_gw2.08.300.1
MVTRAASDTSGLGLLTWLGPVIPQGVLVTGVKAGWRAAWQTMMTELAPQSRDGEYQRPKYAFDGAIAEDPKARFP
SVSERYVLYLGNACPWCHRVSLTVALRGLEDRVRIVKMTDDAERASRGGWVFESDRPDPVFGARDLREVYDLQSG
KSYTYEGRCTAPLMVDAERKVAVNNESADIARMLNDVEWLGSVNGPNKSSRYDEGSVQLRPPELANDIDSLNDWL

YTKLNNGVYRCGFSTNQAAHNRAAVDVAEALEALEKRLRESRFMHGDKVTESDVRAFPTIVRFDAVYATLFKCSS
RRVADMPNLRAWMQDFYLLPGVRETVDVDGYRTSYFGQLFPLNPGGIVPVGPTAKDLGLGLDPSRGSRAHSDLFH
FK
>mgv1a010010m.g
MARSALDEMSATGAFIRTASTFRNSISRDPNSPFPAESGRYHLYVSYACPWASRCLAYLKIKGLSKAITFTSVKP
IWGRTKDTDDHMGWVFPVSDTEERGAEPDYLNGVKNIRELYDLASANYSGKYTVPVLWDKKMKTIVNNESEEIIR
MFNTEFNDIAEHAEVDLYPLHLQSQINEVNDWVYDGINNGVYKCGFAKKQAPYDEAVTKLYEALDKCEGILSKQR
YLCGETLTEADIRLFVTLIRFDEVYAVHFKCNKKLLREYPNIFNYTKEIFQIPGVGSTVNMEHIKKHYYGSHPSI
NPFGIIPHGPNLDYSSPHDRNRFSK*
>mgv1a009815m.g
MMSQLAPSDPTGRYTRSASQFRLPLTNISRQNLHLYVGLPCPWAHRTLIVRALKGLEQSIPVSIAAPGADGSWEF
TNPSLQNKDSSTTTLLPTWDKANRCKTLKEVYNSRSGGYNGRSTVPMLWDADNKQVICNESYDIIQFFNSGLNNL
STNPTLDLSPPQLKAKIDKWNRIIYPNINNGVYRCGFAQSQEAYDSAVNELFSTLDMVDEHLERSRYLCGDELTL
ADVCLFTTLIRFDPVYNVLFKCTKKKLIEYPNLHGYMRDIYQIPKVAATCNLGAIMDGYYKTLFPLNPGGIGPAI
PYACEHEMLSKSHNREGLSVVEQNDVNALIV*
>LOC_Os02g21460
MLTRLPHHSSPLVFPCRLSAAAAARTLSTATGSNSTTVKMARSALDEVTDAGAFDRSPSTFRSSISRDSSARFPA
VPGRYHLYVSYACPWASRCLAYLKLKGLDHAIGFTSVKPIFERTRETDDHLGWVFPATGDEEPGADPDPFNGAKT
IRELYEIASPNYIGKPTVPVLWDKQLKTVVNNESSEIIRMLNTEFNEIAKNPDLDLYPAHLQTSVNEINELVYDA
INNGVYKCGFAKKQGPYDEAVTRLYEALDKCEEILSRQRYICGNQLTEADVRLFVTLIRFDEVYAVHFKCNKRLL
REYPNLFNYTKDIYQIPGISSTVNMEHIRKHYYGSHPSINPYGIIPAGPNIDYNAPHDRERFSA
>LOC_Os02g56970
MPMWSQPPPPPSSLQLRRPPPPLPHRPRRLRSRLSPIAASQDPLTALSRLLWGRALPPSQLVLAVRHGWTAAWGL
LMRQLAPSDPATGAFTRTPSRFPAVVGTPSARLHLYVGLPCPWAHRALLVRALLGLERRLPLSVAVPGDDGAWSF
TPDSPDALYGKRKLREVYASARRGGFEGRASVPLLWDAERREVVCNESIEITKFLCDLAAADGSAGGLDLWPPEL
RQDIDRWYSFIYPSVNNGVYRCGFAQSQEAYDAAAGELFAALDRLEDHLSGSRYLCGDTLTLADVCLFTTLVRFD
LVYHSLFRCTRRKLVEYASLHAYTRDIYQMPGVAGTCDMAAIADGYFGALFPLNPGGILPLVPASCSPEALLEPH
GREALSSSAAADAGGGGNGRQLEATSASN*
>fgenesh1_pg.C_Chr_6000045
MDRTAVADVSPTGAFARKASTFRNAFDANGAHPPEAHRYVLVVSLACPWATRCLAALRAKGLEDVLDVAVTHPVW
GKTRPNDPNDAHAGWRFVKPGTVVTGPSGRGAFEADARCSATACEGAEFVRDLYEICGAPTEQRFTVPLIWDTLR
KTIVNNESSEILRELNTKFQDFCSSDAAREVDLYPEHLRPKIDEINQWVYDDINNGVYKCGFAVSQSAYDEAVKN
LFSALDRCEELLARSRYIAGDVFTEADVRLFMTLIRFDEVYVVYFKTNKKFIHQYEHMGAYVRELYQMPALRRAT
DFAHIKQHYFASHPSLNPHAIVPCGPNVDLDAAHGRDGPYPR
>Pavirv00008786m.g
MWATPPQPFPLQLRRPPGPPPRLFLTRHRSSRLNRIAASQDPLAALSNLLWGRALPPAQLVLAVRHGWTAAWQLL
MRQLAPSDPETGAFTRTPSRFPAVVGTPSSRLHLYVGLPCPWAHRALVVRALLGLEARLPVSVAVPGDDGAWSFT
PDSPDGLYGKRKLREVYAVRSGGFEGRASVPMLWDAERREVVCNESIEIIKFLCGLANADGGGLDLWPPELRQDI
DRWYGVIYPSVNNGVYRCGFAQSQEAYDAAASELFGALDRLEAHLAGARYLCGDRLTLADVCLFTTLIRFDLVYN
TLFRCTRRKLAEYPSLHAYTRDIYQMPKVAETCDMEAIMAGYFKTLFPLNPGGIQPLPPASCDSESLLRPHGREA
LSSAAGTPLQAAGVS
>Pavirv00025247m.g
MWATPPQPLPLQLRRPPGLPPRLFLTRHRSSRLNRIAASQDPLTALSRLLWGRALPPAQLVLAVRHGWTAAWQLL
MRQLAPSDPETGAFTRTPSGFPTVVGTPSSRLHLYVGLPCPWAHRALVVRALLGLEARLPVSVAVPGDDGAWSFT
PDNPDGLYGKRKLREVYAVRSGGFEGRASVPMLWDAERREVVCNESIEISKFLCGLANADGGGLDLWPPELRQDI
DRWYGVIYPSVNNGVYRCGFAQNQEAYDAAASELFGALDRLEAHLGGCRYLCGDRLTLADVCLFTTLIRFDLVYN
TLFRCTRRKLGEYPSLHAYTRDIYQMPKVAETCDMEAIMAGYFKTLFPLNPGGIQPLPPASCDSESLLRPHGREA
LSSAAGTPLQAAGVS
>Pavirv00037139m.g/EST FL726592.1
MARSAHDEVTDSGAFDPSPSTFRNFVSRDSTARFPAAPGRYHLYISYSCPWACRCLAYLKLKGLDHAIGFTSVKP
IFERTKETDDHMGWVFPATGDEEPGAEPDPFNGAKSIRELYEIASGNYAGKPSVPVLWDKQLKTIVNNESSEIIR
MLNTEFNEFAENPDLDLYPAHLQACIDGINELVYEAINIGVYKCGFAKQQGPYDEAVTKLFEALDKCEDILSQQR
FLCGNQLTEADVRLFTTLIRFDEVYSVYFKCNKKLIREYPNLFNYTKDIYQIPGISSTVNMEHIRKSYYGGYSPI
NPYGIIPAGPNIDYNALHDREKFSA
>Phvul.002G098300
MARSSMDEISNSGAFVRSASTFRNWISRDPNSQFPPESGRYHLYVSYACPWASRCLAYLNIKGLNKAISFSAVKP
IFERTKESDEYKGWVFPDSETEVPGAEPDRLNGAKSIRELYEIASANYTGKYTVPVLWDKKLKTIVNNESSEIIR
MLNTEFNNIAENPSFDLYPADLKALIDETNEWVYDSINNGVYKCGFAKKQEPHNEAARQLFGALDKCENILSKQR
YICGNTLTEADIRLFVTLIRFDEVYAVHFKCNKKLLHEYPNLFNYTKDIFQIPGISSTVNMEHIKLHYYGSHPSI
NPFGIVPVGPNIDFSAPHDRERFSA
>Phvul.007G189800

MSSCTSCCSASSLLLVNLPRGPSRHDIRGSLRMSFNNDNTNSKFTSSSLLNSVTKLLWGQSLPPGILVATVRTAW
NSTWGLMMSQLAPSDSSGGYSRPASKFRFLGPVSPGSLHLYVGLACPWAHRTLIVRALKGLEEAVAVSVASPGMD
GSWEFKRVGGADSGSIGPSLDKANGCKTLKEVYGLRRGGYDGRSTVPMLWDKGSKDVVCNESYDIIQLLNSGLNS
VAGNPEMDLSPPQLKEQIEEWYRIIYPNVNNGVYRCGFAQSQEAYDRSVNELFCTLDELEGHLANSRYLCGDQLT
LVDICLFTTLIRFDLAYNVLFKCTKKKLCEYTNLHAYMRDIYQIPKVAATCNFTEIMDGYYKVLFPLNPGSIRPV
MPSTSEHQILCRPHGRESLSSATPVFVK*
>Pp1s126_26V6
MMLRSCATQLMNPGQQIVSIWSATPRCAAPWNVSVALLSTRHAPWRPSAGLHHLSAYRVPFRSSTGSEVLKNTRR
PYFLKPFSFAMSRTSLHETHDGEFKRKESEFRHFISREPGAEFPPEADRYHLYISYACPWASRCLAFLKLKGLDH
AIGVTVTKPKWGETKPSVDDHHGWMFLEDGEDVPGAMKDSLNNAKSIRDLYEIASTGYTGKYTVPVLWDIKTMRI
VNNESSEITKMFNDEFNDIAKHPEVDLFPEHLKASIDSVNSWTYNAINNGVYRCGFATKQKPYEEAFNQLFEALD
RCEEILSKQRYIAGNELTEADIRLFMTLIRFDEVYVVHFKTNKKFIREYPNLFNYTKDLFQVPGIGATVNMYHIK
HHYYGSHPSINPFGIVPVGQKIDYSAPHDRDRFSKESA
>Pp1s348_23V6
MGDGSNSSMWSSPADFVKFLGQNLWGRNLPPGALVSVVKEVWTTGWLTMMAQLAPPTDKKDYSRPTSQFRGRLPS
PSSAKPGQYHLYVALACPWAHRTAIVHSLKRLGDAVPISVAVPGSTGLWEFAPKVGVNSKSGVVAERLRPTLDRA
NGQKLVMDVYKSQQGGYNGRSTVPMLWDSLQKRVMNNESSDIIEILNSDFNHLAENPDLDLAPADLKSQIEEWNE
FIYPNINNGVYRCGFAQSQEAYDSAVESLFNALDKLEAHLAGSRYLCQDTFTLADVRLFTTLFRFDAVYQILFKC
SKQKLKEYPNLEGYMRDIYQIPGVASTCDMDAIMHGYYKVLFPLNPGGISPAMPRVADSASLLEPHNRQKVADLV
VGQ
>Potri.015G121600
MICVFTKPHLLALGPNKNNISFILPQLSLKCNLQMARSAIDETSDTGAFKRTASTFRNFISKEPNSQFPPESGRY
HLYVSYACPWASRCLAYLKIKGLEKAIAFTSVKPIWERTKESDEHMGWVFPASETEEAGAEPDPLNGARSIRELY
ELASTNYAGKYTVPVLWDKKLKTIVNNESSEIIRMFNTEFNDIAENAALDLYPSHLQARIDETNEWVYNGINNGV
YKCGFARKQGPYEEAAIQLYEALDKCEEILGRQRYICGNTLSEADIKLFVTLIRFDEVYAVHFKCNKKLLRDYPN
MFNYTKDIFQIPGMSSTVNMQHIKRHYYGSHPTVNPFGIIPLGPDIDYSSPHDRNRFSS
>Potri.014G192300
MTILSRCIVIMSGVLLFSSQFPIFSTTTTATQKTHRFACHVIPRQSLNQTTPPDQQSPNPKTILSTITNLLWGQS
LPPGLLISTVRTTWNSTWQLMMSQLAPSDSSGRYTRPASKFRLNPPFTLQNSTTLHLYVGLPCPWAHRTLIVRAL
KGLEDAVPVSIAGPGQDGSWEFKDIPISNRDRNILVPGRDNANGCRNLKGVYGLRRSGGYSGRATVPMLWDVEKK
EVGCNESYDIIEFFNSGLNGLARNPGLDLSPKELKGKIGEWNGLIYPNVNNGVYRCGFAQSQDAYDSAVNGLFTT
LEAVEDHLTTSRYLCGDTLTLADVCLFTTLIRFDIVYNVLFKCTKKKLIEYPNLHGYMRDIYQMPKVAETCNFSA
IMDGYYKVLFPLNPGSICPVMPSGCEHDVLLSTPHNRESLSLANKTTKQDH
>ppa008612m.g
MARSALDETSLSGAFVRTASVFRNFISRDPNSQFPAEPGRYHLYISYACPWASRCLAYLKIKGLEKAISFTSVKP
IWERTKESDEHMGWVFPASDTELAGAEPDPLNGAKSIRELYELASTNYTGKYTVPVLWDKRLKTIVSNESAEIIR
MFNTEFNDIAENAALDLYPPHLQSQIDQTNEWIYNKINNGVYKCGFARKQEPYDEAVKQLYEALDKCEEILSKQR
YLCGNTLSEADIRLFVTIIRFDEVYAVHFKCNKKLIREYPNLFNYTKEIFQVPGMSSTVNIDHIKRHYYGSHPSI
NPFGIIPSGPAIDFSSPHDRDRFSI
>ppa006395m.g
MSCTSLLSPPNFPTPPPLKKLSTAKWARPKPITCHVSTKMSSNQPPDPTSLLSSAAKLLWGPSLPPGLLISTVRT
AWNSTWRIMMSQLAPSDPTLAYTRPPSKFRATPNQIPRQAQTSLHLYVGLPCPWAHRTLIVRALKGLNDAVPVSI
AAPGQDGSWQFSSTPIPDPNTLVPGPDNANGFKTLKEVYKSRAGGYDGRSTVPMLWDVKKREVVCNESYDIIQLF
NSGLNELARNPGLDLSPPPLKQKIETWNSIIYPNVNNGVYRCGFAQSQQAYDTAVNELFDALDMVEDHLGSSRFL
CGDDLTLADVCLFTTLIRFDLVYNVLFKCTKKKLIEYRNLHAYMRDIYQIPEVAETCNFTSIMEGYYKTLFPLNP
GGIQPVMPSGSEHEVLCRPHNRDSPSVKEKSAAALYIS
>30060.t000030
MFASVFSKTHLAFDPKKRKSSIIFILSSFIFKHHLQMARSALDEMSDTGAFLRTASTFRNFISRDPNSQFPAESG
RYHLYVSYACPWASRCLAYLKIKGLDKAISFTSVKPIWERTKDSDEHMGWVFPASETEEPGAEPDPLNGAKSIRE
LYELASANYVGKYTVPVLWDKKLKTIVSNESSEIIRMFNTEFNDVAENAALDLYPSHLQVQIEETNEWVYSGINN
GVYRCGFAKKQGPYEEAAKQLYDALDKCEKILGKQRYICGNTLSEADIRLFVTLIRFDEVYAVHFKCNKKLLREY
>29637.t000026
MYCTSLPSSHYPTPTVPTKSNRKCHTFPKMSLDQTPPPNPKTLITTLTNLLWGQSLPPGLLISTVRTTWNSAWQL
MMSQLAPSDSSGSYSRPTSKFRISRNPNFQSFHLYVGLPCPWAHRTLIVRALKGLEHAIPVSIAAPGQDGSWVFD
SGQNMDKDTLVPGKDSANGCKSLKEVYGLRQGGYNGRATVPMLWDSERKEVLCNESYDIIEFFNSGLNGLARNPD
LDLAPKFLKGKIEEWNQVIYPNVNNGVYRCGFAQSQAAYDRAVNDLFSTLDRVENHLGSSRYLCGDTITLADVCL
FTTLIRFDLAYNVLFKCTKKKLLEYPNLHGYMCDIYQFINLLFVPFKLKSLLLLQLCYTYSVSVCICFPKQIPKV
AETCNFSAIMDGYYKVLFPLNPGSIRPVIPSGCEHEFLSAPHNRESLLSSVDKTMQVFVS
>230204
MGKTAIDETSTDGEFKRTESSFRSWISSKEGCDIPAVADRYHLYISYACPWASRCFATLKLKGLDHAIGVTSTKP
KWERTKEGESHFGWTFPSSDNEEPGAQPDPINNAKTVRELYDIANPNYTGKYTVPVLWDKAKKTIVNNESSDIIR

MLNSKFNHLAKRPDFDLYPRDLQAKIDEVNAWVYDSINNGVYRCGFATKQKPYEEAFEALFNALDRCEDILSKQR
YLCGNVFTEADLRLFKTLIRFDEVYVVHFKCNKKLIREYPNLFNFVKDVYQMDGISDTVRMDHIKKHYYGSHPSI
NPYGVIPVGSVIDYSTPHDRHKFNT
>266696
MGKTAIDETSTDGEFKRTESSFRSWISSKEGCDIPAVADRYHLYISYACPWASRCFATLKLKGLDHAIGVTSTKP
KWERTKEGESHFGWTFPSSDNEEPGAQPDPINDAKTVRELYDIANPNYTGKYTVPVLWDKAKKTIVNNESSDIIR
MLNSEFNHLAKRPDFDLYPRDLQAKIDEVNAWVYDSINNGVYRCGFATKQKPYEEAFEALFNALDRCEDILSKQR
YLCGNVFTEADLRLFKTLIRFDEVYVVHFKCNKKLIREYPNLFNFVKDVYQMDRISDTVRMDHIKKHYYGSHPSI
NPYGVIPVGSVIDYSTPHDRHKFNT
>79804
MGKTAIDETSTDGEFKRTESSFRSWISSKEGCDIPAVADRYHLYISYACPWASRCFATLKLKGLDHAIGVTSTKP
KWERTKEGESHFGWTFPSSDNEEPGAQPDPINDAKTVRELYDIANPNYTGKYTVPVLWDKAKKTIVNNESSDIIR
MLNSEFNHLAKRPDFDLYPRDLQAKIDEVNAWVYDSINNGVYRCGFATKQKPYEEAFEALFNALDRCEDILSKQR
YLCGNVFTEADLRLFKTLIRFDEVSSGVFFFLAFSRSFIQIHYLCQVYVVHFKCNKKLIREYPNLFNFVKDVYQM
DGISDTVRMDHIKKHYYGSHPSINPYGVIPVGSVIDYSTPHDRHKFNT
>EST DN839210.1/80092
MGKTAIDETSTDGEFKRTESSFRSWISSKEGCDIIPAMADRYHLYISYACPWASRCFATLKLKGLDHAIGVTSTK
PKWERTKEGESHFGWTFPSSDNEEPGAQPDTINNAKTVRELYDIANPNYTGKYTVPVLWDKAKKTIVNNESSDII
RMLNSEFNHLAKRPDFDLYPRDLQAKIDEVNAWVYDSINNGVYRCGFATKQKPYEEAFEALFNALDRCEDILSKQ
RYLCGNVFTEADLRLFKTLIRFDEVYVVHFKCNKKLIREYPNLFNFVKDVYQMDGISDTVRMDHIKKHYYGSHPS
INPYGVIPVGSVIDYSTPHDRHKFNT
>231328
MMQQLAPRDSGDGTYRRPESQFRGRFEEPVSVEHAGRYHLYASPVCPWAHRALIVHSLKGLGSAVPISLALPGKS
GLWEFEAGRYPDKAHNCSRLIDVYRMNSGGFQGRATVPMLWDRVRKAVVNNESADIIAMLNSDFNALAENQHLDL
APASLGQEMDRWNQLVYANINNGVYRCGFAQSQNAYDHAVHNLFSALETVEKHLQCSRFLCGDSLTLADIRLFTT
LYRFDAVYHILFKCSKRKLSEFSNLYGYMRQIYQIPGVSSTCNLPSIMDGYYGALFPLNPGGIQPAIPSSMEPKV
LYNQ
>Si022398m.g
MLHPSIDAETAECDSAEGRRQSTRFPSRRTSTATAARTLSTTFVDVKMARSGHEEVTDSGAFDPSPSTFRSFVSR
DSSARFPAAPGRYHLYVAYSCPWACRCLAFLKLKGLDHAIGFTSVKPVFERTKETDDHMGWVFPATKDEEPGAEP
DPFNGAKSIRELYEIASRNYAGKPSVPVLWDKQLKTIVNNESAEIIRMLNSEFNEFAKNPDLDLYPAHLQASIDE
INELVYEAINIGVYKCGFAKQQGPYDEAVTKLYEALDKCEDILSKQRFLCGNQLTEADVRLFTTLIRFDEVYSVY
FKCNKKLIREYPNLFNYTKDIYQIPGISSTVNMEYIKKSYYGGYSPINPYGIIPVGPNIDYNAPHDREKFNA
>Si017438m
MWATPSPPLPLRLRRPPGPPPRPGPFLTRHRRRLNRISASQDPLSTLSRLLWGRALPPAQLVLAVRHGWTAAWQV
LMRQLAPSDPATGAFTRTPSRFPAVVGTPSSRLHLYVGLPCPWAHRTLVVRALLGLEARLPVSVAVPGDDGAWSF
TPDSPDGLYGKRRLREVYAVRSGGFEGRASVPLLWDAERREVVCNESIEIIKYLCGLADADGGGGGGGGLDLWPS
ELRQDIDRWYGVIYPSVNNGVYRCGFAQSQEAYDAAASELFGALDRLEAHLASSRYLCGDRLTLADVCLFTTLIR
FDLVYNTLFRCTRRKLAEYPSLHAYTRDIYQMPRVADTCDMEAIMAGYFKTLFPLNPGGIQPLRPASCDGESLLR
PHGREALPSAAGTPLEAAAVS
>Solyc02g068900.2
MSATGAFERTASTFRNIVSREPGSVFPVESGRYHLYISYACPWASRCLAYLKIKGLDQAIDFTSVKPVWERTKDS
DEHTGWVFASSSTEEAGADLDPLNGAKSIRELYELASTNYSGKYTVPVLWDKKLKTIVNNESAEIIRMFNSEFND
IAENAALDLYPPHLQSLINEANEWIYDGINNGVYQCGFAKKQEPYDEAVQKVYKALDKCEEILSKQRYICGDQVT
EADIRLFVTLIRFDEVYAVYFKCNKKLLREYPNLFNYTKDIFQIPGMSSTVNMEHIKKHYYRSHPSINPFGIIPQ
GPNIDYSSPHDREKFSK*
>Solyc06g083770.2
MYSAQVSSFISIPFPPSKSKTHKLKYPKILHTKLCNSTPKMSLNQNSNTNLINTITKLLWGPSLPPQLLISTVRS
TWSATWQLMMSQLAPSDPTGSYTRPTSQFRLYSNPELKFSPKDLHLYVGLPCPWAHRTLIVRALKGLEDSVPVSI
ASPGIDGSWEFRVFSDPDKDKLVPGLDKANGCKTLREVYKLRRGGYSGRSTVPMLWDMGKKEVLCNESYDIIEFF
NSGLNEISGNPELDLSPPALKVDIRKWNDIIYPNVNNGVYRCGFAQSQEAYNKAAEGLFRTLEMLEDHLAGSRYL
CGDVLTLADVCLFTTLIRFDVVYNVLFKCTKKKLIEFTNLHGYLRDIYQIPKVAETCNMGQIMEGYYKILFPLNP
GGINPIMPSGCEDEVLSKPHNRDCLSLETKVVQHSVS*
>PGSC0003DMG400010333
MARSALDEMSATGAFERTASTFRNIVSREPGSVFPVESGRYHLYISYACPWASRCLAYLKIKGLDQAIDFTSVKP
IWERTKDSDEHMGWVFASSSTEEAGADLDPINGAKSIRELYELASTNYSGKYTVPVLWDKKLKTVVNNESAEIIR
MFNSEFNDIAENAALDLYPPHLQSQINETNDWIYDGINNGVYRCGFAKMQEPYDEAVQKVYKALDKCEEILSKQR
YICGDQVTEADIRLFVTLIRFDEVYAVHFKCNKKLLREYPNLFNYTKDIFQIPGMSSTVNMEHIKKHYYGSHPGI
NPFGIIPQGPNIDYSSPHDREKFSK
>PGSC0003DMG400020077

MSLNQNSNTNLINTITKLLWGPSLPPQLLISTVRSTWSTAWQLMMSQLAPSDPTGSYTRPTSQFRLYSNPKLKVS
PKDLHLYVGLPCPWAHRTLIVRALKGLEDSVPVSIASPGIDGSWEFRFFSDPDKDKLVPSLDKANGCKTLREVYK
LRRGGYSGRSTVPMLWDMEKKEVLCNESYDIIEFFNSGLNEIAGNPELDLSPPALKDDIRKWNDIIYPNVNNGVY
RCGFAQSQEAYDKAAEGLFRTLEMLEDHLGGSRYLCGDVLTLADVCLFTTLIRFDVVYNVLFKCTKKKLIEFTNL
HGYLRDIYQIPKVAETCNMGQIMEGYYKILFPLNPGGINPIMPSGCEDEVLSKPHNRDSLSLETKVVQHSVS
>Sb03g044550
MPTLGTASSLLLTHRHRLLAARTHSASIKMARSALDEVTDTGAFDRSPSTFRSSVSRDGRFPVVAGRYHLYVSYA
CPWASRCLAFLKLKGLDHAIGVTAVKPIFERTKETDDHLGWVFPAAADEEPGAEPDTLNGARSVRELYEIASSNY
AGKPTVPVLWDKQLKTVVNNESSEIIRMLNTEFNDIAGNPGLDLYPPHLRASIDEANELVYDAINNGVYKCGFAK
KQGPYDEAVARLYEALDKCEEILGKQRYICGNQLTEADIRLFVTLIRFDEVYAVHFKCNKKLLREYPNLFNYTKD
IYQIPGISSTVNMEHIRKHYYGSHPSINPYGIIPAGPNIDYNAPHDRERFGA
>Sb04g036980
MWATSPSPHPLHLRRPPPPPFLVRRHNKRLDRIAASQDPLTALTRVLWGRALPPAQLVLAVRHGWTAAWQLLMRQ
LAPSDPSTGAFTRTPARFPAVAGEPSSRLHLYVGLPCPWAHRTLVVRALLGLEARLPVSVAVPGDDGAWSFTPGS
PDRLYGKRKLREVYTLRSGGFEGRASVPMLWDAERREVVCNESIEIVKFLCGLVGDGAGGLDLWPPELRQDIDRW
YGLIYPSINNGVYRCGFAQTQEAYDAAATELFGALDKLEAHLAGSRYLCGDRLTLADVCLFTTLIRFDLVYNTLF
RCTRRKLAEYPSLHAYTRDIYQMPKVAETCDTEAIMAGYFKTLFPLNPGGIQPLPPATCDRESLLRPHGREALSS
AADTPLEAAAVS
>Thecc1EG029392
MAQSALDEMSDSGAFMRTASTFRNIISRDPNSRFPPESGRYHLYVSYACPWASRCLAYLKIKGLDKAISFTSVKP
IWERTKETDEHMGWVFPTSNTEEPDAEPDPFNGAKSIRKLYELASTNYTGKYTVPVLWDKKFKTIVSNESAEIIR
MLNTEFNDIAENPALDLYPSHLRAQIDETNEWIYSGINNGVYKCGFARQQGPYDEAVKQLYEALDKCEEILSKQR
YICGNVLTEADVRLFVTLIRFDEVYAVHFKCNKKLLREYPNLFNYTKEIYQIPGISSTVNVQHIKKHYYGSQPSI
NPFGIIPLGPNIDYSSPHDRARFSA
>Thecc1EG046844
MLTLLCRTPSALGVNNSSSLPFFAFKRTFQMAQSALDEMSDSGAFMRTASTFRNIISRDPNSRFPPESGRYHLYV
SYACPWASRCLAYLKIKGLDKAISFTSVKPIWERTKETDEHMGWVFPTSITEEPGAEPDPFNGAKSIRELYELAS
TNYTGKYTVPVLWDKKFKTIVSNESAEIIRMLNTEFNDIAENPALDLYPPHLRAQIDETNEWIYSGINNGVYKCG
FARKQGPYDEAVKQLYEALDKCEKILSKQRYICGNILTEADVRLFVTLIRFDEVYAVNFKCNKKLLREYPNLFNY
TKDIYQIPGISGTVNVEHIKKTYYGSHPSINPSGIIPLGPNIDYSSPHDRARFSA
>Thecc1EG003927
MHCISLPSSHHPLPSPPKTTFKLTCHVVPRMSLDNNNNSRSNGFNIINNISKLLWGPSLPPGLLISTVRTAWTST
WQIMMSQLAPSDPSGGYTRPPSKFRLSHQNPTASTTKLHLYVGLPCPWAHRTLIVRALKGLEEAVPVSVAAPGLD
GSWEFKDIPDKDKNRDKDILVPSRDKVNGCQNLKEVYRLRKGGYNGRATVPLLWDVDKKEVVCNESYDIIEFFNS
GLNGLAQNPGLDLSPVELKGKIEEWNRAIYPNVNNGVYRCGFAQSQEAYDVAVNGLFSTLDRIDDHLGGSRYLCG
DRLTLADICLFTTLIRFDLVYNVLFKCTKKKLLEFTNLHAYMRDIYQIPKVAATCNFPVIMDGYYQILFPLNPGS
IRPVMPIGCEHEFLSRPHNRESMSSVGKSVQYVL*
>GSVIVG01012071001
MLKKKKKTTHRNGLHVDLGKEVNSILVNNEEEKDLAAISLHFFLPRLPGCFQFIARFLCSSNQTGSFRDKHILHT
LLMARSALDEMSESGAFMRTASTFRNFVTRDPNSPFPAESGRYHLYVSYACPWASRCLAYLKIKGLDKAISFTSV
KPKWERTRDTDEHMGWVFPASDTEEPGAGPDPLNGTRSIRELYELASTNYSGKYTVPVLWDKKLKTIVNNESSEI
IRMFNTEFNDIAENPSLDLYPPHLQAQIDKINDWIYSGINNGVYKCGFAKKQEPYDEAVKNLYEALDKCEEILSK
QRFLCGNTLTESDIRLFVTLIRFDEVYAVHFKCNKKLLREYPNLFNYTKEIFQIPGMSSTVHVEHIKKHYYGSHP
SINPFGIIPQGPDVDYSAPHDRERFSS*
>GRMZM2G105005
MITTRTPTLSTAASLLLTHRHRILAARTLSVSVSAAAGSSSIPVKMTARSALDEVTDTGAFDRSPSTFRSSVSRD
GRFPAVAGRYHLYVSYACPWASRCLAFLKLKGLDHAIGVTAVKPIFERTKESDEHLGWVFPAAADEEPGAEPDPL
NGARSVRELYEIASSNYAGKPTVPCSIAFQVLWDKQLKTVVNNESSEIIRMLNAEFNGIARNPGLDLYPAHLRAS
IDEANELVYDAINNGVYKCGFAKKQGPYDEAVTRLYEALDRCEEILGKRRYICGDQLTEADIRLFVTLIRFDEVY
AVHFKCNKKLLREYPNLFNYTKDIYQIPGISSTVNMEHIRKHYYGSHPSINPYGIIPAGPNIDYNAPHDRERFSA
>GRMZM2G150167
MRAREMNARSALDEVTDTGAFGRSPSTFRSSVSRDRRFPAVAGRYHLYVSYACPWASRCLAFLKLKGLDHAIGVT
VVKPIFERTKESDEHLGWVFPAADDEEPGAEPDLLNGARSVRELYEIARSNYAGKPTVPVPWDKQLKTVVNNESS
EIIRMLNDEFNGITRNPGLDLYPAHLRASIDEANELVYDAINNDVYKCGFAKKKDDRVLVPDLGSLTSIHDRARE
LFYYLKGGQVDYGEEHSKACSHNRFGRIYHTGHYPICYEHNPVHFVGHSAGAQVVRVLQQMLADKDRDNYWKMMH
KYIGSDVTSLVTLPVIIFEPMTMLQKMAEKLGCL
>GRMZM2G136185
MRTCQMTARSALDEVTDTGAFGRSPSTFRSSVSRDRWFPAVAGRYHLYVSYACPWASRCLAFLKLKGLDHAIGVT
VVKPIFERTKGSDEHLGWVFPAAADDEPDAEPNPLNGAQSVRELYEIARSNYAGKPTVPVLWDKQLKTVVNNESS
EIIRMLNDEFDGITRNPGLDLYPAHLRASIDEANELVYDAINNDVYKCGFAKKKDDFVLVPDLGSLTSIHDRALS

CLLRAQPSAFCGALCWRAGCEGAATDACRQGIGETQTILRLLSVVLHSTPDRDNYWKMMHKYIGSDVTSLVTLPV
IIFEPMTMLQKMAEVCTMFRIRLLEIYTSHLQLKDVSAIV
>GRMZM2G009588
MTARSALDEVTDTGAFGRSPSTFRSFVSRDRRFPAVAGRYHLYVSYACPWASRCLAFLKLKGLDHAIGVTVVKPI
FERTKKSDEHLGWVFPAAADEEPGAEPDLLNGARSVRELYEIARSNYAGKPTVPVSLQSRHSKSTKQAADLIPPS
SFPVLWDKQLKTVVNNESSEIIRMLNDEFNGITRNPGLDLYPAHLQASIDEANELVYDAINNSVYKCGFAKKKDD
RVLVPDLGSLNSIHDRARELFYYLKGGQVDYGEEHSKACSHNRFGRIYHTGHYPVCYEHNPVHFVGHSAGAQVVR
VLQQMLADKIEHTPFNRIGETQTILRLLSVVLHSTPDRDNYWKMMHKYIGSDVTSLVTLPVIIFEPMTMLQKMAE
LMEYCELLDKADECEDPYMRMVYASTWAVSVYFAYQRTWKPFNPILGETYEMVNHQGITFLAEQVSHHPPMGVAH
CENEHFTYDITSKVKSKFLGNSLEIYPVGRTRVTLKKSGVVLDLVPPLTKVNNPIFGRTWVDSPGEMVMTNLTFR
DKVVLYFQPCGWFGCRYLNEGSDSDNAPETSSDVSNESDNDNGRSIGRTTQCLAQNICTDQERLSSDDCDSSNQE
PLSVFQYMEHDAPYGRQPLADMIYCKGILSLNKGQQQQLL
>GRMZM2G103725
MAPPSVPRLFPSAASPLSTAMSLLSSASFLRLLRTRRAHALAPRRHAQPPRPRPHRQPLQDGIGCHPPRHGEKRV
TAFARKIDLARRWDPLPNPRPPESSAGAAGSCDPWQGTFLGSRVHRRRSALVSPPTTPRCCRHGLLIDACSVHGL
MRARQMTARLALDEVTDTGAFDRSPSTFRSSVSRDGRFPAMAGRYHLYVSYACPWASRCLAFLKLKGLDHAIDVM
VVKPIFERTKESDEHLGWVFPAADEEPDAEPDPLNGAWSVRELYEIASSNYAGKPTVPCSIAFRVLWDKQLKMVV
NNESSEIIRMLNAEFNGIARNPRLDLYPAHLRASIDEANELVYDAINNGVYKCGFAKKKDGCVLVQDLGSLTSIH
DSARELFYYLKGGQVDYGEEHSKACGHNRALFCLLRAQPSAFCVALCWRVGCEGAASDACRQGRGSCGGKDQNND
KKPLHLVKGAFGQFFIASCVHLGPVVLGLASTSVAKQSSGGLCSWLLGGKSSELPPLDVPLPGISIPSPLPDFVE
PSKTKVTTLPNGVKIASETSSSPAASVGLYIDCGSICETPASSGVSHLLERMAFKSTVNRTHLQLVREVEAIGGN
VSASASREQMSYTYDALKSYTPEMVEVLIDSVRNPAFLDWEVKEQLQNIKSEIADVSANPQGLLLEALHSVGYSG
ALAKPLMASESAVNRLDVSSLEEFVAEHYTAPRMVLAASGVDHDALISVVEPLLSDLPCVKRPEEPKYVYVGGDY
RCQADSPNTYIALAFEVPGGWNQEKTAMVVTVLQGFPPEGTMRVCDSCFKGMLINLRIDNNQSCLQWLWPELAPG
INHCTAVQR
>GRMZM2G376760
MRARQMTARSALDEVTDTSAFDRSPSTFRSSVSRDGRFPVVAGRYHLYVSYACPWASRCLAFLKLKGLDHAIGVT
VVKPIFEQTKESDEHLGWVFPVAADEEPGAEPDPLNGAQSVRELYEIASSNYAGKPTVPMSLQSRHSKSTKQAAD
LIPPPPFQHAEVQNSPTAKLIPVFHCISGAVGQAVEDGGEQRELQEHPDAQRRVQRHRQEPRAGPHPAHLWASID
EANELVYDAINNGVYKCGFAKKKDGCVLVPNLGSLTSIHDRARELFYYLKGGQVDYGEEHNKACGHNRLSLVMVL
LKIVVVVMDGGNGRGSCGGKDQNNDKKPLHLVKGAFGQIVQTVSLLLLKSIA
>GRMZM2G102216
MWATSPPPLPLQLRRPPHPRFIARRRNKRLDRIAASQDPLTALTRVLWGGALPPAQLVVAVRHGWTTAWQLLMRQ
LAPSDPATGGFARAPARFPAVVGKPSSRLHLYVGLPCPWAHRALVVRALLGLEARLPVSVAVPGDDGAWSFTPGS
PDLLYGKRKLREVYALRGGGFEGRASVPMLWDAERCEVVCNESIEIVKFLCDLAGDGAGDLDLWPPELRQDIDRW
YSIIYPSVNNGVYRCGFAQSQEAYDAAASELFGALDRLEAHLAGCRYLCGDRLTLADVCLFTTLIRFDLVYNTLF
RCTRRKLAEYPSLHAYTRDIYQMPKVAETCDMEAIMAGYFKTLFPLNPGGIQPHPPATCDRESLLRPHGREALSS
AAGTPLEAAAVS

GSTL class

>Aquca_046-00004
MALGNFHEVIPPALDSTSDPPAFFDGTTRLYTALLCPFAQRVWITRNCKGLQEEIKLVPIDIKNRPAWYKEKVNP
ANKVPALEHNNQIIAESLDLIKYIDSNFEGPALYPDDPKKREFAEELLSYTDTFTTAVFRSFKGDAGTDFGAPFD
YLETALSKFDDGPFFLGKFSLVDIAYAPFIERFQPFLLEVKNYDIAVGRPKLVAWVEEMNKIEGYTHTKGDPQQL
LEIHKKIFLA
>Aquca_033_00051
MSFVTLVYIAFCFSTCFHFFFLDIINRNFHEVIPPVLDSTSDPPAFFDGTTRLYTALLCPFAQRVWITRNCKGLQ
EEIKLVPIDLYNRPAWYKEKVNPANKVPALEHNNQIIPESLDLIKYIDSNFEGPALYPDDPNKREFAEELLSYAD
TFTTAVFRSFKGDAGTDFGAPFDYLETALSKFDDGPFFLGEFSLVDIAYAPFIERFQPFLLEVKNYDIAVGRPKL
VAWVEEMNKIEGYTHTKGDPQQLLEINKKIFLA
>Aquca_046_00003
MATGSFEEILPPILNSTSEPPSLFNGTTRLYTSYICPYAQRVWITRNYKGLQEKIKLVPIDLQNRPAWYKEKVYP
TNKVPSLEHNNEIRGESLDLIKYVDSNFEGPSLYPEDPDKRKFAEELLSYSDTFNTAVTTSFKGDAGTEIGAPFD
YLETALSKFDDGPFFLGQFSLVDIAYAPFVERYQAFLLQVKEYDILQGRPKLAAWIQEMNKIEAYRQTKRDSWTQ
EVLEIYKKRFMVPHLTTILKIMSVI
>Aquca_007_00187
MAATLKSSSFLYSLRLNLKSCSINNKLSTIFISHSKSICQFPIMASASVQEIIPKALDATSDPPTLFDGTTRLYT
SLLCPYAQRVWITRNVKGLQDEIKLVPIDVGNRPAWYKEKINPANKVPALEHNNQIKGESLDLIKYIDSHFDGPS
LFPNDDDVEKQEFAKELLAYTDTFNGAVVKLCKGEEGSSGAPFDYFETALSKFDDGPFLLGKFSLVDIAYAPFIE
RFVPFLLEVKNYDSTQGRPKLGAWIDEMNKIEGYNQTKRDPQELIAIHKKVFSA

```
>Aquca_007_00205
MASASVQEIIPKALDATSDPPALFDGTTRLYTSLLCPYAQRVWITRNVKGLQDEIKLVPIDVGNRPAWYKEKINP
ANKVPALEHNNQIKGESLDLIKYIDSHFDGPSLFPNDDDVEKQEFAKELLAYTDTFNGAVVKLCKGEEGSSGAPF
DYFETSLSKFDDGPFLLGTFSLVDIAYAPFIERFVPFLLEVKNYDSTKGRPKLGAWIDEMNKIEAYNQTKRDPQE
LLAIHKKVFSA
>Aquca_046_00005
MITDFPFENENSLEKKGKNKNENHFSKKANNMLRLGLGVVVQVRTATALIQSSSYKYKNINISSSSSSCCCCIKS
TTTSANNNKSFFLIHPFSHRLLSFNKTKAQTQTTTSMATGVQELLPPVLNSASDPPSLFDGTSRLYISYICPYAQ
RAWIARNCKGLQDKIELVAIDLNDRPAWYKEKLYPANKVPALEHNNEVKGESLDLIKYLNSQFEGPALLPDDPAK
LEFAEELFSYTDTFNGGVITAIKGNGDVDAPFDYLEKTLSKFEDGPFFLGQFSLVDIAYAPFIQRYHPLLLDVKK
YDVTTGRPKLTSWIEEVNKIEGYKQTIMDAEMLLGVLKKVMGLN
>487053
MALSPQKCFVEDRQVPLDATSDPPALFDGTTRLYISYTCPFAQRVWITRNLKGLQEEIKLVPIDLPNRPAWLKEK
VNPANKVPALEHNGKIIGESLDLIKYVDINFDGPSLYPEDSAKREFGEDLLKYVDATFVKTVFGSFKGDPVKETA
SVFDHVENALKKFDDGPFFLGELSLVDIAYIPFIERFQIFLDEVFKYEIIIGRPNLAAWIENMNKMVAYTQTKTD
SDYIVNYFKKFM
>938433
MSVGLKVSVCLYPSLTLSARDFSPPSSSYLYLDRKILRPCSGKRWCKSNGKRRTEPILAIVDSSRVPELDSSSEP
VQVFDGSTRLYISYTCPFAQRAWIARNYKGLQNKIELVPIDLKNRPAWYKEKVYSANKVPALEHNNRVLGESLDL
IKYIDSNFEGPSLTPDGLEKQVVADELLSYTDSFSKAVRSTLNGTDSNAADAAFDYIEQALTKFNEGPFFLGQFS
LVDVAYAPFIERFQLILSHVMNVDIKSGRPNLALWIQEMNKIEAYTETRQDPQELVERYKRRVQAEARL
>AT5G02780
MALSPPKIFVEDRQVPLDATSDPPALFDGTTRLYISYTCPFAQRVWITRNLKGLQDEIKLVPIDLPNRPAWLKEK
VNPANKVPALEHNGKITGESLDLIKYVDSNFDGPSLYPEDSAKREFGEELLKYVDETFVKTVFGSFKGDPVKETA
SAFDHVENALKKFDDGPFFLGELSLVDIAYIPFIERFQVFLDEVFKYEIIIGRPNLAAWIEQMNKMVAYTQTKTD
SEYVVNYFKRFM
>AT3G55040
MSVGLKVSAFLHPTLALSSRDVSLSSSSSSLYLDRKILRPGSGRRWCKSRRTEPILAVVESSRVPELDSSSEPVQ
VFDGSTRLYISYTCPFAQRAWIARNYKGLQNKIELVPIDLKNRPAWYKEKVYSANKVPALEHNNRVLGESLDLIK
YIDTNFEGPSLTPDGLEKQVVADELLSYTDSFSKAVRSTLNGTDTNAADVAFDYIEQALSKFNEGPFFLGQFSLV
DVAYAPFIERFRLILSDVMNVDITSGRPNLALWIQEMNKIEAYTETRQDPQELVERYKRRVQAEARL
>AT5G02790
MAPSFIFVEDRPAPLDATSDPPSLFDGTTRLYTSYVCPFAQRVWITRNFKGLQEKIKLVPLDLGNRPAWYKEKVY
PENKVPALEHNGKIIGESLDLIKYLDNTFEGPSLYPEDHAKREFGDELLKYTDTFVKTMYVSLKGDPSKETAPVL
DYLENALYKFDDGPFFLGQLSLVDIAYIPFIERFQTVLNELFKCDITAERPKLSAWIEEINKSDGYAQTKMDPKE
IVEVFKKKFM
>Bostr.2128s0160
MALSPQKNFVEDRQVPLDATSDPPALFDKTTRLYISYTCPFAQRVWITRNLKGLQEEIKLVPIDLQNRPAWYKEK
VNPTNKVPALEHNGKIIGESLDLIKYVDSNFDGPSLYPEDFAKREFGEDLLKYVDATFVKAVFGSFKGDPVKETA
SVFDHVENALQKFDDGPFFLGELSLVDIAYIPFVERFKVFLDEVFKYEIIIGRPKLAAWIEEMNKMVGYSQTKTD
SEYIVNFFKKFM
>Bostr.2128s0161
MASPIVVEDHPAPLDATADPPALFDGTTRLYTSYVCPFAQRVWITRNVKGLQEKIKLVPLDLGNRPAWYKEKVYP
ENKVPALEHNGKIIGESLDLIKYLDNTFEGPSLYPEDHAKREFGDELLKYTDTFVKTMYVSLKGDPSKETAPVLD
YLENALYKFDDGPFFLGQLSLVDIAYIPFIERFQIVLNELFKCDITAERPKLSAWIEEMNKIDGYAQTKINPKEI
VEIFKKKFM
>Bostr.0697s0121
MSVGVKISVCSYPSFALSSRDFSIPSSTLYLDRKILRPVSGRLRCKSYVKRRTEPILAVKESSRVPELDSSSEPV
QVFDGSTRLYISYTCPFAQRAWIARNYKGLQNKVELVPIDLKNRPAWYKEKVYAANKVPALEHNNRVMGESLDLI
KYIDNNFEGPSLAPDGLEKQAVADELLSYTDSFCKAVRSTLNGTDSNTADAAFDHIEQALSKFNEGPFFLGQFSL
VDVAYAPFIERFQLILSDVMNVDITSGRPNLALWIQEMNKIEAYTETRQDPQELVERFKRRVQAEARL
>Bradi1g66030
MNSLAFLCRPSPLTPSASPSISLSVPQSSCIKLPRSRPAAHRHRTAAAARISSRTVAMATAAAPPVIFPKENLPP
SLTSTSEPPPLFDGTTRLYVAYHCPYAQRAWITRNCKGLQDKIKIVAIDLADRPVWYKEKVYPENKVPSLEHDNQ
VKGESLDLVKYIDSNFEGPALLPEDSAKKQSAEELLAYTDEFNKALYSSILSKGDVSEETVAALDKIEAALGKFT
DGPFLLGQFSSVDIAYLPFIERFQIFYSGIKNYDITKGRPNFQKYIEEANKIDAYTQTKLEPQFLLDQTKKRLGI
E
>Bradi1g66020
MAAAARSSAGEVLPPALGAVSEPPPLFDGTTRLYICYFCPFAQRAWVTRNCKGLREEIKLVGIDLQDKPAWYKEK
VYPRGTVPSLEHDGKVTGESLDLIKYIDSNFEGPALLPQDPAKRQFADELIAYAGAFTKALYSPLTSQVDMSDDT
```

VAALDKIEAALSKFSDGPFFLGQFSLADIAYVTILERVQIYYSHLRNYEIAKGRPNLEKYIEEMNKIEAYTQTKN
EPLNLLDMAKRHLKIA
>Brara.B00064
MASPSVLEHRPASLDATADPPALFDGTTRLYTSYGCPYAQRVWITRNFKGLQEKIKLVPLNLGNRPAWYKDKVYP
ENKVPALEHNGKIIGESLDLIKYLDNTFEGPSLFPEDQAKREFGEELLKYTDTFTKTMWASLKGDPFTETAPVLD
YLENALYKFDDGPFFLGQFSLVDIAYIPFIERFQIALNELFKCDITVERPKLSAWIEEMNKIDAYVQTKTDSKEI
VEIFKRKLM
>Brara.B00063
MFFSLLFIERSLVEDRQVPLDATSDLPTLFDGTTRLYISYTCPFAQRVWITRNLKGLQENIKLVPIDLTSLVPAL
EHNGKIMGESLDLIKYVDSNFEGPSLYPLVRPSFDFLHRDFGEDMLKYVDTTFTKVVFGSFKGDPAKDTAPVFDH
LENALQKFDDGPFFLGDLSLVDVAYIPFVQRFQVFLGEVFKYDITAGRPKLAAWIEEMNKMVAYTQTITDSEYVI
NFFKKFMVKVSNLK
>Brara.I03769
MSAGVRVSVCSSYPSLALPSKDVSLPSSSLYFGRKIHRSSFDTDLKLRCNSNGTRRTKPVLAVTSSSRVPELDSS
SEPPQVFDGSTRLYISYSCPFAQRAWLARNYKNLRDKIELVPIDLKNRPAWYKEKVYPANKVPALEHNNRVIGES
LDLIKYIDTNFEGPSLAPNSVEKEAFTDELISYTDSFSKAVRATLSGEDSDAADGAFDYIEKALSKFKEGPFFLD
LFSLVDVAYVPFIDRFHLIFKDVMNVDITAGRPNLALWIEEMNRIEAYTETRQDPQELVERYKKRAQAEARP
>Carubv10003856m
MALSPRKIIVEDRIAPLDATSDQPALFDGTTRLYMSYTCPFAQRVWIIRNLKVTRLKKLKMNKMVAYTQTKTDSD
YTVNYFKKFMNQIFERVLLLNTYSSMASPIVVEERPALLDATADPPALFDGTTRLYTSYVCPFAQRVWITRNFKG
LQEKIKLVPLDLGNRPAWYKEKVYPENKVPALEHNGKIIGESLDLIKYLDNTFEGPSLYPEDHAKREFGDELLKY
TDTFVKTMYMSLKGDPFRETGENIDCSMFRSIKGEIMEDMTGMFFFTAAPVLDYLENALYKFDDGPFFLGQLSLV
DIAYIPFVERFQIVLNELLKCDITAERPKLSSWIEQMNKIDGYAQTKIDPKEIVEIFKKKFM
>Cagra.2240s0029
MALSPRKIIVEDRIVPLDATSDQPALFDGTTRLYMSYTCPFAQRVWITRNLKGLQEEIKLVPIDLFNRPAWYKEK
VNPDNKVPALEHNGKIIGESLDLIKYVDSNFDGPSLYPEDPAKREFGEELLKYVDATFVKTVFGSFKGDPVKETA
SVFDHVENALSKYDDGPFFLGELSLVDIAYLPFIERFQVFLEEVFKYEIIIGRPNLATWIEKMNKMVAYTQTKTD
SDYTVNYFKKFM
>Cagra.2240s0028
MASPIVVEERPAPLDATADPPALFDGTTRLYTSYVCPFAQRVWITRNFKGLQEKIKLVPLDLGNRPAWYKEKVYP
ENKVPALEHNGKIIGESLDLIKYLDNTFEGPSLYPEDHAKREFGDELLKYTDTFVKTMYMSLKGDPFRETAPVLD
YLENALYKFDDGPFFLGQLSLVDIAYIPFIERFQIVLNELLKCDITAERPKLSSWIEQMNKIDGYAQTKIDPKEI
VEIFKKKFM
>evm.model.supercontig_114.37
MATAVQEKLPPALDSTADQPPLFDGTTRLYISDICPFAQRVWITRNYKGLQDKIKLVPLNLRDRPTWYKEKVYPQ
NKVPSLEHNGKVIGESLELIKYVDCNFEGPSLLPDDPAKKDFAEEMVKYTDTFIMFIFTSFKGDTIKEAGPAFDH
LENSLHKFDDGPFFLGGFSLVDIANIPFVERFQLFFLDVWKYDIREGRPKLAAWIEEMNKMDAYKQTKYDPDELL
EYYRRRFLFLLS
>evm.model.supercontig_114.38
MATGAEEDLPLPLDATAEQPNLFDGTTRLYTCYTCPYAQRVWITRNYKGLEDEIKLVPLNLRNRPAWYKEKVYSL
NKVPSLEHNGKVMGESLDLIKYVDSNFRGPSLLPDDPTKREFAEEMLRYTDTFNMAVYISLNGDAVKESGPSFDY
LENALHKFDDGPFFLGGFSLVDIAYIPFVERFQIFFADVWNYDITESRPKLATWIEELNKIDAYKPTKTDPKELV
EFYKQRFQAPQ
>evm.model.supercontig_1.295
MEIQQRIAKIVGRSAGEILPPPLDSSSDPPPLFDGTTRLYISYTCPYAQRVWITRNCKGLEDKIRLVPIDLQNRP
AWYKENVNPANKVPALEHNNEVKGESLDLIRYVDSHFEGPSLFPDDPVKKEFAEELFSYTDTFNKAVVSSLNGKI
NGASDAFDHIEKALSKFDDGPFLLGQFSLVDIAFAPFIERFEPVLLDVKNYDITSGRPKLTAWIEEMNKIEAYKR
TRHDPKEHVERYRRRFLGN
>Ciclev10012511m
MYEIASRDSTEQKAYKKSTRKKVNAMATVVHENLPPVLDSKSEQPPSVFDGTTRLYMAYTCPFAQRVWITRNYKG
LQDEIKLVAISLEDKPVWYKEKVHPANKVPALEHNGKIVGESLDLIKYVDSNFEGPSLLSDDPEKRKFAEELFSY
SDTFNITVYGSFKGDPAKEAGPCFDYLEKALHKFDDGPFLLGQLSLVDIAYIPFVERAQIFLSEVIKYDITAGRP
KLAAWIEELNQVDAYKQTKLKDPKGLVELYKIRYM
>Ciclev10012631m
MATTAQENLPPPLDSKAELPPLFDGTPRLYISYSCPFAQRAWITRNYKGLQDKIKLVPLNLRDRPTWYKEKVYPV
NKVPALEHNGKVIGESLDLIKYVDSNFEGPSLLPDDPEKRKFADELFSHIDTFTNDVYTSFKGDPAKQAGPAFDY
LEKALDKFDDGPFFLGQFSQVDIAYIPFVERFQIFLSEEFKYDITAGRPKLAAWIEELNKLDAYKSTKADPKELV
EFYRSRFVAKQ
>Ciclev10012621m
MASVVYENLPPVLDSKSEQPPPPLFDGTTRLYMAYTCPFAQRVWITRNYKGLQDEIKLVAFDLQDKPAWYKEKVY
PSNKVPSLEHNGKIIGESLDLINYVDSNFEGPSLLPDDPEKRKFAEELFSYSDTFIKDVSTSFKGDTAKEAGPAF

DFLEKALDKFDDGPFFLGQFSLVDIAYIPFVDGYQMILSEGFKYDATVGRPKLAAWIEELNKMDVYKQTKYFKDP
KQLFEYYKIRFLQ
>orange1.1g026482m
MASVVYENLPPVLDSKSEQPPPPLFDGTTRLYMAYTCPFAQRVWITRNYKGLQDEIKLVAFDLQDKPAWYKEKVY
PSNKVPSLEHNGKIIGESLDLINYVDSNFEGPSLLPDDPEKRKFAEELFSYSDTFIKDVSTSFKGDTAKEAGPAF
DFLEKALDKFDDGPFFLGQFSLVDIAYIPFVDGYQMILSEGFKYDATIGRPKLAAWIEELNKMDVYKQTKYFKDP
KQLFEYYKIPFLQ
>orange1.1g022381m
MATSNCADALSLIFSPSFSSNTASKLPHIRKFSQTNLCLHYHNTLSSVLRNKNAIAACVSATMASNVVKEVLPPA
LDSTSEPPPLFDGTTRLYISYTCPYAQRVWITRNCKGLQEKIKLVPIDLQNRPDWYMEKVHLANKVPSLEHNNEV
KGESLDLMKFIDSHFEGPSLFPDDPAKREFAEELFSYTDTFNKTVRSSLQGDGNEASAAFDYLETALSKFNDGPF
LLGHFSIADIAYAPFIERYQPFLLEVKKNDITAGRPKLAAWIEEMNKNEAFNQTRRDPKELVETYKKRFAAKI
>orange1.1g028416m
MASVVYENLPPVLDSKSEQPPPPLFDGTTRLYMAYTCPFAQRVWITRNYKGLQDEIKLVAFDLQDKPAWYKEKVY
PSNKVPSLEHNGKIIGESLDLINYVDSNFEGPSLLPDDPEKRKFAEELFSYSDTFIKDVSTSFKGDTAKEAGPAF
DFLEKALDKFDDGPFFLGQFSLVDIAYIPFVDGYQMILSEGFKYDATIGRPKLAAWIEQ
>Cucsa.307370
MATATVVPEVRPPLLEADAEQPPLFDGTTRLYMAYYCPFAQRAWITRNYKGLQDKIKLVPLNLQNRPAWYKEKVN
PTNKVPALEHNGKVIGESLDLIKYIDSNFEGPSLFPDDAAKRQFGEELIAYTDTFTGAVYPSFKGDPAKEAGPQF
DYLENALQKFDDGPFLLGQFSGVDIAYITFIERFHVFLNEVFKYDITEGRPHLATWIEKFEKIDAYKQTKYDPTA
IVELYKKRFMA
>Cucsa.307350
MAALVEEILPPSLDATAEQPPLFDGTTRLYTAYICPYAQRVWITRNYKGLQDKIKLVPLNLFNRPDWYKEKVYSP
NKVPSLEHNGKVIGESLDLMKYVDSHFEGPSLLPNDPAKREYAEELLSYSDTFNGAMISSFKGDTAKEAGAQFDY
LENALQKFDGPFLLGEISLVDIAYIPFVERFSVFLLEVFKIDITKGRPKLAAWIEEFNKIDAYKQTKADPKLVVE
VYTKRFLG
>Cucsa.165850
MATSTLGSTIGFSYSSSSSDISAYCILSSTTRFSFSSIIPSPLKLQKQPPGRRARALIAVKMAAQAFQEVLPPAL
TSVSEPPPIFDGTTRLYISYTCPYAQRVWITRNCKGLQNRIQLVPINLQDRPSWYKEKVYPPNKVPALEHNNEVK
GESLDLIKYIDSNFEGPSLFPDEPEKREFAEELINYVNSFTGSVVSSFKGDGNEADATFDYIESALSKYGDGPFF
LGQFSLVDIAYAPFIERFRPFLLEVKTYDITAGRPKLAAWIEETNMIEGYRQTRRDPQEHVDSYKKRFLVILSNI
SILFFFSFIHKCHSQLASLSDIILNVRANMHPFTSCKFST
>Eucgr.A01200
MASPAAVENLPPPLNSSAEQPPLFDGTTKLYTCYTCPFAQRVWITRNYKGLQDKIKLVPLNLQDRPAWYKEKVYP
MNKVPALEHNGKIIGESLDLVKYVDSNFEGPSLFPEDPEKKKFGKELWSYIDEFIKIVFTSFKGEGPKECGPAFD
HLESALGKFEDGPFFLGQFSGVDIAYIPFVERFQIFLSEAFKYDITAGRPKLAAWIEEMNKIDAYKQTKTDPKEL
VEFYNKRFTAQQ
>Eucgr.J01142
MAMATLKALVGHTSYPSSSPLTSSSDIFTPSAPLCVWRTSRLIRPADSATVCLLRPPAPEPVLARVALARPGRRA
SVSATMATGVKETLPPALTSTSDPPPVFDGTTRLYISYTCPFAQRVWITRNHKGLQDQIKLVPIDLQNRPAWYKE
KVYPANKVPALEHNNEVRGESLDLIKYLDANFGGPSLWPEDPAKKEFAEELFSYTDTFSKSVVSSFKGEGDDQAA
TAFDFIENALSKFEDGPFFLGQFSLVDIAYAPFIERFTPYLQEVKSIDITAGRPKLATWIEEMNKNEAYTQTRRD
PKELVESYKKRFAAQV
>Eucgr.A01198
MATPAVVENLPARLDSSAEQPPLFDGTTRLYTSYACPFAHRIWITRNYKGLQDKIKLVPLNLQDRPAWYKEKVYP
VNKVPALEHNGKIIGESLDLIKYLDSNFEGPPLFPDDPERKKFGEELWSYVDEFVSVVFNSFKGEGPKECASAFD
HVENSLGKFEDGPFFLGQCSGVDIAYIPFVERFQSYLSEVWNYDITAGRPKLAAWIEEMDKVDAYKQTKNLSNTK
EFVEYWKARLRAQL
>Eucgr.A01201
MASPAVVENLPPSLNSSAEQPPLFDGTTKLYTCYTCPFAQRVWIARNYKGLQDKIKLVPLILEDRPAWYKEKVYS
VNKVPALEHNGKIIGESLDLIKYVDSNFEGPSLFPDDPEKKKFVEELWSYVDEFIKIVYTSFKGEGPKECGPAFD
HLESALGKFEDGPFFLGQFSGVDIAYITFVERFQIFLSEAFKYDITAGRPKLAAWIEEMNKIDAYKQTKTDPKEL
VEFYKKRFTAQQ
>Eucgr.A01202
MASPAVVENLPPSLNSSAEQPPLFDGTTKLYTCYTCPFAQRVWIARNYKGLQDKIKLVPLILEDRPAWYKEKVYS
VNKVPALEHNGKIIGESLDLIKYVDSNFEGPSLFPDDPEKKKFVEELWSYVDEFIKIVYTSFKGEGPKECGPAFD
HLESALGKFEDGPFFLGQFSGVDIAYITFVERFQIFLSEAFKYDITAGRPKLAAWIEEMNKIDAYKQTKTDPKEL
VEFYKKRFTAQQ
>Eucgr.A01203
MASPAAVENLPPPLNSSAEQPPLFDGTTRLYISYVCPYAQRVWITRNYELQGLQDKIKLVPLNLPDRPAWYKEKV
YPVNKVPALEHNGKITGESLDLIKYVDSNFEGPSVFPEGPEKKKFGDEMWSYVDEFLKMVYTSFKGEGPKESGSA

FDHLESALGKFEDGAFFLGQFSGYVDIAYIPFVERFQIFLSEVWKDVITAGRPKLAAWIEEMNKIDAYKQTKTDP
KELAEFYKKHFTVMLLTQIQFHVTNFS
>Eucgr.K03194
MATDPKAGETLPPPLDSTSNPPSLFDGTTRLYITYRSPFAQRAWITRNCKGLQDEIKLVPLSLTDKPAWYREKVY
PVDKVPSLEHDNKVIGESLDLMKYIDSKFEGSSLFPTDLTKRDYGEELWSYSLKFIETMYSALNGETVKELGPPL
DHLDRALQKFDDGPFFLGQFSLVDIAFIPFVERIQSLLSDVWKHDITLGRPNLASWIEEMNKIDAYTQTKYDPQD
IIAYMKTRFL
>Eucgr.K03192
MATDLKARETLPPPLDSTSNPPPLFDGTTRLYITYRSPFAQRALITRNCKGLQDEIKLVPLSLTDKPAWYREKVY
PVDKVPSLEHDNKVMGESLDIMKYIDSKFEGPSLFPTDPAKRDYGEELWSYSLEFVETMYSALKGETVKELGPPL
DHLDRALQKFDNGPFFLGQFSLVDIAFIPIVERIQLLLSDVWKHDITLGRPNLASWIEEMNKIDAYTQTKYDPQD
IVAYIKTHFLVKH
>Thhalv10023665m.g
MALSPLLVEDRQVPLDATSDPPTLFDGTTRLYISYTCPFAQRVWITRNLKGLQEKIKLVPIDLSNRPAWFKEKVN
PANKVPALEHNGKIIGESIGLIKYVDNNFDGPSLYPEDPARRDFGEEMLKYVDTTFIKSVFGSFKGDPAKDTESV
FDHVENALQKFDDGPFFLGELSLVDVAYITFIQRFQVFLGEVFKYDIVVGRPKLAAWIAEMNKMVAYTQTKTDSE
YVINFFKKFI
>Thhalv10023667m.g
MASPLVVESLPASLDATADPPTLFDGTTRLYTSYVCPFAQRVWITRNFKGLQEKIKLVPLDLGNRPAWYKEKVYP
ENKVPALEHNGKIIGESLDLIKYLDTNFEGPSLYPEDHAKREFGEELLKYTDTFIKTMYVSLKGDPFQETAPVLD
YLENALHMFDDGPFFLGQFSLVDIAYVPFIERFQIVLNELFKCDITAERPKLSAWIEEINKINAYAQTKIDPKEI
VDIFKKKFM
>Thhalv10010587m.g
MMMSVGVKASVCSPYSSLSISSRDFSFPSSSSLYVGRKILRPSSGVYLNRRCNSSVLAVIESSRVPELDSSSEPI
QVFDGTTRLYIAYTCPFAQRAWIARNYKGLQDKIELVPIDLKNRPAWYKEKVYPANKVPALEHNNRVIGESLDLI
KYIDTNFEGPSLTPDGVEKQAVAEELLSYTDSFSKAVRSTFNGAYNDAADGAFDYIEQALSKFKDGPFFLGQFSL
VDVAYAPFIGTFQIILKDVMNVDITSSRPNLALWFQEMNKIEAYTETRRDPQVIVERYKKLIQAEGRL
>gene18167-v1.0-hybrid
MASTQEVLPTPLDATSNPPPLFDGTTRLYTYYSCPFAQRVWITRNYKGLQDQIKLVPLNLANRPAWYKEKVYPEN
KVPALEHNGKIIGESLDLIKYVDSNFEGPSLFPKDPEKAKFGEELISHVGTFTGALYTAFKADDTVKQADAQFDY
LENALKKFDDGPFLLGQFSLADIAYIPFVERFQSFLSAVWKYDITAGRPKLAAWLEEINKIDAYKVTKTDPNELV
GFYKKRFLEQQ
>gene23342-v1.0-hybrid
MTFTMKVGFRGIAPTSSSLKSRPHVLDSLYVAKFPNTTVLCPPKLRLQAKTIRASLSATMATGVTSVQEALPPAL
TSTSDPPSIFDGKTRLYISYTCPFAQRAWIARNCKGLEEKIELVPINLQDRPSWYKEKVYPPNKVPSLEHNNEVK
GESLDLIRYIDSNFEGPSLFPEDPAKREFAEELFTYTDSFSKPVISFFKGEGTEAAAGAAFDYIETALSKFEDGP
FFLGQFSLVDIAYAPFIDRFQHFALDVKKYDITAGRPKLAAWIEEMNKNEGFNKTRRDPKELVEIYKKRFSPQP
>gene23340-v1.0-hybrid
MTTLKVSFRGTALPASSSPQTSRHRFSDSVTFSLPKKSLIPKFPNTTILCHPKLVLQASFGKKTRASVSAIVATG
VQEVLPPALTSSSVPPTLFDGKTRLYVSYTCPFAQRAWIVRNCKGLEEKIELVPLDLLDRPSWYKEKVNSTNKVP
SLEHNNEVIVESLDLIKYMDCNFEGPSLFPDDPAEREFAEELFSYTDSFNTSVFSFFKGDGTEAAAGAAFDYIET
ALLKFEEGPFFLGQFSLVDIAYAPFIERFQPFALDVKKFDITAGRPKLAAWIQEMDKNNAYNRTRRDPKVHVEIY
KKRFPVQV
>Glyma.19G177200
MATAGVQEVRVPPLTSTSEPPSLFDGTTRLYISYICPYAQRVWITRNYKGLQDKIKLVPIDLQNRPAWYKEKVYP
ENKVPSLEHNGKVLGESLDLVKYIDDNFEGPSLVPSDPAKKEFGEELISHVDTFTKELYSALKGDPIHQAGPAFD
YLENALGKFGDGPFFLGQFSWVDIAYVPFVERFQLVFADVFKHDITEGRPKLATWIEEVNKISAYTQTRADPKEI
VDLFKKRFLAQQ
>Glyma.13G135600
MLMATLGLQAVRPPPLTSTSDPPPLFDGTTRLYISYSCPYAQRVWITRNYKGLQDKIKLVPIDLQDRPAWYKEKV
YPENKVPSLEHNGKVLGESLDLIKYVDVNFEGTPLVPSDPAKKEFGEHLISHVDTFNKDLNSSLKGDPVQQASPS
FEYLENALGKFDDGPFLLGQFSLVDIAYIPFIERYQIVFAELFKQDIAEGRPKLAAWIEEVNKIDAYTQTKNDPQ
EIADKYKKRLLSQQ
>Glyma.13G135500
MASLGVRPVLPPPLTSISDPPPLFDGTTRLYISYSCPYAQRVWIARNYKGLQDKINLVPINLQDRPAWYKEKVYP
ENKVPSLEHNGKVLGESLDLIKYVDANFEGTPLFPSDPAKKEFGEQLISHVDTFSKDLFVSLKGDAVQQASPAFE
YLENALGKFDDGPFLLGQFSLVDIAYIPFVERFQIVFAEVFKHDITEGRPKLATWFEELNKLNAYTETRVDPQEI
VDLFKKRFLPQQ
>Glyma.10G047700
MASLGVQAVHPPPLTSTSDPPPLFDGTTRLYISYSCPYAQRVWIARNFKGLKDKINLVPINLQDRPAWYKEKVYP
ENKVPSLEHNGKVLGESLDLIKYVDENFEGTPLFPRDPAKKEFGEQLISHVDTFSRDLFVSLKGDAVQQASPAFE

YLENALGKFDDGPFLLGQFSLVDIAYIPFAERFQIVFAEVFKHDITEGRPKLATWFEELNKLNAYTETRVDPQEI
VDLFKKRFLSQQ
>Glyma.03G176300
MATPSVLEVRPPALTSISEPLPFFDGTTRLYICYLCPFAQRAWITRNCKGLQDKIELVPIDLKNRPAWYKEKVYP
TNKVPSLEHNSKVLGESLDLIRYIDANFEGAPLFPTDPAKREFGEQLISHVDTFTSGIYPTFKGDPIQQTSAAFD
YLENALGKFDDGPFFLGQFSLADIAYVSFLERFQIVFSEIFKHDITAGRPKLATWIQEGNKIDGYKQTKVDREEY
LEAFKKKFLA
>Gorai.002G252300
MATETVESLPPTLEANAEQPPLFDGTTRLYTSYTCPFAQRVWITRNYKGLQDEIKLVPLNLQNRPAWYKEKVYPV
NKVPSLEHNGKIIGESLDLIKYVDSNFEGPSLVPNDPDKKRTLEELLSYADKFMEMLFASFKGDPEKEAGAAFNY
LEDALKKYDDGPFLLGRDFSLADIAYIPFVERFQIFLSEVFKYDTIAGRPKLAAWIEELNKIDAYKQTKTVDPKQ
LVEYYKQRFMVPKLSTPLRL
>Gorai.013G024200
MAVCYISGIGFGPSPTFQYSPNRTIPFSSNQITTTTLPRTKALSCFHHATTSVQPTFSFHCKTKTHFPFRPPWPA
SNKPTTISSAMSSGYVQEDLPPALDSTSDPPPIFDGTTRLYISYTCPYAQRVWITRNCKGLQDKIKLVPIDLKNR
PTWYKENVYPPNKVPALEHNNEVKGESLDLIKYIDSHFEGPSLFPDDPAKKQFADELLSYIDSFYKTVTSSFKGE
GTEAGIAFDNIETALTKFEDGPFFLGQFSLVDIAYAPFIERFHPFLLDVKKYDITLGRTKLATWIEEMNKNEGYT
QTRCDPKELVESYKKRFMVIKI
>Gorai.006G177600
MATPSVEYLPPPLDATAQQPAIFDGTIRLYLAYPCPYAQRVWIARNCKGLQDKIKLVPLNLQNRPAWYKEKVYPE
NKVPSLEHNGKVIGESLDLIKYVDSNFEGPSLLPDDADKKEYFETLLSHMDEFLGLVYATFKGDSTKDADAAFDH
LETALAKYDGPFLLGNEFTLADIAFIPFVERFQIYLSEVFNYDLTAGRPKVAAWIEAADKIDAYKQTKKSDPKEI
VEIYKRIFSAQK
>Lus10003994
MAATALDRTVPENLPPSLDSTAAQPPLFDGTTRLYTAYICPYAQRVWITRNFKGLQNEIKLVPLNLQDRPTWYKD
KVYPLNKVPALEHNGKIIGESLDLIKYLDSNFEGPSLTPGDRVKRQFADELLSSCDTFVGSVYSSFKGDLATEVD
PVFDYLENALSRFHDGPFFLGQFSLVDIAYIPFIERFQVFLSEVFKYDITSGRPGLAALIEECNKIEAYKPTKVD
PKETVLNLSKRFLVTDVHLL
>Lus10015049
MAATLLDKSVAEDLPPVLDASSEQPPLFDGTTRLYTNYQCPFAQRTWIARNFKGLQDKIKLVPLDLQNRPGWYPE
KVYAGNKVPALEHKGKIIGESLDLVKYLDTNFEGPSLLPDDPAKKQFAEEMFTYTDTFNGKVFTAFKGDAVKEAG
PVLDYMEEALSKFNDGLFFLGQFSMVDIAYIPFVERFQIFFSEVFKHDITQGRPKLAAWFEEMNKVEAYKQTKLK
DPKQFVELYSKRFLGK
>Lus10040347
MTTFALNGSSAAFSLRPSSSLTRHIQPVGTLHNESIASAKLPSWSSGAIFTFNLRHKFRVAPIFATVVAAAGVGE
NLPQPLTSASDPPPIFNGTTRLYLSYTCPYAQRVWITRNCKGLQDEIKLVPLDLKDRPSWYKDKVYPPNKVPALE
HNKEVKGESLDLMKYIDTHFHGPPLLPHEPAKREFAEELLGSVGSFYKAVTSTLKGEADEGLLSTAFDRVETALS
KFDDGPFFLGQFSLVDIAYVPFIERFQPAVLDMRNYNITSRRPKLAAWIEEMNKNEAYNETRRDSKEHVENYKKR
FAVFSLLSEHIIFTNFAVISTWFH
>Lus10019895
MVFNLHIKAATPRAVLGLASAIHDDVRAPPINFHPFSLRLSTDPKCIRVQLVQPRFPSVSPAANGDRRRGCQVAG
ARDTEKEGDLSNSEAEKISSERHDDGLGKAEGDKSYVTSVKTVALCVGSAVAFGIGIGLKEGVGKASEFFAGYLL
EQSLSVDNLFVFVLIFNYFKVPLKYQNRVLSYGIAGAIIFRLSLILLGTVTLQRFEAVNLLLAGILLYSSFKLLF
VSEEDDTDLSDKFIVKICQRFIPVTSKYDGDRFITMHEGVWKATPLLLTVAVLELSDIAFAVDSIPAVFGVTRDP
FIVISSNLFAILGLRSFFTLISQSMSELEYLQPSIAIVLGFIGCKMILDFFAKSSIVSPLRVMLVGSSYISNARE
SLPPSLDSASHAPPPFNGTATARLYTSYRSPFAQRAWIARNCKGLKDKIQLVPINLEDKPSWYKQVNPQGKVPAL
EHNGKIIAESLDILRYVDTNFDGPPLFPDDTERKSFAEELLSYSDTFTEMILNSFKGETVREADPAFDVVEAALG
KFKDGPFFLGRQFSLVDIAYIPFVERAEIFLPAAWNYDITGGRPQLAAWIQEMKRVDGVKETKSDSREVVGFYKN
RFLAQDQ
>MDP0000191661
MAYRHEXLPPXLDSTSSPPPLFDGTTRLYVNYSCPFAQRVWITRNYKGLQDKIKLVPINLQNRPDWYKEKVYPGN
KVPALEHNGKVIGESLDLIKYIDSNFEGPSLXPKDDPERKKFGEELLTYVDTFTGSLYRSFKGDAVKAADEQFDY
LENALKKFDDGPYFLGQFSLVDIAYIPFIERFQVFLSDVFKYDITAGRPKLAAWFEEINKIEAYKVTKTDPKELV
AFYKKRFLEQQ
>MDP0000300987
MAYKYDFSFSWHEELPPPLDSTSSPPPLFDGTTRLYVNYTCPFVQRVWITRNYKGLQXKIKLVGIDLENRPAWYK
EKVYPGNKVPSLEHNGKVIGESLDLIKYLESNFEGPSLVPXDDPXRKKFGXELLXYVXTFVGSLYRSLKGDTVKA
ADEQFDYLENALKKFDDGPYLLGQFSLVDIAYIPFVEXFQVFLSDVFKYDITAGRPKLAAFLEEINKIEAYKGLQ
XKIKLVGIDLENRPAWYKEKVYPGNKVPSLEHNGKVIGESLDLIKYLESNFEGPSLVPXDDPERKKFGXELLXYV
DTFVGSLYRSLKGDTVKAADEQFDYLENALKKFDDGPYLLGQFSLVDIAYIPFVEKFQVFLSDVFKYDITAGRPK

LAAFLEEINKIEAYKVTKADPKEIVAAYKKKFLVHSLLLTYNSKFVLSRSFYIHYAGLITSAXYFRYRSNSXPVX
YSAADAVX
>MDP0000188674
MAYKHEELPPPLDSTSSPPPLFDGTTRLYVNYTCPFVQRVWITRNYKGLQDKIKLVGIDLENRPCLVPSLEHNGK
VIGESLDLIKYLESNFEGPSLVPSDDPERKKFGEELLTYVDTFVGSLYRSLKGDTVKAADEQFDYLENALKKFDD
GPYLLGQFSLVDIAYIPFVEXFQVFLSDVFKYDITAGRPKLAAFLEEINKIXAYKVTKADPKEIXAAYKKKFLEQ
Q
>MDP0000189838
MAALKVSLRGTAAPTCSPSPSLKSWPHISDSLSKTSXIARFPNAAVLSPPKLHLQASPGKKTRASVSATMATGGQ
EVLPPALTSTSDPPPIFDGNTRLYISYQCPYAQRAWIARNCKVVSRTFHCLVLLXLIRMXXKILKEXKXXLRXFH
PCRLALQKVNRTSQGLEEKIQLVPIDLQDRPAWYKEKVYPANKVPSLEHNNEVKGESLDLIRYIDSHFEGPSLFP
DDQAKKEFAEELLSYTDTFNKSVFSSIKEDEIXAAGAAFDYIEXALSKFEDGPFFLGKFSLVDIAYAPFLERFQP
LLLDVKKYDITAGRPKLAAWIEEMDKNVAYKQTRRDPKELVESYKRRLRNEISITKLGVDADVPSFARMXCELLM
VVLRTWRTKS
>MDP0000226223
MAYKHEELPPPLDSTSXPPPLFDGTTRLYVNYTCPFVQRVWITRNYKGLQDKIKLVGIDLENRPAWYKEKVYPGN
KVPSLEHNGKVIGESLDLIKYLESNFEGPSLVPXVRYXEFDCASNEQFDYLENALKKFDDGPYLLGQFSLVDIAY
IPFVEKFQVFLSDVFKYDITAGRPKLAAFLEEINKIEAYKSFFLYSLCWFDNLCXILFVTGATVNLXNILLRTPY
K
>MDP0000176510
MAYKHEELPPPLDSTSSPPPLFDGTTRLYVNYTSPFAQRVWITRNYKGLQDKIKLVGIDLENRPCLVPSLEHNGK
VIGESLDLIKYLESNFEGPSLVPNVRYPEFDCASSNMTLSSQKFDQSDAAKLFLGRPWEKKVWRRIINLCCYLRG
VLVSLIERRHDEQFDYLENALKKFDDGPYLLGQFSLVDIAYIPFVKQFQVFLSDVFKYDITAGRPKLAAFLEVH
>cassava4.1_015100m.g
MALLDKSVPEQLPPVLDATAEQPPLFDGTTRLYTAYTCPFAQRVWITRNYKGLQDKIHLVPLNLQNRPSWYGEKV
YSVNKVPALEHNGKIIGESLDLIKYIDSNFEGPSLLPDDPAKKELAEELFSYTDKFNTTVFTSFKGDVAKESGPA
FDYLENALHKFDDGPFLLGQFSLVDIAYIPFVERFQIFLSEVFKYDTTAGRPKLAAWIEEMNKIGAYKQTKTDPK
QLVEFYKKRILGQ
>cassava4.1_015004m.g
MAATAVDKSVTEQLPQVLDPTAEQPPLFDGTTRLYTAYICPFAQRVWITRNYKGLQDKIKLIPLNLQSRPAWYGE
KVYPTNKVPALEHNGKIIGESLDLIKYLDSNFEGQSLLPDDPAKKEFAEELFAYTDTFNSIVFTSFKGDPAKEAG
PAFDYLENALHKFDDGPFLLGQFSLVDIAYIPFVERFLPFLAEVFKYDLTAGRPKLAAWIEEINKFEAYKQTKLD
PKEIVETFKKRFLGQ
>cassava4.1_012409m.g
MAALAVSYFNVSSPLTSSRDLSTHFSFSFISNSSASFANVKNPIKLHSHSLQFHQVASFLRKSRDAPAPTSASMA
TGSWKEDLPPALTSTSDPPPIFDGTTRLYISYTCPYAQRVWITRNCKGLQDKIKLVPVDLQNRPTWYKEKVYPPN
KVPSLEHNNEVKGESLDLIKYIDSNFDGPSLFPDDTAKKELAEELFAYTGLFNQALVSLFKGDASEAGTALDFIE
TTLSKYDDGPFFLGQFSLVDIAYAPFIERFQPALLDLRKYDITEGRPNLSAWIEEMKKNEGYNQTRRDPKEHVET
YKKRFLAPL
>Medtr1g116270
MAISSSSSSLVTNNNSSLFSVSARPQILVTSSLPFMSTTSIHFHYSPHNKLHLRQRFNSVTKSCDVRVTTLAIST
GYVLCNKFFHHPLLPLHLHLPYLMELPGLYISYKCPYAQRVWITRNTKGLQDKIQLVPIDLQDRPSWYKDKVHPT
NKVPSLEHNNEVRGESLDLIKYIDTHFEGPSLYPSGHDDKEFAEELLSYTDTFYKTVVSYFKGDVTEAGTAFDYL
ETVLSKYDHGPFFLGQFSLVDIAYAPFMERFQPFLMDVKNYDITVGRPRLTAWIELLRKYVMIWEIYLSLNQGRF
MGINNIEGYKITRSDPKELVESYKKQIHEFNVRYHSRSQEFQNGSENQSQMHYIHPGTRNHTTSTDR
>Medtr7g100320
MATTVKEIRTPPLTSNSDPPPIFNGDTRLYISYVCPYAQRAWIARNYKGLQDKIKLVSINLQDRPAWYKEKVYPE
NKVPSLEHNGKVLGESLDLISYIDVNFEGLSLVPSDPAKKEFGDQLISHVDTFTKDLYSSLKGDPIKQAGPAFDY
LENALGKFDDGPFFLGQFSWVDIAYVPFVERFHIVFSEVFKHDITEGRPKLAAWIEVQLKQLSLKLQFSIIAFIY
ELITNQGHQSKLEENFICSEN
>Medtr1g067180
MATIGVKPVLPPPLTSTSQPPPLFDGTTRLYVSYSCPFAQRTWITRNYKGLQNNIHLVPIDLQNRPAWYKEKVYP
ENKVPSLEHNGKVLGESLDLIKYIDANFDGPPLFPNDPAKKEFAEQLLSHVDTFTKELFVSLKGDTVQQSSPTFE
FLENALGKFDDGPFLLGQLSLVDIAYIPFVERFHIVLAEVFKHDITEGRPKLATWIEELNKIDAYTQTRVDPQEI
VDLFKNRFLPKQ
>Medtr1g067170
MASISGVQPPLNSTSEQPPLFDGTTRLYTSYSCPYAQRVWITRNYKGLQDKIELVPIDLSNKPAWYKEKVYPEGK
VPSLKHNGKVLGESLDLIKYVDANFEGTPLFPNHHPAKKEFGPASDFLENALGKFDDGPFFLGQFSLVDIAYIPF
VERFHIVLAEVFKHDITEGRPKLATWIEELNKIGAYTQTRVDPQIIVDKFKERHMNSDLILIFLFATWLKLVHFY
CAASTVKVELQQTSSEIFL
>Migut.J00400

MAAASVQEVLPPILDSTSDPPPLFDGTTRLYVNYECPFCQRVWIVRNYKGLQDKIKLIAIHLQDKPLWFKQKLYP
ENKVPALEHNGKVIGESLDLIKYLDSTFDGPSLLPDDPTKRDFAEEMITYLDTFLKNVFTSLKRDPIEEASGEFD
YLETSLKKFDDGPFFIGELSQVDAAYVPFIERFQIFLQEVFEYDITAGRPKLAAYLEEINKIDAYKQTKCDPKWL
VQLYKTRYMGSG
>Migut.J00399
MATPAAVQEVLPPSLDSTSEPPPLFDGTTRLYTNYQCPYAQRVWIVRNYKGLHDEIKLVGIDLQNRPAWYKEKVY
PENKVPALEHNGKIIGESLDLVKYIDANFKGPALLPDDPAKKKFIEELVTYSDTFLKNVFTSFKAEDPLKQAGSE
FDYVETALQKFEDGPFFTGQFSQGDVVYAPFIERFHILFQEFFKYDITSGRPALAKWIEEINKIDAYKPTKCVPE
VLVQFYKTRYMA
>Migut.F02019
MATIFNNISYHHSIIHCACNNNNTNRINLCPNLGSPPFKKPLILFPRKNLLNYSGISRTVSAISSGVREEALPPA
LDSSSDPPPIFDGTPKLYISYSCPFAQRAWITRNCKGLQDTIKLVPINLKNRPSWYKEKVYPPNKVPSLEHNNEV
KGESLDLIRYIDANFEGPSLFPDDPTKIEFAEELLSFTSSFSKAVTNSLKENSTNEAGAAFDEIEIFLSKFDDGP
FFLGQFSLVDIAYAPFVERYQPFFFEVMSYDITSCRPKLAVWIEEMNKIEAYKQTQRDPKEHVETYRKRLVSLKS
>LOC_Os03g17470
MNSLAFPRGRCSPSPLTRPSTSRSSSVPSSRTIKIRPSSRSVARCNLRDAKNLSPSRTVAMAAAAAAPASSEKEV
LPPSLTSSSEPPPLFDGTTRLYVAYHCPYAQRAWIARNYKGLQDKIKIVAIDLADRPAWYKEKVYPENKVPSLEH
NNQVKGESLDLVKYIDTNFEGPALLPDDSEKQQFAEELLAYTDAFNKASYSSIVAKGDVCDEAVAALDKIEAALS
KFNDGPFFLGQFSLVDIAYVPFIERFQIFFSGIKNYDITKGRPNLQKFIEEVNKIHAYTETKQDPQFLLEHTKKR
LGIA
>LOC_Os03g17460
MEVLPPTMPSASKQPPLYDGETRLINHYICPYAQRAWIARNYKGLQEKIKLVPMDTNDRPAWYKEVYPKNTLPSL
EHNNKIIGESLDLIKYIDINFAGPRLTPDDSEKQRLAEELLAYSDIFNQAVRSALISKDAMTAEAAAALDNIEFS
LSKFDDGPFFLGQFSLVDIAYAPFIDGFQTLFAGIKNYDITEGRANIQIFIKELNKIDAYMHTKQDPSEVIALTK
KKLGVRT
>LOC_Os03g17480
MAAAAAPRSSGKEALPAALGSASEPPRLFDGTTRLYICYFCPFAQRAWIIRNFKGLQDKIELVGIDLQDKPAWYK
EKVYEQGTVPSLEHNGKIMGESLDLIKYIDSHFEGPALLPEDPEKRQFADELIAYANAFTKALYSPLISKADLSA
ETVAALDKIEAALSKFGDGPFFLGQFSLVDIAYVTIIERIQIYYSHIRKYEITNGRPNLEKFIEEINRIEAYTQT
KNDPLYLLDLAKTHLKVA
>Pavir.J34940
MNSLAFPRGCPSPQTLASTSAPVTTNSPPARIKIRRPRREAHRIAARKPSRIVAMAAAAPASSVKEVLPFPLTAA
SEPPPLFDGTTRLYVAYHCPYAQRAWIARNYKGLHDKIKIVAIDLADRPAWYKEKVYPENKVPSLEHNNQVKGES
LDLVKYIDSNFEGPSLLPDDPAKKQFAEELLAFTDAFNKALYSSIISKEDVSEETVAALDKIEEALGKFNDGPFF
LGQFSLVDIAYVPFIERFQIFFENIKNYDITKGRPNLQKFIEEVNKIDAYTQTKQDPQFLLEHTKKRLGIA
>Pavir.Ib01315
MNSLAFPRCGPSPLTPASTSTSAPVTTDAPPARIKIRRPRREAHRIAAQKPSRIVAMAAAAPASFVKEVLPSPLT
SVSEPPPLFDGTTRLYVAYHCPYAQRAWIARNYKGLQDKIKIVAIDLADRPAWYKEKVYPENKVPSLEHNNQVKG
ESLDLVKYIDSNFEGPSLLPDDPAKKQFAEELLAFTDAFNRALYSSIVSKEDVSEETVAALDKIEEALGKFTDGP
FFLGQFSLVDIAYVPFIERFQIFFQNIKNYDITKGRPDLQRFIEEVNKIDAYTQTKQDPQFLLEHTKKRLGIA
>Pavir.J08457
MAAAAAGPGPTSAPAKETLPPALGSTSQPPPVFDGTTRLYICYFCPFAQRAWVTRNFKGLQDKIELVAIDLQDKP
AWYKEKVYPQGTVPSLEHNNEIRGESLDLIRYIDSNFDGPARLPEDAAKKQFADELIAYADAFTKALYSPLMAHA
DMSDEVVAALDKLEAALSKFNDGPFFLGQFSLADIAYVTILERVQIYYSHLRNYDIAKGRPNLERFIEEMNKIDA
YTQTKNDPLFLLDLAKNHLKIA
>Pavir.Ib01317
MAAAAAKEALPPALGSTSQPPPVFDGTTRLYICYFCPFAQRAWVTRNFKGLQDKIELVAIDLQDKPAWYKEKVYP
QGTVPSLEHNNEIRGESLDLIRYIDSNFDGPALLPEDAAKRQFADELIAYADAFTKALYSPLMAHADVSDEVVAA
LDKLEAALSKFNDGPFFLGQLSLADIAYVTILERVQIYYSHLRNYDIAKGRPNLEKFIEEMNKIDAYTQTKNNPL
FLLDLAKNHLKIA
>Pavir.Ia03625
MRHSAMSSLFFFCECSFEMETLPPTLPSACEQPLLYDGTTRLYMSYVCPYAQRAWITRNYKGLQEEIKLVPMDMA
DKPAWYKKVYPKNEVPALEHNKKIIGESLDLIRYIDINFDGPKLITNDPQKQRFAEELLGYSDAFNRAMLEGLRS
KGPVIAEAVAALDKIDSSLSKFDDGPFFLGQFSLVDIAYVPFINGFQIFFAGIKNYEITKGRVHIQKFIEELNKI
DAYTQTKQDPEVLLALTKKKFGI
>Pavir.Ib01200
MEVLPPTLSSACEQPLLYDGTTRLYMSYVCPYAQRAWITRNYKGLQEEIELVPMDMADKPAWYKKVYPKNQVPAL
EHNKKIIGESLDLIRYIDTNFDGPKLITNDPQKQRFAEELLGYSDAFNRAMLDGLRSKGPVTAEAVAALDKIDSS
LSKFDYGPFFLSQFSLVDIAYAPFIDGFQIFFAGIKNYDITKGRVHIRKFIEELNKIDAYMQTKQDPEVLLALTK
KKFGV
>Phvul.001G173500

MATAGVQEARIPPLTSTSEIPPLFDGTTRLYISYLCPYAQRVWITRNYKGLQDKIELVPIDLQNRPAWYKEKVYP
ENKVPSLEHNGKVLGESLDLVKYIDDNFEGQSLVPSDPAKKEFGEQLISYVDTFTKELYSALKGDPIQQASPAFD
YLENSLGKFDDGPFFLGQFSWVDIAYVPFVERFQLVFFEVFKHDITEGRPKLAAWIEEVNKINAYTQTRGDPKDI
VDLFKKRFLAQQ
>Phvul.001G173300
MATSLSDVRPPALTSISEPLPFFDGTTRLYICYLCPYAQRAWITRNCKGLQDKIELVPIDLRNRPSWYKEKVYPL
NKVPSLEHNGKVLGESLDLIRYIDANFEGASLFPSDPAKREFGEQMISHVDTFTSGIYSSYKGDPVEQTSAAFDY
LENALGKFDDGPFFLGQFSLVDIAYVSFLERIQLVFSEIFKHDITAGRPKLATWIQEANKIDGYKQTKVDKEEYL
EVFKIKFLA
>Phvul.007G162000
MATLGVQAFRPPPLTSTSDPPSLFDGTTRLYISYSCPYAQRVWITRNYKRLQAKIQLVPIDLQDRPAWYKEKVYP
ENKVPSLEHNGKVLGESLDLIKYVDANFEGTPLFPGDPAKKEFGEHLISHVDTFNKDTSSSLKGDAEHQDSFAFE
YLENALGKFDDGPFFLGQFSLVDIAYIPFVERFQPVFLDFFKHDITEGRPKLSTWIEEVNKIDAYTETKMDPQEI
VDRFKKRYLSQQ
>Phvul.007G161900
MATFGAQVVRPPPLTSTSDPPPLFDGTTRLYINYDCPYAQRVWIARNYKGLQNKINLIPINLQDRPAWYKEKVYS
ENKVPSLEHNGKVLGESLDLIKYVDVNFEGTPLFPTDPAKKEFGEQLISHVDTFTRDLFISLKGDAVQQASSAFD
YLENALGKFDDGPFLLGQFSLVDIAYIPFVERFHIVFAEAFKHDVTEGRPKFATWLKELNKIDAYTETKVDPQDI
IDLFKKRFLPQL
>Phpat.010G058400
MATLVTSYLHACNATFATTLPRHSRLIQAPSVQFSQVCGKNLGQSFSSPSARILRRNYDFRRELVSVTRSMASSF
ENREVLDSKSASPAIFDGTTRLYFSSRCPYAQRVWVAVKYKGLDEIECVEISLSDKPTWYKEKVYPVGKVPALEH
NGTVTGESMDLLTYLDDHFGGPKLAPTEESKKQAAAELLQYADTFNKLGFTGLSMKSSTPDEIAAAVAPAFDFLE
NALAKFSSEGPLFLGNFGLVDIVYAPFIERFEIAFGGIRNYDIRAGRPRLAKWIEAMDNVEAYSSTKVPRATLLE
LYKKMLENDYFIRVGVAANQNNSSGSSVAVN
>Potri.016G083500
MAAAALDKSVPEKLPPSLDATAEQPPLFDGTTKLYTCYTCPFAHRVWITRNFKGLQDEIKLVPLILQNRPAWYSE
KVYPPNKVPSLEHNGKITGESLDLIKYLESNFQGPSLLPEDPAKKEFAEELFSYTDTFNRTVFTSFKGDPAKEAG
PAFDHLENALHKFGDGPFFLGQEFSLVDIAYIPFVERFCIFLSEVFKYDITAGRPKLAAWIEELNKIEAYKQTKT
DPKEMVEVYKKRFMA
>Potri.006G133500
MGSLDKSVPEKLAPPLDATAEQPPLFDGTTKLYTCYTCPFAQRVWITRNFKGLQDEIKLVPLILQNRPAWYPEKV
YPPNKVPSLEHNGKITGESLDLIKYLESNFEGPSLLPKDPAKKEFAEELFSYTDKFNGTVYTAFKGDLAKEAGPA
FDYLENALHKFDDGPFFLGKEFSLVDIAYIPFVERLNIFLLEVFKYDIAAGRQKLAAWIEEVNKIEAYKQTKTDP
KELVEFYKKRFLVKYQSHLDMPIASNHFPPSKFMVVVVK
>Potri.008G046800
MATLLPMNYSNGSAPLTPSKHLSLRFSVHLSSKKTRRWLDADAVKLPRYHSLQLQPLSKSKTGPISATMATGFSF
FFFFGKILYRSGKEVLPPVLTSNSEPPPVFDGTTRLYISYTCPYAQRVWITRNCKGLQDKIKLVPIDLQDRPAWY
KEKVYPPNKVPSLEHNNEVKGESLDLIKYIDSHFDGPSLFPDDPAKKEFAEDLFSYTGSFSKANNSTFKGEADEA
GAAFDYIETALSKFDDGPFFLGQFSLVDIAYAPFIERFQPALLEFKKYDITAGRPKLAAWIEEMNKIEAYNQTRR
EPKQHVETYKKRFAAHL
>ppa010816m.g
MAYKHEDLSPPLDSTSSPPPLFDGTTRLYVCYTCPFAQRVWITRNYKGLQDKIKLVPINLQNRPAWYKEKVYPEN
KVPSLEHNGKVIGESLDLIKYVDNNFEGPSLFPTDPERRKFGEELITYTDTFTRALYSSFKGDAAKEADAQFDYL
ENALKKFDDGPFFLGQFSLVDIAYIPFVERLQVFLSEVFKYDITAGRPKLAAWFEEINKIEAYKVTKTDPKQLVE
FYKKRFLDQQ
>ppa009087m.g
MAILNVSLRGTAASTSSSSLPSWPHLSNSVPFSLSNKPVIAKFPNTTILSPPKLRLQASFGKKTRASLSATMATG
GQEVLPPALTSTSDPPPLFDGKTRLYISYQCPYAQRAWISRNCKGLEENIQLVPIDLQDRPAWYKEKVYPPNKVP
SLEHNNEVKGESLDLIRYIDSHFEGPSLFPDDPAKREFAEELLSYTDSFNKSVFASFKEDGTKAAGAAFDYIETA
LSKFEDGPFFLGTFSLVDIAYAPFLERFQPFSLEVKKYDITAGRPKLAAWFEEMNKNLAYKKTRRDPKELVESYK
RRFLAQK
>29805.t000040
MAAATALDKSAQENLPPILDAAADQPPLFDGTIRLYTAYACPFAQRVWITRNYKGLQDSIKLVPLNLQNRPSWYP
EKVYSVNKVPALEHNGKIIGESLDLIKYVDSNFEGPSLFPDDPAKREFAEELFSYTDTFSRTVFTSFKGDVAKEA
GPAFDYLENALQKFDDGPFLLGQFSLVDIAYIPFVERLEIFLSEVFKYDITAGRPKLAAWVGEVNKIGAYKQTKT
DPKELVEYYKKRFFAQQ
>29758.t000019
MASLTVSSPLLTSPTLVSSHCSPRLISKRTARFSNTNSTIQLFSYSLQFQPLTSFHDSLRTSKTAPISATMATGI
VKEVLPPALTSTSDPPPVFDGTTRLYISYTCPYAQRVWITRNCKGLQDLIKLVPIDLQDRPAWYKEKVYPPNKVP
ALEHNNEVKGESLDLIQYIDSNFDGPLLFPDDPAKKDLAEELIAYIDSFNKALVSLFKGEANEAGAVLDFIETSL

TKFDDGPLFLGQFSLVDIAYAPFIDRFQPALLDVRDYDITAGRPNLAAWIEEMNKNEAYNQTRRDPKEYVESYKR
RFLAQL
>29805.t000041
MHALSIKSQALSMATDSLYRNAHEVLPPSLNSTSPRPPLSDGTTRLYISYRSPFAQRAWITRNYKGLQDQIELVA
IDLENKPVWYREVYSEEKVPALEHNSKVIVESLNIIKYIDNHFEGPSLFPDDTARREFGEEMISYSETFNEMVYN
SFKGVTVREADPAFDVLEASFKKFDDGPFLLGQFSMRHAL
>Si036831m.g
MNSLAFPRCCPSPLTPASTSSPVTSYAPSSASIKIRRSRRAAHRIAVQKPTRIVAMAATAPPSSVKEVLPSPLTS
ASEPPPLFDGTTRLYVAYHCPYAQRAWIARNYKGLQDKIKIVAIDLADRPSWYKEKVYPENKVPSLEHNNQVKGE
SLDLVKYIDSNFEGPSLLPDDPAKQQFAEELLAYTDAFNKALYSSILSKEDVSEESVAALDKIEEALGKFNDCPF
FLGQFSLVDIAYVPFIERFQIFYSNIKNYDITKGRPNLQKFIEEVNKIDAYTQTKQDPQLLLEHTKKRLGIA
>Si037213m.g
MAAAAGLASSAKETLPPALGSTSQPPPVFDGTTSLYICYFCPFAQRAWVTRNFKGLQDKIKLVAIDLQDKPAWYK
EKVYPQGTVPSLEHNNEIVGESLDLIKYIDSNFDGPALLPGDAAKRQVADELIAYANAFTKALYSPLISHAEVSD
EVVAALDKLEVALSKFNDGPFFLGQISLVDIAYVTILERVQIYYSHLRSYEITKGRPNLEKFIEEMNKIDAYTQT
KNDPLFLLDLAKNHLKIA
>Si039928m.g
MATSAAAPAARLYMGYFCPFSQRTWVTRNFKVLQEEIKLVAIHVQDKPAWYKEKLYPKGTVPSLEHNNEIRAESL
DLIKYIDSNFGGPALLPQDPAKRQFADELITFADTFTKALYSPLMSHVEMSEEAAAALDKIEAALSKFNDGPFFL
GKFSLADISYVKILERVQIYYSHVRNYDITEGRPNLEKFIEEMNKIEAYTQTKYEPMFLLDLAKKHLK
>Si030665m.g
MATAASPAATSGSGSAAAGEKLMKEEVLPPTLASNSQPPNLLDGTTRLYISYICPYVQRVWIARNFKVPIDGGVP
AGLIISSCFRYQNLENFLGQGLQDKIQLVAIDLQDKPAWFLEKVYPPGKVPVLEHNGNVIAESLDLLSYLDANFE
GPKLLPQDDPAKQAFADELIGSSDPVIVALFRAGRAAAGAGAGGDDISELVAPALDKVEEALGRFSDGPFFLGQS
MSAVDMVYAPFVERFKDFFAAAKQYDMTQGRPKLKEWIEELNKIDAYAATWGDRRLQIAAMMSKFGLEIPVA
>Solyc12g044530.1
MLLTQPVLLLSPLPFKKKQLSMASPSVQDLLPPSLDSTSQPPSLFDGTTRLYINYQCPYSQRVWITRNVKGLQDM
IKLVPIDLQNRPDWYKEKVYPKNKVPSLEHNNKVTGESLVLVKYVDCNFEGPSFLPDDQEKRKFVEELIAYSDTT
FVPEVYKSFAKDARTQAGVQFDYLEKALHKFDDGPFFLGQLSQVDIIYAPFVERFHVFMPEGFNYDITTGRPKLA
KWIEEMNNLDGYKQTKVLEQEKMVGYYKNRFLLVPTS
>Solyc04g009530.2
MAASSIGHQIHINVNSPILLPLRTNFSSLSFTFSNARYPLKWNHIGCPKICALPAVSIIASGSSREMLPPALDSS
SEPPAIFDGTPKLYISYSCPYAQRTWIARNCKALQEEIKLVPIDLKNRPDWYKEKVYPANKVPSLEHNNEVKGES
MDLIRYIDSNFEGPSLFPDDPSKREFAEELFSYFDSFYKAVISSLKEDKINDAIAAFDSIETALSKFVDGSFFLG
SLSLVDIAYAPFIERFQPFLLEVKNYDITTGRTKLAAWIKEMNQIEGYTVTKRDPKEHLENYKRRFLSQL
>Solyc00g007030.1
MASSSVQDLLPPSLDSTSQPPSLFDGTTRLYINYQCPYSQRVWITRNVKGLQDMIKLVPIDLQNRPDWYKEKVYP
KNKVPSLEHNNKVIGESFVLVKYVDYNFEGPSFMPDDQEKQKFAEELIAYSDTTFVPEVYRSFAKDARKLAGAQF
DYLEKALHKFDDGPFFLGQFSQVDIIYAPFIERFHVFMPEGFNYDITTGRPKLAKWIEEMNNLDGYKQTKVLEQE
KMVEYYKNRFLPKA
>Solyc12g044520.1
MASPSVQDLLPPSLDSTSQPPSLFDGTTRLYINYQCPYSQRVWITRNVKGLQDMIKLVPIDLQNRPDWYKENVYP
KNKVPSLEHNNKVTGESLVLVKYVDCNFEGPSFLPDDQEKRKFAEELIAYSDTTFVPEVYRSFAKDARTLAGAQF
DYLEKALHKFDDGPFFLGQFSQVDIIYAPFVERFHVFMPEGFNYDITTGRPKLAKWTEEMNNLDGYKQTKVLEQE
KMIEYYKNRFLPKA
>Solyc09g007150.2
MAALSVQEVLPATLESTSEPPSLFDGTTRLYINYQCPYSQRVWITRNVKGLQDKINLVPIDLQNMPDWYKEKVYP
QNKVPSLEHNNKMIGESLDLVKYVDSNFEGPSLLPDDPEKRKFAEELIAYSDIFVPEVYKSFFRDAQTLAGAQFD
YLEKALDKFDDGPFFLGQFSQVDIAYVPFIERFQIFMEKGINYDITSARPKLAKLIEEMNKLDGYKQTKVLDPEK
LVEYYKNLFLKKA
>PGSC0003DMG400011049
MATPRSVQQIRPASLDSTSEPPALFDGTTRLYISYICPFAQRAWITRNFKGLQDKIELVPIDLQNRPVWYKEKVY
PQNKVPSLEHKNKVIGESLDLVKYIDSNFEGPSLLPDDPEKQKFAEELIAYSDTFLKEIYGNFKGDIEKHAGPQF
DYLEKALDKFDDGPFFLGQFSQADIVYAPFVERFQIFLKEVFDYDITSGRPKLAKWIEELNKLDSYIQTKADPKE
VVDLYKKKYLA
>PGSC0003DMG400032537
MAASSIGYQIHINVNSPILLPLRTNFSSLSFTFSNAKYPLKWNHIGCPKICALPAVSIMASGSSREILPPALDSS
SEPPAIFDGTPKLYISYSCPYAQRTWIARNCKGLQEEIKLVPIDLKNRPDWYKEKVYPANKVPSLEHNNEVKGES
MDLIRYIDSNFKGPSLFPDDHSKREFAEELFSYFDSFYKAVISSLKEDKINDAIAAFDSIETALSKFVDGSFFLG
SFSLVDIAYVPFIERFQPFLLEVKNYDITTGRTKLAAWIKEMNQIEGYTVTKRDPKEHLENYKRRFLSQL
>PGSC0003DMG400002018

MAAPSVQEVLPPSLDSTSQPPSLFDGTTRLYISYQCPYAQRVWITRNVKGLQDKINLVPIDLQNRPDWYKEKVYP
PNKVPSLEHNNKVIGESLDLVKYIDSNFEGPSLLPDDPEKQKFAEELIAYSDTFVPEIYRSFMRDAQTLAGAQFD
YLEKALGKFDDGPFFLGQFSQVDIAYVPFIERFQIFMPAGFNYDITSGRPKLAKWIEEMDKLDGYKQTKVLEPEK
LVEYYKNLFLKA
>Sobic.001G412800
MNSLAFPGCPSPLTPASTAPGLHPIPSPRIKIRFQRLEDAAHHHLRARSSASSRTRTVAMASGLPVSSKEVLPSP
LTSASEPPPLFDGTTKLYVAYYCPFAQRAWIARNYKGLQDKIKIVAIDLADRPAWYKEKVYPENKVPSLEHNNQV
KGESLDLVKYIDSNFEGPSLLPEDPAKKQFAEELLAYTDAFNKALYSSLLSKEDVSEETVAALDKIEDALGKFND
GPFFLGQFSLVDIAYVPFIERFQIFYSNIKNYDITKGRPNLQKFIEEVNKIDAYTQTKLDPDFLLEQTKKRLGIA
>Sobic.001G412700
MAAAAGPSAGSVAETLPPALGSTSQPPPVFDGTTRLYICYFCPFAQRAWVTRNFKGLQDKMELVAIDLQDKPAWY
KEKVYPQGTVPSLEHDNEVRGESLDLIKYIDSNFDGPTLLPEDAAKRQFADELIAHANAFTKALYSPLMAHAAVS
DEVVAALDKLEAELSKFNDGPFFLGQFSLADIAYVTILERVQIYYSHLRNYDITKGRPNLEKFIEEMNKIEAYTQ
TKKDPLFLLDLAKNHLKIA
>Sobic.002G421200
MATAAAPDDSTVRETTKASEEVLPPTLGSGSQPPNLFDGTTRLYISYICPYVQRVWIARNFKGLQDKIQLVAIDL
QDKPAWFLEKVYPPGKIPVLEHNGNIIAESLDLLSYLDANFEGPKLFPGDQDPAKQAFADELIANSDSVITAFFK
AGRAYAQGQGDDDISKILAPALDKVEESLGRFSDGPFLLGQSMSAVDMVYAPFVERFKDFFAAVKHYDITQARPK
LKEWIEELNKIDAYAVTWGDRRLQLAALMNKFGVILVLFYLYSTVLSSNRSGLTHLNLYLQIQSPVA
>Sobic.009G033200
MATAAAPDDSTVRETTKAEEVLPPTLGSGSQPPNLFDGTTRLYISYICPYVQRVWIARNFKGLQEKIQLVAIDLQ
DKPAWFLEKVYPPGKVPVLEHNGNIIAESLDLLSYLDANFEGPKLFPGDQDPAKQAFADELIANSDSIIIALFRA
GRAYAEGQGDDDISKLLAPALDKVEESLGRFSDGPFLLGQSMSAVDMVYAPFIERFKDFFAAVKHYDMTQERPKL
KEWIEELNKIDAYTATWGDRRLQLAALMNKFGVISVLFYLYSTVLSSNGSGLTQLCICRYKAQ
>Thecc1EG022060
MATDTVEVLPPPLDATAEQPPLFDGTTRLYTCYTCPFAQRVWITRNYKGLQDKIKLVPLILQNRPAWYKEKVYPE
NKVPALEHNGKIIGESLDLIKYVDSNFEGPSLLPDDPEKKKFFEELLSYLDTFVRTVFTSFKGDPAKEVGAVFDY
LENALQKFDGPFFLGQISLADIAYIPFVERFQIFLLEAFQYDIIAGRPKLAAWIEEMNNIDAYKQTKTDPKELVA
FYKQRFMGQK
>Thecc1EG043031
MATGSVREVLPPALDSHSDPPPIFDGTTRLYISYTCPYAQRVWITRNCKGLQDKIKLVPIDLKNRPAWYKQKVYP
ANKVPALEHNNEVKGESLELIKYIDSHFEGPSLFPDVKSQKFLISCFSLFSYIDSFYKTATSSFKGDGSKAGVAF
DYIETALSKFEDGPFFLGQFSLVDIAYAPFIERIHPFLLEVKKYDFTLGRPKLATWIEEMNKNEAYTQTKSDPKD
LVQSYKERFMVLHLFKSLSSIGNFSTLILLTCFFCRLSFEHWGFSACPHHEKKN
>GSVIVG01025566001
MATTCVEEVLPPVLDSTSDQPPLFDGTTRLYISYSCPFAQRVWITRNYKGLQEQIKLVPLNLQNRPAWYKEKVYP
GNKVPALEHNNKVIGESLDLIKYVDSNFGGPSLCPDGPGKREFAEELISYTDTFIRIVYTSFKGDPAKEVGATFD
HLETALHKFDDGPFFLGQFSLVDIANIPFVEKFQIFLSEVWKYDITAGRPKLAAWIEELNKIGAYKQTKCDPKVV
VEAYTKLFLTQ
>GSVIVG01025559001
MATTCVEEVLPPVLDSTSDQPPLFDGTTRLYISYSCPFAQRVWITRNYKGLQEQIKLVPLNLQNRPAWYKEKVYP
ENKVPALDHNNKVIGESLDLIKYVDSNFGGPSLCPDGPGKREFAEELISYTDTFNRILYTSLKGDPTKEVGGPFD
HLETALHKFDDGPFFLGQFSLVDIAYIPFVERFQIFLSEVWKYDITVGRPKLAAWIEELNKIGAYKQTKCDPKEL
VEAYKKRFLTIYKLLTS
>GSVIVG01032639001
MATGSVREVLPPALDSTSEPPPLFDGTTRLYISYTCPYAQRVWITRNCKGLQEKIKLVPIDLQNRPAWYKEKVYP
ANKVPSLEHNNEVKGESLDLIKYIDSNFEGPSLFPDDPAKREFAEELLAYTDSFSKAVMTLLKGDGVDEAGAAFD
YIEMALSKFEDGPFFLGQFSLVDIAYAPFVERFVPFLREVKKYDAMAGRPKLEAWFEELSKNEGFQQTRREPKEL
VEAYKKRFAAHL
>GSVIVG01025083001
MAVTAEEVLPPSLDSTSEPPPLFDGTTRLYTSYICPYAQRVWIARNYKGLQDKIKLVPIDLGNRPAWYKEKVYPE
NKVPSLEHNNKVTGESLDLIKYIDSHFEGPSLYPDDPNKRQFAEELLSYTYTFNRAVIFSLKGDSQNEINAAFDY
LETALSKFNDGPFFLGQFSLVDIAFAPFIERFHPLLLDVKKCDITSGRPKLVSWIEEMNKIEAYKQTKRDPQELV
DALKKRFMAN
>GRMZM2G162486
MAAAAPASSVKEVLPSPLTSASEPPPLFDGTTRLYVAYLCPFAQRAWIARNYKGLQDKIKIVAIDLADRPAWYKE
KVYPENKVPSLEHDNQVKGESLDLVKYIDSNFEGPSLLPEDHAKQQFAEELLGYTDAFNKAFYSCLVDREDVSEE
AVAALDKIEDALGKFNDGPFFLGQFSLVDVAYVPFIERFQILYSNIKNYDVTKGRPNLQKFIEEVNKIDAYTQTK
LDPQFLLEQTKKRLGIA
>GRMZM2G043291

MNSLAFPSCPSALTPASTTAPASSPFPSPRIKIRCPRQEDAAHYHLRLRDRSSSQTVIVAMAAAAPASSVKEVLP
SPLTSASEPPPLFDGTTRLYVAYHCPYAQRAWIARNYKGLQDKIKIVAIDLADRPTWYKEKVYPENKVPSLEHNN
QVKGESLDLVKYIDSNFEGPSLLPEDPSKKQFAEELLAYTDAFNKALYFCIVSKEDVSEEAVAALDKIEDALGKF
NEGPFSLGQFSLVDIAYVPFIERFQIFYSNIKNYDITKGRPNLQKFIEEVDKIDAYTQTKQDPQFLLEQTKKRLG
IA
>GRMZM2G042639
MAAAAGPSSSVKETLPPALGSTSQPPPVFDGTTRLYICYFCPFAQRAWVTRNLKGLQDKMELVAIDLQDKPAWYK
DKVYAQGTQVPSLEHDSEVRGESLDLIRYIDSNFDGPALLPEDAAKRQFADELFASANAFTKALYSPLLSHAAVS
DEVVAALDKLEADLSKFDDGPFFLGQFSLVPRCILPGLKRGLIAQDIFFTLSNNNMLDPPFQADVAYVTILERVQ
IYYSHLRNYDIAQGRSNLQEFIDEMNKIEAYAQTKNDPLFLLDLAKNHLKIA
>GRMZM2G338131
MEALPPTLTSASEQPPLYDGTTRLYMSYVCPYAQRAWITRNYKGLQEKIKLVPMNMADKPGWYKEVYPNNQVPSL
EHNKRVIGESLDLIKYIDSNFDGPKLTITDDPERQRFAEELLGYSDAFNRAFLDALRSEGAMTTEAVAALDKIDS
ALLKFDDGPFFLGQLNLVDIAYAPFIEGFQIFFAGMKNCDITQGRVHIQKFIEEMNKIDACTQTKQDPQVLLALT
KKKFGI

mPGES2 class

>Aquca_034_00412.1
MRRAQQLILLNGRAAVSAGGSLASSTPIHTNNNWYNLQQAATLYSSSNRSDGSRSHWLPLSNSVAGRYGGGVFAG
AVSFSVLASDVYAKEPLPLDLRPKDVVLFQYEACPFCNKVKAFLDYYDIPYKVVEVNPISKKEIKWSEYKKVPIL
TVDGENLVDSSDIINKMSQKICPTHSIPDDGTEESKWRRWVDDHLVHMLSPNIYRNPSEALESFDYITSHGNFSF
TERVTAKYAGAVAMYFVSKKLKKKYNIVDERAALYEAAEIWVEALNGRNFLGGSKPNLADLAVFGVLRPIRYLRS
GKDMVEHTRIGEWYTRMESAVGESSRIAA
>Aly949105
MRRVTGLAARTISSSVAINPRLSQTMAITTISSSEPISRRFGGLPEISTPSFAGGVAGIVFFSAAAASSLGQEVH
AKEMAQKFNPKEVVLYQYEACPFCNKVKAFLDYNKIPYKIVEVNPISKKEIKWSDYKKVPILTVDGEQMVDSSVI
IDSLFQKMHPEISKSEDDEETKWRKWVDNHLVHLLSPNIYRNTSEALESFDYITTHGNFSFTERLVAKYAGATAM
YFVSKKLKKKYNITDERAALYDAAETWVDALKERPYLGGSKPNLADLAVFGVLRPIRYL
>AT5G42150.1
MRRVTGLAARTISSSVAINSRLTQSMAITTISSSEPISRRFGGLPEIKTPSFAGGVAGVVFFSAAAVSSLGQEVH
AKEMAQKFNPKEVVLYQYEACPFCNKVKAFLDYNKIPYKVVEVNPISKKEIKWSDYKKVPILTVDGEQMVDSSVI
IDSLFQKMHPEISKSEDDEETKWRKWVDNHLVHLLSPNIYRNTSEALESFEYITTHGNFSFTERLVAKYAGATAM
YFVSKKLKKKYNITDERAALYDAAETWVDALKERPYLGGSKPNLGDLAVFGVLRPIRYLRSGKDMVDNTRIGEWY
SRMENTVGEPSRIKE
>Bostr.22495s0002.1
MRRVTGLAARTISSSFAVNPRLIQSMAITTISSSEPFSRRFGGLPEISTPSFAGGVAGIVFFSAAAASSLGQEVH
AKEMAQKFNPKEVVLYQYEACPFCNKVKAFLDFNKIPYKIVEVNPISKKEIKWSDYKKVPILTVDGEQLVDSSAI
IDSLFQKMHPEISKSEDDEETKWRKWVDNHLVHLLSPNIYRNTTEALESFDYITTHGNFSFTERLVAKYAGATAM
YFVSKKLKKKYNITDERAALYDAAETWVDALKERPYLGGSKPNLADLAVFGVLRPIRYLRSGKDMVDNTRIGEWY
SRMENTVGEPSRIKE
>Bradi5g04550.1
MRSLRAAHTLASRSLLLSARALHGTACPGAAAAAGGRWGAPPPAPSSRVVPAGIAGAVSFSLTFATVAAAEVQAK
ERLPVDLLPQNVVLYQYQACPFCNKVRAFLDYHDIPYKVVEVNPLSKKEIKWSEYKKVPILTVDGEHLVDSSDII
NILQRKISPDDDVMSEEEAKWRRWVDEHLVHILSPNIYRTTSEALESFDYIAKHGNFSTVERFAAKYAGAAAMYM
VSKKLMKKYNITDARASLYEACNTWTEALNGRNFLGGSKPNLADLAVFGVLRPIRYLRSGKDMVEHTQIGEWYQR
MEDAVGEPSRIQDE
>Brara.B02462.1
MRRVTGLAARTISSSVAVHPRLAHTTAMMTTIPSSEPPSPRFGGLPTPSFAGGVAGIVFFSAAAASSLGQEVHAK
EMSHKFNPKEVVLYQYEACPFCNKVKAFLDFNKIPYKIVEVNPLFKKEIKWSDYKKVPILTVDGEQLVDSSVIID
SLFQRMHPGISKSEDDEETIWRKWVDNHLVHILSPNIYRSTSEALESFDYITTHGNFSFTERLVAKYAGATAMYF
VSKKLKKKYNITDERAALYDAAETWVDALNGRPFLGGSRPNLADLAVFGVLRPIRYLRSGKDMVDNTRIGEWFSR
MENTVGEPSGIKE
>Carubv10026807m
MRRVTGLAARTISSSVGIHPRLAQSMAITTISSSSEPITRRFGGLPEISTPSFAGGVAGIVFFSAAAASSLGQEV
HAKEMAQKFNPKEVVLYQYEACPFCNKVKAFLDFNKIPYKIVEVNPISKKEIKWSEYKKVPILTVDGEQMVDSSA
IIDSLFQKMHPEISKSEDDEETKWRKWVDNHLVHLLSPNIYRNTSEALESFDYITTHGNFSFTERLVAKYAGATA
MYFVSKKLKKKYNITDERAALYDAAETWVDALKERPYLGGSKPNLADLAVFGVLRPIRYLRSGKDMVDNTRIGEW
YSRMENTVGEPSRIEE
>Cagra.7999s0003.1

MRRVTGLAARTISSSVAIHPRLAQSMAITTISSSSEPITRRFGGLPEISTPSFAGGVAGIVFFSAAAASSLGQEV
HAKEMAQKFNPKEVVLYQYEACPFCNKVKAFLDFNKIPYKIVEVNPISKKEIKWSEYKKVPILTVDGEQMVDSSA
IIDSLFQKMHPEISKSEDDEETKWRKWVDNHLVHLLSPNIYRNTSEALESFDYITTHGNFSFTERLVAKYAGATA
MYFVSKKLKKKYNITDERAALYDAAETWVDALKERPYLGGSKPNLADLAVFGVLRPIRYLRSGKDMVDNTRIGEW
YSRMENTVGEPSRIKE
>evm.model.supercontig_3.395
MRRASTLASSIIPRTIAAAHDGGATTVHNRILQAAFFCSSSRISFCNRRRWLSPFLNSFSNGSARAVSLGVTGAL
ASVVVAASFSQAVYAKEPPAGELVPKDVVLYQYEACPFCNKVKAFLDYYDIPYKVVEVNPLSKKEIKWSDYKKVP
ILMVDGEQLVDSSDIIDKLNQKIFPEKSVDSALDENEERKWRRWVDNHLVHVLSPNIYRNTSEALESFDYITSNG
NFGFTEKITVKYAGAAAMYFVSKKLKKKYNITDERAALYEAAETWVDALNGREFLGGCKPNLADLAVFGVLRPIR
YLRSGRDMVEHTRIGEWYSRMESAVGESSKDQGLV
>Cre07.g319100.t1.1
MSSAVRRLAPALLFQAAQTRAAGLQPAASARFLAIGPTDQPHTNSNGGRPGILAAAAALLGAGAVTVGATVAADA
STPVAAASDPYARPAAARPLPDHVTLYQYEVCPYCCKVRAMLDYYKLPYTVIEVNPLTKGELAWSTYKKVPVVKL
GEEVVVDSSAIMSRLAVDVAAAGRDPMAAAAAAATAAPAKAAAAPAAAKAAGGGGWFGSSSSSSSSGSDAGAGAK
DQAAASSSSGGGGADAVSEEVRWRKWVDEKLVKILTANIYRNWDESVDTFKYITDQTGWSWGTREVARWAGAVMM
WQIGKRMPAKYGIEGDLRVALYDTANDFVDGALAGGKKRFAGGERPNLADLAAFGVIRAVRQTGAFRDLMANSRI
APWFAAMEEAVGGSARVATTGAKSG
>jgi|ChlNC64A_1|11521|gw1.25.104.1
VVLYQYEVCPFCCKVKAFLDYYKIPYRCVEVNPLTKAEIKWSDYKKVPVVVVDGEQLNDSSAIISRLAAEVRATQ
AAGGKQPSGSGGSGSSGAPAKKGWLGGLFGGGGGGSAGGGGGAPMPATAAEEEMWRRWVDDWLVKVITVNIYRNM
HESFQTFEYISEAGNFGWVSREAARVVGATLMWGISGKLRKKYGVEGDVREQLYKSADDWVGAVGGRAFLGGDAP
DLADLAVFGVIRAVVGTDTFNDLMQNSQIGGWYSRM
>Ciclev10008944m
MRRSTTVTSLILSSRTLATATINHRLLTTNSTSYSRWRCFSTGSAAAAATASLGVAGALASAAAIASLSAQSVYA
KEPLPTDLVPKEVVLYQYEACPFCNKVKAFLDYYDIPYKVVEVNPINKKEIKWSEYKKVPILMVDGEQLVDSSAI
IDQLDQKLTPKRKADSPSGDDEEKKWRGWVDNHLVHLLSPNIYRNTSEALESFDYITSSGNFSFTEKLTAKYAGA
AAMYFVSKKLKKKYNITDERAALYEAAETWVDALNGREFLGGSKPNLADLAVFGVLRPIRYLRSGRDMVEHTRIG
EWYTRMERVVGESSRIKAS
>Ciclev10001861m
MRRLNRLATLSRTVSSGATSAATQHRHYQLAQPAALFSTFSSCQSQGLAQKIVDRLGPSFAGGVAGFSAAAPSLA
QDAKPQAKEQPRSEKFGDVVLYQYEACPFCNKVKAFLDYYRIPYKVVEVNPISKKEIKWSDYKKVPILKVDGEQM
VDSSDIMDKLFQRIHLDNASSQSDEEKKWRAWVDNHLVHVLSPNIYRTTSEALESFDYISTQGNFSFTEKLVAKY
VGAAAMYFVSKKLKKRHNITDERASLYEAAEIWVDALNGRHYLGGSRPNLADLAVFGVLRPIRHLKSGRDMVEHT
RIGDWYTRMESSVGSSRIKE
>orange1.1g020964m
MRRSTTVTSLILSSRTLATATINHRLLTTNSTSYSRWRCFSTGSAAAAATASLGVAGALASAAAIASLSAQSVYA
KEPLPTDLVPKEVVLYQYEACPFCNKVKAFLDYYDIPYKVVEVNPINKKEIKWSEYKKVPILMVDGEQLVDSSAI
IDQLDQKLTPKRKADSPSGDDEEKKWRGWVDNHLVHLLSPNIYRNTSEALESFDYITSSGNFSFTEKLTAKYAGA
AAMYFVSKKLKKKYNITDERAALYEAAETWVDALNGREFLGGSKPNLADLAVFGVLRPIRYLRSGRDMVEHTRIG
EWYTRMERVVGESSRIKAS
>orange1.1g040684m
AGFSAAAPSLAQDAKPQAKEQPRSQKFSDVVLYQYEACPFCNKVKAFLDYYRIPYKVVEVNPISKKEIKWSDYKK
VPILKVDGEQMVDSSDIMDKLFQRIHLDNASSQSDEEKKWRAWVDNHLVHVLSPNIYRTTSEALESFDYITTQGN
FSFTEKLVAKYAGAAAMYFVSKKLKKRHNITDERVSLYEAAEIWVDALNGRHYLGGSKPNLADLAVFGVLRPIRH
LKSGRDMVEHTRIGDWYTRMESSVGSSRIKE
>14039
PNEIILYQYEVCPFCCKVKAFLDYHKLPYRTVEVSPLTKKQLKWSEYRKVPVALLDGEIVTDSTAIITRLAAEIA
GQEQQEQQQQQKSHGEEEWRRWVDERLVRLLTVNIYRNMRESYQTFDYITASSCNFGFFEREAARVVGAVMMWGI
SGRLKKKYGIEGDVREELYQAANKWTEALGDQRFHGGSKPDLADLSVFGVVRSITCTDTFMDLMHTTRIGTWYEH
MMDAVGDSTRLSTS
>Cucsa.393900.1
MRKINGVHLLSRLVSADATTTTTYRQLLRQSAVLRTCTGSNIRCFSQVANPFGSYDPSSVRKVAGNARFVSVASS
SLAEDLVNGSPRPSFVPKDVVLYQYEACPFCNKVKAFLDYYNVPYKVVEVNPIFKKEIKWSEYKKVPILMVDGVQ
MVDSTDIIHNLYQRIHPENSASNLEEEKKWLGWVDNHLVHVLSPNIYRNYKEALESFNYITTHGNFSFAQRIIAK
YGGATAMYFVSKKLKEKHNITDERKALYGAAETWVDALKDRQFLGGANPNLADLAVFGVLRPIRHLQSGKDMVEH
TRVGEWYTRMEKAVGKSARING
>Cucsa.111600.1
MRRSSSIASSFLSRSISTSHGSSAISTHRLLQAALFSSTSHNHRRWFSSLLDSFSGRSTRAVSLGVVGAFASIAA
AVSMSQEVYAEERLRQDLIPKEVVLYQYEACPFCNKVKAFLDYYDIPYKVVEVNPLSKKEIKWSDYKKVPILVVD
GEQLVDSSAIIDQLSHRVLPDKNVSSVSEDDEETKWRRWVDNHLVHMLSPNIYRNTSEALESFDYITSNGNFGFA

EKISVKYAGAAAMYFVSKKLKKKYNITDERAALYEAAETWVDALAGRDFLGGSKPNLADLAVFGVLRPIRYLRSG
KDMVEHTRIGEWYTRMESAVGDSSRIRS
>Eucgr.B01000.1
MRRASSTLAPSFLSRALASSATASGHGHRHRLLQAALYGSATGGGGGYSRRAGRRWFSRLLDSPRAASLGIAGAV
FAASAASAALSGEVLAKEPPSPEVVPKEVVLYQYEACPFCNKVKAFLDYYDIPYKVVEVNPISKKEIKWSDYKKV
PILMVDGEQLVNSSDIIDKLGRKILPERSAKPATEDADDEERKWRRWVDNHLVHVLSPNIYRNTSEALESFDYIT
SNGNFSFTEKITVKYAGAAAMYFVSKNLKKKYNITDERAALYDSVETWVDALNGREFLGGSKPNLADLAVFGVLR
PIRYLRSGKDMVEHTRIGEWYSRMESSVGESSRIKA
>Eucgr.H00953.1
MTTRSIRGAAALTRAAAAVGAASAGASQHRLLGAALRNPRAGTSSPWLSEVLTGRRWPSIPSVANGAAGARLVSS
AAAVATSAAREEGSRGARFLPGDVVLYQYEACPFCNKVKAFLDYYKIPYKVVEVNPINKKEIKWSDYKKVPILMV
DGKQMNDSSAIIDQLFEKIHPDELAENEEERKWRGWVDNHLVHVLSPNIYRTPSEALESFDYITTHGNFSFMERL
IAKYSGAAAMYMVSKKLKKRHNITDERAALYEAAETWVDALKGRPFCGGAEPNLADLAVFGVLRPIRHLQSGKDM
VEHTRIGEWYDRMQHAVGNSTRAMT
>Eucgr.H00955.1
MTTRSIRGAAALTRAAAAGGAAAASASQHHRLLRAAVRDLRAGTSSPWLSEVLSGRRWPSIPSASNGATGARLVS
SAAAAAAATSPAREEGSQGARFLPGDVVLYQYEACPFCNKVKAFLDYYKIPYKVVEVNPISKKEIKWSDYKKVPI
LMVDGNPMNDSSAIIDQLFGKIHPDDLAENEEERKWRGWVDNHLVHVLSPNIYRTPSEALESFDYITTHGNFSFM
ERLIAKYSGAAAMYMVSKKLKKRHNITDERAALYEAAETWVDALKGRPFCGGAEPNLADLAVFGVLRPIRHLQSG
KDMVEHTRIGEWYDRMQHAVGNSTRAMTQL
>Thhalv10027850m
MRRVTGLAARTISSSVAIHPRLAQTTVMMTTISSSEPPSPRFGGLPTPSIAGGVAGIVFFSAAAASSLCQEVHAK
ETSGLAQKFNPKEVVLYQYEACPFCNKVKAFLDFNKIPYKIVEVNPMSKKEIKWSDYKKVPILTIDGEQLVDSSA
IIDSLFQRMHPEILKSNDDEETKWRKWVDNHLVHLLSPNIYRNTSEALESFDYITTHGNFSFTERLVAKYAGATA
MYFVSKKLKKKYNITDERAALYEAAETWVDALKERPYLGGSKPNLADLAVFGVLRPIRYLRSGKDMVDNTRIGEW
YSRMENTVGEPSRIKE
>mrna09372.1-v1.0-hybrid
MRRASTLASFPILSRSIATLHGCATATSSSASHRLLQVALYTTAGSAGSHARRPWSESFSGRKLAFGVAGTLASV
AVATSLAQEVHAKEPPPAELVPKEVVLYQYEACPFCNKVKAFLDYYDIPYKIVEVNPLSKKEIKFSEYKKVPILM
VDGEQLVDSSVIIDTLTEKILPDRVGERVAVSTSNADDEEKKWRKWVDSHLVHMLSPNIYRNTSEALESFDYITS
NGNFSYTEKITVKYAGAAAMYFVSKKLKKKYNITDERASLYEAAETWVNALDGREFLGGSKPNLADLAVFGVLRP
IRYLRSGKDMVEHTRIGEWYSRMERAVGEPSRIKE
>mrna08557.1-v1.0-hybrid
MRRINGLAFLGRTAAGLGGATRVAGATPQRLVQAATISSCSNSNPLWFSQSSGDRFGLSKPSTAAGGAIWFSTTA
AQEMHSHVSGDKFAPNDVVLYQYEACPFCNKVRAFLDYKKIPYKVMEVNPMSKKEIKWSDYKKVPILKVDDEQMV
DSSDIIDKLSQRINPENNVVDNEEEKKWRHWVDNHLVHVLSPNIYRTPSEALESFDYITSHGNFSLSERLVAKYG
GAAAMYFVSKKLKKRHNITDARAALYGAAETWVDALKGRQFLGGSTPNLADLAVFGVLRPIRHLKSGKEMVENTR
IGEWYSRMESAVGESARINA
>Glyma.07G102200.1
MRRVPSILFRTLTSPRVATSFSVPNRLLQAALYRSAAARSHRRRFSSLLGPLAAASLGVAGALVSQEVLAKEPPP
PEALPNDVVLYQFEACPFCNKVKAFLDYYDIPYKIVEVNPLSKKEIKWSEYQKVPILMVDGEQLNDSSVIIDKLG
HKILSKKIVDSTSEDEETKWRRWVDNHLVHVLSPNIYRNTSEALESFEYITSNGNFSYLEKFSVKYAGAAAMYFV
SKKLKKKYNITDERAALYEAAETWVDALDGREFLGGSKPNLADLAVFGVLKPIRYLRSGKDMVEHTRIGEWYARM
ESAVGEPSRIKA
>Glyma.09G177000.1
MRRVPSILFRTLAAPRAATSFAVPNRLLQAALYGSATAASRSHRRRFSSLLGPFAAASLGVAGALISQEVLAKEP
PPPEALPNDVVLYQFEACPFCNKVKAFLDYYDIPYKVVEVNPLSKKEIKWSEYQKVPILMVDGEQLNDSSVIIDK
LGHKILSKIIVDSTSEDEETKWRRWVDNHLVHVLSPNIYRNTSEALESFEYITSNGNFSYLEKFSVKYVGAAAMY
FVSKKLKKKYNITDERAALYEAAETWVDALDGREFLGGSKPNLADLAVFGVLKPIRYLRSGKDMVEHTRIGEWYA
RMESAVGEPSRIKA
>Glyma.04G023200.1
MRGVKRAVFVGRALRSNSNTAVYGVGATLHRDRMAPHGCLTPSNPNPSVSVTGFRYLSASAAATSLAQDAQTKAF
KPKELVLFQYQACPFCNKVAAFLDYYDIPFKVVEVNPINKKEIKWSDYKKVPILTVDGEQIVDSSDIIDKLIKRI
HPDYDLNAEEEKKWRQWVDNHLVHVLSPNIYRNVPEALESFDYITTQGNFSFSERLVAKYGGAAAMYFVSKKLKK
KHNITDERAALYGAAEQWVDALKGRKFLGGLDPNLADLAVFGVLRPIRHLKSGRDMLEHTRIGKWFSQMDRVVGQ
SSRVSEQS
>Gorai.005G133000.2
MRRASFTSSILSRTLSTVHDGGTVNLHPRLLRVALFSSSRTDNSNSRRHWFSPLLNSFSKTSGQAVSLGLVGVVA
SVASAASVYAKEPPPAEIIPKDVVLYQYEACPFCNKVKAFLDYYDIPYKVVEVNPISKKEIKWSDYKKVPILMVD
GQQLVDSSAIIDQLSEKILPGKAIISVADEDEETKWRRWVDNHLVHVLSPNIYRNTSEALESFDYITSNGNFSFT

EKITVKYAGAAAMYFVSKNLKKKYNITDERAALYEATETWVDALNGRNFLGGSKPNLADLAVFGVLRPIRYLRSG
RDMVEHTRIGEWYSRMEKVVGESSRIKA
>Gorai.009G193000.2
MRRVNGLATVGRALTCGGAVEKRLAQAALMSTCSSSSLNSHIFSQKLADRLGLSRPSLVRRVAGTMLFSVAASSL
AQDALAKEKPRSEKFLPKEVVLYQYEACPFCNKVKAFLDYYNIPYKIVEVNPISKKEIKWSDYKKVPILKVDGEQ
MVDSSDIIDKLFHRINPDSSIPDGEEKKWREWVDNHLVHVLSPNIYRSTSEALESFDYITTHGNFSFTERLVAKY
AGAAAMYFVSKKLKKKHNITDERAALYEAAETWVDALDGRHYLGGSKPNLADLAVFGVLRPIRYLTSGKDMVEHT
RIGEWYGRMENAVGESARIKE
>Lus10013568
MRRAHGLTTYGRAIASGSGFQAAVAPTPTPLFRSAMFSTSSVSGPQWPSRRLADYFGLKGTSVAAGVVGTMFFSV
AASSMAQEVHAKELPKFIPKDVVLYQYEACPFCNKVKAFLDYYSIPYTVVEVNPISKKEIKWSEYKKVPILTVDG
EHMVDSSEIVSQLFKKIHPEGSNVEVEEETKWRKWVDGHLVHVLSPNIYRSTSEALESFDYITSHGNFSFTERLV
AKYAGATAMYFVAKKLKKKYNITDERAALYESAETWVDALKGRDYLGGSRPNLADLAVFGVLRPIRYLKSGKDMV
EHTRIGEWYSRMEGAVGEPSRINA
>Lus10017275
MRRAHGLNTYGRAIAAGSGFQAAVAPTPNPLFRSAMLSTSSVSGPQWPSRRLADYFGLKGTSVAAGVVGTMFFSV
AASSMAQEVHAKELPKFIPKDVVLYQYEACPFCNKVKAFLDYYSIPYTVVEVNPISKKEIKWSEYKKVPILTVDG
EHMVESSEIVSELFKKIHPEGSNVEAEEETKWRKWVDGHLVHVLSPNIYRSTSEALESFDYITSNGNFSFTERLV
AKYAGATAMYFVAKKLKKKYNITDERAALYESAETWVDALKGRDYLGGSRPNLADLAVFGVLRPIRYLKSGKDMV
EHTRIGEWYSRMEDAVGEPSRINA
>MDP0000130760
MRRISGIALLTRAAVSMGGATTATSATQHRLVQAAMLSTCSNSNPLWFTQRLGDRFGLSRPSTAAPCAGGHVCFS
TNAAAXLAQEPHAHEKFLSKDVELYQYEACPFCNKVRAFXDYYNIPYKVVEVNPISKKEINWSDYKKVPILKVDG
EQMVDSSDIIDKLYLRINENPVLNGEEEMKWRGWVDNHLVHVLSPNIYRTVSEALESFDYITSHGNFSLYERMVA
KYTGAAAMYFVSKKLKKRHNITDERASLYGAAETWVDALNGRQFLGGSNPNLADLAVFGVLRPIRHLKSGKDMVE
NTRIGEWYARMESAVGESARVKA
>MDP0000127757
MRRISGIALLTRAAVSMGGATTATSATQHRLVQAAMLSTCSNSNPLWFTQRLGDRFGLSRPSTAAPCAGGHVCFS
TNAAAXLAQEPHAHEKFLSKDVELYQYEACPFCNKVRAFXDYYNIPYKVVEVNPISKKEINWSDYKKVPILKVDG
EQMVDSSDIIDKLYLRINENPVLNGEEEMKWRGWVDNHLVHVLSPNIYRTVSEALESFDYITSHGNFSLYERMVA
KYTGAAAMYFVSKKLKKRHNITDERASLYGAAETWVDALNGRQFLGGSNPNLADLAVFGVLRPIRHLKSGKDMVE
NTRIGEWYARMENAVGESARVKA
>MDP0000138039
MRRASTLASLPLLSRSLSTAHGGGAVTSAPTTYGLLQVALYGTTSTGSQAPRRWLDSFSGGSGRKLAIGVAGTLA
SVAVATSLAPEVYAKEPPPAALVPKEVVLYQYEACPFCNKVKAFLDYHDIPYKVVEVNPLSKKEIKWSDYKKVPI
LMVDGEQLVDSSAIIDQLNSKIVPERAAASSSDDDEEKKWRQSCVLLTLWSLSFEAVGVAYLELWWVDNHLVHML
SPNIYRNTSEALESFDYITSNGNFSYTEKISVKYAGAAAMYFVSKKLKKKYNITDERASLYEAAETWVDALNGRD
FLGGSKPNLADLAVFGVLRPIRYLRSGKDMVEHTRIGEWYSRMERAVGEXARIKE
>MDP0000238668
MRRASTLASLPRLSXSLSTAHGGAASTSAPXTYGLLQVALYGTTSTGSQARRRWFDSFSGGSGRKLAIGVAGTLA
SVAVATSLAPEVYAKEPPPAALVPKEVVLYQYEACPFCNKVKAFLDYHDIPYKVVEVNPLSKKEIKWSDYKKVPI
LMVDGEQLVDSSAIIDQLNSKIVPERAAASSPDDDEEKKWRQYVIFSSDSFLCLYSYIHVWIKWEEWYXYTVSRS
REGCRLEPLNHNLNGSTYLRWVDNHLVHMLSPNIYRNTSEALESFDYITSNGNFSYTEKISVKYAGAAAMYFVSK
KLKKKYNITDERASLYEAAETWVDALNGRDFLGGSKPNLADLAVFGVLRPIRYLRSGKDMVEHTRIGEWYSRMEQ
AVGEPARIKA
>cassava4.1_011619m
MRRASTLASSVFSRTLATTYDGGATTTNRRLLQAALYCTGGAGGSYNHNRWFYRLSNSFSARAASVGVAGALVSV
AATTFLAQEAYAKEPPPQELVPKDVVLYQYEACPFCNKVKAFLDYYDIPYKVVEVNPISKKEIKWSDYKKVPILM
VDGEQLVDSSVIIDKLRSKISKKKIDLASDGGGEEEKWRRWVDDHLVHVLSPNIYRNTSEALESFDYITSHGNFS
FTEKFTVKYAGAAAMYFVSKKLKKKYNITDERAALYEAAETWVDALNGREFLGGSKPNLADLAVFGVLRPIRYLR
SGRDMVEHTRIGEWYTRMEHAVGESSRIRA
>cassava4.1_029365m
MRRVNGLVTVIRTVAAASAAGTTARHRVFQAAMMSTCASTNSSQRIADRFGISSPSVAHGVAGTMFFSVAASSLE
QEVHAKEAPPAEKFLPKDVVLYQYEACPFCNKVKAFLDYYSIPYKIVEVNPISKKEIKWSNYKKVPILTVDGEQM
VDSSDIINKLFQRIIQMIQSQVMMKKANGRVHVLSPNIYRTTSEALESFDYITTHGNFSFTERLVAKYAGAAAMY
FVSKKLKKRHNITDERAALYEAAETWVNALKGREYLGGSKPNLADLAVFGVLRPIRHLKSGKDMVEHTHIGEWYS
RMEQAVGEPARIKA
>Medtr3g114070.1
MRGLSGTISVARALRANSTAASTFLRPPRAASYAVTYWTTSNPQNLVDRSNLSTPSVIRSDVIGFRHYGSAAAAS
PAEQDLKPRISEQNSFNPKEVVLFQYEACPFCNKVKAFLDYHGIQYKVVEVNPTNKKEINWSHYKKVPIVIVDGE
QLVDSSDIIDKLVKRIHPDYDLNADEEKKWREWVDNHLVHVLSPNIYRTVSEALESFDYITTKGNFSLYERLVAK

YGGAAAMYFVSKKLKKRHNITDERQALYGAAEQWVDALKGRKFLGDLEPNLADLAVFGVLRPIRHLKSGRDMVEH
TRIGNWFSEMENAVGQASRMSA
>Medtr6g069420.1
MRRASSVLLYRTLAAARDGSATSIALPNRFLQSTFYGTTAGSSPSRRRLFSSAIAVAAGTSLGVTGALFAASSLS
QEVLAKEPPPSDALPNEVVLYQYEACPFCNKVKAFLDYYDIPYKVVEVNPLSKKEIKWSEYKKVPIIMIDGEQLN
DSSAIIDKLGEKILSKKKADSTSEDEVDEETKWRRWVDNHLVHVLSPNIYRNTSEALESFDYITSNGNFSYMEKI
SVKYAGAAAMYFVSKKLKKKYNITDERAALYEAAETWVDALNGREFLGGSKPNFADLAVFGVLRPIRYLRSGKDM
VDHTRIGEWYTRMESVVGEPSRIKA
>5265
VTLYQYDVCPFCNKVKAQLDFLGIAYDVVEVNPLTKSELGFSKEYRKVPIVIVDGEQINDSAVIMREMETRMKKA
GLRGRGARPRPGSAAAKKEDEWFAWVDSRFVHVVTPNIYRTWEEAQRSFDYITERGNFNWFMRQAIALSGAASMY
VISHRVLKKRHGIEDERAALYEALDDWMQNAVGPKNAFCGGSEPNLADLAVFGVLRAVKTFDTFSDAMENTNAKP
WYERMRVEVGEATR
>88190
KVTLYQYDVCPFCNKVKAYLDYRGIPYDVVEVNPLTKSEIKFSKEYRKVPIVMVDDEQLNDSAHIIATLDERLGK
IAPPGFIGGKAMTEKEEKWAKWVDAWFVHVITPNIYRTWAEAFKSFDYITERGKFGWVERQSVRLSGAVSMYLIS
QNVLKKRHGIEDERLELYKALEDWMENGVGGAAFCGGDVPNVADISVFGVLRAVKTFETFDDALANVKSVEGWYR
RMEKEVGEATRTDGIAN
>Migut.N03175.1
MRRVSASGLAAISRAVGGVAVAPSAAGNHAQHHHRLFQAALFSTNSNSSCESRSHWFSNNRIAADQVARAVAGTM
FFSVAATTLVEEVNAKEPVQSKFRPKDVVLYQYEACPFCNKVKAFLDYYDIPYKIVEVNPMNKKEIKFSEYQKVP
ILMVDGEQMVDSSDIIDKLDKKIHPEVSKDSVGQDGEEKKWRGWVDNHLVHILSPNIYRSPSEALESFDYITSHG
NFSFTERITAKYAGAAAMYFVSKRLKKKYNITDARASLYEAAETWVDALKGQDFLGGGKPNLADLAVYGVLRPIR
YLRSGKDMVEHTRIGDWYSRMENAVGVSSKI
>Migut.C00863.1
MRRINGLAAICRAVEGVAAAPSVAGNLVRHHRLVQAALSTTTNNGSLSRSNWLSCNRTAAGQSTSAAAHAVGGVI
LFSVAASAIGVEAHAKEPVQSEFRPKDVVLYQYEACPFCNKVKAFLDYYDIPYKVVEVNPLSKKEIKWSDYKKVP
ILMVDGEQMVDSSDIIDKLAKKIHPDRAVGSVADNEEKKWRGWVDNHLVHMLSPNIYRNTSEALESFDYITSHGN
FSFAERIAAKYAGAAAMYFVSKKLKKKYNITDERASLYEAAETWVDALDGRDFLGGTKPNLADLAVYGVLRPIRY
LKSGRDMVEQTRIGDWYARMEAAAGPSSRIQA
>LOC_Os04g17050.1
MRSIRAAQALASRSLLLSSRALHGDAASTAAAAAGGGRLGVQPSPPSQASSSSSSRAMPAGIAGAVSFSLTFATM
AAAEAKERPPMDLLPQNVVLYQYQACPFCNKVRAFLDYHDIPYKVVEVNPLSKKEIKWSEYKKVPILMVDGEQLV
DSSDIINILQQRVRPDDKATNEEEEKWRRWVDEHLVHVLSPNIYRTTSEALESFDYISKHGNFSFTERFAVKYAG
AAAMYMVSKKLKKKYNITDARASLYDAANTWMEALDGRDFLGGSKPNLADLAVFGVLRPIRYLTAGKDMVEHTQI
GDWYQRMEDAIGEPSRIQE
>29981 (primary)
MTHRASTAATTARASTRTRAGTAATATAAAAAAAALAATTAGREASAEAPRTSPADAALGGARVTLYQYDVCPFC
NKVKAFLDYHGTPYDVVEVNPMTKGELGWVEDGWKKVPIVTVGDEKLNDSSAIIAELTKRFDASGSSANAGAWFG
AKKTKAYLEREATWTKWVDERFVHVLTPNIYRTWAEAVKSFDYITKRGNFGYFERESARWVGAASMYVIAHRVLK
KRHGIEDERADLYAECDKFVDEAVGSRKFCGGDAPNNADLCVFGVLRAVKTFETFADVMANTSIRPWYERMEKAV
GPATRTDGLEN
>Pavir.Ea01564.1
MRSLRAARTFRSLLSARPLHGAASPAAAAAASAAAGSRWCAPLVPSPRLPPPSPRAMPAGVAGAVSFSLTFATVA
AAEAKAKERPPTDLLPRNVVLYQYQACPFCNKVRAFLDYHDIPYKVVEVNPLSKKEIKWSDYKKVPILTVDGEQL
VDSSDIINILQHRIRPDDELTNEEEAKWRRWVDEHLVHMLSPNIYRTTSEALESFDYIAQHGNFSFTERFAVKYA
GAAAMYMVSKKLKKKYNITDERASLYDAANTWVEALNGRDFLGGSKPNLADLAVFGVLRPIRYLRAGKDMVENTQ
IGEWYQRMEDAVGEPSRIQE
>Phvul.009G009900.1
MRGLNRGILLSRALRSNFTAVSGATATLHPVAPHGTTTLRSSNSSVTGFRYVSFSASASATCLAQEAQIKAFEPK
ELLLFQYQACPFCNKVAAFLDYYNIPFKVVEVNPINKKEIKWSDYKKVPILSVDGEQMVDSSDIIDKLIKRIHPD
YDLNAEEEKKWREWVDNHLVHVLSPNIYRNVPEALESFDYITTQGNFSFSERLVAKYGGAAAMYFVSKKLKKKHN
ITDERAALYGAAEQWVDALNGRKFLGGLDPNLADLAVFGVLRPIRDLKSGRDMVEHTRIGKWFSEMDRAVGQSSR
VWERS
>Phvul.004G150900.1
MAQSRKAKTLCAWIHQTHINPSNSQRPKPSSHSESQRSESILRRHAMRRVPSILFRTLAATRATRATSYVVHNRL
LHGSAATAAAHSHSRRFSYLLAPFAAASIGVAGALVSQEVLAKEPPPPEALPRDVVLYQFEACPFCNKVKAFLDY
HDIPYKVVEVNPLSKKEIKWSEYQKVPILVVDGEQLNDSSAIIDQLGLKILSKKIADSTSEDEETKWRRWVDNHL
VHVLSPNIYRNTSEALESFEYITSNGNFSYIEKISVKFAGAAAMYFVSKKLKKKYNITDERAALYEAAETWVEAL
NGREFLGGSKPNLADLAVFGVLKPIRYLRSGKDMVEHTRIGEWYARMESAVGEPSKIKA
>Phpat.022G051000.1

MAAVRVMAQRIARARRSGLAAAAQQYGRVIGTSEGDDAVLRVGTRAAGMEKAMAVVEEEQQQGARNWRRWSSGSW
IAAGAASLSFAASTMTVAYGKERVTDRFSPKEVVLYQYDACPFCNKVKAFLDYHDIAYKVVEVNPVGKKEIKWSD
YKKVPILVVDGEALNDSTAIITELTRRIQGGNAKDLAQKIGSDEEEKWRSWVDEHLVHLLSPNIYRTPREALQAF
DYLTTNGNFSSIERATGKYVGATAMYFIGKRLKKRHNIIDARASLYEAAEEWVAALNGRSFMGGSKPNLADLAVF
GVLRPIRHLDTGKDLLASTQIGEWYMRMEDAVGETARLPEEPLMGTIDSSMKV
>Phpat.019G052100.1
MASLPCGRRPCPLKLNEEWELLVRQRAERMLGVDCLRFHHCLARCTEIHCCHCGEKHIFVIGARSIVVISGRIGT
EAAGTMMSVRAVAHRCARARSGAQAVQRCAGVVWTSEGHDDVRRFGMQAAGVAKGNSSGLDRRTWSEEDARGRSW
NARWIGAGSFSLSFATSTIGVAYGKERVADRFAPKDVVLYQYETCPFCNKVKAFLDYHDIAYRVVEVNPMGKKEI
KWSDYQKVPILVVDGEALKDSTAIITELTRRIEGGNANAPALKPESDEEERWRRWVDEHLVHLLSPNIYRTPRES
LQALDYLTTSGNFSMMERATGKYFGAAAMYIIGKRLKKRHNIVDERISLYDAVEEWVKALDGRQFMGGSKPNLAD
LAVFGVLRPIKSLDTGRDMLASTKIQEWYSRMEDTVGATARLQEESLMGSIDNSVKV
>POPTR_0005s26400.1
MRRVNHLATLSRAFSGAASHQRLVQGAAMISTYAASNSQCFASRIAESFRISNPPVARGVTGTMFFSVAASSLAQ
EAQAKEAPPVEKLMPKDVVLYQYEACPFCNKVKAFLDYYNIPYKVVEVNPINKKEIKWSDYKKVPILKIDGEQMV
DSSDIVDKLFQRIHPDNSVTDSDEERQWRGWVDNHLVHVLSPNIYRSVSEALESFDYITTHGNFSFTERLVAKYA
GATAMYFVSKKLKKRHNITDERAALYGAAETWVDALKGRQYLGGLKPNLADLAVFGVLRPIRYLKSGKDMVEHTR
IGEWYSRMENAVGEPSRIKA
>POPTR_0012s04410.1 (eugene3.00120387)
MRRASTLASSVLSRTLTPTLHEGGALSTINHRFLLVALYSTTSNTGSSHSRRIFNPFSSSLGVAGALVSAAAAAS
LSQDVLAKEPPRAELVPKEVVLYQYEACPFCNKVKAYLDYYDIPYKVVEVNPISKKEIKWSDYKKVPILTVDGEQ
LVDSSAIIDKLRNKIHGKEIVESASDKDDDEEIKWRRWVDNHLVHVLSPNIYRNTSEALESFDYITSNGNFSFTE
KITVKYAGAAAMYFVSKKLKKKYNITDERAALYEAAETWVDALNGREFLGGSKPNLADLAVFGVLRPIRYLRSGR
DMVEQTRIGDWYTRMENAVGESARIKA
>POPTR_0015s04620.1|POPTR_0015s04610.1
MRRASTLASSVLSRTLSSTLHDCSGLSTTSAATINHRFLHAALFSTTTSTGSSSTRRIFNPFSTYLGVAGALVSA
AAAASLSQEVLAKEPPPAELVPKEVVLYQYEACPFCNKVKAYLDYYDIPYKVVEVNPISKKEIKWSDYKKVPILL
VDGEQLVDSSAIIDKLGNKIHGKEIVDSASDKDDDEEKKWRRWVDNHLVHVLSPNIYRNTSEALESFDYITSNGN
FSFTERITVKYAGAAAMYFVSKNLKKKYNITDERAALYEAVETWVDALNGREFLGGSKPNLADLAVFGVLRPIRY
LRSGRDMVEQTRIGDWYTRMENAVGESSRMKA
>ppa008397m
MRRASTLASFNVLSRSLATVHGGAVATSASPSHSLLQVALYGTSSTTGPHVRRRWFDSFSGGPAGKLAIGVAGTL
ASVAVASSLTQEVYAKEPPPAELVPKDVVLYQYEACPFCNKVKAFLDYHDIPYKIVEVNPLSKKEIKWSDYKKVP
ILMVDGEQLVDSSAIIDKLNDKIVPERVAAPSSNDDEEKKWRQWVDNHLVHMLSPNIYRNTSEALESFDYITSNG
NFSYTEKFTVKYAGAAAMYFVSKKLKKKYNITDERASLYEAAETWVNALNGRDFLGGSKPNMADLAVFGVLRPIR
YLRSGKDMVEHTRIGEWYSRMERAVGESARIKA
>ppa008706m
MRRVNGAALLSRAVVCMGSSTAVGGTTQQRLVQAAMLSTCSNSNPLWFTQRLGDHFGLSKPYVACGAGGHVCFSS
NATPLAQEVHAQDKFLPKDVELYQYEACPFCNKVRAFLDYYKIPYKVMEVNPISKKEIKWSDYKKVPILKVDGEQ
MVDSSVIIDKLFQRINPENNVVNGEEEKKWRGWVDNHLVHVLSPNIYRTASEALESFDYITSHGNFSLYERIVAK
YTGAAAMYFVSKKLKKKHNITDERAALYEAAETWVDALKGRQFLGGSNPNLADLAAFGVLRPIRHLKSGKDMVEN
TRIGEWYTRMESSVGESARIEA
>30170.m014258
MRRATGFVTLTRTLAGGVSSAQQRVAPAAMMSTCSSSNSSQRIFDRFGISNPFVARGIAGIMFFSAAAASPFAQE
VHAKEPPPREKFLPNDVILYQYAACPFCNKVKAFLDYYNIPYKIVEVNPISKKEIKWSDYKKVPILTVDGEQMVD
SSDIINKLFERIHTGNSIPDSDEESKWRGWVDNHLVHVLSPNIYRNTTEALESFDYITTHGNFSFTERLTAKYAG
AVAMYFVSKKLKKKYNIIDERAALYDAAETWVDALNGRDFLGGSKPNLADLAVFGVLRPIRYLKSGKDMVEHTRI
GEWYSRMEREVGERNGVEA
>Smo103322
MPKEVILYQYEACPFCNKVKAFLDYYDVPYRIVEVNPLGKKEIAWSEYKKVPILMVDGEQMNDSTGKVLVDLCCS
SSLLIVLRVLSLEIISTLDGKLNPKRSPALTAAAKEEEEKWRKWVDGHLVHLLSPNIYRSTKEALQAFDYITREG
NFTAWERATAKYAGAAAMYFISKRLKKRHNITDERAALYEAANTWVEALDNRNVFSGGSKPNLADLAVFGVLRPI
RNLDSGNDMIANSKIGAWYSRMEQAVGNSARVLAQEAFKTQPVEMST
>Si002281m
MRSLRAAQTLAFRSLLSARALHGAASPAAAAAAGARWSAPLVPPPRSPPPSSRAVPAGVAGAVSFSLTFATVAAA
EAKAKERPPADLLPQNVVLYQYQACPFCNKVRAFLDYHDIPYKVVEVNPLSKKEIKWSDYKKVPILTVDGEQLVD
SSDIINILQGRIRPDDELTNEDEAKWRRWVDEHLVHVLSPNIYRTTSEALESFDYIAKHGNFSFTERFAVKYAGA
AAMYMVSKKLKKKYNITDERASLYDAANTWVEALNGRDFLGGSKPNLADLAVFGVLRPIRYLRAGKDMVENTQIG
EWYQRMEDAVGEPSRIEE
>Solyc04g081740.2.1

MTSWAEIFPEPTAPGAQFKLLTWQSLKPSLNPKLSCLQIATKAHRIKVCIFQIFGRGKRIAGMRRVSRIAALYRA
VDGAAAMEVPQHRMSTAAQFSTSSNKSSTRSNWLFNNLLTDLSARTSAHAVAGTMLFSVAATTLTEEVHAKEVVP
PELRPKDLVLYQYEACPFCNKVKAFLDYYDLPYKIIEVNPISKKELKWSDYKKVPVVLVDGEQMVNSSDIIDKLY
EKVRSGDSTFDADEESKWRKWVDDHLVHMLSPNIYRNTSEALESFDYITSHGNFSFTERITAKYAGAAAMYFVSK
KLKKKYNITDERAALYEAAETWVDALKGRDFLGGSKPNLADLAVYGVLRPIRYLKSGRDMVENTRIGDWYSRMES
EVGVSARIQA
>PGSC0003DMT400025560
MRRVSRIAALYRAVDGAAAVEYRAGEGAAAVEVPQHRMSTAAQFSTSSNKSSAKSNWLFNNLLTDLSARTAAHAV
AGTMLFSVAATTLTDEVHAKEAVPPELRPKDLVLYQYEACPFCNKVKAFLDYYDLPYKIIEVNPISKKELKWSDY
KKVPVVLVDGEQMVNSSDIIDKLYEKVRSGDSTFDADEESKWRKWVDDHLVHMLSPNIYRNTSEALESFDYITSH
GNFSFTERITAKYAGAAAMYFVSKKLKKKYNITDERASLYEAAETWVDALKGRDFLGGSKPNLADLAVYGVLRPI
RYLKSGRDMVENTRIGDWYSRMESEVGVSARIQA
>PGSC0003DMT400025561
MRRVSRIAALYRAVDGAAAVEVPQHRMSTAAQFSTSSNKSSAKSNWLFNNLLTDLSARTAAHAVAGTMLFSVAAT
TLTDEVHAKEAVPPELRPKDLVLYQYEACPFCNKVKAFLDYYDLPYKIIEVNPISKKELKWSDYKKVPVVLVDGE
QMVNSSDIIDKLYEKVRSGDSTFDADEESKWRKWVDDHLVHMLSPNIYRNTSEALESFDYITSHGNFSFTERITA
KYAGAAAMYFVSKKLKKKYNITDERASLYEAAETWVDALKGRDFLGGSKPNLADLAVYGVLRPIRYLKSGRDMVE
NTRIGDWYSRMESEVGVSARIQA
>Sobic.006G032100.1
MRSLRAAQTLVSRSLLSARHLNGAASPATAAAAAGARWCATPAPVPPPRSPLPSSRVMPAGVAGAVSFSLTFATV
AVAEAKAKERPQPDLLPQNVVLYQYQACPFCNKVRAFLDYHDIPYKVVEVNPLSKKEIKWSDYKKVPILTVDGEE
LVDSSDIINILQRRINPDEPTNDEEAKWRRWVDEHLVHVLSPNIYRTTSEALESFDYIAKHGNFSFTERFAVKYA
GAVAMYMVSKKLKKKYNITDERASLYDAANTWIEALNGRDFLGGSKPNLADLAVFGVLRPIRYLRAGKDMVENTE
IGDWYQRMEVAVGEPSRIQDAEGN
>Thecc1EG012795t2
MRGASSFTSSVLSRTLATVHDRGAAAALQPRSLRVALFCSSRTNNSYTRLRWFSPVLTSFSRSSARAVSLGLAGA
VASVATAASVYAKEPPPAEIMPKDVVLYQYEACPFCNKVKAFLDYYDIPYKVVEVNPISKKEIKWSDYKKVPILM
VDGEQLVDSSAIIDKLSEKVLPKKMINSGANEDEETKWRRWVDNHLVHVLSPNIYRNTSEALESFDYITSNGNFS
FTEKITVKYAGAAAMYFVSKNLKKKYNITDERAALYEAAETWVDALNGRNFLGGSKPNLADLAVFGVLRPIRYLR
SGRDMVEHTRIGEWYSRMEEVVGESSRIKA
>Thecc1EG034263t1
MRRANGLPTLGRALSCGGASTEQRLVQAALISTCSSSSSNSHIFSQKLADLLGLSRPSLERGVAGTMLFSVAASS
LAQDAHAKEPPRSKKFLYNEVVLYQYEACPFCNKVKAFLDYNNIPYKIVEVNPISKKEIKWSDYKKVPILKVDGE
QMVDSSDIIDKLFHRINPDSSIPDDGEEKKWCQWVDNHLVHVLSPNIYRTTSEALESFDYITTHGNFSFTERLVA
KYAGAAAMYFVSKKLKKKYNITDERAALYEAAETWVDALKDRHYLGGSKPNLADLAVFGVLRPIRYLKSGKDMVE
HTRIGEWYTRMENTVGESARIKE
>GSVIVT01008442001
MRRASALASSALSRALLSAQDGATTTAVIQHRIFQAALYGTTTSAAAQSTRRRWYSTALYSLSGGSAKTVSLSIA
GALFSAAAATSLSEEVLAKEPPSPDLVPKDVVLYQYEACPFCNKVKAFLDYYDIPYKVVEVNPLSKREIKWSDYK
KVPILMVNDEQLVDSSAIIDKMSDKILPKKSADSASDDEERKWRQWVDNHLVHVLSPNIYRSTSEALESFDYITS
NGNFSFTEKLTVKYAGAAAMYFVSKKLKKRHNITDERAALYEAAETWVDGLNGREFLGGSKPNLADLAVFGVLRP
IRYLRSGRDMVENTRIGEWYTRMENAVGESSRIKA
>GSVIVT01009954001
MLGLNSASKLQRFPHKLAGHFPLPALAVSGTRLLSGLAQDVPAEDPVHPKKFLPNDVVLYQYEACPFCNKVKAFL
DYYDIGYKVVEVNPINKKEIKWSQYKKVPILTVDGEQMVDSSDIINKLFQRIHPEKYLDSVPDGDEERKWLGWVD
NHLVHVLSPNIYRSASEAIESFDYITTHGNFSFTERIIAKYGGAAAMYFVSKKLKKRHSITDERAALYGAAETWV
DALKGRKFLGGLEPNLADLAVFGVLRPIRHLKSGRDMVEHTGIGEWYSRMEAAVGESSRIKV
>Vocar20012624m
MSSAGALRRLPLLLLAQGQRANTIAHIAASSRLSFTGINDQRYGDSHRHRPGILLAAASLLGIGAAATVTTASAD
APTPIEVASDPYARPAEAHPLPSKITLYQYEVCPYCCKVRAFLDYYKLPYTVIEVNPLTKGELKWSSYKKVPVVK
LDEEVVVDSSAIMSRLAKDVAAAARPVTSLPPPPPPPPSPPPPSPPLPAKRSWWPFGSGGTTDPTNVGGGAKNPP
TVVPPPPPLPSSSSSSLEEEVLWRKWVDEKLVKVLTANIYRNWDESVETFKYITEQTGWSWGAREVARWAGAVMM
WQVGKRMPAKYGIEGDLRMALYDVANDFADNALRGRRFAGGDVAPNLADLAAFGVIRAVRQTGAFRDMMANTRLA
PWYAAMEGMVGESARINPGQGKPAVAA
>GRMZM2G084863_T01
MRSLRAAQTLVSRSLFSARHLSGAASPATAAAAGARWCAAPAPPPRSPLPSSRVMPAGVAGAVSFSLTFATVAAA
EAKAKERPQPDLLPQNVVLYQYQACPFCNKVRAFLDYHDIPYKVVEVNPLSKKELKWSDYKKVPILTVDGEQLVD
SSDIINILQSRINPNEELTSDEEAKWRRWVDEHLVHVLSPNIYRTTSEALESFDYIAKHGNFSFTERFAVKYAGA
AAMYMVSKKLKKKYNITDERASLYDAANTWIDALNGRDFLGGSKPNLADLAVFGVLRPIRYLRAGKDMVENTEIG
DWYRRMEDAVGEPSRIQDAEGN

GSTI class

>XP_001690194
MLVTGAPTARLTTRSDFLVATPRALGAALRRSVNHQLAGPASSRDVRRMSILKGIMQGFSKGGSAAPAAPQKSEP
TVLSFSDNAPSWQQLEEMVKWGDLHPMATRRTFGSSAPVRVKLYRDHASRCPYCHKVWMQLEEKRIPYEIEKINM
RCYGDKPASFMAKVPNGLLPVIELDGRVVTESAVIMNLLEQAFPDNKPLMPPQGTPERARADQLMRLERRFFSDW
LGWLTSDWNHARGKAQFEATVDAVAAELERAGGPYFLGSDISLVDITFAPMLERAAASLTYYKGFHLRGQGRWPA
VDRWFAAMESRPTYLGTRSDYYTHAHDLPPQLGGCAMHEEGEPLAAALDGTDGVHWRLPLPPLSASSLPEPYCPG
DNPPVDRLEAAAKLVRNHDAVVRFALRGPGQPGPRPVTAPLADPTAIPNTNYTAEADAALRHVAHALLAGVEAKQ
LSQHALQTSASGGLSGAAVVLAADYLRDRVGVPRDMRYPAARQLRAHLNWLIDSLTAAAPTSA
>jgi|ChlNC64A_1|58587|estExt_fgenesh3_pg.C_180030
MASPALQGPAASFLASIASSRRACGHAFRSAAFRSRSGSHPAARPSNGGSRGGGCSAMFAGLKKALGQAGTAAAV
PKPSLRAVPFLDTAPSWEELQAMVEAKQEALNAVPADLETGPASAYALRRTFGQPGEPRVKLYRDHAAWCPYCHK
IVLQLEEKQIPYLIEKINMRCYGDKPPEFLRKVPSGLLPVLEIDGQVITESAVIQQVLEQIQPDPPMLPPEGSAE
RQRAAQLMRLERRLFSDWLQWLCNGWGHESNRDQFCRTMDAIDKELREAPGPYFLSEFGLVDITFAPFLERIVSS
LLYYKGFVVRGEGRWEAVERWFDAMESRPAYLGFKSCHYTHVHDLPPQLGGCVSVPEAAAYAAAINGTDGRSWHL
PLPPLTSTSLPTAHAPGEEPEVDRLQAAARLVQNREAVTKFALRGVGQRGARPVSARLCDPTAVPGLEFMPEVDA
ALRHVAHALLVGVEAKQAGSAALGAAGASEGALDGSPVVPSLSYLRDRAERLRNRSCCDLLAMEFVEEGNTVKQF
SPCKTSSTNAWGQARGPRRLFGDGGAPAGSPTAVADQPAYATASQPSSATSSLAAALADLQVSSRQSSAGDLGDR
SRILAFAAAKASAAPAAHSRSSSGDELPGLAQEEGLCATVLIGAVRCFDSMVLKTLTPIKTRSRASSHDGAAASP
PVAYVETLVSPFGGNLKPKGPMGRPVVYHSRGSSGMGAPGAYNPYDLTAAALSSEFRSPAKSMYSPYPSSSRTAS
ASATPNQWGSGSYRGSAYRAAAQQQAMAQEEEPDYYVSVPGVRRQQSLSSSAKASQAAAAAAQQGEAQAAASGAA
SPTPLQRQPSTSAALLRGGSGGGAGLLRQASSGTASLGSGTAVHGFKGLFRSVIARGPSQ
>estExt_Genewise1.C_70307
MSCHLRWVLPSTVCPPLLLSRRSNLTQPLHERSSLRHSDRHSTLITRASIPSVPSMLGSLQSLLNQPGAKAGKQT
TTFSDNAPSWEALQQLVEEKGRQLNWQQPDLENGPTNPLALKRTFGQPGTPRVKLYRDHAAWCPYCQKVWLQLEE
KQIPYTLEKINMRCYGDKPPEYTAKVPSGLLPAMELDGQLIVESAEIMRILEEAFPDNKPLLPPKGSKERQRADS
LMRLERRLFSDWLSWLTTSWQAPSSHTIPLPHCMLALRTTREFIATMDAVDRELGAAGGPFFLGADLSMVDVVFS
PFLERIAASILYYKGLTVRGDGRWKNLERWFESMEARPAYIGTRSDFYTHVHDLPPQLGGCASVPDADEAAAAID
GVDGKSWQLPLPPLNATSFPEPYSPGEDPPLDRLRAAARLVGNHGAIAKFAARGCGKPGRRPVSAPLSDPTATAG
ENFLEAVDAGLRHVAHALLEGAHASSLRVGDGENLPGDAVVASAEYIRDRVGVPRDLPYPAARQLRAHLNWLIGS
LTA
>MicpuC2.EuGene.0000050480
MATATLPAAPSWDELKSAAAGTETGMKIAAEEELRAKGEGPAHVKAKLRLFGAKEEDVRLTFYRDHAGWCPYCQK
VWMMIEEKKIPCRIERINMRSYGDKPDWFLKKVPSGLLPVIELDGEMMTESLVIMQVLEREFPDIPMLPEGQFER
ANTLLRLERQLFSDWCGLVFRPSMPGPFGGGGRKTFEETFDKVDAALGEMDGPWFLGGDTPSIVDLQYVSHIERM
NASCLYWKGMNLRGQERWANIEKWFLAFEERPSYKACKSDYYTHVMDIPPQYGPGYSDSGKEVEDAQRAIDGDTT
WVLPVNLSANDLEPTPDSMNASDEAARHEAAYMLTANAKAITKFACRGKGEKGAKQFGAPLADPYAVPNLNYESK
VDELLRVTASLLLDGTAEPSGGKTSQDGADVAKCLAYLRDRIGVPRDMSYPAAMQLRAHLNYVMDAV
>EuGene.0100010816
MSSVAFAVHRAAPSRGTLVRRKPARSTPRSSVRSDAGGFDAMMTGLRRLRAMRDDQSSSASKTFTKLDDVSFEDE
APSWDELAAVADALKTSHGVPLAVDLENGPANPKALVRRFGTSDEPRVLFYRDHAAWCPYCEKIWMQLEEKRIPY
RVEKINMRCYGDKKRSFTAKVPSGMLPAMEIDGRLLTESASIATALEREFPNHKPLLAPPGSALRKRQDDLNRLE
RALFGRWMQWLTSSWMDGANRSGFEEALDAVEAELSVDTTGPYFCGDELTLIDITFAPFLERMAASLAYYKGFRL
EGNGGRWPNIDAWYEAMAKRTETYTHIKSDYYTHAHDLPPQLGGCEPNGDAEQVTFMNEIDGTDLSVGGDSPSGG
DNSWKLPLKPLKDPANVEPWWGPGEENAPLDRLEAATAVIGNADAVVRFAARGCGAVGTRPVAAPLADPTAVPNE
THVPAVDAALRRVCAELLSAEGARVGVKIGRVSSEGSAKGLLAGKETAASLAYLRDRVGVPRDMKYPAARQLRAH
LNWFIDGLAA
>XP_001419151
GPAHQDNALRLFGAREEDVRVTFYRDHAGWCPYCQKLWIMLEEKQIPYRVEKINMRSYGDKPKAFLDKIPSGLLP
VVEIDGNMITESLVIMQILEREFPERPTLPEDKFEAANVLLKLERQLFSDWCGLVFRPSMPGPLGARAGFEKTLD
KVDEALGSTEGPWFLGGESPSIVDFQYVSHVERMNASVLYWKGLQMRGTKRWANIERWLLAFEARPTYQATKSDY
YTHIMDIPPQYGPGYADKNAAVDEAVAVIGGEKSWRLPVSLSADGLEPLPESMNRGEEDAKHEAAYKLIANSANI
VKFACRGMGEPGRKHSEAKSVRKCLAYLRDRVGVPRDMSYPAAMQLRAHL
>KC119478
MRSPVGSLVACAIPVSRLSVSSISAAASRHQQLDTGKSIKLHLNTKLQSSLVKWKSRAAFEGRVSGEHRRCERVV
RLTAMEASVESSEGKKTTLASWDALAGIVGNDEDFPERLPNMKRRVFEDGQKLLLTFYRDNSSWCPYCQRVWLQL
EEKKIPYQVEKINMRCYGDKPAWFTKMVPSGLLPVIELDGRIITESMDIMILIEKRFPEFNPLLPAGGPELAAVN
SLLGLERRLAGAWMNRLRSSWPDMGAFENTMDKVNSALQTFGGPYFLGSKFSLVDAVYAPFLERTAASMPYWPGV
KVRGNDRWNAVNLWFDAMDSRPSYQAMKSDDFTHTHDLEPQIGPCYSAPAGKAYRDKIDGKDGGWELPLKPEETA

WGFDDGTGKGGAKEEAARTLISNHEKVVGFALRGVNDGDKHRDAMGHGLHLVANALVQGVENLDGFVTPDTLPPQ
VAVAAAYLRDRVGVPRDLSYPAARQLRAHLNWLIRSLGRPDL
>SELMODRAFT_450419
MASTAEVSSWEELSEKVSRLPGYNAAAAAARLPNVQQRDFGDTSIAQVVFYRDNSSWCPYCQRVWLQLEEKRIPY
KVEKINMNCYGPKPAWYLEKVPSGLLPALELKGRLLTESLDIMLILEEAFPERHPLLPPKGSPKANAVDGLLRLE
RLLAGAWLSRLRSFWTKPGQFERAMDDVDGALAKFGGPFFLGDTFSLVDAVYAPFLERIAASMPYWQGVMIRGET
RWPHLQAWFDAMDAKPSYQAVKSDDYTITHTLEPQIGACRALPAGEAYRAKVDGKDGSWDLPLKAEVTAWGREEG
NGNKAEAAQTLVDNNAAVVGFALRALNSDEKYRESVDLGFRCVAQALVDGVESATPPAVPQHFSREVAKAAAYLR
DKVGVPRDLSYPAARQLRAHLHWLVKALGSELYQ
>Vocar20010018m.g
MQSLIAPSRSVGPKQHNGPFGGRKSIQPRTSTSFPGLHSSHAPAGHRAARQGVKAMSILQGIMKGFSSGSGKAAE
KDRQPAATLSFSDNAPTWEQLADLVRAQHAEFGVDFWADPNEGPTHPLALRRLFGTSGPVRVKLYRDHAAWCPYC
HKVWLQLEEKRIPYVIEKINMRCYGDKPAAFMAKVPSGLLPVIELDGRVVTESAVIMTLLEDTFPEHKPLMPPAG
TPARQRADSLMRLERRLFSDWLNWLCSDWNYDRARRQFESTMDLVAQELEREGGPYFLGSELSLVDITFCPMLER
IAASLSYYKGLYIRGQGRWPAVDRWFEAMESRPTYLGTRSDFYTHAHDLPPQLGGCAMAAEGEPVAAAIDGTDGR
SWRLPLPPLTATSLPEPYSPGDNPPLDRLEAAAKLVRNHEAVVKFALRGPGQPGPRPVMAPLADPTAVPALEHTQ
EADAALRHVAHALLVGVEAKQVSPQALQTSSSAGLSGGAVVSAAEYLRDRVGVPRDMRYPAARQLRAHLNWLINN
LLPREQ

GSTH class

>KC119485
MGGCCQKQVREHVETSKQSAVVPSWASSPAYEDEGTRKVKLVGDVTCPYTQRVRIALLEKGVPVEASLVMPDDLR
GCKGEDLGTISPDGKFPVFQHGDRKITGSVDSILAYVEENFENSLLPNGLEKEVAQWVSYIRDHFSNVIEDALHN
GDPYAQDSLEARLNESLAHLDSGLELHHKEGKHFLGSKFTLVDVYLIPFLSLMDLITYIRGFAINPSYSRLCAYK
SAMCTFKCYKPVQVGTELSKTILVKSSIEKPSQPMVCMTLLQHKSILRHLDAFVKLVKELDSINRKTSLDSVQRT
IFGTRLKEVPKLYGRLLELLQEHAQMEERIIFPALETADQALTESAIKDHARDLPVMNGIREDIKGIMSLRQGNF
DHREALSALARRVEAFEVHAKEHFQGEEKEQLPLLEAAGFATNKQQAMLGQSFLVMEVSHSRLLPYLLEALKPHE
VHQYLGIMKSSTSDTENCCTLPKIAQALNNDEFEYVRKVALDRIPSLGPLQHTKLQYRPSMARG
>KC119486
MGGCCQKQVRQVVQSSKHSVVMPSRVSPPVCVQVNKDADEVKVKLVGDITCPYTQRVRIALLEKSVPAETSLVMP
EDLMGGKGDNLAAESPDGKFPVFQHGPHKITGSVDSILTYIEENFENPLLPSGLEKDVAQWVSYIRDIFSNVIED
ALHNGDPYAQDSLEARLNESLACLDDGLELHHKEGKHFLGSNFTLVDVYLIPFLSLMDLVTYIRGFEINSSFTRL
SAYKSAMCTFKCYKPVQMGTDLTKTILVQSSIDKPSQPMVGLALLQHKSIIWHLKAFVKLVKEFDGINRSPSMDS
MQRTVFVTRLKKLPKVYGRLVELLLEHAQMEERVIFPALETADQALTESALKDHARDLPVMNGIREDIKGIMSLR
QGNFDHREALTALAGRVEAFEVHVKEHFQEEEKEQLPLLEAAGFGTSKQQPMVAQAFLVMEASHSRLLPYLLEGL
KPHEVHQYLGIMKLSTADNAEKNSMLRRIAHVLNNEEFEYVRKVAIDRIPSLGGLPLTSVQGRPGMARG
>KC119487
MGACLQKHVREKVDSKSAAIVPSWASSPGYIDDDEGRVKLVGDVTCPYTQRVRIALLHKNIPVNASLVMPDDLRS
YRAENLSATSPDGKYPVFQHGDQKFTGSVDAMLAYIEETFENPPLLPNGLEKEVARWVSYIRDTFTVNVLDILHN
GDPYAQDSLEARLNESLARLDSGIKPQGKHFLGSKFTLVDVYLIPFLSLVDLVTYVRGFTIDSSYSRLLSYKFSM
CKFKCYKPVQVGVDLSKDILVKSSIERPPLPLVSFALLQHKSITWHLEAFVKLVKEFDGINRKPFVDTMQRTVFG
TRLKELPIAYGRLLEILQEHAQMEERIIFPALEMADQALTESALRDHSRDLPVMNGIREDIKGVMSLRLGNSDHR
EALSALVGRVEAFEVHVKEHFKEEEKEQLPLLQAAGFGTKKQQPMVGQALAIMESSHSRLLPYILEGLRPHEVHQ
YLGIMKSTSSDAEKEYLIPKIAHALNNDEYENVRKVAVDRVPALGGPQYTKGQSRPSMDQGDSWYSRRAVSFIH
>KC119488
MGICFPKAAQPKYKDEPLNPKPSAVVPTSSSPIYCEETPEPLAPSTPTHEHSYSEAESNLPRVQLIGDMLCPFTL
RVQIALQFKGVIVDPTWLTPVDLTNPKLVNASPNGKYPVLHYGLHKLSGSTDAMLDYIEETFEDPTLIPKPVETE
VMQWVAFIRDELTPIVGQLLYDGSPLVQQELESKLESCFMKLDSGIWEHGKQGRFFFGNQFTLVDVYLIPILLLV
DVAKFFRGIEISTLHSHLLAYSRAMHSFPNYAPVRVNTELLKGAVAKTLVERAPSPLIVMTVLQHRSILWHLERL
VVLADGLPVDKLANEDSGRRGVAGKQMQLLWKMYGRSLDLMQEHAQMEERVIFPAIESTEEGMSECALADHARDL
PVMNGIREDIKGVMALEQGCSDHLEGLQAVVKRLHILQENSVEHYHEEERDLLPQLNAAGLGNKKQEELVTQSIA
VMEETHGRLLPFFLQGLEPHEISQYFGLLHSLFDGGKSRVFTRMSFCLKNADEEFKEVCRVAQAKMTELANPSNE
KTVGL
>KC119489
MGICWPKLAPSKHKDELVSSKSSTVVPTSSSTTDYESSTHETISCEANRAEAKRNIPGVELIGDLLCPFTLRVLI
ALQFKGILVNPTWLTPADLKNPNRIIIGSPNGKYPVLQYGLHRLIGSTGTMLDYIEETFQDPPLIPRFIRDEVMK
WVAFIRDEFTPILVQLIYDGSHLEQHKMTLNLESAFAELNNGKCEHGKHGRYFLGNQFTLVDVYLIPSLLLVDVA
KFFRGITIGTVHSHLLSYSQAMHSFSNYAPVRLDLDSLKGAVAQILVERASSPLIVMTVLQHRSIMWHFKKLVVL
ADTLPENKPTVEVREFGRRGATAGKQMQLLWKMYGRLVVLMQEHAQMEEMVLFPAIDSTDEGMSGTALTDHARDL

PVMNGIREDIKGVMALEQGYSDYLGVQALATRLRVYQHNTVVHYREEERDLLPQLNLVDLGCKKQEDLVTQCFQI
MEESHERLLPFLLQGMEPYEVNQYLGLLQKSFVGGTSRLFTRNSHYLKNLDEEFGDVCQIARERISELVAP
>KC119490
MAPAKHKDEQLNSNPSAVVPASSSPTYCESSPSETAPSEMTYADAESNLPRVQLIGDVLCLFTLRVLIALQFKGV
VVDATWLTPADLTNPKVVNTSPDGKYPVLKYGHHKIVHSTDVMLEYIEETFQDPTLIPSPIRSEVMNWVAFIRDE
FTPIVGQLVYDGSPLVQQELRPNLESAFAKLDSGKLVHGKQGRFFFGNHFTLVDVYLIPALLLVDVAKFFRGIEI
GAAHPHLLSYSLGLHSFPNYAPVRVDLELLKGAIAKVLDERAPSPLIVMTVLQHRSISWHLEKLLALADELLVNK
LEIEVGEFGRRGAGKRMQLLWKMYGRLVDLMQEHAQIEERVIFPAIDCTEEGMSESALMDHARDLPVMNGIREDI
KGVMALEQGGSDHLEGLQALAARLRVYQKNVVEHYHEEERDLLPQLNIADIGRDKQEELVIQCFGVMEESHGRLL
PFLLQGLERHEVNQYLGLFQKSFEGGNSRLFMRMSSCLENADEEFNEVCKVAQERMGEVMAPAKDNA
>KC119491
MGICFPKAAHSKYKDEPLNPKPSAVVPTSSSPIYCESTPEPSTAPSIPTDEQSYSEAESNLPRVQLIGDMLCPFT
LRVQIALQFKGVVVDPTWLTPADLTNPKLVNASPTGKYPVLHYELHKLSGSTDAMLDYIEETFEVPTLIPKPVEA
EVMQWVAFIRDELTPIVGQLLYDGSPLVQQELEPKLESCFMKLDSAIWEHGKQGRFFFGNQFTFVDVYLIPILLL
VDVAKFFRGIEISTLHSHLLAYSRAMHSFPNYSPVRMNTELLKGAVAKTLVERAPSPLIVMTVLQHKSILSHLER
LVALADGLPVDKLANEDSGRRGVAGKQMQLLWKMYGRLLDLMQEHAQMEERVIFPAIESTEEGMSECALADHARD
LPVMNGIREDIKGVMALEQGCSDHLEGLQAVVKRLHALQENSVEHYHEEERDLLPQLNAAGLGNKKQEELVSQSI
AVMEETHGRLLPFFLQGLEPHEISQYIGLLHSSCDGGKSRVFTRISFCLKNADDEFKEVCMAAQGIISELATPSI
DKAAGSYCLLQE
>KC119492
MGICCPKLAPSRHGDDTLSSKPSTVVPTSSCPTLRETTHNMTTYAEAKRIPRVQLIGDKFCPFTLRVLIALQFKG
IHVDAAWLTPEDLRNPNRIITASPNGKYPVLQNGLHRLISSTSAMLDYIEETFQYPTLIPSHIKGEVIKWASFIR
DEFTPTLAQLIHDGNPVVQQRMTQDLKSAFAELNNGKRQHGKQGRFFSGSQFTLVDVYLIPTLLQVDVAKFFRGV
SIDTVNSHLLSYSRAMHSFPNYAPVRVDMDLLKEAVAKLLFERDSLPFVVMTMLQHRSILRHMKKLVVLADELLV
NKLASEVGQFGTLGAVPGKHVHLLWKMDGRLVDLMQEHAQMEERVLFPAIDSRGQGMTGTALTDHARDLPVMNGI
REDIKGVMALERDCADYLEGLQALSTRLRAFQENTVDHYHEEERDLLPKLNVIDLATKKQDELMVQCFGIMEDSH
GRLLPFLLQGMEPHEVIQYLELLKKSFEGGKSRLFTGILFCLENADEEFKDVCKTAQERIIEMMALENAKTAGS
>SELMODRAFT_46109
VSKAIDERTCPAVASLCLLQHRSILFHVEKVVKISEELAATKVNSSTISPVKAGLAMKIKKLSSEYLRLVELMQE
HAQMEERTMFPVLENADKGLTELVHADHARDLPIMNGIREDLKSVLALQQGSCVHNEALVALATRLKVFQVLLGD
HFDEEERDILPLL
>SELMODRAFT_420348
CKELEDRSQGAPAVAYTVLQHLSIVRHLERLVDVSDGVRDFYHGQGKSKQKKKSKPQVAQRGSTMIQVGIATKLK
NAARDYDRLLALMQEHAQMEEKVIFPALEKAERGVTKFANEDHARDFPMMNGVREDIKTVMVLEQGSFSHIEALS
ALADKLRTLKGSTVKHFLDEERELLPSL
